# Cdk1-phosphorylated Nur77 accumulates at the centrosome during mitosis to regulate the Cep192–PLK1 signaling axis

**DOI:** 10.1038/s41419-026-08870-3

**Published:** 2026-06-03

**Authors:** Guobin Xie, Qiqiang Wang, Mingxuan Du, Yuqi Zhou, Ziwen Chen, Shuangzhou Peng, Hualan Zhou, Xiaoli Zhang, Qi Cai, Gulimiran Alitongbieke, Xiaohui Chen, Jiajin Yi, Xiaoxuan Ye, Hongli Wang, Xiaohong Ye, Lin Xu, Boning Niu, Haishan Wang, Yihong Ke, Yang Xu, Hu Zhou, Ying Su, Xiao-kun Zhang

**Affiliations:** 1 School of Pharmaceutical Sciences, Fujian Provincial Key Laboratory of Innovative Drug Target Research, Xiamen University, Xiamen, China; 2 NucMito Pharmaceuticals Co. Ltd., Xiamen, China; 3 Department of Clinical Laboratory, The Fifth Affiliated Hospital of Sun Yat-sen University, Zhuhai, China

**Keywords:** Mitosis, Phosphorylation, Nuclear receptors, Small molecules, Targeted therapies

## Abstract

The orphan nuclear receptor Nur77 is a multifunctional regulator involved in diverse cellular processes, including proliferation, survival, and apoptosis, through both transcription-dependent and -independent mechanisms. This regulatory complexity underscores the need for mechanistic studies in defined biological contexts. Here, we uncover a previously unrecognized non-genomic function of Nur77 at the centrosome that promotes mitotic progression in cancer cells. We show that Nur77 is phosphorylated at threonine 143 by cyclin-dependent kinase 1 (Cdk1), leading to its accumulation at the centrosome, where it binds the scaffold protein Cep192. This interaction is critical for maintaining centrosome integrity in tumor cells and facilitating the recruitment of Polo-like kinase 1 (PLK1), a key driver of centrosome maturation. Notably, Cdk1-mediated phosphorylation of Nur77 is aberrantly elevated in tumors, contributing to malignant proliferation through its mitotic role. Depletion of Nur77 or treatment with NMA39, a novel small-molecule Nur77 modulator that disrupts the Nur77-Cep192 interaction, results in mitotic arrest and cell death in tumor cells. These findings reveal a tumor-selective mitotic function of Nur77 and establish a mechanistic rationale for targeting phospho-Nur77 signaling as a cancer vulnerability.

## Introduction

Nur77 (also known as TR3, NGF1-B, or NR4A1) is an orphan nuclear receptor belonging to the NR4A subfamily of transcription factors. It plays diverse roles in cell proliferation, inflammation, immunity, autophagy, and apoptosis [1–7]. Structurally, Nur77 features a conserved central DNA-binding domain (DBD) and a C-terminal ligand-binding domain (LBD), along with a unique N-terminal A/B domain containing an intrinsically disordered region (IDR) enriched in phosphorylation sites [8–11]. This structural plasticity enables Nur77 to integrate diverse signaling cues and regulate gene expression through monomeric, homodimeric, or heterodimeric binding to DNA response elements [2, 10, 12]. Beyond its classical transcriptional functions, Nur77 exerts non-genomic roles in the cytoplasm and mitochondria, particularly in modulating apoptosis and mitophagy [13–17]. Although its LBD lacks a conventional ligand-binding pocket, small molecules have been identified that can bind alternate sites to modulate its activity [6, 18–20]. Nur77 is frequently overexpressed and posttranslationally-modified across multiple malignancies and has been implicated in tumor progression, immune evasion, and therapy resistance [1, 12, 21].

Mitosis is a tightly regulated phase of the cell cycle in which replicated chromosomes are distributed equally into two daughter cells [22]. This process depends on key regulators such as cyclin-dependent kinase 1 (Cdk1), Polo-like kinase 1 (PLK1), and Aurora kinases [23–27]. At the heart of mitotic control is the centrosome, the primary microtubule-organizing center, composed of a pair of centrioles and surrounding pericentriolar material (PCM). Centrosome maturation involves recruitment of PCM components and γ-tubulin ring complexes (γ-TuRC), enabling bipolar spindle formation essential for chromosome alignment and segregation [28, 29]. Among centrosomal scaffold proteins, Cep192 plays a critical role by recruiting PLK1, which in turn phosphorylates Cep192 to reinforce γ-TuRC anchoring and centrosome function [30, 31]. Cep192 mutants lacking canonical PLK1-binding motifs still permit partial PLK1 recruitment, suggesting that additional regulatory components likely participate in centrosomal PLK1 localization [30, 31].

Nur77 is rapidly induced by mitogenic stimuli in cancer cells [32] and has been associated with abnormal mitotic phenotypes when genetically deleted [33]. However, its role in mitosis remains poorly understood. Here, we identify a tumor-selective, non-genomic role for Nur77 at the centrosome during mitosis. We show that Nur77 is phosphorylated at threonine 143 by Cdk1/cyclin B1 and accumulates at the centrosome, where it interacts with the scaffold protein Cep192 to recruit PLK1. This Nur77–Cep192–PLK1 axis is required for centrosome maturation and mitotic progression in tumor cells. Importantly, phosphorylation of Nur77 at Thr143 is elevated in human and murine tumors, while Nur77 is dispensable for mitosis in normal tissues. As a therapeutic strategy, we identify NMA39, a Nur77-targeting compound that disrupts this mitotic axis, selectively impairing tumor cell division and promoting mitotic cell death in vitro and in vivo. Together, these findings reveal a transcription-independent, tumor-specific function of Nur77 as a mitotic scaffold and define a previously unrecognized vulnerability in cancer mitosis.

## Results

### Nur77 interacts with PLK1 during mitosis via its N-terminal A/B domain

**Fig. 1:**
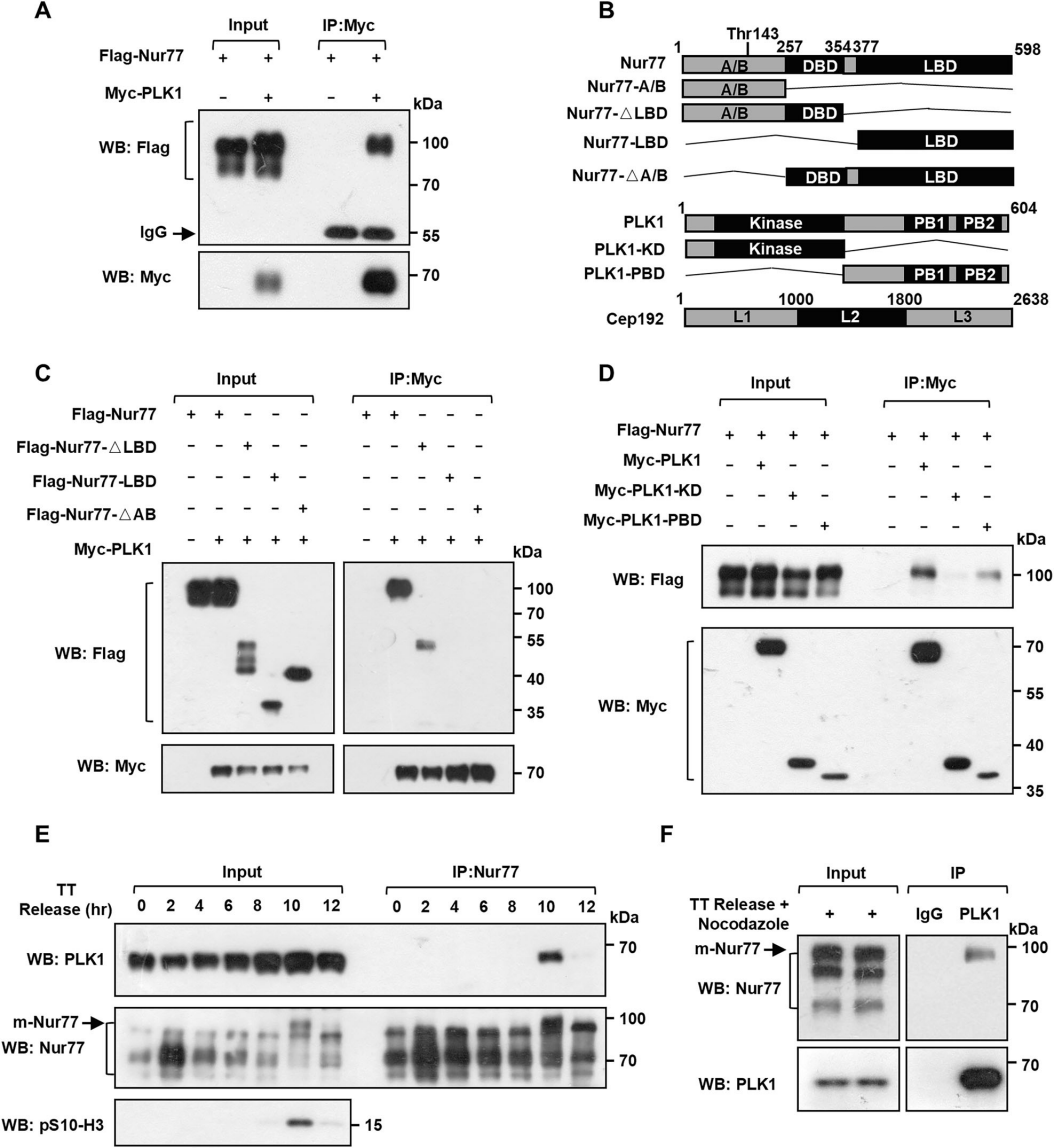
Nur77 interacts with PLK1 during mitosis. **A** Interaction of transfected Flag-Nur77 with Myc-PLK1 was analyzed by co-immunoprecipitation (CoIP) assay in HeLa cells. Immunoprecipitates were analyzed by Western blot (WB). **B** Schematic representation of Nur77, PLK1, Cep192 and their mutants. AB, N-terminal A/B domain; DBD, DNA-binding domain; LBD, ligand-binding domain; KD, kinase domain; PB, polo-box; PBD, polo-box domain. **C** CoIP analysis of the interaction of Myc-PLK1 with the indicated Flag-tagged Nur77 mutants in HeLa cells. **D** CoIP analysis of the interaction of Flag-Nur77 with the indicated Myc-tagged PLK1 mutants in HeLa cells. **E** Interaction of endogenous Nur77 with PLK1 was analyzed by CoIP assay after HeLa cells were released from the TT block for the indicated time. **F** Interaction of endogenous Nur77 with PLK1 during mitosis was analyzed by CoIP after HeLa cells were released from a double thymidine block (TT block, 2 mM) followed by nocodazole (50 ng/mL) treatment for 10 h. Data represent at least three independent experiments.

Nur77 is known to interact with diverse intracellular proteins to exert its regulatory functions in cell growth, apoptosis, and metabolism [6, 11, 21]. To identify novel mitotic partners of Nur77, we performed stable isotope labeling with amino acids in cell culture (SILAC) followed by immunoprecipitation and quantitative proteomics (Supplementary Fig. 1A, B) [34]. This screen identified Polo-like kinase 1 (PLK1), a master regulator of mitosis, as a high-confidence interactor of Nur77 (Fig. 1A and Supplementary Fig. 1C).

To map the interaction domains, we generated Nur77 truncation constructs and assessed their binding to PLK1. The N-terminal A/B domain of Nur77 was sufficient to bind both exogenous and endogenous PLK1, whereas the C-terminal ligand-binding domain (LBD) failed to do so (Fig. 1B, C and Supplementary Fig. 1D). Conversely, domain mapping of PLK1 revealed that its C-terminal polo-box domain (PBD), but not the N-terminal kinase domain (KD), mediated the interaction with Nur77 (Fig. 1D), consistent with the PBD’s known role in docking partner proteins [25]. These data indicate that Nur77 interacts with PLK1 through its A/B domain, specifically binding the PBD of PLK1.

A time-course experiment in synchronized HeLa cells revealed that the Nur77–PLK1 interaction emerged around 10 h post-release from a double thymidine (TT) block [35], coinciding with the peak of histone H3 Ser10 phosphorylation (pS10-H3), a mitotic marker (Fig. 1E). This interaction was absent in interphase cells, indicating that Nur77–PLK1 association is mitosis-specific. Notably, we observed that a strikingly modified form of Nur77 (m-Nur77) exhibited reduced mobility on SDS-PAGE during mitosis, suggesting that mitotic Nur77 undergoes post-translational modification (Fig. 1E). Interestingly, co-immunoprecipitation (Co-IP) assays demonstrated that m-Nur77 specifically interacts with PLK1 in mitotic cells synchronized by TT block followed by nocodazole treatment (Fig. 1F) [36], consistent with the interaction between the upshifted band of the N-terminal domain (∆LBD) and PLK1 (Fig. 1C). Reverse Co-IP assays further confirmed that endogenous Nur77 and PLK1 interacted during mitosis in HeLa and MCF-7 cells (Supplementary Fig. 1E, F). Given that Nur77 can heterodimerize with RXRα, a known PLK1 interactor during mitosis [37], we tested whether RXRα mediates Nur77’s association with PLK1. Depletion of RXRα had no effect on Nur77–PLK1 binding (Supplementary Fig. 1G), indicating that Nur77 interacts with PLK1 independently of RXRα. Altogether, these data demonstrate that PLK1 is a new Nur77-binding protein during mitosis.

### Cdk1 phosphorylates Nur77 at Thr143 to enable PLK1 binding during mitosis

**Fig. 2:**
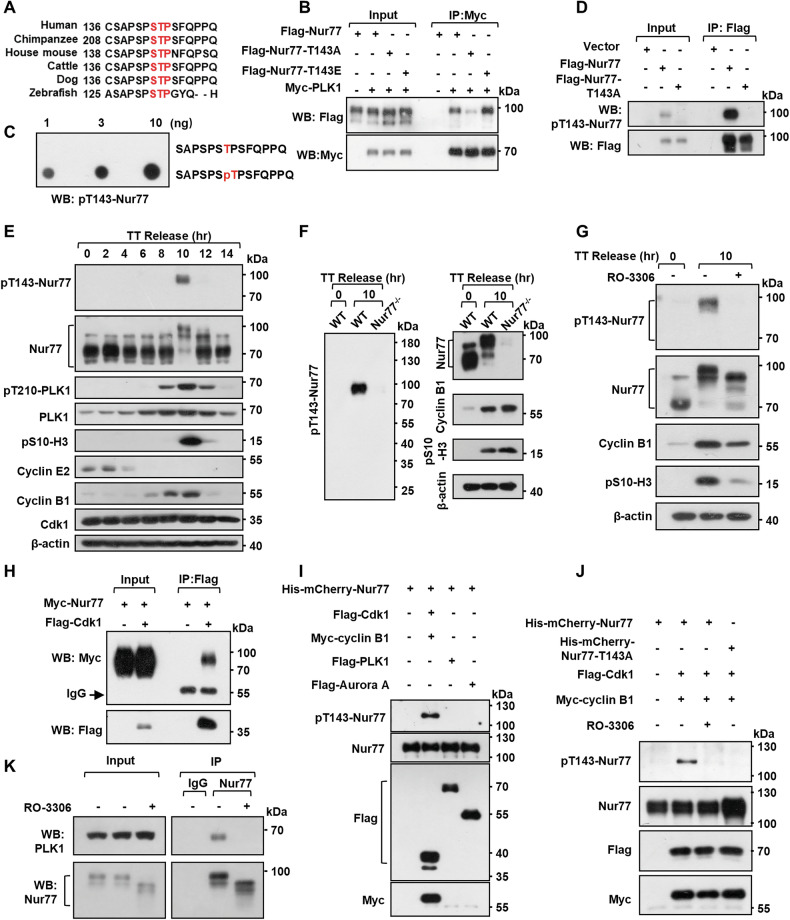
Nur77 is phosphorylated at Thr143 by Cdk1 during mitosis. **A** Comparison of the N-terminal sequence of Nur77 protein harboring PLK1-binding motif from different species. The unique PLK1 binding motif is highlighted. **B** CoIP analysis of the interaction of PLK1 with the indicated Nur77 mutants in HeLa cells. **C** Characterization of pT143-Nur77 antibody by peptide spot arrays. **D** WB analysis of Flag-Nur77 or Flag-Nur77-T143A protein immunoprecipitated from mitotic HeLa cells using pT143-Nur77 antibody. **E** HeLa cells released from TT block for the indicated time were analyzed by WB. **F** Parental HeLa cells (WT) and Nur77-depleted HeLa cells (Nur77^–/–^) released from TT block for 0 h (G1/S phase) and 10 h (mitotic phase) were analyzed by WB. **G** HeLa cells released from TT block for the indicated time were treated with Cdk1 inhibitor RO-3306 (10 μM) for 15 min and analyzed by WB. **H** CoIP analysis of the interaction of Nur77 with Cdk1 in HeLa cells. **I** In vitro kinase assay. His-mCherry-tagged Nur77 protein incubated with Flag-Cdk1/Myc-cyclin B1 or Flag-PLK1 or Flag-Aurora A was analyzed by WB. **J** In vitro kinase assay. His-mCherry-tagged Nur77 or mutant was incubated with Flag-Cdk1/Myc-cyclin B1 followed by RO-3306 (10 μM) treatment for 30 min and analyzed by WB. **K** Interaction of endogenous Nur77 with PLK1 was analyzed by CoIP after mitotic HeLa cells were treated with or without 10 μM RO-3306 for 30 min. Data represent at least three independent experiments.

PLK1 recognizes binding partners that are “primed” by phosphorylation of specific docking motifs (Ser-pSer/pThr-Pro), where the Ser/Thr residue preceding Pro is phosphorylated [25, 38, 39]. Full-length sequence analysis of Nur77 identified a single conserved PBD-docking motif centered on threonine 143 (Thr143) in its N-terminal A/B domain (Figs. 1B, 2A). This motif is evolutionarily conserved across vertebrates (Fig. 2A), suggesting functional importance. Mutational analysis confirmed that substitution of Thr143 with alanine (T143A) significantly impaired PLK1 binding, while a phosphomimetic substitution (T143E) enhanced the interaction (Fig. 2B), consistent with classical behavior of PLK1 partners [40, 41].

To test whether Nur77 is phosphorylated at Thr143 during mitosis, we generated a phospho-specific antibody against phosphorylated Thr143 (pT143-Nur77). This antibody recognized the phosphorylated peptide in dot-blot assays (Fig. 2C) and selectively detected wild-type but not T143A-mutant Nur77 in mitotic cells (Fig. 2D), confirming its specificity. Using this antibody, we observed Thr143 phosphorylation in mitotic HeLa cells released from thymidine block (10 h) (Fig. 2E) and after nocodazole treatment (Supplementary Fig. S2A). Notably, Thr143 phosphorylation was evident across several tumor cell lines but undetectable in non-tumorigenic MCF-10A cells (Supplementary Fig. 2B, C). Nur77 depletion by CRISPR/Cas9-based genome editing markedly reduced the pT143 signal (Fig. 2F), and the characteristic mitotic upshift of wild-type Nur77 was absent in the T143A mutant (Supplementary Fig. 2D). The upshift was reversed by phosphatase treatment but not by inhibitors of ubiquitination, sumoylation, or PARylation, nor was it reversed by Peptide N-Glycosidase F (PNGase F), confirming that it reflected phosphorylation (Supplementary Fig. 2E–H).

Given that the minimal Cdk1 consensus motif (S/T-P) overlaps with PLK1-binding motifs [42, 43], we hypothesized that Cdk1 is responsible for Thr143 phosphorylation. Indeed, treatment with the Cdk1 inhibitor RO-3306 or the pan-CDK inhibitor Flavopiridol abolished Thr143 phosphorylation, whereas the Cdk4/6 inhibitor Palbociclib had no effect (Fig. 2G and Supplementary Fig. 2I). Conversely, inhibition of PLK1 with BI2536 did not affect Thr143 phosphorylation, indicating that PLK1 is not the priming kinase (Supplementary Fig. 2I). Other kinases known to modify Nur77, including p38, JNK, AKT, and GSK3β, were also ruled out (Supplementary Fig. 2J, K), suggesting they are not responsible for Thr143 phosphorylation of Nur77 during mitosis [44–47].

Co-IP assays revealed that Nur77 associates with Cdk1 (Fig. 2H), potentially via mitotic cyclin docking motifs (LXF) within Nur77 (Supplementary Fig. 2L) [48]. In vitro kinase assays confirmed that Cdk1 phosphorylates Nur77 at Thr143, as phosphorylation was detected in wild-type but not T143A-mutant Nur77, and was abolished by RO-3306 treatment (Fig. 2I, J). Other mitotic kinases, including PLK1 and Aurora A, did not phosphorylate Nur77, reinforcing Cdk1 specificity. Importantly, pharmacological inhibition of Cdk1 disrupted the Nur77–PLK1 interaction (Fig. 2K), phenocopying the T143 A mutation.

Collectively, these findings establish that Cdk1/cyclin B1 directly phosphorylates Nur77 at Thr143 during mitosis, and this phosphorylation is required for subsequent binding to PLK1, a critical priming event in Nur77’s mitotic function.

### Phosphorylated Nur77 localizes to the centrosome and interacts with PLK1 during mitosis

Nur77 action is tightly coupled to its subcellular localization, which dictates its role in transcriptional regulation, apoptosis, and mitophagy [6, 21, 49]. To examine the spatial dynamics of Nur77 during mitosis, we performed immunofluorescence staining in HeLa cells. While Nur77 localized predominantly to the nucleus during interphase, it relocalized to centrosomes at the onset of mitosis, with peak accumulation during prometaphase and metaphase, followed by dispersal in telophase (Supplementary Fig. 3A). This centrosomal localization was confirmed using an independent Nur77 antibody (Supplementary Fig. 3B) and was abrogated upon Nur77 depletion (Supplementary Fig. 3C, D), validating specificity. Notably, centrosomal accumulation of Nur77 was also observed in primary mouse breast tumor cells (Supplementary Fig. 3E, F), indicating conservation across species and cell types.

**Fig. 3:**
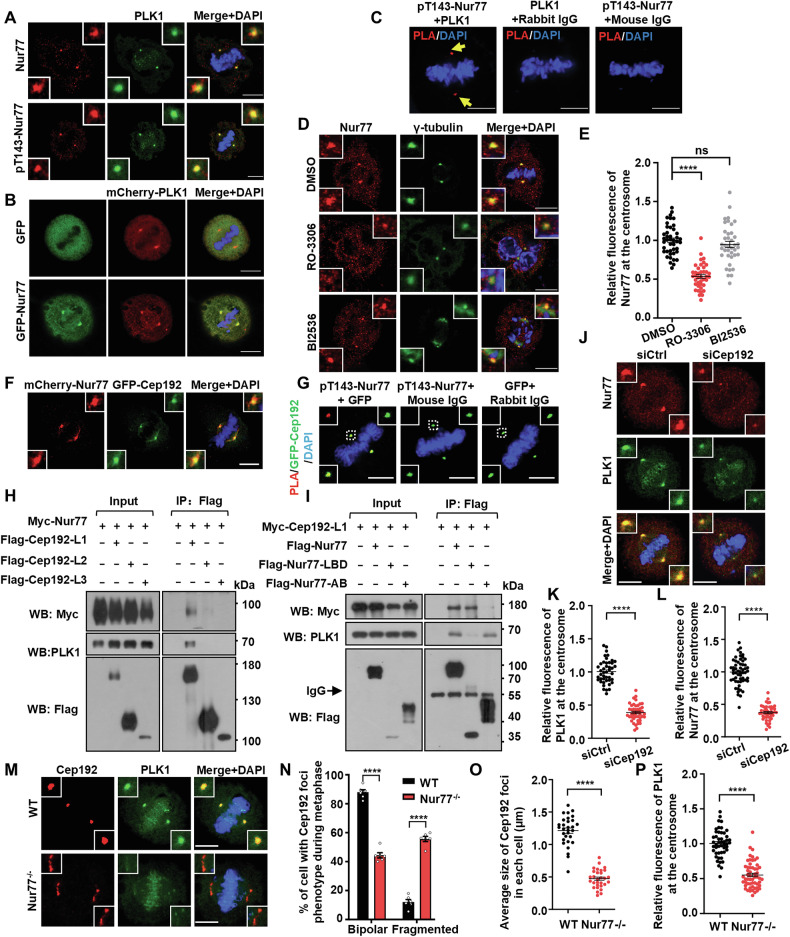
Centrosomal localization of pT143-Nur77 modulates Cep192-PLK1 signaling axis. **A** Colocalization of Nur77/pT143-Nur77 and PLK1 at the centrosome during mitosis revealed by immunostaining in HeLa cells. Nur77/pT143-Nur77 (red), PLK1 (green), and DAPI (blue). Scale bar, 10 μm. **B** Mitotic HeLa cells transfected with the indicated expression vector were analyzed by confocal microscopy. DAPI (blue). Scale bar, 10 μm. **C** In situ pT143-Nur77 interaction with PLK1 at the centrosome in metaphase HeLa cells was revealed by PLA assay. Scale bar, 10 μm. **D** Mitotic HeLa cells released from TT block for 10 h were treated with 10 μM RO-3306 or 0.25 μM BI2536 for 30 min and stained for Nur77 (red), PLK1 (green), and DAPI (blue). Relative fluorescence intensity of Nur77 at the centrosome during metaphase was calculated **E** (independent cells measured from left to right: 43, 40, 39; ****p < 0.0001, ns *p* = 0.2763; two-tailed unpaired t-test)). Scale bar, 10 μm. **F** Colocalization of transfected mCherry-tagged Nur77 and GFP-Cep192 at the centrosome in metaphase HeLa cells was revealed by confocal microscopy. DAPI (blue). Scale bar, 10 μm. **G** In situ pT143-Nur77 interaction with GFP-Cep192 at the centrosome in HeLa cells during metaphase was detected by PLA assay. Scale bar, 10 μm. **H** Interaction of Nur77 with the indicated Cep192 mutants in mitotic HeLa cells released from TT block for 10 h was analyzed by CoIP. **I** CoIP analysis of Cep192-L1 interaction with the indicated Nur77 mutants in mitotic HeLa cells. **J**–**L** HeLa cells transfected with control siRNA or Cep192 siRNA were synchronized at mitosis by TT block and stained for Nur77 (red), PLK1 (green), and DAPI (blue). Relative fluorescence intensity of PLK1 (**K**) and Nur77 (**L**) at the centrosome during metaphase was calculated respectively (left graph, *n* = 44, and 45 cells, respectively; right graph, *n* = 56, and 45 cells, respectively; ****p < 0.0001; two-tailed unpaired *t*-test). Scale bar, 10 μm. **M**–**P** Mitotic HeLa cells with Nur77 depletion were examined by immunostaining. Cep192 (red), PLK1 (green) and DAPI (blue). Percentage of cells with fragmented Cep192 (**N**), size of Cep192 foci (**O**) and relative fluorescence intensity of PLK1 at the centrosome (**P**) were calculated respectively (left graph, *n* = 200 cells across six experiments; middle graph, *n* = 30 cells per group; right graph, *n* = 48, and 56 cells, respectively; ****p < 0.0001; two-tailed unpaired *t*-test). Scale bar, 10 μm. All quantitative data in this figure are presented as mean values ± SEM. Data represent at least three independent experiments.

Co-staining of PLK1 and Nur77 revealed colocalization at centrosomes in mitotic tumor cells (Fig. 3A and Supplementary Fig. 3G). Moreover, the phospho-Nur77 (pT143) signal was concentrated at the centrosome and overlapped with PLK1 staining (Fig. 3A and Supplementary Fig. 3H, I). Similar centrosomal colocalization was observed in HeLa cells co-expressing GFP-Nur77 and mCherry-PLK1 (Fig. 3B). To confirm the interaction between Nur77 and PLK1 at centrosomes, we performed in situ proximity ligation assays (PLA) [50], which revealed punctate PLA foci specifically at centrosomes in mitotic cells (Fig. 3C), consistent with a direct interaction.

We next tested whether Cdk1-mediated phosphorylation at Thr143 regulates centrosomal targeting of Nur77. Inhibition of Cdk1 with RO-3306, but not of PLK1 with BI2536, significantly reduced centrosomal localization of Nur77 (Fig. 3D, E and Supplementary Fig. 3J). Similarly, cells expressing the non-phosphorylatable Nur77-T143A mutant showed impaired centrosomal localization (Supplementary Fig. 3K), indicating that Thr143 phosphorylation is necessary for centrosomal accumulation.

To identify the domain responsible for centrosome targeting, we analyzed the localization of Nur77 truncation mutants. Full-length Nur77 and its C-terminal LBD localized to centrosomes, whereas the A/B domain alone, despite being sufficient for PLK1 binding, failed to do so (Supplementary Fig. 3L). These results indicate that centrosomal localization of Nur77 is mediated by its LBD, and that this targeting is enhanced by Cdk1-dependent phosphorylation at Thr143.

Together, these findings demonstrate that Cdk1-phosphorylated Nur77 accumulates at the centrosome during mitosis, where it directly interacts with PLK1, suggesting a critical role in coordinating centrosomal signaling.

### Phosphorylated Nur77 mediates the Cep192-PLK1 signaling axis at the centrosome

Given that Nur77 localizes to centrosomes via its LBD, we sought to identify centrosomal scaffold proteins that mediate this targeting. Cep192 is a key scaffold component of the PCM and is essential for centrosome maturation through recruitment of PLK1 and γ-tubulin ring complexes [30, 51]. We hypothesized that Cep192 might anchor Nur77 to centrosomes. Immunofluorescence staining showed colocalization of mCherry-tagged Nur77 with GFP-tagged Cep192 at centrosomes in mitotic cells (Fig. 3F). This colocalization was supported by in situ PLA, which confirmed a direct interaction between endogenous phospho-Nur77 and Cep192 during mitosis (Fig. 3G). To further characterize the interaction, we generated three truncation mutants of Cep192 (L1, L2, L3; Fig. 1B). Co-IP assays revealed that Nur77 selectively interacted with the L1 region, which is also responsible for PLK1 binding and PCM recruitment [30], but not with L2 or L3 (Fig. 3H). Endogenous PLK1 was co-precipitated with Cep192-L1, confirming its dual docking function. Notably, mutating PLK1-binding residues Thr44 and Ser995 in Cep192-L1 (Cep192-L1-AA) abrogated PLK1 binding without affecting Nur77 association, indicating that Nur77 and PLK1 bind distinct sites within Cep192-L1 (Supplementary Fig. 3M). Domain mapping further revealed that the LBD of Nur77 bound Cep192-L1 but not endogenous PLK1, while its A/B domain displayed the opposite binding specificity (Fig. 3I). Neither knockdown of PLK1 nor the kinase-dead mutant (PLK1-K82R) noticeably disrupted the Nur77–Cep192 interaction, suggesting that PLK1 and its kinase activity may not be strictly required for their association (Supplementary Fig. 3N, O). Nur77 interaction with Cep192 was also independent of RXRα, as RXRα knockdown or overexpression did not alter Cep192 binding (Supplementary Fig. 3P, Q). The role of Cep192 in Nur77 centrosomal localization was further supported by data showing that Cep192 knockdown inhibited the centrosomal recruitment of both PLK1 and Nur77 (Fig. 3J–L and Supplementary Fig. 3R). Taken together, our results revealed that Cep192 is responsible for centrosomal localization of Nur77, and there is a unique mode of Nur77 action at the centrosome, in which Nur77 interacts with Cep192 through its C-terminal LBD domain and PLK1 via its N-terminal A/B domain.

Given that Nur77 interacts with Cep192 and PLK1 at the centrosome, we explored its role in the Cep192-PLK1 signaling cascade. CRISPR/Cas9-mediated knockout of Nur77 caused fragmentation of Cep192 foci, reduced size of Cep192 foci, and diminished PLK1 accumulation at the centrosome (Fig. 3M–P and Supplementary Fig. 3S). Similar phenotypes were observed with siRNA-mediated Nur77 knockdown (Supplementary Fig. 3T). Immunostaining with centrin1, a centriole marker, confirmed that these changes reflected PCM disorganization rather than the overduplication or disengagement of centrioles (Supplementary Fig. 3U) [52]. Nur77 depletion also impaired the interaction between Cep192 and PLK1, which was rescued by reintroduction of wild-type Nur77 (Supplementary Fig. 3V). To further study this mechanism, we co-expressed GFP-Cep192 with Flag-tagged Nur77 variants in cells. Cep192 formed cytoplasmic condensates resembling biomolecular droplets (Supplementary Fig. 3W), and co-expression with wild-type Nur77, especially the phosphomimetic T143E mutant, enhanced condensate size and PLK1 recruitment (Supplementary Fig. 3W–Y). In contrast, the T143A mutant failed to promote PLK1 recruitment, suggesting that Nur77 facilitates PLK1 incorporation into centrosomal scaffolds via a phosphorylation-dependent mechanism. Thus, Nur77 may serve as an additional regulator to maintain the structural integrity of Cep192 foci at the centrosome and promote the recruitment of PLK1 to the centrosome during mitosis.

### Phosphorylated Nur77 promotes centrosome maturation and mitotic fidelity in tumor cells

**Fig. 4:**
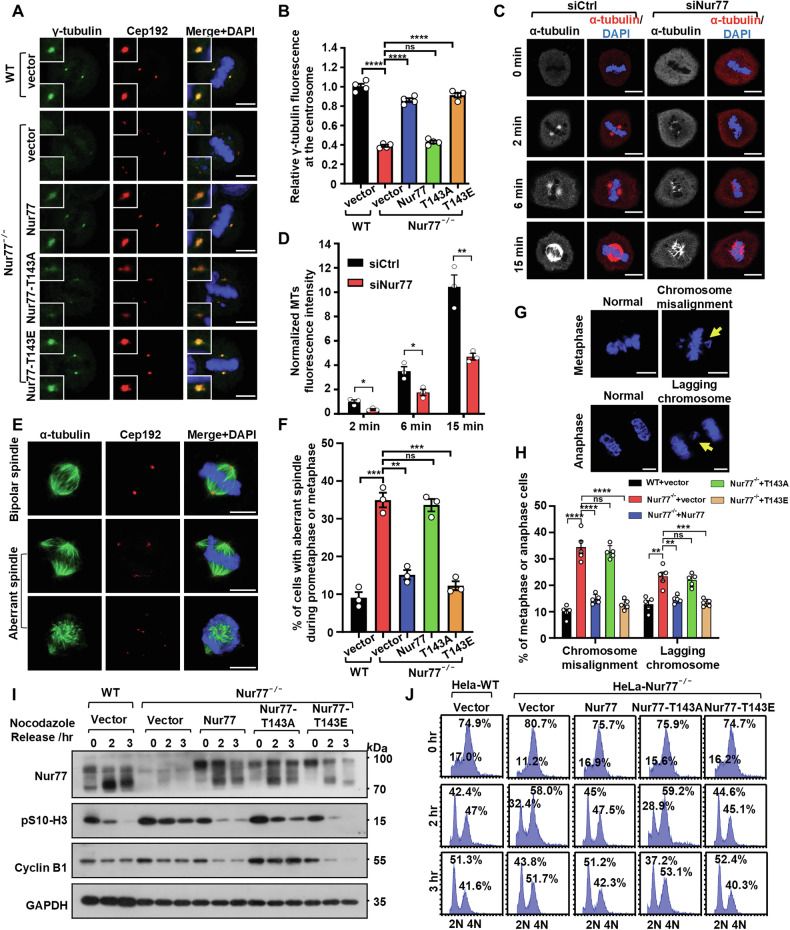
pT143-Nur77 modulates centrosome maturation and mitotic progression. **A**, **B** Thr143 phosphorylation of Nur77 modulates **c**entrosomal localization of γ-tubulin during metaphase. Parental HeLa cells (wild type, WT) and Nur77-depleted (Nur77^–/–^) HeLa cells transfected with control empty vector or Flag-Nur77 or Flag-Nur77 mutants were synchronized at mitosis by TT treatment and analyzed by immunostaining γ-tubulin (green), Cep192 (red), and DAPI (blue). Relative fluorescence intensity of γ-tubulin at the centrosome was calculated (**B**) (*n* = 50 cells across four experiments; ns *p* = 0.0767, ****p < 0.0001; two-tailed unpaired t-test). Scale bar, 10 μm. **C**, **D** Effect of Nur77 on the MT nucleation activity of centrosome by MT regrowth assay. HeLa cells transfected with control or Nur77 siRNA were synchronized at mitosis by TT block, cold-treated, then reheated for the indicated time at 37 °C, followed by staining α-tubulin (red) and DAPI (blue). Relative MT nucleation activity at the centrosome during metaphase was scored (**D**) (*n* = 50 cells across three experiments; *p = 0.0193, *p = 0.0196, **p = 0.0049; two-tailed unpaired t-test). Scale bar, 10 μm. **E**, **F** Effect of Nur77 on the mitotic spindle. Wild-type HeLa cells and Nur77-depleted HeLa cells transfected with empty vector or Flag-Nur77 or Flag-Nur77 mutants were synchronized at mitosis by TT treatment, then stained with Cep192 (red), α-tubulin (green) and DAPI (blue). Percentage of aberrant spindles was calculated, respectively **F** (*n* = 200 cells across three experiments; ***p = 0.0005, **p = 0.0010, ns *p* = 0.6184, **p = 0.0006; two-tailed unpaired t-test). Scale bar, 10 μm. **G**, **H** Nur77 deficiency induces chromosome misalignment during metaphase and lagging chromosome during anaphase. Wild-type HeLa cells and Nur77-depleted HeLa cells transfected with empty vector or Flag-Nur77 or Flag-Nur77 mutants were synchronized at mitosis by TT treatment, then stained with DAPI (blue). Percentage of chromosome misalignment during metaphase and lagging chromosome during anaphase was calculated **H** (*n* = 200 cells across five experiments; ****p < 0.0001, ns *p* = 0.4999, **p = 0.0011, **p = 0.0015, ns *p* = 0.5195, ***p = 0.0006; two-tailed unpaired t-test). Scale bar, 10 μm. Effect of Nur77 and mutants on the mitotic progression. Wild-type (WT) and Nur77 depleted (Nur77^–/–^) HeLa cells were transfected with or without Flag-Nur77 and mutants, and synchronized by nocodazole treatment for 12 h. Cells were then released for the indicated time, and analyzed by WB (**I**) and FACS (**J**). All quantitative data in this figure are presented as mean values ± SEM. Data represent at least three independent experiments.

To determine whether Nur77 regulates centrosome maturation, we examined mitotic HeLa cells following Nur77 depletion via CRISPR-Cas9 or siRNA. Nur77 depletion disrupted centrosome integrity and significantly reduced γ-tubulin intensity at the centrosome, a hallmark of defective centrosome maturation (Fig. 4A, B and Supplementary Fig. 4A–D). This phenotype closely resembled that of Cep192 knockdown (Supplementary Fig. 4E), suggesting that Nur77 operates within the Cep192-dependent centrosomal maturation pathway. Importantly, this defect was rescued by reintroduction of wild-type Nur77 or the phosphomimetic T143E mutant, but not the non-phosphorylatable T143A mutant (Fig. 4A, B), demonstrating that Thr143 phosphorylation is essential for Nur77’s mitotic function in tumor cells. Although Nur77 depletion affects the localization of other components involved in γ-tubulin recruitment at the centrosome [53–55], it did not interact with them in Co-IP assays (Supplementary Fig. 4F–4J). Thus, Nur77 most likely promotes γ-tubulin recruitment primarily through direct regulation of the Cep192–PLK1 axis. Since centrosome maturation is required for microtubule nucleation and bipolar spindle formation [29, 56], we next assessed whether Nur77 influences microtubule dynamics. Microtubule regrowth assays revealed impaired nucleation and spindle assembly in Nur77-deficient metaphase tumor cells (Fig. 4C, D and Supplementary Fig. 4K), phenocopying the effect of Cep192 depletion [51]. Furthermore, Nur77 depletion resulted in abnormal spindle structures, including multipolar or malformed spindles (Fig. 4E, F and Supplementary Fig. 4L, M). This phenotype was reversed by reintroducing Nur77 or Nur77-T143E, but not Nur77-T143A, reinforcing the functional importance of Thr143 phosphorylation. Disrupted spindle assembly typically leads to chromosome misalignment, lagging chromosomes, and subsequent micronucleus formation, ultimately compromising genome integrity [29, 56, 57]. Indeed, Nur77 depletion led to prominent chromosome alignment defects and increased lagging chromosomes, which were again rescued by Nur77 or Nur77-T143E, but not Nur77-T143A (Fig. 4G, H). Strikingly, these phenotypes extended to primary mouse breast tumor cells, where Nur77 depletion disrupted Cep192 organization, impaired spindle assembly, and increased chromosomal defects and micronucleus formation (Supplementary Fig. 4N–Q). Altogether, these results establish a critical role for Thr143-phosphorylated Nur77 in centrosome maturation and mitotic fidelity.

### Cdk1-mediated phosphorylation at Thr143 is required for proper mitotic progression in tumor cells

To investigate the biological consequences of Nur77 loss on mitotic progression, we synchronized various cancer cell lines depleted of Nur77 using nocodazole arrest followed by release and monitored mitotic progression (Supplementary Fig. 4R–W). Compared to controls, Nur77-deficient cells exhibited delayed mitotic progression, as evidenced by prolonged cyclin B1 and phospho-H3 (pS10-H3) expression (Supplementary Fig. 4R, T, V) and delayed G1 re-entry (Supplementary Fig. 4S, U, W). These effects mirrored those observed following PLK1 knockdown (Supplementary Fig. 4X). Consistent with impaired mitotic progression, colony formation assays showed reduced proliferation in Nur77-depleted cells (Supplementary Fig. 4Y). Reconstitution of Nur77 or Nur77-T143E, but not Nur77-T143A, restored mitotic progression and cell proliferation (Figs. 4I, J and Supplementary Fig. 4Z), demonstrating that Cdk1-mediated phosphorylation of Nur77 at Thr143 is necessary for regulating the mitotic progression of tumor cells.

### Phosphorylated Nur77 is elevated in tumors and drives tumor growth in vivo

**Fig. 5:**
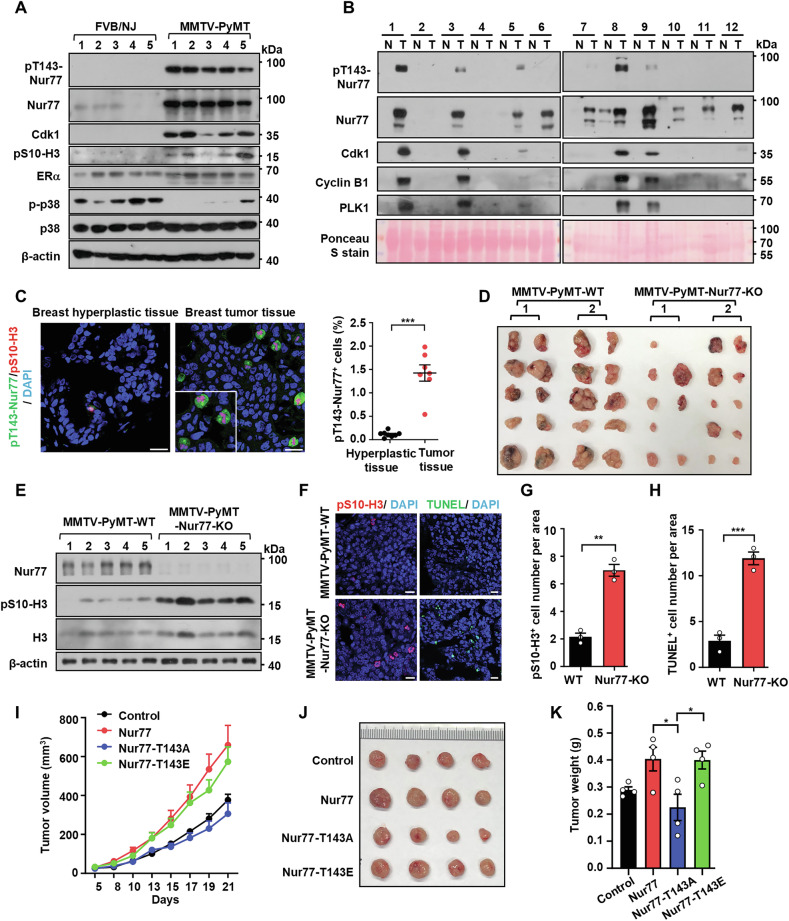
pT143-Nur77 is elevated in tumor and promotes tumor growth. **A** Normal mammary glands from FVB/NJ mice and breast tumor tissues from MMTV-PyMT mice were analyzed by WB. **B** WB analysis of pT143-Nur77 expression in human breast cancer tissues (T) and their corresponding tumor-adjacent normal tissues (N). **C** IF analysis of pT143-Nur77 (green) and pS10-H3 (red), and DAPI (blue) in human breast hyperplastic tissues and breast tumor tissues. Left: images of breast hyperplastic tissues (*n* = 8) and breast tumor tissues (*n* = 7). Right: percentage of pT143-Nur77^+^ cells (twenty random fields per tissue were analyzed; ***p = 0.003; two-tailed unpaired t-test). Scale bar, 20 μm. **D** Nur77 knockout in MMTV-PyMT mice suppresses the growth of breast tumor. Representative images of tumors excised from wild type and Nur77 knockout MMTV-PyMT mice were shown. **E** WB analysis of breast tumor tissues from wild type and Nur77 knockout MMTV-PyMT mice. **F**–**H** Nur77 deficiency induces mitotic arrest and apoptosis in breast tumor tissues. Breast tumor tissues from wild-type and Nur77 knockout MMTV-PyMT mice were stained with pS10-H3 (red) and DAPI (blue), or analyzed by TUNEL staining. Percentage of cells with positive pS10-H3 **G** and TUNEL staining **H** was calculated respectively (ten random fields per sample were analyzed; **p = 0.0016, ***p = 0.0007; two-tailed unpaired t-test). Scale bar, 20 μm. Effect of Thr143 phosphorylation of Nur77 on the growth of breast tumor in vivo. The tumor volume was monitored and recorded once in two or three days since mice were transplanted with MDA-MB-231 cells stably expressing Nur77 or mutants for 5 days (**I**). Tumors excised from mice were shown (**J**) and weighed (**K**) (*n* = 4 per group; *p = 0.0274, *p = 0.0268; two-tailed unpaired t-test). All quantitative data in this figure are presented as mean values ± SEM. Data of **A,** **E,** **F,** **G**, **H** are representative of three independent experiments.

Given the critical role of phosphorylated Nur77 (p-Nur77) in mitotic fidelity of tumor cells, we next examined its expression in tumor tissues. Analysis of spontaneous mammary tumors from MMTV-PyMT mice revealed markedly elevated levels of both total Nur77 and p-Nur77 in tumors compared to normal mammary glands (Fig. 5A). In contrast, the expression of estrogen receptor alpha (ERα), another nuclear receptor, remained unchanged, highlighting the tumor-selective nature of Nur77 upregulation. Elevated p-Nur77 levels correlated with increased expression of Cdk1, consistent with our mechanistic data implicating Cdk1 as the upstream kinase responsible for Thr143 phosphorylation. The activation of p38 MAPK (a known kinase for Nur77 Thr143 phosphorylation) [44] did not align with p-Nur77 expression levels, reinforcing the specificity of Cdk1 in tumor-associated Nur77 phosphorylation.

To extend these findings to clinical human cancer, we analyzed paired tumor and adjacent normal tissues from breast cancer patients. Total Nur77 was upregulated in 25 of 36 tumor samples, and among these, a distinct subset exhibited elevated p-Nur77, which correlated with the co-expression of Cdk1 and cyclin B1 (Fig. 5B, Supplementary Fig. 5A, and Table S1), supporting the clinical relevance of Cdk1/cyclin B1-mediated Thr143 phosphorylation of Nur77. Despite the low mitotic index characteristic of solid tumors [58, 59], p-Nur77 was enriched in breast tumor tissues relative to hyperplastic tissues (Fig. 5C), supporting its role in mitosis and tumor growth.

To evaluate the in vivo function of Nur77 in tumorigenesis, we crossed MMTV-PyMT mice with Nur77-knockout mice, generating MMTV-PyMT-Nur77-KO animals. Loss of Nur77 significantly suppressed tumor growth (Fig. 5D and Supplementary Fig. 5B, C). Tumors from Nur77-deficient mice displayed elevated levels of phospho-Histone H3 (Ser10) and mitotic arrest, accompanied by increased chromosome misalignment, micronuclei formation, and multicentrosomes (Fig. 5E–G and Supplementary Fig. 5D–F). These mitotic abnormalities related to aberrant centrosome function phenocopied those observed in Nur77-depleted tumor cells (Supplementary Fig. 4O–Q). Unlike in tumors, Nur77 depletion did not cause mitotic arrest in normal liver tissues (Supplementary Fig. 5G), which underscores the tumor-specific reliance on Nur77 for mitotic progression. Mechanistically, this mitotic arrest was coupled with reduced proliferation and increased apoptotic cell death, as demonstrated by decreased Ki-67 staining, elevated TUNEL staining, and enhanced cleaved caspase-3 immunostaining in Nur77-null tumors (Fig. 5F, H and Supplementary Fig. 5H, I). These results support a model in which tumor cells depend on Nur77-mediated centrosome function to complete mitosis and avoid mitotic catastrophe [58, 60–64].

To further establish the requirement for Thr143 phosphorylation in tumor growth, we performed xenograft studies using MDA-MB-231 cells stably expressing wild-type Nur77, Nur77-T143E, or Nur77-T143A. While wild-type and T143E-expressing cells formed rapidly growing tumors, T143A-expressing cells exhibited significantly impaired tumor growth (Fig. 5I–K and Supplementary Fig. 5J), confirming that Cdk1-mediated phosphorylation of Nur77 at Thr143 is not only essential for mitotic function but also for in vivo tumor growth.

Together, these results establish p-Nur77 as a tumor-enriched mitotic effector that supports centrosome function, mitotic progression, and tumor growth. This defines a cancer-selective mitotic mechanism that may be therapeutically targeted to exploit tumor dependency on p-Nur77.

### NMA39 targets Nur77 to induce mitotic arrest and death in tumor cells

**Fig. 6:**
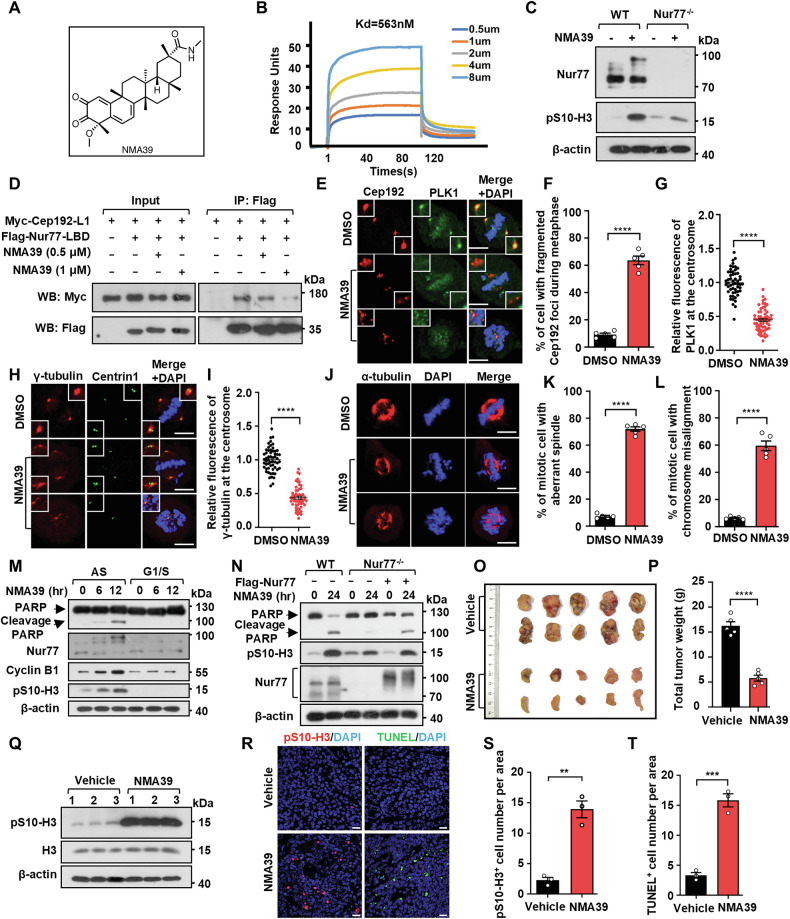
Celastrol derivative NMA39 exerts anti-mitotic activity by targeting Nur77. **A** NMA39 structure. **B** Binding of NMA39 to purified Nur77-LBD was analyzed by surface plasmon resonance (SPR) assay. **C** HeLa cells (WT) and Nur77 depleted HeLa cells (Nur77^–/–^) were treated with NMA39 (0.5 μM) for 12 h and analyzed by WB. **D** HeLa cells transfected with Myc-Cep192-L1 and Flag-Nur77-LBD were treated with or without NMA39 (0.5 μM or 1 μM) for 1 h and analyzed by CoIP. **E**–**G** HeLa cells released from TT block for 10 h were treated with 1 μM NMA39 for 0.5 h fixed, and stained. Cep192 (red), PLK1 (green), and DAPI (blue). Percentage of mitotic cells with fragmented Cep192 foci (**F**) and relative fluorescence intensity of PLK1 at the centrosome during prometaphase or metaphase (**G**) were calculated respectively (left graph: *n* = 200 cells across five experiments; right graph, *n* = 54, and 59 cells, respectively; ****p < 0.0001; two-tailed unpaired *t*-test). Scale bar,10 μm. **H**, **I** Mitotic HeLa cells released from TT block were treated with 1 μM NMA39 for 0.5 h fixed, and stained. γ-tubulin (red), centrin1 (green) and DAPI (blue). Relative fluorescence intensity of γ-tubulin at the centrosome during prometaphase or metaphase was calculated (**I**) (*n* = 57, and 55 cells, respectively; ****p < 0.0001; two-tailed unpaired *t*-test). Scale bar,10 μm. **J**–**L** HeLa cells synchronized at mitosis by TT treatment were treated with NMA39 (1 μM) for 0.5 h fixed, then stained. α-tubulin (red), and DAPI (blue). Percentage of disordered spindle during mitosis (**K**) and percentage of chromosome misalignment during metaphase (**L**) were calculated, respectively (left graph: *n* = 200 cells across five experiments; right graph, *n* = 200 cells across five experiments; ****p < 0.0001; two-tailed unpaired *t*-test). Scale bar,10 μm. **M** Asynchronous HeLa cells (AS) or HeLa cells synchronized at the G1/S transition by TT block were incubated with 0.5 μM NMA39 for the indicated time and analyzed by WB. **N** The anti-mitotic effect of NMA39 is Nur77-dependent. HeLa cells (WT) and Nur77- depleted HeLa cells (Nur77^–/–^) transfected with empty vector or Flag-Nur77 were treated with NMA39 (0.5 μM) for 24 h and analyzed by WB. Female wild-type MMTV-PyMT mice (12 weeks old) were randomly divided into two groups (*n* = 5) and treated daily with NMA39 (10 mg/kg) for 14 days. Representative images of tumors from MMTV-PyMT mice administered with or without NMA39 were shown (**O**) and tumors were weighed (**P**) (*n* = 5 per group; ****p < 0.0001; two-tailed unpaired *t*-test). Tumor specimens from MMTV-PyMT mice treated with or without NMA39 were analyzed by WB (**Q**), stained with pS10-H3 (red) and DAPI (blue) (**R**), or analyzed by TUNEL staining (**R**). Percentage of cells with positive pS10-H3 (**S**) and TUNEL staining (**T**) was calculated respectively (ten random fields per sample were analyzed; **p=0.0013, ***p = 0.0004; two-tailed unpaired t-test). Scale bar, 20 μm. All quantitative data in this figure are presented as mean values ± SEM. Data of **B**–**M**, **Q**, **R** are representative of three independent experiments.

While Nur77 lacks a canonical ligand-binding pocket, accumulating evidence suggests it can be modulated by small molecules that engage alternative structural regions [6, 18, 19]. We previously reported that celastrol, a triterpene derived from the traditional Chinese medicine *Tripterygium wilfordii*, binds Nur77 and modulates its activity [15, 65]. In addition, we developed a series of celastrol analogs with improved selectivity for Nur77 [66]. To explore whether Nur77 could be pharmacologically targeted to disrupt its mitotic function, we screened a panel of celastrol derivatives and Nur77 modulators for their ability to induce mitotic arrest, measured by elevated pS10-H3 levels and G2/M arrest. Our study identified NMA39, a celastrol analog, as a potent inducer of mitotic arrest that outperformed its parent compound (Fig. 6A and Supplementary Fig. 6A, B). Surface plasmon resonance (SPR) confirmed direct binding of NMA39 to Nur77 with submicromolar affinity (Kd = 563 nM; Fig. 6B). Functionally, the mitotic effects of NMA39 were substantially attenuated in Nur77-deficient cells (Fig. 6C and Supplementary Fig. 6C), demonstrating that Nur77 is its primary target.

Mechanistically, NMA39 disrupted the interaction between Nur77 and the centrosomal scaffold Cep192. Co-IP showed that NMA39 impaired the binding of Nur77-LBD to the PLK1-docking domain of Cep192 (L1) in a dose-dependent manner (Fig. 6D), and immunofluorescence analysis confirmed that NMA39 reduced both the centrosomal colocalization and cytoplasmic condensates of these proteins (Supplementary Fig. 6D, E). NMA39 also blocked Nur77–PLK1 interaction during mitosis (Supplementary Fig. 6F), mimicking the effect of Cdk1 inhibition. As a result, NMA39 treatment led to disrupted centrosomal architecture, reduced PLK1 recruitment (Fig. 6E–G), and impaired centrosome maturation, as evidenced by reduced γ-tubulin loading, microtubule nucleation, and defective spindle assembly (Fig. 6H–K and Supplementary Fig. 6G). These mitotic defects culminated in severe chromosomal instability, including misaligned chromosomes (Fig. 6L), similar to the effects of Nur77 depletion. Consistent with its selectivity, NMA39 also exerted these effects in primary mouse breast tumor cells (Supplementary Fig. 6H–J).

In addition to mitotic disruption, NMA39 induced apoptosis across multiple tumor cell lines (Supplementary Fig. 6K, L), including primary mouse breast tumor cells (Supplementary Fig. 6M, N). Notably, NMA39 failed to induce apoptosis or mitotic arrest in non-malignant MCF-10A cells, while PLK1 inhibitor BI2536 remained effective (Supplementary Fig. 6L, O), indicating a tumor-selective mechanism of action.

To confirm the link between mitotic arrest and cell death, we treated synchronized HeLa cells with NMA39 after release from G1/S block. Cells entered S phase normally but accumulated in mitosis and underwent apoptosis thereafter (Supplementary Fig. 6P). This effect was abolished in G1/S-arrested cells (Fig. 6M and Supplementary Fig. 6Q) but not in asynchronous cells, suggesting that NMA39 induces cell death in a mitosis-dependent manner. Immunofluorescence analysis further confirmed that NMA39-treated cells undergo mitotic cell death, as evidenced by a subset of cells exhibiting both mitotic arrest/multipolar spindles and apoptotic signals (Supplementary Fig. 6R–U). Rescue experiments in Nur77-deficient cells further demonstrated that NMA39-induced mitotic cell death requires Nur77 (Fig. 6N). Finally, we tested the therapeutic potential of targeting p-Nur77 in vivo using NMA39. In MMTV-PyMT mice, systemic administration of NMA39 significantly inhibited tumor growth (Fig. 6O, P), accompanied by increased mitotic arrest and elevated tumor apoptosis (Fig. 6Q–T and Supplementary Fig. 6V).

Taken together, these results demonstrate that p-Nur77 can be pharmacologically targeted. The Nur77 modulator NMA39 provides evidence for this, as it selectively targets the Nur77–Cep192–PLK1 axis to induce mitotic catastrophe and death in tumor cells. This study thus identifies a tumor-selective mitotic mechanism driven by Cdk1-phosphorylated Nur77 and introduces Nur77 modulation as a promising strategy for anti-mitotic cancer therapy.

## Discussion

**Fig. 7:**
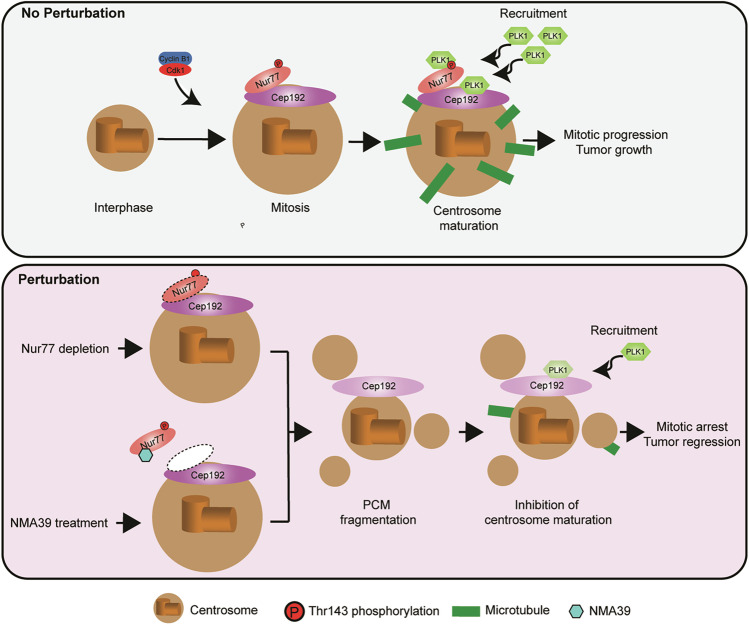
Model for Nur77–Cep192–PLK1 signaling axis. At the onset of mitosis, Cdk1-mediated phosphorylation of Nur77 accumulates at the centrosome where it interacts with Cep192. This interaction facilitates the centrosomal recruitment of PLK1 and promotes centrosome maturation, thereby driving mitotic progression and tumor growth. Depletion of Nur77 or treatment with NMA39, a novel small-molecule Nur77 modulator that disrupts the Nur77-Cep192 interaction, compromises centrosome integrity and inhibits centrosome maturation, resulting in mitotic arrest and tumor regression.

As a multifunctional orphan nuclear receptor, Nur77 regulates diverse cellular processes not only through its well-characterized nuclear transcriptional roles but also through its extranuclear actions at the mitochondria and the endoplasmic reticulum, highlighting its functional complexity [21]. This study uncovers a new mitotic function of Nur77 in tumor cells through its centrosome targeting during mitosis. We show that Nur77 is phosphorylated at threonine 143 by the Cdk1/cyclin B1 complex, leading to its centrosomal accumulation during mitosis. There, phosphorylated Nur77 binds to the scaffold protein Cep192 to maintain centrosome integrity and facilitates PLK1 recruitment, thereby promoting centrosome maturation and mitotic progression (Fig. 7). Importantly, this function occurs despite global transcriptional silencing during mitosis when transcription factors are dissociated from condensed chromosomes [67–70], demonstrating a pivotal role of Nur77 in supporting the survival and growth of dividing tumor cells.

Mechanistically, we demonstrated that Nur77 forms a complex with Cep192 and PLK1 at the centrosome through a unique mechanism. Nur77 interacts with Cep192 via its C-terminal LBD and with PLK1 via its N-terminal A/B domain, which requires phosphorylation at Thr143 for this interaction. As an orphan nuclear receptor, the functional plasticity of Nur77 is primarily governed by its expression level and post-translational modifications. Nur77 contains multiple phosphorylation sites within its intrinsically disordered N-terminal A/B domain, which is known to facilitate dynamic protein–protein interactions [16]. We previously showed that this domain mediates liquid–liquid phase separation (LLPS) with the autophagy receptor p62 at mitochondria to regulate mitophagy [15, 16]. Notably, LLPS has been implicated in the assembly and organization of the pericentriolar PCM at centrosomes [71, 72]. Exogenous Cep192 could form droplet-like condensates in cells [51], and purified *C. elegans* SPD-2 protein (orthologue of mammalian Cep192) could be recruited into SPD-5 (orthologue of mammalian Cep215) condensates in vitro [73], suggesting that Cep192 may be capable of phase separation. Our data show that exogenously expressed Cep192 forms condensates in cells, and that Nur77, particularly its phospho-mimetic T143E mutant, enhances these condensates and facilitates PLK1 recruitment (Supplementary Fig. 3W–Y). The substantial mobility shift of Nur77 on SDS-PAGE during mitosis suggests the existence of additional modifications, which may enhance its multivalency and interaction versatility. These findings suggest a model wherein Nur77 modifications promote centrosomal integrity and function by driving LLPS of Cep192—a mechanism that warrants further investigation.

Mitotic dysregulation is a well-established hallmark of cancer and a clinically validated target, as evidenced by microtubule-targeting agents like taxanes [58, 59, 74]. Despite their efficacy, these agents suffer from limited tumor specificity [58, 59, 74]. Thus, the identification of tumor-specific vulnerabilities during mitosis is critical for the next generation of safer and more effective anti-mitotic therapies [22, 23, 58, 59, 74, 75]. Our work identifies a tumor-selective mitotic mechanism driven by Cdk1-mediated phosphorylation of Nur77 at Thr143. This modification licenses Nur77 to scaffold PLK1 at the centrosome, thereby regulating mitotic progression in tumor cells. Nur77, Cdk1, and PLK1 are frequently overexpressed in tumors [21, 58, 74, 76–78], and we found that p-Nur77 is significantly elevated in both murine and human tumors, correlating with mitotic activity and PLK1 expression (Fig. 5A, B and Supplementary Fig. 5A; Table S1). Notably, as an immediate-early response gene, Nur77’s expression is minimal or absent in most normal adult tissues in the absence of stimuli, and Nur77 knockout mice are viable with no overt mitotic phenotype [21, 79]. Our data demonstrate that Nur77 depletion impairs mitosis and reduces tumor growth without affecting normal hepatocytes. This suggests that its centrosomal role is tumor-restricted and dispensable in non-transformed cells. Thus, p-Nur77 emerges as a tumor-selective mitotic vulnerability, offering a promising therapeutic target. We further identify NMA39, a celastrol-derived Nur77 modulator, as a proof-of-concept compound for targeting this tumor-specific mitotic axis. NMA39 binds Nur77 with high affinity, disrupts its interactions with Cep192 and PLK1, and impairs centrosome maturation, leading to mitotic arrest and apoptosis in tumor cells (Fig. 7). Remarkably, NMA39 spares non-malignant cells, unlike traditional PLK1 inhibitors (Supplementary Fig. 6O), and exhibits potent antitumor activity in vivo (Fig. 6O–T and Supplementary Fig. 6V). These findings position NMA39 as a lead compound for Nur77-based anti-mitotic therapy with improved selectivity and reduced toxicity.

In summary, this study reveals a tumor-specific mitotic mechanism whereby Cdk1-phosphorylated Nur77 coordinates Cep192–PLK1 signaling at the centrosome to promote centrosome maturation and mitotic progression. This non-genomic, transcription-independent function of Nur77 occurs during mitosis and depends on its posttranslational modification. Our discovery of NMA39 as a Nur77-targeting mitotic disruptor highlights a new therapeutic strategy for selectively eliminating tumor cells by exploiting their dependency on Nur77 modification. Together, these findings define Thr143-phosphorylated Nur77 as a new tumor-selective mitotic scaffold and therapeutic vulnerability in cancer.

## Method

### Experimental model and subject details

#### Cell lines

Human cervical carcinoma cell line HeLa was maintained in Minimum Essential Medium (MEM)/EBSS (Hyclone) containing 10% fetal bovine serum (FBS). Human lung adenocarcinoma cell line A549 was maintained in Kaighn’s Modification of Ham’s F-12 Medium (Gibco) supplemented with 10% FBS. Human colorectal cancer cell line HCT116 was cultured in RPMI-1640 (Hyclone) containing 10% FBS. Human hepatocellular carcinoma cell line HepG2, human breast cancer cell lines MCF7 and MDA-MB-231 were cultured in Dulbecco’s Modified Eagle’s Medium (DMEM) (Hyclone) supplemented with 10% FBS. Human breast epithelial cell line MCF-10A was maintained in DMEM/F-12 Medium (1:1) (Gibco), supplemented with 10 μg/mL insulin, 20 ng/mL EGF, 100 ng/mL cholera toxin, 0.5 μg/mL hydrocortisone and 5% horse serum. The culture media above were supplemented with 100 U/mL penicillin-streptomycin (Hyclone). The cells used in this study were purchased from American Type Culture Collection (Manassas) and routinely checked for mycoplasma contamination and cultured at 37 °C in a humidified 5% CO_2_ atmosphere. For cell culture experiments, dishes were randomly divided into control and treatment groups.

#### Mice

Nude mice (BALB/c, female, 4-6 weeks old) were purchased from Beijing Vital River Laboratory Animal Technology (China). Wild-type and Nur77^–/–^mice (C57BL/6 genetic background) and MMTV-PyMT transgenic mice (FVB/N background) were purchased from The Jackson Laboratory (Bar Harbor, Maine, USA). These mice were maintained in an animal room with 12 h light/12 h dark cycles at the Animal Facility in Xiamen University. The protocols for animal studies were approved by the Animal Care and Use Committee of Xiamen University, and all mice were handled in accordance with the “Guide for the Care and Use of Laboratory Animals” and the “Principles for the Utilization and Care of Vertebrate Animals.”

#### Plasmids

Plasmids pcmv-myc-Nur77, pGFP-Nur77, p3×Flag-cmv-10-PLK1, pcmv-myc-RXRα, pcmv-HA-RXRα, pcmv-myc-cyclin B1, p3×Flag-cmv-10-Cdk1, p3×Flag-cmv-10-Aurora A, pET-21a-mCherry-Nur77 were described previously [15, 16, 37]. p3×Flag-cmv-10-Nur77, p3×Flag-cmv-10-Nur77-AB, p3×Flag-cmv-10-Nur77-LBD, p3×Flag-cmv-10-Nur77-ΔAB, p3×Flag-cmv-10-Nur77-ΔLBD, p3×Flag-cmv-10-Nur77-T143A, p3×Flag-cmv-10-Nur77-T143E, pcmv-Nur77, pcmv-Nur77-T143A, pmCherry-Nur77, pcmv-myc-PLK1, pcmv-myc-PLK1-KD, pcmv-myc-PLK1-PBD, pcmv-HA-PLK1-K82R, pmCherry-PLK1, p3×Flag-cmv-10-Cep192-L1, p3×Flag-cmv-10-Cep192-L2, p3×Flag-cmv-10-Cep192-L3, pcmv-myc-Cep192-L1, pcmv-myc-Cep192-L1-T44A/S995A and pEGFP-α-tubulin, pmCherry-H2B were constructed using polymerase chain reaction (PCR) or the QuikChange mutagenesis kit.

#### Antibodies and reagents

Anti-Nur77 (CST, 3960S), Anti-PLK1 (208G4) (CST, 4513S), Anti-Phospho-PLK1(Thr210) (CST, 5472S), Anti-β-actin (CST, 4970S), Anti-α-tubulin (CST, 3873S), Anti-Cyclin B1 (CST, 4138S), Anti-Cyclin B1 (CST, 12231S), Anti-Cyclin E2 (CST, 4132), Anti-p-Histone H3 (CST, 3377S), Anti-Cleaved-caspase3 (CST, 9661S), Anti-PARP (CST, 9542), Anti-p-JNK (CST, 4668S), Anti-JNK (CST, 3708S), Anti-p38 (CST, 9212S), Anti-Phospho-p38 (CST, 9215), Anti-Phospho-GSK3β (Ser9) (CST, 5558), Anti-Phospho-AKT (Ser473) (CST, 4060S), Anti-Nur77 (M-210) (Santa Cruz, sc-5569), Anti-Myc (Santa Cruz, sc-40), Anti-RXRα (D-20) (Santa Cruz, sc-553), Anti-cdc2 (Santa Cruz, sc-54), Anti-AKT1/2/3 (Santa Cruz, sc-8312), Anti-HA (Y-11) (Santa Cruz, sc-805), Anti-ERα (D-12) (Santa Cruz, sc-8005), Anti-NEDD1 (Santa Cruz, sc-398733), Anti-FLAG (Sigma, F1804), Anti-γ-tubulin (Sigma, T5326), Anti-Cep192 (BETHYL, A302-324A), Anti-Nur77 (Proteintech, 12235-1-AP), Anti-centrin1 (Proteintech, 12794-1-AP), Anti- Histone H3 (Proteintech, 17168-1-AP), Anti-GAPDH (Proteintech, 60004-1-Ig), Anti-Cep215 (Proteintech, 31702-1-AP), Anti-pericentrin (Proteintech, 22271-1-AP), Anti-Aurora A (Proteintech, 10297-1-AP), Anti-PAR/pADPr (R&D systems, 4335-MC-100), Anti-Nur77 (Affinity, DF7850), Anti-PLK1 (Invitrogen, 37-7000), Thymidine (Sigma, T1895), Nocodazole (Sigma, M1404), Ginkgolic acid (Sigma, 49962), LiCl (Sigma, L9650), SP600125 (Sigma, S5567), FastAP Thermosensitive Alkaline Phosphatase (AP) (Fermentas, EF0654), PNGaseF (Beyotime, P2318S), BI2536 (Santa Cruz, sc-364431), PYR-41(Selleck Chemicals, S7129), RO-3306 (Selleck Chemicals, S7747), Palbociclib HCL (Selleck Chemicals, S1116), Flavopiridol (Selleck Chemicals, S1230), SB203580 (Selleck Chemicals, S1076), AKTi-1/2 (Selleck Chemicals, S7776), Niraparib (MedChemExpress, HY-10619), Nur77 siRNAs (Sigma, SASI_Hs02_00333289, SASI_Hs02_00333290, and SASI_Hs02_00333291), PLK1 siRNAs (Sigma, SASI_Hs01_00194343), Cep192 siRNA (5’-GGAAGACAUUUUCAUCUCU-3’)[80] and PLK1 siRNA (5’- CCUUGAUGAAGAAGAUCAC-3’) [81] were synthesized by GenePharma (Shanghai, China). Control siRNAs from Sigma and GenePharma were used.

### Method details

#### Transfection

Transfection was performed using Lipofectamine 2000 (Invitrogen, USA) or PEI reagent (Polysciences, USA) according to the manufacturer’s instructions. To establish stable clones expressing Nur77, HeLa cells were transfected with control vector p3xFlag-cmv-10 or p3xFlag-cmv-10-Nur77 plasmids by Lipofectamine 2000. After 48 h, the medium was replaced with G418-containing (1 mg/mL) medium. Individual colonies were picked up after a 12-day selection, and a Western blot assay was conducted to examine the expression of Nur77. To establish MDA-MB-231 cells stably expressing Nur77, Nur77-T143A, or Nur77-T143E, cells were transfected with the pLenti-GFP vector, pLenti-GFP-Nur77, pLenti-GFP-Nur77-T143A, or pLenti-GFP-Nur77-T143E plasmid by Lipofectamine 2000, respectively. Puromycin (0.5 μg/mL) was added to culture media after 48-h transfection. After a 10-day selection, positive cells were sorted by flow cytometry (BD FACS Aria™Ⅲ) based on GFP signals. Expression of Nur77 or its mutants in transfected cells was further determined by Western blotting.

#### Cell synchronization

Cells were synchronized with a double thymidine block at the G1-S boundary as reported before [82]. Briefly, cells were treated with 2 mM thymidine for 16 h and released into the normal culture medium for 8 h. Subsequently, cells were subjected to a secondary thymidine block for 16 h again, released into normal medium and harvested at the indicated time. Alternatively, a thymidine-nocodazole arrest was used for synchronization at prometaphase that cells were subjected to 18-h thymidine arrest and a 5-h release, followed by a 5-h 100 ng/mL nocodazole arrest.

#### SILAC-based immunoprecipitation quantitative proteomics

SILAC-based immunoprecipitation quantitative proteomics was performed as described before [37]. Briefly, HeLa cells stably transfected with control Flag plasmid were grown in “light” medium (SILAC MEM without lysine and arginine, Thermo), supplemented with 10% dialyzed fetal calf serum (Thermo), 100 U/mL penicillin (Hyclone), 100 U/mL streptomycin (Hyclone), 100 μg/mL L-arginine and 200 μg/mL L-lysine (both from Sigma). For stable isotope labeling with amino acids in cell culture (SILAC), HeLa cells stably expressing Flag-Nur77 were cultured in “heavy” medium (SILAC MEM without lysine and arginine, Thermo), supplemented with 10% dialyzed fetal calf serum (Thermo), 100 U/mL penicillin (Hyclone), 100 U/mL streptomycin (Hyclone), 200 μg/mL L-proline (Sigma), 100 μg/mL L-arginine-HCl (13C6;15N4) and 200 μg/mL L-lysine-2HCl (13C6;15N2) (both from CIL, USA). Above two groups of cells were cultured in the corresponding medium by changing medium every 2 days for at least seven cell divisions.

To perform immunoprecipitation coupled with liquid chromatography tandem mass spectrometry (LC-MS/MS), two groups of cells were grown in 10-cm culture dishes (*n* = 10 per group), then harvested and lysed in IP buffer (50 mM Tris-HCl pH 7.4, 150 mM NaCl, 0.5% NP-40, 10 mM EDTA) supplemented with protease and phosphatase inhibitors (Roche). The protein concentration of cell lysates from both groups was determined by BCA assay (Thermo), and an equal amount of cell lysates was incubated with anti-Flag M2 beads (Sigma) at 4 °C for 2 h. Two samples were washed by IP buffer for three times and mixed at a 1:1 ratio then separated by denatured 4–20% gradient SDS-PAGE. After silver staining, the gel lane was cut horizontally into 25 gel pieces followed by in-gel destained, reduced, alkylated and digested with trypsin. Peptides in gel were extracted and concentrated by Speed-Vac centrifuge, desalted, filtered through a C18 ziptip, and redissolved with 0.1% formic acid in ultrapure water. At the end, peptides were analyzed by HPLC with a Q-Exactive tandem mass spectrometer (Thermo) as described before [37].

#### Co-immunoprecipitation

Co-immunoprecipitation (CoIP) assays were conducted as described before [15, 37]. Briefly, cells were harvested in lysis buffer (10 mM Tris [pH 7.4], 150 mM NaCl, 0.5% NP40, 5 mM EDTA) containing protease and phosphatase inhibitors, gently rotated for 20 min at 4 °C. Cell lysates were centrifuged at 12,000 rpm for 15 min at 4 °C. 40 μl supernatants were mixed with 10 μl of 5× sodium dodecyl sulfate (SDS) sample loading buffer and boiled for 5 min at 100 °C as total input. The remaining supernatants were incubated with 1 μg antibody at 4 °C for 2 h or overnight. Immunocomplexes were precipitated with 30 μl protein G-Sepharose for 1 h. After washing three times with lysis buffer, the beads were mixed with 50 μl 2× loading buffer, boiled, and assessed by Western blotting.

#### Western blotting

Western blot (WB) was performed as described previously. In brief, cells were incubated and adequately lysed on ice with lysis buffer (25 mM Tris-HCl pH 7.4, 150 mM NaCl, 1% NP40, 5 mM EDTA) containing protease and phosphatase inhibitor cocktail. After centrifugation at 12,000 rpm for 15 min at 4°C, the supernatant was collected and quantified using Pierce BCA Protein Assay Kit with BSA as standard. Cell lysates mixed with SDS sample loading buffer were boiled, separated on 8–12% SDS–polyacrylamide gel electrophoresis (SDS-PAGE) and transferred to PVDF membranes. The membranes were blocked in 5% non-fat milk in Tris buffered saline and Tween 20 (TBST; 10 mM Tris–HCl [pH 8.0], 150 mM NaCl, 0.05% Tween 20) for 1 h at RT and then incubated with appropriate primary antibodies at 4 °C overnight. After washing with TBST, the membranes were incubated with horseradish peroxidase-labeled secondary antibodies (1:5000 dilution) for 1 h at RT. After washing with TBST, immunoreactive products were visualized using enhanced chemiluminescence reagents.

#### Generation of anti-pT143-Nur77 antibody

Rabbit polyclonal phosphospecific antibody (anti-pT143-Nur77) was generated by ABclonal company (Wuhan, China) using Nur77 phosphopeptide, SAPSPS(pT)PSFQPPQ, derived from amino acids 137 to 150 in Nur77. The antibody was affinity-purified using the phosphorylated peptide-conjugated gels.

#### Phosphatase assays

To determine the nature of Nur77 modification during mitosis, HeLa cells were synchronized to mitosis by a double thymidine block and lysed on ice. After centrifugation, the supernatant was collected and incubated with thermosensitive Alkaline Phosphatase (TAP, Thermo) at 37°C for 30 min. Reactions were boiled in SDS sample loading buffer and analyzed by Western blotting.

#### Deglycosylation analysis

For deglycosylation assays, protein extracts from mitotic HeLa cells were treated with Peptide N-Glycosidase F (PNGase F) (P2318S, Beyotime) following the supplier’s instructions. In brief, samples were first denatured in PNGase F denaturing buffer at 100°C for 10 min. After cooling, the denatured lysates were supplemented with concentrated PNGase F reaction buffer, 0.5 mol/L sodium phosphate (pH 7.5), and 10% NP-40 (at a 1/10 volume ratio), followed by incubation with PNGase F at 37°C for 1 h The digestion was quenched by adding 2-mercaptoethanol-containing sample buffer, and the mixtures were subsequently heated at 100°C for 5 min prior to Western blotting.

#### Nur77 knocking down and knocking out

##### Nur77 siRNA approach

To knock down Nur77, siRNA duplexes targeting Nur77 were transfected into HeLa cells using Lipofectamine 2000 in serum-free culture medium. Nontargeting siRNA was used as a negative control. 4 h after transfection, the cell culture medium was changed to normal medium with 10% FBS. Cells transfected with siRNA for 36–48 h were collected and analyzed by Western blotting, flow cytometry, and immunofluorescence staining.

##### CRISPR/Cas9 genome editing

Nur77 knockout cells were generated by CRISPR/Cas9-based genome editing. Briefly, sgRNA targeting sequence of Nur77 (sequence: 5’-ACCTTCATGGACGGCTACAC- 3’; designed based on CRISPR design tool; website: http://crispr.mit.edu/) was cloned into vector pX330-U6-Chimeric_BB-CBh-hSpCas9. Cells were transfected with pX330-U6- Chimeric_BB-CBh-hSpCas9-Nur77 or empty vehicle vector pX330-U6-Chimeric_BB-CBh-hSpCas9 using Lipofectamine 2000. To screen positive clones, pEGFP-C1 plasmid was co-transfected with pX330 that contains a neomycin resistance cassette (Neor). Transfected cells were diluted and grown in culture medium with 800 μg/mL G418 for 14 days. Colonies were analyzed and screened by Western blotting using anti-Nur77 antibody.

##### Fluorescence-activated cell sorter (FACS) analysis

Cell cycle analysis was performed by FACS. Cells digested by trypsin were harvested, washed with PBS, and fixed with precooled 70% ethanol at 4°C overnight. The fixed cells were centrifuged at 3,000 rpm, washed twice with PBS, stained with 0.5 μg/mL 4’,6-diamidino-2-phenylindole (DAPI) in PBS for 10 min at room temperature, and analyzed by FACScan flow cytometer (Beckman Coulter) using CytExpert software. For studying the cell cycle profile of breast tumor tissues from MMTV-PyMT mice, breast tumor tissues should be surgically removed from mice first, then digested to obtain single cells. The procedure is described below.

##### Confocal microscopy

Cells grown in a 24-well plate with glass slides were fixed with -20°C methanol (4% paraformaldehyde as fixing agent for α-tubulin staining) for 10 min and washed with PBS buffer. Fixed cells were permeabilized with Triton X-100 (0.05% for HeLa cells; 0.1% for breast cancer cell lines) in PBS buffer for 8 min at 4°C, washed with PBS buffer and blocked with 1% bovine serum albumin (BSA) in PBS for 30 min at room temperature. For staining, cells were incubated with various primary antibodies at room temperature for 2 h, washed with PBS, and then incubated with FITC-labeled secondary antibody (1:200), Goat anti-mouse/rabbit IgG conjugated with Cy3 (1:200) and/or Cy5-labeled anti-rabbit IgG (1:400) at room temperature for 1 h, followed by nuclei staining with 4′,6-diamidino-2-phenylindole (DAPI) (1:10000 dilution). The images were taken under a Leica TCS SP8 confocal laser scanning microscope system (Leica Microsystems GmbH, Mannheim, Germany). Fields were chosen by moving the stage randomly without looking at the fluorescence signal to avoid bias. For quantification of immunofluorescence images, the investigator was blinded to the experimental conditions.

##### In situ proximity ligation assay

The In Situ Proximity Ligation Assay (PLA) was conducted in HeLa cells by using a Doulink assay kit (Sigma/Aldrich) as described before [82]. To study Nur77 interaction with PLK1 at the centrosome, we used pT143-Nur77 (1:600) and PLK1 (Invitrogen, 1:200) as primary antibody. For detecting the interaction between Nur77 and Cep192 at the centrosome, cells transfected with GFP-Cep192 were incubated with pT143-Nur77 antibody (1:600) and GFP antibody (1:1000).

##### Live cell imaging

HeLa cells and Nur77^–/–^HeLa cells co-transfected with GFP-α-Tubulin and mCherry-H2B plasmids were grown in 6-well glass bottom culture plate and synchronized through a double thymidine block. During the first thymidine release, cells were transfected with vector, Flag-tagged Nur77 or its mutants. For mitotic progression analysis by live cell imaging, cells were released into normal media and imaging was performed using Zeiss Celldiscoverer 7 with 20X/0.95 water immersion objective. Time-lapse movies were acquired at 4 min/frame for 16 h and data were processed with ZEN software (Zeiss).

##### Expression and purification of proteins

The mCherry-Nur77 and 6xHis-Nur77-LBD proteins expression and purification were described in our earlier study [16]. Nur77 and Nur77-T143A mutant were cloned into a modified version of a T7-promoter-pET expression vector. The base vector was engineered to include a 6×His tag followed with mCherry. The resulting expression constructs were confirmed by sequencing. The N-terminal 6×His-tagged mCherry-Nur77-T143A and mCherry-Nur77-T143E were directly expressed in Escherichia coli BL21 (DE3) cells. Transfected cells were cultured in LB medium at 37°C with 220 rpm shaking until OD600 reached ~ 0.8, protein expression was induced by 0.25 mM IPTG after cooling down to 16°C. The induced cells were grown at 16°C with 220 rpm shaking overnight. Cell pellets were harvested by centrifuge and lysed by sonication in binding buffer containing 50 mM Tris pH 8.0, 500 mM NaCl, 5 mM Imidazole, and supplemented with 1 mM PMSF. The homogenates were purified using Ni-NTA Sepharose (GE Healthcare) affinity column and eluted with 50 mM Tris pH 8.0, 500 mM NaCl, 300 mM imidazole. The eluted proteins were immediately purified by size exclusion chromatography using HiLoad 26/600 Superdex 200 pg column, pre-equilibrated in a buffer containing 50 mM Tris pH 8.0, 300 mM NaCl, 1 mM EDTA,1 mM DTT. Proteins were concentrated with Amicon ultra centrifugal filter (MWCO = 10 kD, Millipore) to ~10 mg/mL and aliquotted into small fractions. Proteins were then frozen in liquid nitrogen and moved to -80°C for long term storage.

##### In vitro kinase assays

HeLa cells transfected with Flag-Cdk1/Myc-cyclin B1, Flag-PLK1 or Flag-Aurora A were synchronized to mitosis by a double thymidine block. Mitotic Flag-tagged kinases above were extracted by immunoprecipitation assay using anti-Flag antibody. 1 μg purified 6xHis-mCherry-tagged Nur77 protein or its mutant (T143A) was incubated with Flag-Cdk1/Myc-cyclin B1, Flag-PLK1 or Flag-Aurora A immunoprecipitates in 40 μl kinase reaction buffer (50 mM Tris-HCl pH 8.0, 150 mM NaCl, 10 mM MgCl_2_, 100 mM DTT, 0.3 mM ATP) with or without RO-3306 (10 μM) treatment for 30 min at 30°C. Supernatant of the reaction was mixed with 10 μl 5× sample loading buffer and analyzed by Western blotting.

##### Microtubule regrowth assay

HeLa cells growing on coverslips were synchronized at mitosis by double-thymidine block. Then cells in dish were treated with 100 ng/mL nocodazole on ice for 60 min to completely depolymerize microtubules. After that, cells were washed with cold PBS three times to remove nocodazole, then cultured in 37°C warmed medium for the indicated times and analyzed by IF. For studying the effect of Nur77 on microtubule regrowth, HeLa cells were transfected with control or Nur77 siRNA during the second thymidine arrest. For NMA39 treatment, HeLa cells were treated with or without 1 μM NMA39 for indicated times when cells were reheated at 37°C.

##### Isolation and culture of primary mouse breast tumor cells

Mammary tumors were surgically removed from female MMTV-PyMT transgenic mice with large tumor burden (i.e., 12 weeks of age) by cervical dislocation, cut into small pieces, and incubated at 37°C for 1 h in DMEM medium containing DNase I (0.25 mg/mL), hyaluronidase Type I-S (1 mg/mL), and collagenase, Type IV (6 mg/mL). Tumor cell suspension was filtered through 70 µm cell strainers (Falcon; Franklin Lakes, NJ USA) and concentrated by centrifugation at 400 g for 5 min at room temperature. Cell pellet was resuspended and washed in DPBS three times. A small fraction of the cells was stained with trypan blue to evaluate cell viability using a hemocytometer under a microscope. Usually, 90–95% of the cells prepared from tumors were viable. After that, the isolated primary mouse breast tumor cells were resuspended and cultured with normal DMEM medium in an incubator at 37°C with 5% CO_2_.

##### MDA-MB-231 xenografts

MDA-MB-231 cells stably expressing pLenti-GFP, pLenti-GFP-Nur77, pLenti-GFP-Nur77-T143A, pLenti-GFP-Nur77-T143E were harvested, centrifuged at 1000 rpm, and resuspended in 1:2 PBS and Matrigel (Biocoat) mixtures at a density of 1.2 × 10^7^ cells/mL. Nude mice (BALB/c, 5 to 6 weeks old) were randomly assigned to different groups using a random number generator and then injected subcutaneously with these mixtures (100 μl per mouse). After 7 days, body weight and tumor sizes of mice were measured every 2 days, with tumor volume (V) calculated by the following formula: V = (width)^2^ ×length/2. The mice were sacrificed on the 21st day after injection. No blinding was performed during the animal experiments or outcome assessment.

##### MMTV-PyMT transgenic mouse model

For the tumorigenesis experiments using MMTV-PyMT-Nur77-KO mice, all mice were kept on a mixed but yet homogenous (50% C57BL/6 and 50% FVB/N) genetic background. FVB/N Nur77^+/−^ mice were produced by backcrossing for 10 generations from the original C57BL/6 Nur77^−/−^and maintained as heterozygotes. Heterozygous C57BL/6 Nur77^+/−^ females were crossed with FVB/N Nur77^+/−^ males carrying the MMTV-PyMT transgene to generate the experimental F1 animals. Genotypes were confirmed by tail snip PCR. Experimental and control littermates were co-housed throughout the experiments. For drug treatment, female MMTV-PyMT mice of 12 weeks old were randomly divided into two groups (*n* = 5 each). One group was administered with corn oil as vehicle control, the other group was administered with NMA39 (10 mg/kg) dissolved in corn oil via oral gavage once a day. Mice were sacrificed after 14 days of treatment and tumor tissues were surgically removed for various assessments. No blinding was performed during the animal experiments or outcome assessment.

##### Clinical breast tumor tissues and evaluation

For Western blotting analysis, human breast tumor tissues and the corresponding adjacent normal tissues (*n* = 36) were obtained by surgical resection from Zhongshan Hospital Affiliated to Xiamen University. For immunofluorescence assay, human breast hyperplastic tissue (*n* = 8) and breast tumor tissue (*n* = 7) were collected through surgical resection from the Fifth Affiliated Hospital of Sun Yat-sen University. All tissue samples were collected with written informed consent from all patients. The protocols were approved by the Ethical and Scientific Committee of the Hospitals.

##### Tissue process and histological examination

Tumors, livers, and hyperplastic tissues dissected from mice or humans were fixed overnight at 4 °C in 4% neutral buffered formalin phosphate (pH 7.0). After being dehydrated with graded ethanol and xylene, the fixed tumor tissues were subsequently embedded in paraffin on a tissue embedding machine (Leica) and then sliced into 4-μm sections. Tissue sections were heated at 60 °C for 30 min in the oven and deparaffinized by graded xylene and/or ethanol, followed by boiling in sodium citrate buffer five times (1.8 mM citric acid and 8.2 mM sodium citrate in PBS) for 5 min. Tissue sections were then treated with 3% H_2_O_2_ in methanol for 30 min in the dark, subsequently blocked by glycine (0.1 M in PBS) for 30 min, and 5% goat serum (Zsbio, Cat#ZLI-9056) for 1 h at RT. For immunostaining mouse tissue sections, sections were blocked by 1% BSA in PBS, and incubated with anti-pS10-H3 (1:200 dilution, CST) antibody diluted in 1% BSA overnight at 4 °C, followed by incubation with anti-goat IgG conjugated with Cy3 (1:200) secondary antibody and DAPI (1:5000 dilution). For immunostaining human tissue sections, sections were blocked by 1% BSA in PBS, and incubated with anti-pT143-Nur77 antibody (1:200 dilution) and anti-pS10-H3 (1:20 dilution, Santa Cruz) antibody diluted in 1% BSA overnight at 4 °C, followed by incubation with FITC-labeled secondary antibody (1:200) and anti-goat IgG conjugated with Cy3 (1:200) secondary antibody and DAPI (1:5000 dilution). Apoptosis was detected by TUNEL (YEASEN, cat#40306ES20) staining according to the manufacturer’s instructions. Positive cells per area were counted, measured, and analyzed for at least 10 fields using the Leica TCS SP8 system. For immunohistochemistry, sections were incubated with anti-cleaved-caspase-3 (1:100 dilution) antibody overnight at 4 °C and then incubated with secondary antibodies (Zsbio, Cat# PV-9001 and Cat# PV-9002) according to the manufacturer’s protocol. Sections were washed five times with PBS for 3 min after each incubation. Samples of sections were then exposed to DAB agent (Zsbio, Cat# ZLI-9017) for 15 s-5 min, and stained with hematoxylin (Zsbio, Cat# ZLI-9610) for 2 min followed by differentiation with acid alcohol (1% HCl). After washing with running water, the sections were fully dehydrated and fixed by neutral balsam (Sinopharm Chemical Reagent Co., Ltd). Positive cells per area were counted, measured and analyzed for at least 10 fields by an image analysis system (Olympus, Tokyo, Japan).

##### Synthesis of NMA39

Synthesis of (2 R,4aS,6aS,9S,12bR,14aS,14bR)-9-methoxy-2,4a,6a,9,12b,14a-hexamethyl-10,11-dioxo-1,2,3,4,4a,5,6,6a,9,10,11,12b,13,14,14a,14b-hexadecahydropicene-2-carboxylic acid (NMA36) and (2 R,4aS,6aS,9S,12bR,14aS,14bR)-9-methoxy-*N*,2,4a,6a,9,12b,14a-heptamethyl-10,11-dioxo-1,2,3,4,4a,5,6,6a,9,10,11,12b,13,14,14a,14b-hexadecahydropicene-2-carboxamide (NMA39) followed the scheme described (Supplementary Fig. 7A).

##### Synthesis of (2 R,4aS,6aS,9S,12bR,14aS,14bR)-9-methoxy-2,4a,6a,9,12b,14a-hexamethyl-10,11-dioxo-1,2,3,4,4a,5,6,6a,9,10,11,12b,13,14,14a,14b-hexadecahydropicene-2-carboxylic acid (cas number: 2624191-90-6)

To a solution of celastrol (200 mg, 0.44 mmol), dissolved in 20 mL (0.22 M) MeOH, then MeONa (190 μL, 30%wt in Methanol, *ρ* = 0.97 g/mL, 2.3 equiv) was added, the reaction was stirred at room temperature with air exposed for 2.5 h. Quenched by 1mol/L HCl (40mL), then removing the residual methyl alcohol under vacuum, the residues were extracted with ethyl acetate (20mL **×** 3) and washed with brine three times (15mL **×** 3). The organic layers were combined and dried with anhydrous sodium sulfate. And then purified by flash column chromatography on silica gel (Ethyl acetate/Hexane = 1:4, v/v) to produce NMA36. Light yellow solid, yield: 56%.

^1^H NMR (600 MHz, DMSO-*d*_*6*_) *δ* 12.07 (br. s., 1H), 6.47 (d, *J* = 6.60 Hz, 1H), 6.40 (s, 1H), 6.19 (d, *J* = 6.60 Hz, 1H), 3.17 (s, 3H), 2.32 (d, *J* = 15.59 Hz, 1H), 2.12 (d, *J* = 8.44 Hz, 1H), 2.04 (d, *J* = 13.75 Hz, 1H), 1.94 - 2.01 (m, 2H), 1.77 - 1.85 (m, 1H), 1.69 - 1.73 (m, 1H), 1.62 - 1.66 (m, *J* = 7.90 Hz, 2H), 1.52 - 1.55 (m, 2H), 1.48 - 1.52 (m, 1H), 1.44 (s, 3H), 1.42 (s, 3H), 1.30 (d, *J* = 4.58 Hz, 1H), 1.28 (s, 1H), 1.20 (s, 3H), 1.11 (s, 3H), 1.06 (s, 3H), 0.85 - 0.90 (m, 1H), 0.68 (s, 3H); ^13^C NMR (151 MHz, DMSO-*d*_*6*_) *δ* 195.9, 181.5, 179.4, 174.2, 162.3, 130.6, 126.7, 122.7, 116.0, 86.2, 53.5, 44.3, 43.6, 42.3, 39.4, 37.8, 36.1, 35.4, 34.4, 32.6, 32.4, 31.3, 30.2, 30.2, 29.4, 29.0, 28.0, 26.3, 22.3, 18.4 (Supplementary Fig. 7B, C); HRMS (ESI) *m/z*: calcd for C_30_H_41_O_5_^+^ [M + H]^+^: 481.2949, found: 481.2950 (Supplementary Fig. 7D).

##### Synthesis of (2 R,4aS,6aS,9S,12bR,14aS,14bR)-9-methoxy-*N*,2,4a,6a,9,12b,14a-heptamethyl-10,11-dioxo-1,2,3,4,4a,5,6,6a,9,10,11,12b,13,14,14a,14b-hexadecahydropicene-2-carboxamide (cas number: 2624191-96-2)

To a solution of NMA36 (600 mg, 1.25 mmol), PyBOP (780 mg, 1.5 mmol) stirred to dissolve in 24 mL DMF, then DIPEA (322.5 mg, 412 μL, 2.5 mmol) was added, followed with CH_3_NH_2_ (33% in absolutely ethanol, 528 μL, 3.75 mmol), the solution was kept to react at room temperature for 2 h. Quenched by Brine (50 mL), extracted with ethyl acetate (3 **×** 30 mL). The organic layers were combined and dried with anhydrous sodium sulfate. And then purified by flash column chromatography on silica gel (Ethyl acetate/Hexane = 1:2, v/v) to produce NMA39. Yellow orange solid, 544.6 mg, yield: 88.2%. ^1^H NMR (600 MHz, DMSO-*d*_*6*_) *δ* 7.55 (q, *J* = 4.03 Hz, 1H), 6.46 (dd, *J* = 1.28, 6.60 Hz, 1H), 6.41 (d, *J* = 1.47 Hz, 1H), 6.18 (d, *J* = 6.79 Hz, 1H), 3.16 (s, 3H), 2.45 (d, *J* = 4.40 Hz, 3H), 2.38 (d, *J* = 15.41 Hz, 1H), 2.07 - 2.11 (m, 1H), 2.05 (d, *J* = 14.67 Hz, 1H), 1.98 (dt, *J* = 3.67, 13.94 Hz, 2H), 1.78 - 1.84 (m, 1H), 1.74 - 1.78 (m, 1H), 1.62 - 1.68 (m, 1H), 1.57 - 1.62 (m, 1H), 1.54 - 1.57 (m, 1H), 1.50 - 1.54 (m, 2H), 1.48 (d, *J* = 7.89 Hz, 1H), 1.43 (s, 3H), 1.41 (s, 3H), 1.27 - 1.35 (m, 1H), 1.19 (s, 3H), 1.06 (s, 3H), 1.02 (s, 3H), 0.86 (d, *J* = 13.94 Hz, 1H), 0.56 (s, 3H); ^13^C NMR (151 MHz, DMSO-*d*_*6*_) *δ* 195.9, 181.5, 177.5, 174.2, 162.5, 130.6, 126.7, 122.7, 115.8, 86.2, 53.4, 44.3, 43.9, 42.3, 39.1, 37.8, 36.2, 35.3, 34.8, 33.2, 32.6, 31.4, 30.7, 30.2, 29.4, 28.8, 28.1, 26.4, 26.0, 22.2, 17.5 (Supplementary Fig. 7E, 7F). HRMS (ESI) *m/z*: calcd for C_31_H_44_NO_4_^+^ [M + H]^+^: 494.3265, found: 494.3265 (Supplementary Fig. 7G).

##### Surface plasmon resonance assay

The binding kinetics between celastrol derivative NMA39 and purified Nur77-LBD protein was analyzed on a Biacore T200 machine with CM chips (GE Healthcare) as described before [15]. NMA39 was tested with a gradient concentration of 0.5 μM, 1 μM, 2 μM, 4 μM and 8 μM injected through flow cells immobilized with Nur77-LBD protein. The kinetic profiles were shown.

### Quantification and statistical analysis

Quantitative data were represented as mean ± SEM from at least three independently repeated experiments. No samples were excluded from the analysis. All biological replicates that met quality control standards (cell viability >90%, successful protein extraction) were included. Homogeneity of variance was assessed using Levene’s test. Two-tailed unpaired Student’s *t* test was used for statistical analysis using the GraphPad Prism 10.0 software. *p* values < 0.05 were regarded as statistically significant (*), *p* values < 0.01 as highly significant (**), *p* values < 0.001 as extremely significant (***), *p* values < 0.0001 as more extremely significant (****), and *ns* as not significant.

## Supplementary information

Nur77-mitosis-Supplemental Figures-revised

Table S1

Original data

## Data Availability

All other relevant data supporting the key findings of this study are available within the article and its Supplementary Information files or from the corresponding author upon reasonable request. Source data are provided with this paper.

## References

[1] Wang Y, Li N, Guan W, Wang D. Controversy and multiple roles of the solitary nucleus receptor Nur77 in disease and physiology. FASEB J. 2025;39:e70468. 40079203 10.1096/fj.202402775RR PMC11904867

[2] Herring JA, Elison WS, Tessem JS. Function of Nr4a orphan nuclear receptors in proliferation, apoptosis and fuel utilization across tissues. Cells. 2019;8:1373. 31683815 10.3390/cells8111373 PMC6912296

[3] Rodríguez-Calvo R, Tajes M, Vázquez-Carrera M. The NR4A subfamily of nuclear receptors: potential new therapeutic targets for the treatment of inflammatory diseases. Expert Opin Ther Targets. 2017;21:291–304. 28055275 10.1080/14728222.2017.1279146

[4] Zhao L, Hu H, Gustafsson J-Å, Zhou S. Nuclear receptors in cancer inflammation and immunity. Trends Immunol. 2020;41:172–85. 31982345 10.1016/j.it.2019.12.006

[5] Chen L, Fan F, Wu L, Zhao Y. The nuclear receptor 4A family members: mediators in human disease and autophagy. Cell Mol Biol Lett. 2020;25:48. 33292165 10.1186/s11658-020-00241-w PMC7640683

[6] Zhang X-k. Targeting Nur77 translocation. Expert Opin Ther Targets. 2007;11:69–79. 17150035 10.1517/14728222.11.1.69

[7] Hsu H-C, Zhou T, Mountz JD. Nur77 family of nuclear hormone receptors. Curr Drug Targets Inflamm Allergy. 2004;3:413–23. 15584889 10.2174/1568010042634523

[8] Flaig R, Greschik H, Peluso-Iltis C, Moras D. Structural basis for the cell-specific activities of the NGFI-B and the Nurr1 ligand-binding domain. J Biol Chem. 2005;280:19250–8. 15716272 10.1074/jbc.M413175200

[9] Wansa KD, Harris JM, Muscat GE. The activation function-1 domain of Nur77/NR4A1 mediates trans-activation, cell specificity, and coactivator recruitment. J Biol Chem. 2002;277:33001–11. 12082103 10.1074/jbc.M203572200

[10] Maxwell MA, Muscat GE. The NR4A subgroup: immediate early response genes with pleiotropic physiological roles. Nucl Recept Signal. 2006;4:e002. 16604165 10.1621/nrs.04002 PMC1402209

[11] Kurakula K, Koenis DS, van Tiel CM, de Vries CJ. NR4A nuclear receptors are orphans but not lonesome. Biochim Biophys Acta. 2014;1843:2543–55. 24975497 10.1016/j.bbamcr.2014.06.010

[12] Mohan HM, Aherne CM, Rogers AC, Baird AW, Winter DC, Murphy EP. Molecular pathways: the role of NR4A orphan nuclear receptors in cancer. Clin Cancer Res. 2012;18:3223–8. 22566377 10.1158/1078-0432.CCR-11-2953

[13] Li H, Kolluri SK, Gu J, Dawson MI, Cao X, Hobb PD, et al. Cytochrome c release and apoptosis induced by mitochondrial targeting of nuclear orphan receptor TR3. Science. 2000;289:1159–64. 10947977 10.1126/science.289.5482.1159

[14] Lin B, Kolluri SK, Lin F, Liu W, Han Y-H, Cao X, et al. Conversion of Bcl-2 from protector to killer by interaction with nuclear orphan receptor Nur77/TR3. Cell. 2004;116:527–40. 14980220 10.1016/s0092-8674(04)00162-x

[15] Hu M, Luo Q, Alitongbieke G, Chong S, Xu C, Xie L, et al. Celastrol-induced Nur77 interaction with TRAF2 alleviates inflammation by promoting mitochondrial ubiquitination and autophagy. Mol Cell. 2017;66:141–53.e6. 28388439 10.1016/j.molcel.2017.03.008 PMC5761061

[16] Peng SZ, Chen XH, Chen SJ, Zhang J, Wang CY, Liu WR, et al. Phase separation of Nur77 mediates celastrol-induced mitophagy by promoting the liquidity of p62/SQSTM1 condensates. Nat Commun. 2021;12:5989. 34645818 10.1038/s41467-021-26295-8 PMC8514450

[17] Kolluri SK, Zhu X, Zhou X, Lin B, Chen Y, Sun K, et al. A short Nur77-derived peptide converts Bcl-2 from a protector to a killer. Cancer Cell. 2008;14:285–98. 18835031 10.1016/j.ccr.2008.09.002 PMC2667967

[18] Safe S, Shrestha R, Mohankumar K. Orphan nuclear receptor 4A1 (NR4A1) and novel ligands. Essays Biochem. 2021;65:877–86. 34096590 10.1042/EBC20200164 PMC11410023

[19] Wu L, Chen L. Characteristics of Nur77 and its ligands as potential anticancer compounds (Review). Mol Med Rep. 2018;18:4793–801. 30272297 10.3892/mmr.2018.9515 PMC6236262

[20] Oany AR, Upadhyay S, Tsui WNT, Hailemariam A, Latka S, Landua JD. Orphan nuclear receptor 4A1 (NR4A1) and NR4A2 are endogenous regulators of CD71 and their ligands induce ferroptosis in breast cancer. Cell Death Disease. 2025;16:776. 41184246 10.1038/s41419-025-08143-5 PMC12583510

[21] Safe S, Karki K. The paradoxical roles of orphan nuclear receptor 4A (NR4A) in cancer. Mol Cancer Res. 2021;19:180–91. 33106376 10.1158/1541-7786.MCR-20-0707 PMC7864866

[22] Matthews HK, Bertoli C, de Bruin RAM. Cell cycle control in cancer. Nat Rev Mol Cell Biol. 2022;23:74–88. 34508254 10.1038/s41580-021-00404-3

[23] Malumbres M, Barbacid M. Cell cycle, CDKs and cancer: a changing paradigm. Nat Rev Cancer. 2009;9:153–66. 19238148 10.1038/nrc2602

[24] Nigg EA. Mitotic kinases as regulators of cell division and its checkpoints. Nat Rev Mol Cell Biol. 2001;2:21–32. 11413462 10.1038/35048096

[25] Zitouni S, Nabais C, Jana SC, Guerrero A, Bettencourt-Dias M. Polo-like kinases: structural variations lead to multiple functions. Nat Rev Mol Cell Biol. 2014;15:433–52. 24954208 10.1038/nrm3819

[26] Kapadia N, Nurse P. Spatiotemporal orchestration of mitosis by cyclin-dependent kinase. Nature. 2025;643:1391–99. 40562936 10.1038/s41586-025-09172-y PMC12310531

[27] Milletti G, Colicchia V, Cecconi F. Cyclers’ kinases in cell division: from molecules to cancer therapy. Cell Death Differ. 2023;30:2035–52. 37516809 10.1038/s41418-023-01196-z PMC10482880

[28] Conduit PT, Wainman A, Raff JW. Centrosome function and assembly in animal cells. Nat Rev Mol Cell Biol. 2015;16:611–24. 26373263 10.1038/nrm4062

[29] Nam H-J, Naylor RM, van Deursen JM. Centrosome dynamics as a source of chromosomal instability. Trends Cell Biol. 2015;25:65–73. 25455111 10.1016/j.tcb.2014.10.002 PMC4312731

[30] Joukov V, Walter JC, De Nicolo A. The Cep192-organized aurora A-Plk1 cascade is essential for centrosome cycle and bipolar spindle assembly. Mol Cell. 2014;55:578–91. 25042804 10.1016/j.molcel.2014.06.016 PMC4245277

[31] Meng L, Park J-E, Kim T-S, Lee EH, Park S-Y, Zhou M, et al. Bimodal interaction of mammalian polo-like kinase 1 and a centrosomal scaffold, Cep192, in the regulation of bipolar spindle formation. Mol Cell Biol. 2015;35:2626–40. 26012549 10.1128/MCB.00068-15 PMC4524112

[32] Kolluri SK, Bruey-Sedano N, Cao X, Lin B, Lin F, Han YH, et al. Mitogenic effect of orphan receptor TR3 and its regulation by MEKK1 in lung cancer cells. Mol Cell Biol. 2003;23:8651–67. 14612408 10.1128/MCB.23.23.8651-8667.2003 PMC262666

[33] Guo H, Golczer G, Wittner BS, Langenbucher A, Zachariah M, Dubash TD, et al. NR4A1 regulates expression of immediate early genes, suppressing replication stress in cancer. Mol Cell. 2021;81:4041–58.e15. 34624217 10.1016/j.molcel.2021.09.016 PMC8549465

[34] Ong S-E, Mann M. A practical recipe for stable isotope labeling by amino acids in cell culture (SILAC). Nat Protoc. 2007;1:2650–60. 10.1038/nprot.2006.427 17406521

[35] Fang G, Yu H, Kirschner MW. Direct binding of CDC20 protein family members activates the anaphase-promoting complex in mitosis and G1. Mol Cell. 1998;2:163–71. 9734353 10.1016/s1097-2765(00)80126-4

[36] Zieve GW, Turnbull D, Mullins JM, McIntosh JR. Production of large numbers of mitotic mammalian cells by use of the reversible microtubule inhibitor nocodazole: nocodazole accumulated mitotic cells. Exp Cell Res. 1980;126:397–405. 6153987 10.1016/0014-4827(80)90279-7

[37] Xie G, Zhou Y, Tu X, Ye X, Xu L, Xiao Z, et al. Centrosomal localization of rxrα promotes plk1 activation and mitotic progression and constitutes a tumor vulnerability. Dev cell. 2020;55:707–22.e9. 33321102 10.1016/j.devcel.2020.11.012

[38] Elia AEH, Cantley LC, Yaffe MB. Proteomic screen finds pSer/pThr-binding domain localizing Plk1 to mitotic substrates. science. 2003;299:1228–331. 12595692 10.1126/science.1079079

[39] Elia AEH, Rellos P, Haire LF, Chao JW, Ivins FJ, Hoepker k, et al. The molecular basis for phosphodependent substrate targeting and regulation of Plks by the Polo-Box domain. Cell. 2003;115:83–95. 14532005 10.1016/s0092-8674(03)00725-6

[40] Matthess Y, Raab M, Knecht R, Becker S, Strebhardt K. Sequential Cdk1 and Plk1 phosphorylation of caspase-8 triggers apoptotic cell death during mitosis. Mol Oncol. 2014;8:596–608. 24484936 10.1016/j.molonc.2013.12.013 PMC5528627

[41] Wen D, Wu J, Wang L, Fu Z. SUMOylation promotes nuclear import and stabilization of polo-like kinase 1 to support its mitotic function. Cell Rep. 2017;21:2147–59. 29166606 10.1016/j.celrep.2017.10.085 PMC5728694

[42] Singh P, Pesenti ME, Maffini S, Carmignani S, Hedtfeld M, Petrovic A, et al. BUB1 and CENP-U, primed by CDK1, are the main PLK1 kinetochore receptors in mitosis. Mol Cell. 2021;81:67–87.e9. 33248027 10.1016/j.molcel.2020.10.040 PMC7837267

[43] Holt LJ, Tuch BB, Villén J, Johnson AD, Gygi SP, Morgan DO. Global analysis of Cdk1 substrate phosphorylation sites provides insights into evolution. Science. 2009;325:1682–6. 19779198 10.1126/science.1172867 PMC2813701

[44] Li L, Liu Y, Chen HZ, Li FW, Wu JF, Zhang HK, et al. Impeding the interaction between Nur77 and p38 reduces LPS-induced inflammation. Nat Chem Biol. 2015;11:339–46. 25822914 10.1038/nchembio.1788

[45] Han YH, Cao X, Lin B, Lin F, Kolluri SK, Stebbins J, et al. Regulation of Nur77 nuclear export by c-Jun N-terminal kinase and Akt. Oncogene. 2006;25:2974–86. 16434970 10.1038/sj.onc.1209358

[46] Chen H-Z, Liu Q-F, Li L, Wang W-J, Yao L-M, Yang M, et al. The orphan receptor TR3 suppresses intestinal tumorigenesis in mice by downregulating Wnt signalling. Gut. 2012;61:714–24. 21873734 10.1136/gutjnl-2011-300783

[47] Pekarsky Y, Hallas C, Palamarchuk A, Koval A, Bullrich F, Hirata Y, et al. Akt phosphorylates and regulates the orphan nuclear receptor Nur77. Proc Natl Acad Sci. 2001;98:3690–4. 11274386 10.1073/pnas.051003198 PMC31113

[48] Örd M, Venta R, Möll K, Valk E, Loog M. Cyclin-specific docking mechanisms reveal the complexity of M-CDK function in the cell cycle. Mol Cell. 2019;75:76–89.e3. 31101497 10.1016/j.molcel.2019.04.026 PMC6620034

[49] Moll UM, Marchenko N, Zhang XK. p53 and Nur77/TR3 - transcription factors that directly target mitochondria for cell death induction. Oncogene. 2006;25:4725–43. 16892086 10.1038/sj.onc.1209601

[50] Söderberg O, Gullberg M, Jarvius M, Ridderstråle K, Leuchowius K-J, Jarvius J, et al. Direct observation of individual endogenous protein complexes in situ by proximity ligation. Nat Methods. 2006;3:995–1000. 17072308 10.1038/nmeth947

[51] Gomez-Ferreria MA, Rath U, Buster DW, Chanda SK, Caldwell JS, Rines DR, et al. Human Cep192 is required for mitotic centrosome and spindle assembly. Curr Biol. 2007;17:1960–6. 17980596 10.1016/j.cub.2007.10.019

[52] Maiato H, Logarinho E. Mitotic spindle multipolarity without centrosome amplification. Nat Cell Biol. 2014;16:386–94. 24914434 10.1038/ncb2958

[53] Kollman JM, Merdes A, Mourey L, Agard DA. Microtubule nucleation by γ-tubulin complexes. Nat Rev Mol Cell Biol. 2011;12:709–21. 21993292 10.1038/nrm3209 PMC7183383

[54] Lin T-c, Neuner A, Schiebel E. Targeting of γ-tubulin complexes to microtubule organizing centers: conservation and divergence. Trends Cell Biol. 2015;25:296–307. 25544667 10.1016/j.tcb.2014.12.002

[55] Joukov V, Nicolo AD. Aurora-PLK1 cascades as key signaling modules in the regulation of mitosis. Sci Signal. 2018;11:eaar4195. 30108183 10.1126/scisignal.aar4195

[56] Gönczy P. Centrosomes and cancer: revisiting a long-standing relationship. Nat Rev Cancer. 2015;15:639–52. 26493645 10.1038/nrc3995

[57] Crasta K, Ganem NJ, Dagher R, Lantermann AB, Ivanova EV, Pan Y, et al. DNA breaks and chromosome pulverization from errors in mitosis. Nature. 2012;482:53–8. 22258507 10.1038/nature10802 PMC3271137

[58] Dominguez-Brauer C, Thu KL, Mason JM, Blaser H, Bray MR, Mak TW. Targeting mitosis in cancer: emerging strategies. Mol Cell. 2015;60:524–36. 26590712 10.1016/j.molcel.2015.11.006

[59] Chan KS, Koh CG, Li HY. Mitosis-targeted anti-cancer therapies: where they stand. Cell Death Dis. 2012;3:e411–e. 23076219 10.1038/cddis.2012.148 PMC3481136

[60] Chunduri NK, Storchová Z. The diverse consequences of aneuploidy. Nat Cell Biol. 2019;21:54–62. 30602769 10.1038/s41556-018-0243-8

[61] Sansregret L, Vanhaesebroeck B, Swanton C. Determinants and clinical implications of chromosomal instability in cancer. Nat Rev Clin Oncol. 2018;15:139–50. 29297505 10.1038/nrclinonc.2017.198

[62] Goundiam O, Basto R. Centrosomes in disease: how the same music can sound so different?. Curr Opin Struct Biol. 2021;66:74–82. 33186811 10.1016/j.sbi.2020.09.011

[63] Galluzzi L, Vitale I, Aaronson SA, Abrams JM, Adam D, Agostinis P, et al. Molecular mechanisms of cell death: recommendations of the Nomenclature Committee on Cell Death 2018. Cell Death Differ. 2018;25:486–541. 29362479 10.1038/s41418-017-0012-4 PMC5864239

[64] Sazonova EV, Petrichuk SV, Kopeina GS, Zhivotovsky B. A link between mitotic defects and mitotic catastrophe: detection and cell fate. Biol Direct. 2021;16:25. 34886882 10.1186/s13062-021-00313-7 PMC8656038

[65] Zhang D, Chen Z, Hu C, Yan S, Li Z, Lian B, et al. Celastrol binds to its target protein via specific noncovalent interactions and reversible covalent bonds. Chem Commun. 2018;54:12871–4. 10.1039/c8cc06140h 30376017

[66] Chen Z, Zhang D, Yan S, Hu C, Huang Z, Li Z, et al. SAR study of celastrol analogs targeting Nur77-mediated inflammatory pathway. Eur J Med Chem. 2019;177:171–87. 31132532 10.1016/j.ejmech.2019.05.009

[67] Oh E, Mark KG, Mocciaro A, Watson ER, Prabu JR, Cha DD, et al. Gene expression and cell identity controlled by anaphase-promoting complex. Nature. 2020;579:136–40. 32076268 10.1038/s41586-020-2034-1 PMC7402266

[68] Egli D, Birkhoff G, Eggan K. Mediators of reprogramming: transcription factors and transitions through mitosis. Nat Rev Mol Cell Biol. 2008;9:505–16. 18568039 10.1038/nrm2439 PMC7250051

[69] M.Gottesfeld J, Forbes DJ. Mitotic repression of the transcriptional machinery. Trends Biochem Sci. 1997;22:197–202. 9204705 10.1016/s0968-0004(97)01045-1

[70] Martinez-Balbis MA, Dey A, Rabindran SK, Ozato K, Wu C. Displacement of sequence-specific transcription factors from mitotic chromatin. Cell. 1995;83:29–38. 7553870 10.1016/0092-8674(95)90231-7

[71] Ong JY, Torres JZ. Phase separation in cell division. Mol Cell. 2020;80:9–20. 32860741 10.1016/j.molcel.2020.08.007 PMC7541545

[72] Yeh HW, Chen PP, Yeh TC, Lin SL, Chen YT, Lin WP, et al. Cep57 regulates human centrosomes through multivalent interactions. Proc Natl Acad Sci USA. 2024;121:e2305260121. 38857398 10.1073/pnas.2305260121 PMC11194501

[73] Woodruff JB, Ferreira Gomes B, Widlund PO, Mahamid J, Honigmann A, Hyman AA. The centrosome is a selective condensate that nucleates microtubules by concentrating tubulin. Cell. 2017;169:1066–77.e10. 28575670 10.1016/j.cell.2017.05.028

[74] Otto T, Sicinski P. Cell cycle proteins as promising targets in cancer therapy. Nat Rev Cancer. 2017;17:93–115. 28127048 10.1038/nrc.2016.138 PMC5345933

[75] Cao J, Liang C, Yu H. Aneuploidy as a cancer vulnerability. Curr Opin Cell Biol. 2025;94:102490. 40054068 10.1016/j.ceb.2025.102490

[76] Zhou F, Drabsch Y, Dekker TJA, de Vinuesa AG, Li Y, Hawinkels LJAC, et al. Nuclear receptor NR4A1 promotes breast cancer invasion and metastasis by activating TGF-β signalling. Nat Commun. 2014;5. 10.1038/ncomms4388 24584437

[77] Asghar U, Witkiewicz AK, Turner NC, Knudsen ES. The history and future of targeting cyclin-dependent kinases in cancer therapy. Nat Rev Drug Discov. 2015;14:130–46. 25633797 10.1038/nrd4504 PMC4480421

[78] Strebhardt K. Multifaceted polo-like kinases: drug targets and antitargets for cancer therapy. Nat Rev Drug Discov. 2010;9:643–60. 20671765 10.1038/nrd3184

[79] Hu Y, Zhan Q, Liu H-X, Chau T, Li Y, Yvonne Wan Y-J. Accelerated partial hepatectomy–induced liver cell proliferation is associated with liver injury in Nur77 knockout mice. Am J Pathol. 2014;184:3272–83. 25307349 10.1016/j.ajpath.2014.08.002 PMC4258495

[80] Moser SandraC, Bensaddek D, Ortmann B, Maure J-F, Mudie S, Blow JJ, et al. PHD1 links cell-cycle progression to oxygen sensing through hydroxylation of the centrosomal protein Cep192. Dev cell. 2013;26:381–92. 23932902 10.1016/j.devcel.2013.06.014 PMC3757158

[81] Song H, Kim EH, Hong J, Gwon D, Kim JW, Bae G-U, et al. Hornerin mediates phosphorylation of the polo-box domain in Plk1 by Chk1 to induce death in mitosis. Cell Death Differ. 2023;30:2151–66. 37596441 10.1038/s41418-023-01208-y PMC10482915

[82] Xie G, Zhou Y, Du M, Wang Q, Yi J, Zhang X-k. Protocol to identify centrosome-associated transcription factors during mitosis in mammalian cell lines. STAR Protoc. 2021;2:100495. 34195669 10.1016/j.xpro.2021.100495 PMC8225967

