# Supplementary material for: Cdk1-phosphorylated Nur77 accumulates at the centrosome during mitosis to regulate the Cep192–PLK1 signaling axis
Source: Cell Death Dis. 2026 Jun 3;17(1):695. doi: 10.1038/s41419-026-08870-3 (PMC13448657; doi:10.1038/s41419-026-08870-3)
Supplement: Supplementary file 1 [file 41419_2026_8870_MOESM1_ESM.pdf]

Figure S1

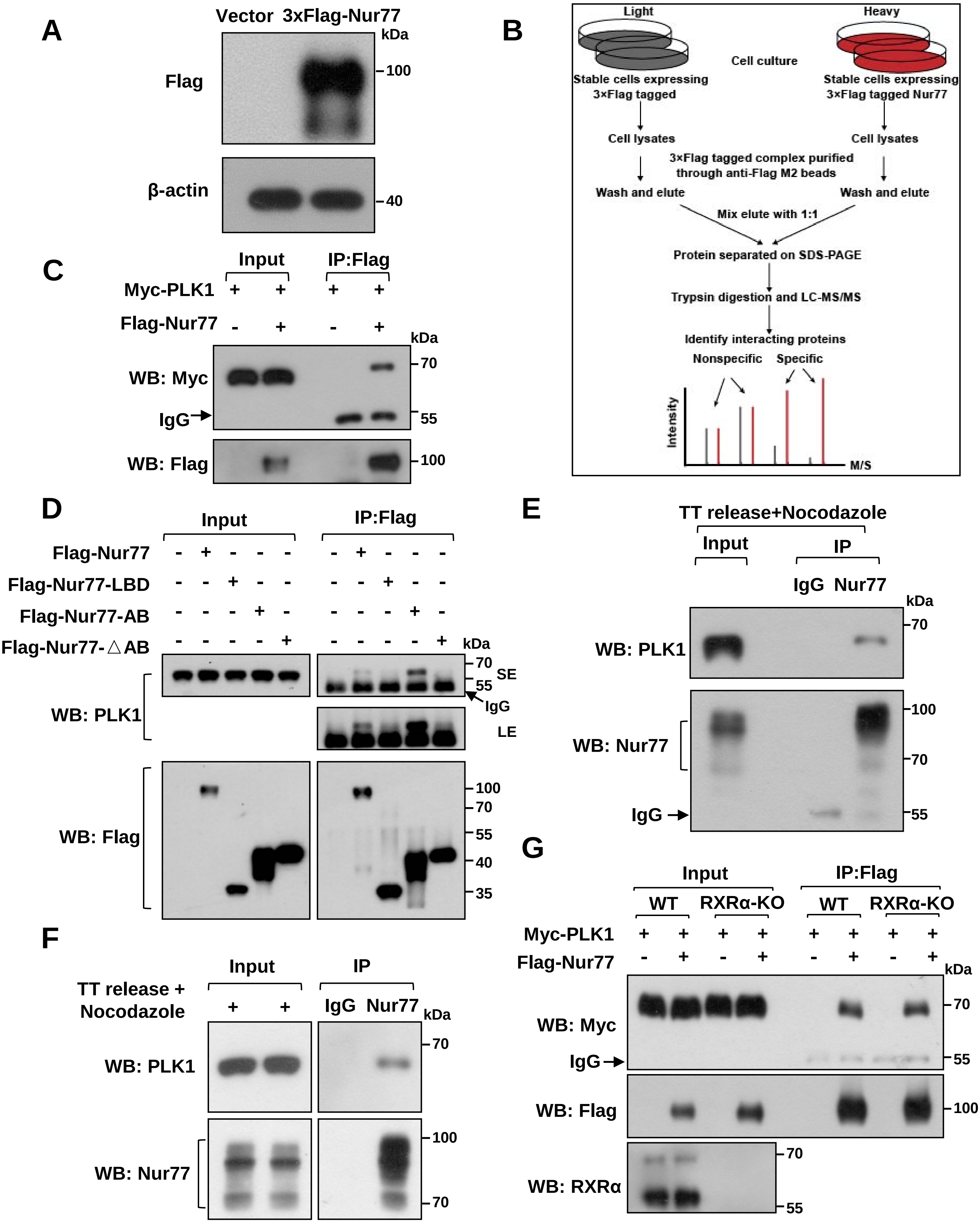

A
Vector
3xFlag-Nur77
kDa
Flag
100
β-actin
40
B
Light
Heavy
Cell culture
Stable cells expressing
3×Flag tagged
Stable cells expressing
3×Flag tagged Nur77
Cell lysates
Cell lysates
3×Flag tagged complex purified
through anti-Flag M2 beads
Wash and elute
Wash and elute
Mix elute with 1:1
Protein separated on SDS-PAGE
Trypsin digestion and LC-MS/MS
Identify interacting proteins
Nonspecific
Specific
Intensity
M/S
C
Input
IP:Flag
Myc-PLK1 + + + +
Flag-Nur77 - + - +
kDa
WB: Myc
70
IgG
55
WB: Flag
100
D
Input
IP:Flag
Flag-Nur77 - + - - - - + - - -
Flag-Nur77-LBD - - + - - - - + - -
Flag-Nur77-AB - - - + - - - - + -
Flag-Nur77-△AB - - - - + - - - - +
kDa
70
SE
55
IgG
LE
WB: PLK1
WB: Flag
100
70
55
40
35
E
TT release+Nocodazole
Input
IP
IgG
Nur77
kDa
WB: PLK1
70
WB: Nur77
100
70
IgG
55
F
Input
IP
TT release +
Nocodazole
+ +
IgG
Nur77
kDa
WB: PLK1
70
WB: Nur77
100
70
G
Input
IP:Flag
WT
RXRα-KO
WT
RXRα-KO
Myc-PLK1 + + + + + + + +
Flag-Nur77 - + - + - + - +
kDa
WB: Myc
70
IgG
55
WB: Flag
100
WB: RXRα
70
55

Figure S1. Identification of Nur77-interacting proteins and confirming the interaction of Nur77 with PLK1.

(A) HeLa cells stably expressing 3xFlag-tagged Nur77 were used to identify Nur77-interacting proteins. WB analysis of the expression of 3xFlag-Nur77 in the stable cell line.

(B) Illustration of stable isotope labeling with amino acids in cell culture (SILAC)-immunoprecipitation Quantitative Proteomics to identify Nu77-interacting proteins.

(C) Co-immunoprecipitation (CoIP) analysis of transfected Flag-Nur77 interaction with Myc-PLK1 in HeLa cells.

(D) Transfected Flag-tagged Nur77 mutants interaction with endogenous PLK1 were analyzed by CoIP assay after HeLa cells were released from a double thymidine block (TT block, 2 mM) for 10 hr. SE, short-time exposure; LE, long-time exposure.

(E, F) Endogenous Nur77 interaction with PLK1 during mitosis was analyzed by CoIP assay after HeLa cells (**E**) or MCF-7 (**F**) cells were released from TT block followed by nocodazole (100 ng/mL) treatment for 10 hr.

(G) Interaction of Flag-Nur77 with Myc-PLK1 in HeLa cells (wild type, WT) and RXRα deficiency HeLa cells (RXRα-KO) was analyzed by CoIP assay.

Figure S2

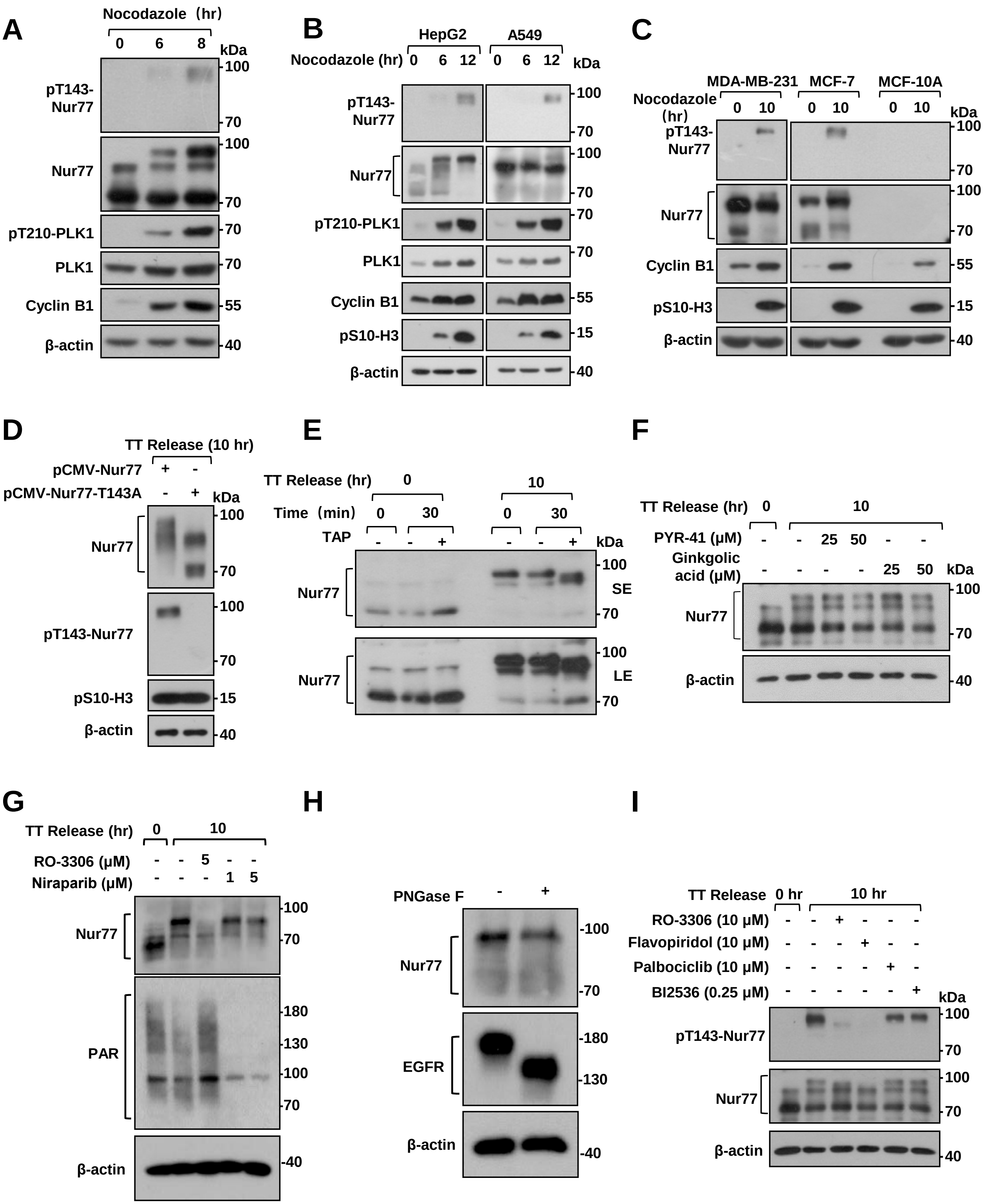

A
Nocodazole (hr)
0 6 8
kDa
pT143-Nur77
Nur77
pT210-PLK1
PLK1
Cyclin B1
β-actin
100
70
100
70
70
70
55
40
B
HepG2
A549
Nocodazole (hr) 0 6 12 0 6 12
kDa
pT143-Nur77
Nur77
pT210-PLK1
PLK1
Cyclin B1
pS10-H3
β-actin
100
70
100
70
70
70
55
15
40
C
MDA-MB-231
MCF-7
MCF-10A
Nocodazole (hr)
0 10 0 10 0 10
kDa
pT143-Nur77
Nur77
Cyclin B1
pS10-H3
β-actin
100
70
100
70
55
15
40
D
TT Release (10 hr)
pCMV-Nur77 + -
pCMV-Nur77-T143A - +
kDa
Nur77
pT143-Nur77
pS10-H3
β-actin
100
70
100
70
15
40
E
TT Release (hr) 0 10
Time (min) 0 30 0 30
TAP - - + - - +
kDa
Nur77
SE
Nur77
LE
100
70
100
70
F
TT Release (hr) 0 10
PYR-41 (μM) - - 25 50 - -
Ginkgolic acid (μM) - - - - 25 50
kDa
Nur77
β-actin
100
70
40
G
TT Release (hr) 0 10
RO-3306 (μM) - - 5 - -
Niraparib (μM) - - - 1 5
Nur77
PAR
β-actin
100
70
180
130
100
70
40
H
PNGase F - +
Nur77
EGFR
β-actin
100
70
180
130
40
I
TT Release 0 hr 10 hr
RO-3306 (10 μM) - - + - - -
Flavopiridol (10 μM) - - - + - -
Palbociclib (10 μM) - - - - + -
BI2536 (0.25 μM) - - - - - +
kDa
pT143-Nur77
Nur77
β-actin
100
70
100
70
40

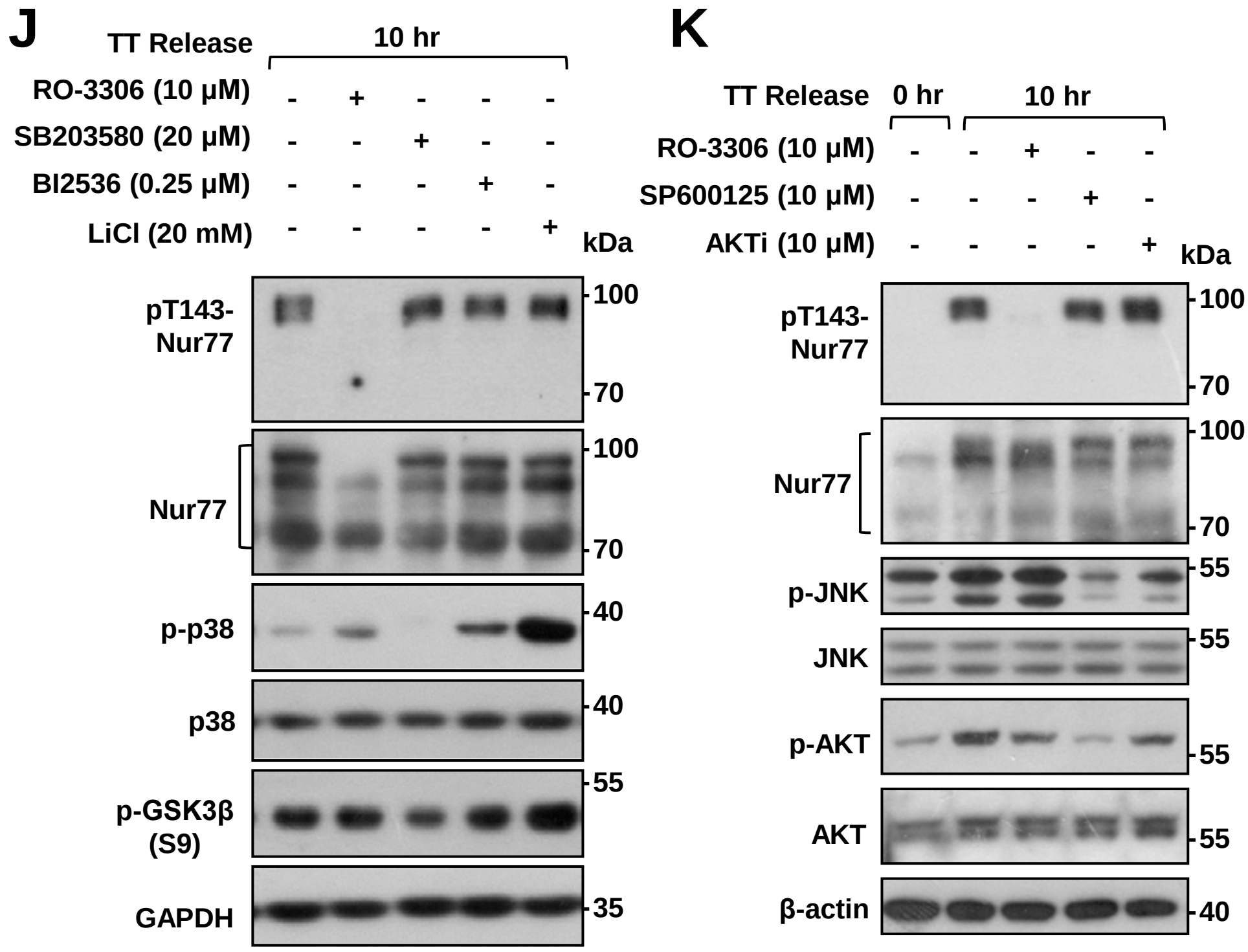

L

Motif #1 ^{458}EGK**LIF**CSG

Motif #2 ^{483}DSI**LAF**SRS

Motif #3 ^{589}KIFMDT**LPF**

"LXF" Motif

Figure S2. Cdk1 is responsible for the Thr143 phosphorylation of Nur77 during mitosis.

(A) WB analysis of HeLa cells treated with nocodazole (100 ng/mL) for the indicated time.

(B, C) Thr143 of Nur77 is phosphorylated during mitosis. Various cell lines treated with nocodazole (100 ng/mL) for the indicated time were analyzed by WB.

(D) Exogenous non-tagged Nur77 and its mutant expressed in miotic HeLa cells were analyzed by WB.

(E) Phosphorylation is responsible for Nur77 modification. WB analysis of thermosensitive alkaline phosphatase (TAP) effect on the mobility shift of Nur77 protein expressed in mitotic HeLa cells.

(F) Ubiquitination and sumoylation are not responsible for Nur77 modification. Mitotic HeLa cells released from the TT block were treated with PYR-41 (ubiquitin-activating enzyme inhibitor) or Ginkgolic acid (sumoylation inhibitor) for 30 min and analyzed by WB.

(G) Mitotic HeLa cells were treated with the Cdk1 inhibitor RO-3306 (5 μM, 1 hr) and the PARP inhibitor Niraparib (1 or 5 μM, 1 hr) and then analyzed by WB.

(H) Lysates from mitotic HeLa cells were treated with PNGase F for 1 hr at 37°C in vitro and then analyzed by WB. Epidermal growth factor receptor (EGFR), a well-characterized transmembrane glycoprotein, served as a positive control.

(I-K) Cdk1 but not other kinase is responsible for Thr143 phosphorylation of Nur77 during mitosis. HeLa cells released from the TT block for 10 hr were treated with the indicated kinase inhibitors for 15 min and analyzed by WB. RO-3306, Cdk1 inhibitor. Flavopiridol, Cdk1/2/4 inhibitor. Palbociclib, Cdk4/6 inhibitor. BI2536, PLK1 inhibitor. SB203580, p38 inhibitor. LiCl, GSK3β inhibitor. SP600125, JNK inhibitor. AKTi, AKT inhibitor.

(L) Three putative cyclin-binding motifs (LXF) in Nur77.

Figure S3

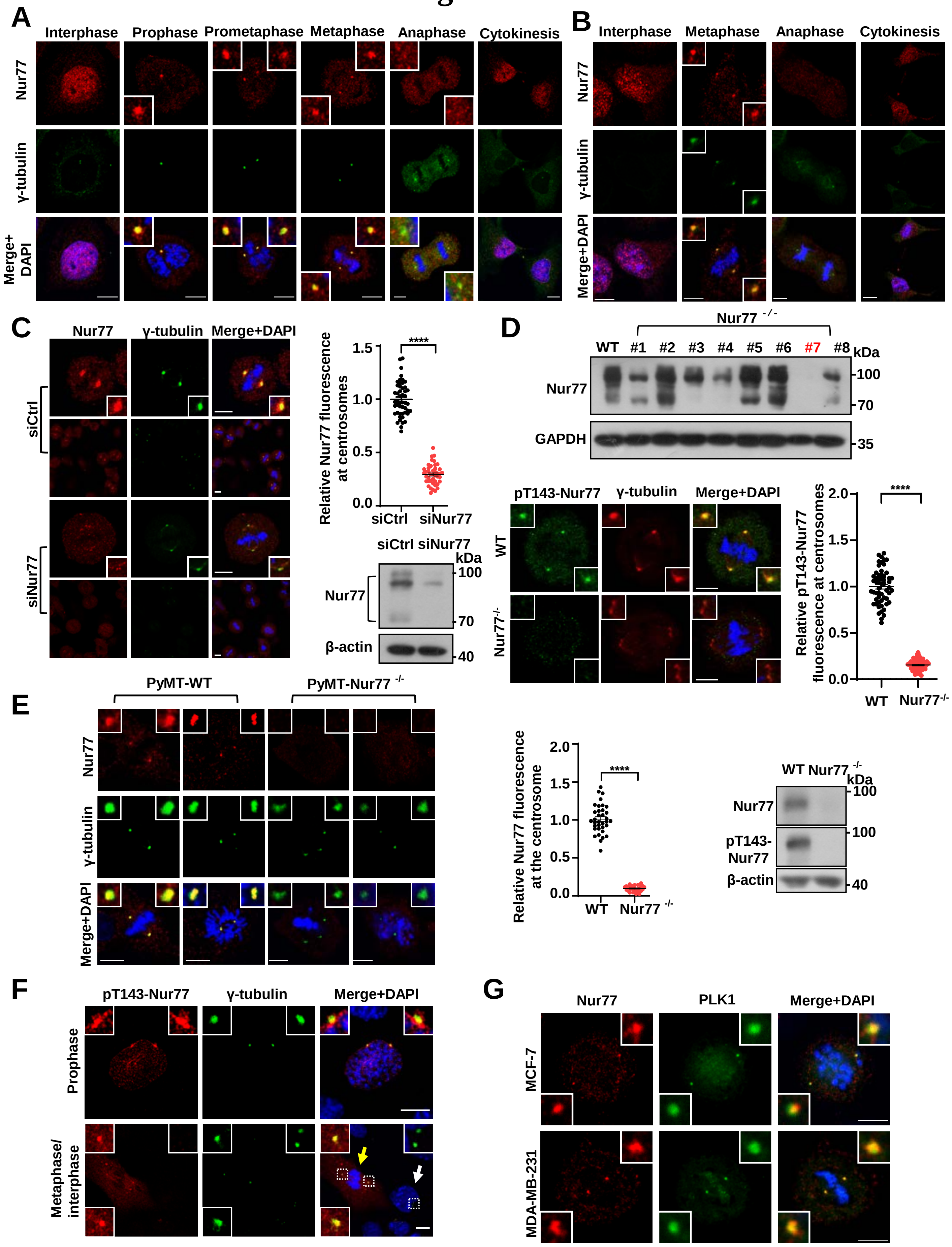
A
Interphase
Prophase
Prometaphase
Metaphase
Anaphase
Cytokinesis
Nur77
γ-tubulin
Merge+ DAPI
B
Interphase
Metaphase
Anaphase
Cytokinesis
Nur77
γ-tubulin
Merge+DAPI
C
Nur77
γ-tubulin
Merge+DAPI
siCtrl
siNur77
Relative Nur77 fluorescence at centrosomes
****
1.5
1.0
0.5
0.0
siCtrl
siNur77
siCtrl siNur77
kDa
100
70
40
Nur77
β-actin
D
Nur77 -/-
WT #1 #2 #3 #4 #5 #6 #7 #8
kDa
100
70
35
Nur77
GAPDH
pT143-Nur77
γ-tubulin
Merge+DAPI
WT
Nur77-/-
Relative pT143-Nur77 fluorescence at centrosomes
****
2.0
1.5
1.0
0.5
0.0
WT
Nur77-/-
E
PyMT-WT
PyMT-Nur77 -/-
Nur77
γ-tubulin
Merge+DAPI
Relative Nur77 fluorescence at the centrosome
****
2.0
1.5
1.0
0.5
0.0
WT
Nur77 -/-
WT Nur77 -/-
kDa
100
100
40
Nur77
pT143-Nur77
β-actin
F
pT143-Nur77
γ-tubulin
Merge+DAPI
Prophase
Metaphase/ interphase
G
Nur77
PLK1
Merge+DAPI
MCF-7
MDA-MB-231

Figure S3

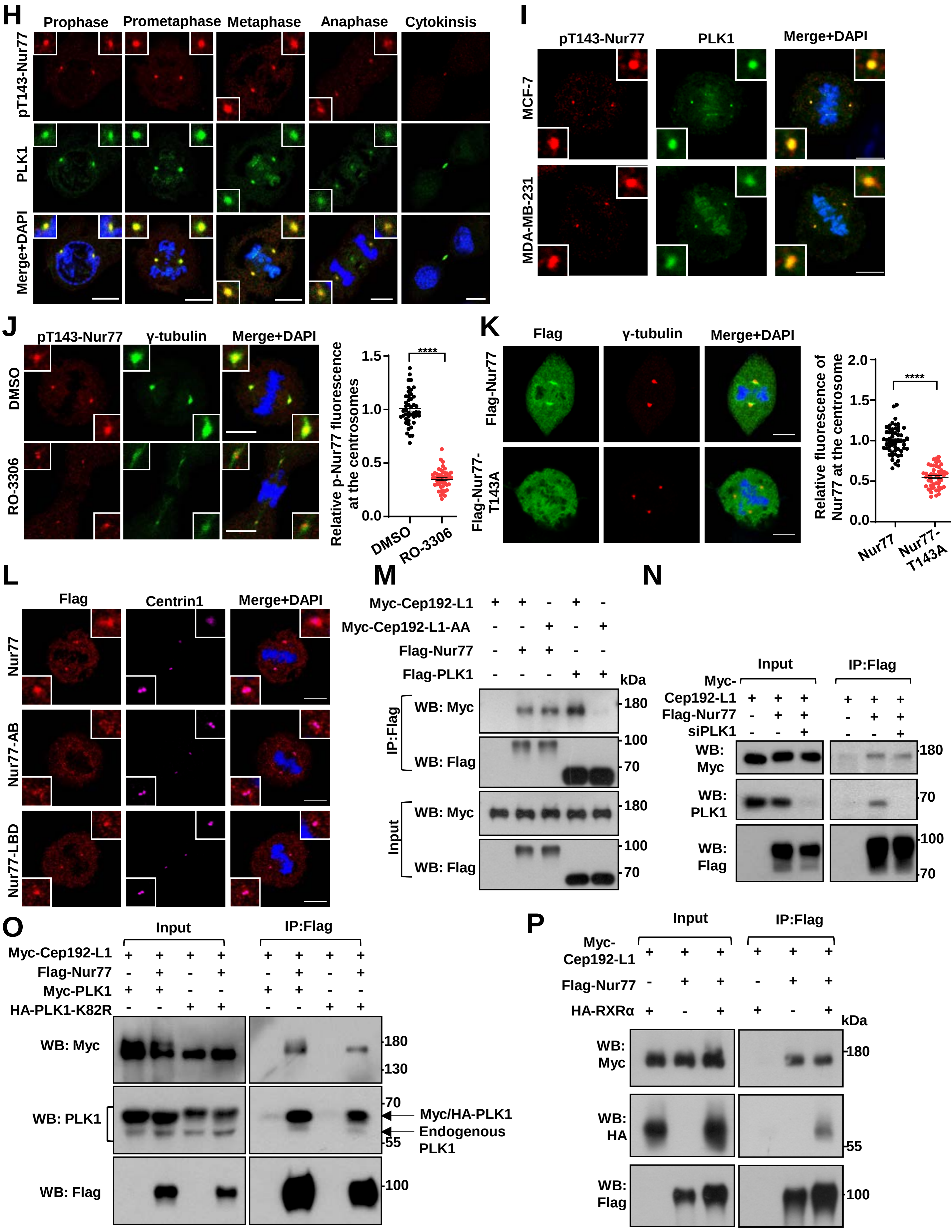
H
Prophase
Prometaphase
Metaphase
Anaphase
Cytokinsis
pT143-Nur77
PLK1
Merge+DAPI
I
pT143-Nur77
PLK1
Merge+DAPI
MCF-7
MDA-MB-231
J
pT143-Nur77
γ-tubulin
Merge+DAPI
DMSO
RO-3306
Relative p-Nur77 fluorescence at the centrosomes
1.5
1.0
0.5
0.0
****
DMSO
RO-3306
K
Flag
γ-tubulin
Merge+DAPI
Flag-Nur77
Flag-Nur77-T143A
Relative fluorescence of Nur77 at the centrosome
2.0
1.5
1.0
0.5
0.0
****
Nur77
Nur77-T143A
L
Flag
Centrin1
Merge+DAPI
Nur77
Nur77-AB
Nur77-LBD
M
Myc-Cep192-L1 + + - + -
Myc-Cep192-L1-AA - - + - +
Flag-Nur77 - + + - -
Flag-PLK1 - - - + +
kDa
IP:Flag
WB: Myc
WB: Flag
Input
WB: Myc
WB: Flag
180
100
70
180
100
70
N
Input
IP:Flag
Myc-Cep192-L1 + + + + + +
Flag-Nur77 - + + - + +
siPLK1 - - + - - +
WB: Myc
WB: PLK1
WB: Flag
180
70
100
70
O
Input
IP:Flag
Myc-Cep192-L1 + + + + + + + +
Flag-Nur77 - + - + - + - +
Myc-PLK1 + + - - + + - -
HA-PLK1-K82R - - + + - - + +
WB: Myc
WB: PLK1
WB: Flag
180
130
70
55
100
Myc/HA-PLK1
Endogenous PLK1
P
Input
IP:Flag
Myc-Cep192-L1 + + + + + +
Flag-Nur77 - + + - + +
HA-RXRα + - + + - +
kDa
WB: Myc
WB: HA
WB: Flag
180
55
100

Figure S3

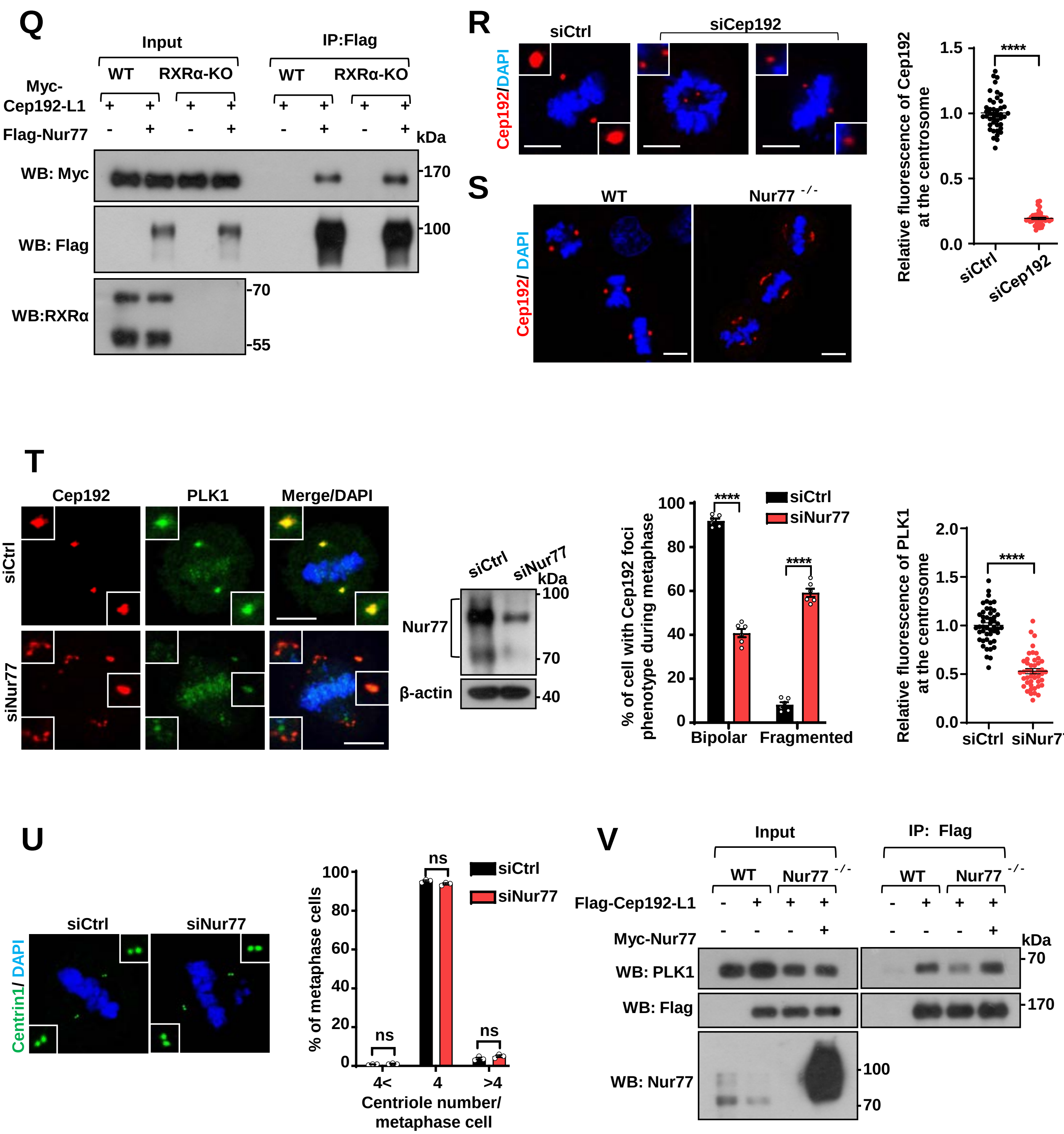

Q
Input
IP:Flag
WT
RXRα-KO
Myc-Cep192-L1
Flag-Nur77
kDa
WB: Myc
WB: Flag
WB:RXRα
170
100
70
55
R
siCtrl
siCep192
Cep192/DAPI
Relative fluorescence of Cep192 at the centrosome
****
S
WT
Nur77 -/-
Cep192/ DAPI
T
Cep192
PLK1
Merge/DAPI
siCtrl
siNur77
Nur77
β-actin
% of cell with Cep192 foci phenotype during metaphase
Bipolar
Fragmented
Relative fluorescence of PLK1 at the centrosome
U
Centrin1/ DAPI
% of metaphase cells
ns
4<
4
>4
Centriole number/ metaphase cell
V
Input
IP: Flag
WT
Nur77 -/-
Flag-Cep192-L1
Myc-Nur77
WB: PLK1
WB: Flag
WB: Nur77

Figure S3

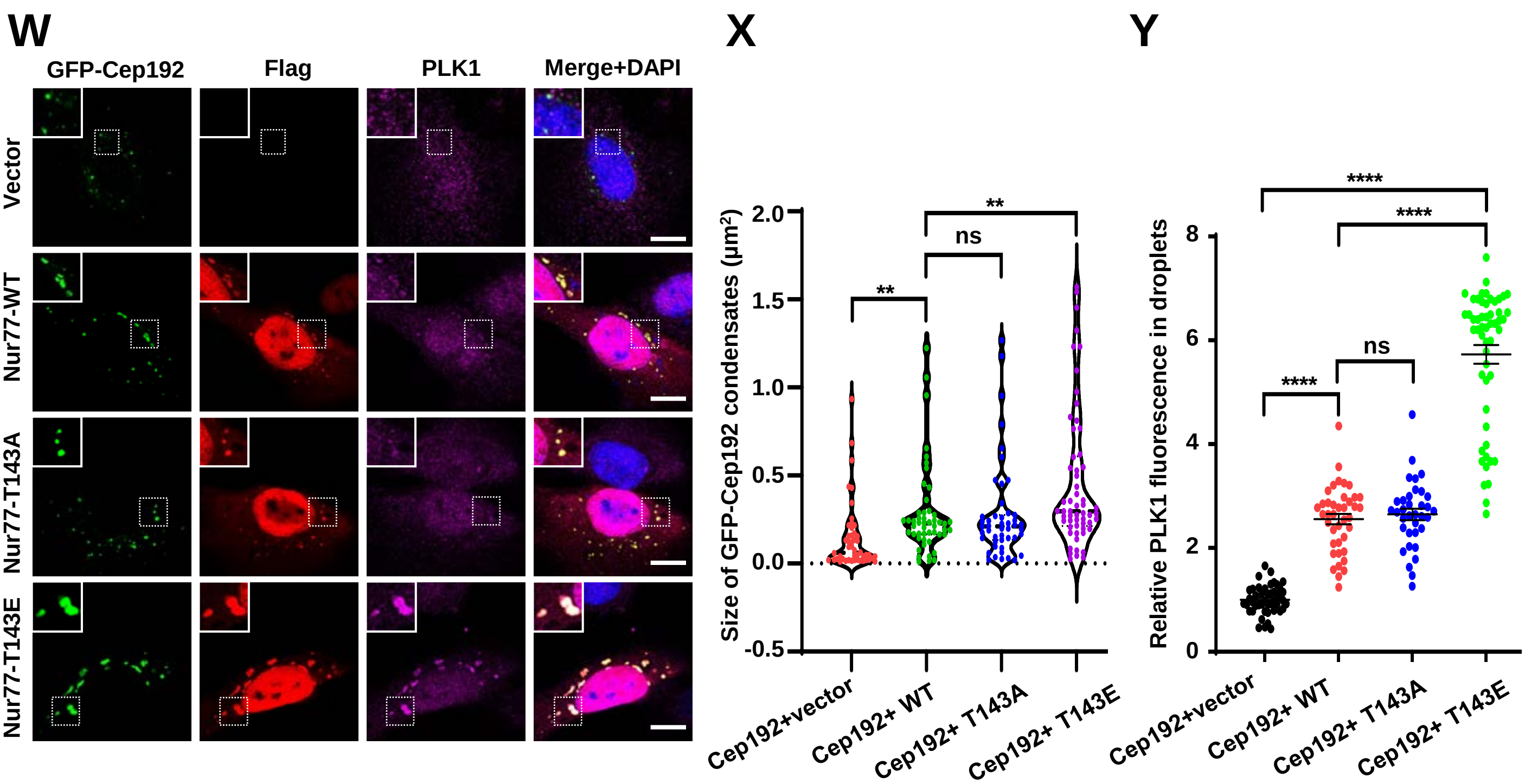

W
GFP-Cep192
Flag
PLK1
Merge+DAPI
Vector
Nur77-WT
Nur77-T143A
Nur77-T143E
X
Size of GFP-Cep192 condensates (μm²)
2.0
1.5
1.0
0.5
0.0
-0.5
**
ns
**
Cep192+vector
Cep192+ WT
Cep192+ T143A
Cep192+ T143E
Y
Relative PLK1 fluorescence in droplets
8
6
4
2
0
****
****
****
ns
Cep192+vector
Cep192+ WT
Cep192+ T143A
Cep192+ T143E

Figure S3. Localization of p-Nur77 at the centrosome during mitosis.

(A, B) Subcellular localization of Nur77 in HeLa cells during interphase and mitosis was examined by immunostaining. Nur77 (red), γ-tubulin (green), and DAPI (blue). Two anti-Nur77 antibodies from Santa cruz (**A**) and Proteintech (**B**) were used respectively. Scale bar, 10 μm.

(C) Specific staining of Nur77 protein at the centrosome was examined by immunostaining of HeLa cells transfected with control or Nur77 siRNA. Nur77 (red), γ-tubulin (green), and DAPI (blue). Scale bar, 10 μm. Quantitation of the immunofluorescence intensity of Nur77 at the centrosome (n= 49, and 43 cells, respectively; ****$p < 0.0001$; two-tailed unpaired t-test). The efficiency of Nur77 siRNA transfection was shown by WB.

(D) Nur77 was knocked out in HeLa cells by CRISPR-Cas9 editing and the stable cell clones were verified by WB. Parental HeLa cells and Nur77-depleted HeLa cells synchronized at mitosis by TT block were analyzed by immunofluorescence. pT143-Nur77 (green), γ-tubulin (red), and DAPI (blue). Relative fluorescence intensity of pT143-Nur77 at the centrosome during metaphase were calculated (n=50 cells, respectively; ****$p < 0.0001$; two-tailed unpaired t-test). Scale bar, 10 μm.

(E) Centrosomal localization of Nur77 in primary mouse breast cancer cells from the mammary specific polyomavirus middle T antigen mouse model (MMTV-PyMT) were analyzed by immunostaining. Nur77 (red), γ-tubulin (green), and DAPI (blue). Scale bar, 10 μm. Quantitation of the immunofluorescence intensity of Nur77 at the centrosome (n= 35, and 30 cells, respectively; ****$p < 0.0001$; two-tailed unpaired t-test). The deletion of Nur77 in the primary mouse breast cancer cells was shown by WB.

(F) Centrosomal localization of pT143-Nur77 in mouse primary breast cancer cells during mitosis was examined by immunostaining. pT143-Nur77(red), γ-tubulin(green), and DAPI(blue). Scale bar, 10 μm. The yellow arrow indicates mitotic cell and the white arrow indicates interphase cell.

(G-I) Colocalization of Nur77/p-Nur77 and PLK1 at the centrosome during mitosis revealed by immunostaining in MCF-7 cells (**G, I**), MDA-MB-231 cells (**G, I**) and HeLa cells (**H**). Nur77/p-Nur77 (red), PLK1 (green), and DAPI (blue). Scale bar, 10 μm.

(J) Centrosomal localization of pT143-Nur77 was analyzed by immunostaining after mitotic HeLa cells were treated with 10 μM RO-3306 for 15 min. pT143-Nur77 (red), γ-tubulin (green), and DAPI (blue). Scale bar, 10 μm. The relative immunofluorescence intensity of Nur77 at the centrosome was calculated (n= 45, and 47 cells, respectively; ****$p < 0.0001$; two-tailed unpaired t-test).
(K) HeLa cells transfected with Flag-Nur77 or Flag-Nur77-T143A were synchronized at mitosis by TT block and analyzed by immunostaining. Flag (green), γ-tubulin (red), and DAPI (blue). Relative fluorescence intensity of Flag-Nur77 and Flag-Nur77-T143A at the centrosome during metaphase were analyzed (n= 56, and 48 cells, respectively; ****$p < 0.0001$; two-tailed unpaired t-test).
(L) HeLa cells transfected with the indicated expression vectors (Nur77 and its mutants) were synchronized at mitosis by TT block and analyzed by immunostaining. Flag (red), centrin1 (violet), and DAPI (blue). Scale bar, 10 μm.
(M) HeLa cells transfected with or without Myc-Cep192-L1, Myc-Cep192-L1-AA(T44A/S995A), Flag-Nur77 and Flag-PLK1 were synchronized at mitosis by TT block and analyzed by CoIP assay.
(N) Knock down of PLK1 have no effect on Nur77 interaction with Cep192. HeLa cells were transfected with control siRNA or PLK1 siRNA, followed by transfection with Myc-Cep192-L1 and Flag-Nur77. Cells were then synchronized at mitosis using a double thymidine block and subjected to Co-IP analysis.
(**O**) Kinase activity of PLK1 has no apparent effect on Nur77 interaction with Cep192. HeLa cells transfected with the indicated plasmids were synchronized at mitosis using a double thymidine block and then analyzed by Co-IP.
(P) Nur77 interaction with Cep192 is RXRα-independent. HeLa cells were transfected with Myc-Cep192-L1, Flag-Nur77 and HA-RXRα and synchronized at mitosis by TT block and analyzed by CoIP assay.
(Q) RXRα is not essential for Nur77 interaction with Cep192. HeLa cells (wild type, WT) and RXRα deficiency HeLa cells (RXRα-KO) were transfected with Flag-Nur77 and Myc-Cep192-L1 and synchronized at mitosis by TT block and analyzed by CoIP assay.
(R) The efficiency of Cep192 siRNA in mitotic HeLa cells was examined by immunostaining. Cep192 (red) and DAPI (blue). Relative fluorescence intensity of Cep192 at the centrosome during mitosis was calculated (n= 43, and 40 cells, respectively; ****$p < 0.0001$; two-tailed unpaired t-test). Scale bar, 10 μm.

(S) Centrosomal localization of Cep192 is abnormal upon Nur77 depletion. HeLa cells and Nur77 depletion HeLa cells were synchronized at mitosis by TT treatment and analyzed by immunostaining. Cep192 (red) and DAPI (blue). Scale bar, 10 µm.

(T) Centrosomal localization of Cep192 is abnormal upon Nur77 knock down by siRNA. HeLa cells transfected with control siRNA or Nur77 siRNA were synchronized at mitosis by TT block and analyzed by immunostaining. Cep192 (red), PLK1 (green) and DAPI (blue). Scale bar, 10 µm. The efficiency of Nur77 siRNA transfection was shown by WB. Percentage of fragmented Cep192 foci and relative fluorescence intensity of PLK1 at the centrosome during metaphase were calculated respectively (left graph: n=200 cells across six experiments; right graph, n=48, and 45 cells, respectively; ****$p < 0.0001$; two-tailed unpaired *t*-test)

(U) Fragmented Cep192 foci induced by Nur77 depletion was not due to centriole overduplication and disengagement. Mitotic HeLa cells transfected with control siRNA or Nur77 siRNA were analyzed by immunostaining. Centrin1 (green) and DAPI (blue). Scale bar, 10 µm. Centriole number per metaphase cell was calculated (n=150 cells across three experiments; ns $p= 0.2302$, ns $p= 0.0739$, ns $p= 0.2341$; two-tailed unpaired *t*-test).

(V) Interaction of transfected Flag-Cep192-L1 with endogenous PLK1 in mitotic HeLa cells (WT) or Nur77 depletion HeLa cells ($Nur77^{-/-}$) with or without reexpression of Nur77 were analyzed by CoIP assay.

(W-Y) Endogenous PLK1 is recruited to the Flag-Nur77-T143E/GFP-Cep192 droplets. HeLa cells transfected with GFP-Cep192 and Flag-Nur77 or its mutants were analyzed by immunostaining. GFP-Cep192 (green), Flag (red), PLK1 (purple) and DAPI (blue). The size of GFP-Cep192 droplets was calculated (**X**) (independent cells measured from left to right: 48, 50, 47, 58; **$p= 0.0027$, ns $p=0.9947$, **$p= 0.0065$; two-tailed unpaired t-test). Relative fluorescence intensity of PLK1 at GFP-Cep192 droplets (**Y**) was calculated (independent cells measured from left to right: 46, 40, 35, 53; ns $p=0.5479$, ****$p< 0.0001$; two-tailed unpaired *t*-test). Scale bar, 10 µm.

All quantitative data in this figure are presented as mean values ±SEM.

Figure S4

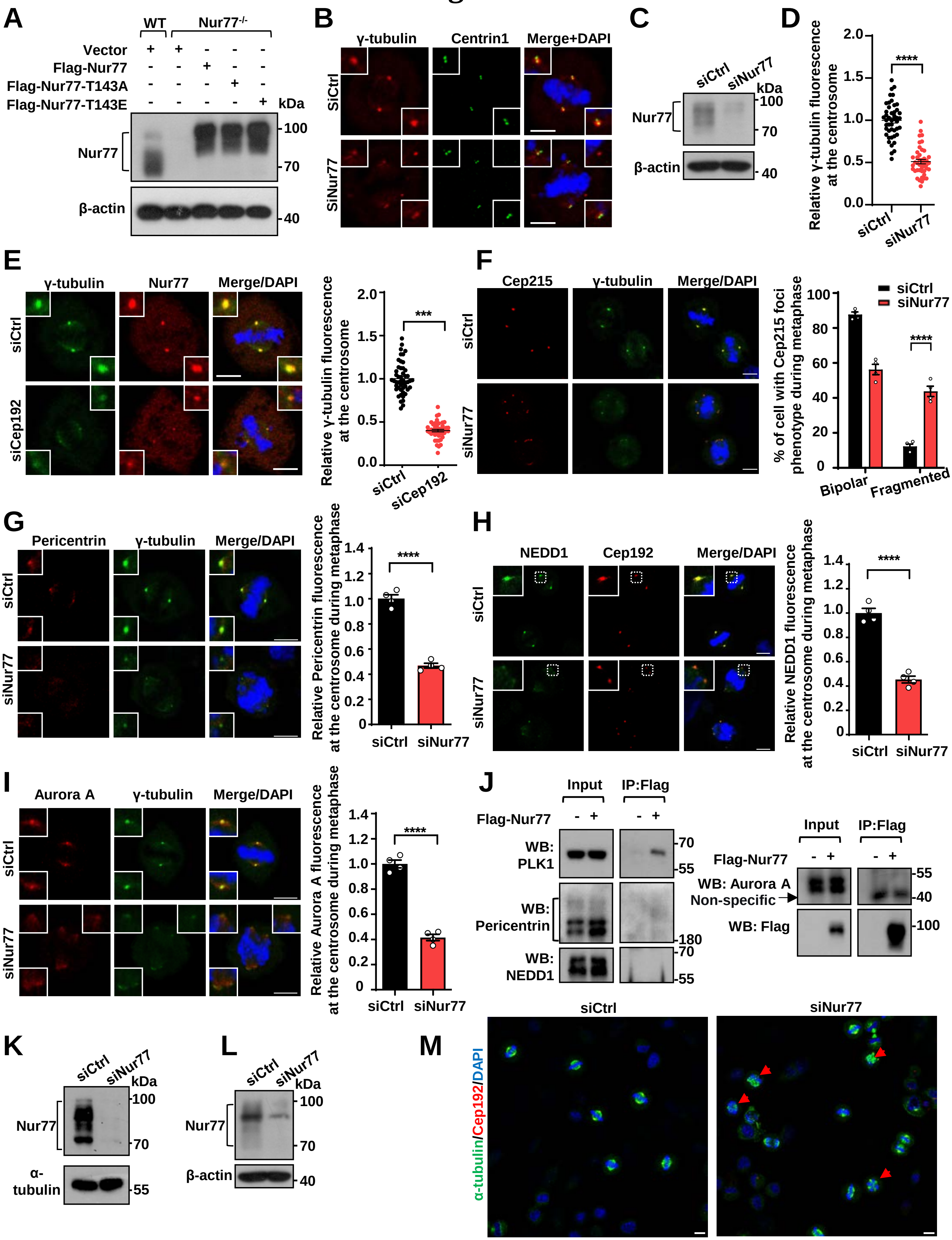

A
WT
Nur77-/-
Vector
Flag-Nur77
Flag-Nur77-T143A
Flag-Nur77-T143E
kDa
Nur77
β-actin
100
70
40
B
γ-tubulin
Centrin1
Merge+DAPI
SiCtrl
SiNur77
C
siCtrl
siNur77
kDa
Nur77
β-actin
100
70
40
D
Relative γ-tubulin fluorescence at the centrosome
****
siCtrl
siNur77
E
γ-tubulin
Nur77
Merge/DAPI
siCtrl
siCep192
Relative γ-tubulin fluorescence at the centrosome
***
siCtrl
siCep192
F
Cep215
γ-tubulin
Merge/DAPI
siCtrl
siNur77
% of cell with Cep215 foci phenotype during metaphase
****
Bipolar
Fragmented
G
Pericentrin
γ-tubulin
Merge/DAPI
siCtrl
siNur77
Relative Pericentrin fluorescence at the centrosome during metaphase
****
H
NEDD1
Cep192
Merge/DAPI
siCtrl
siNur77
Relative NEDD1 fluorescence at the centrosome during metaphase
****
I
Aurora A
γ-tubulin
Merge/DAPI
siCtrl
siNur77
Relative Aurora A fluorescence at the centrosome during metaphase
****
J
Input
IP:Flag
Flag-Nur77
WB: PLK1
WB: Pericentrin
WB: NEDD1
70
55
180
WB: Aurora A
Non-specific
WB: Flag
40
100
K
siCtrl
siNur77
kDa
Nur77
α-tubulin
100
70
55
L
siCtrl
siNur77
kDa
Nur77
β-actin
100
70
40
M
siCtrl
siNur77
α-tubulin/Cep192/DAPI

Figure S4

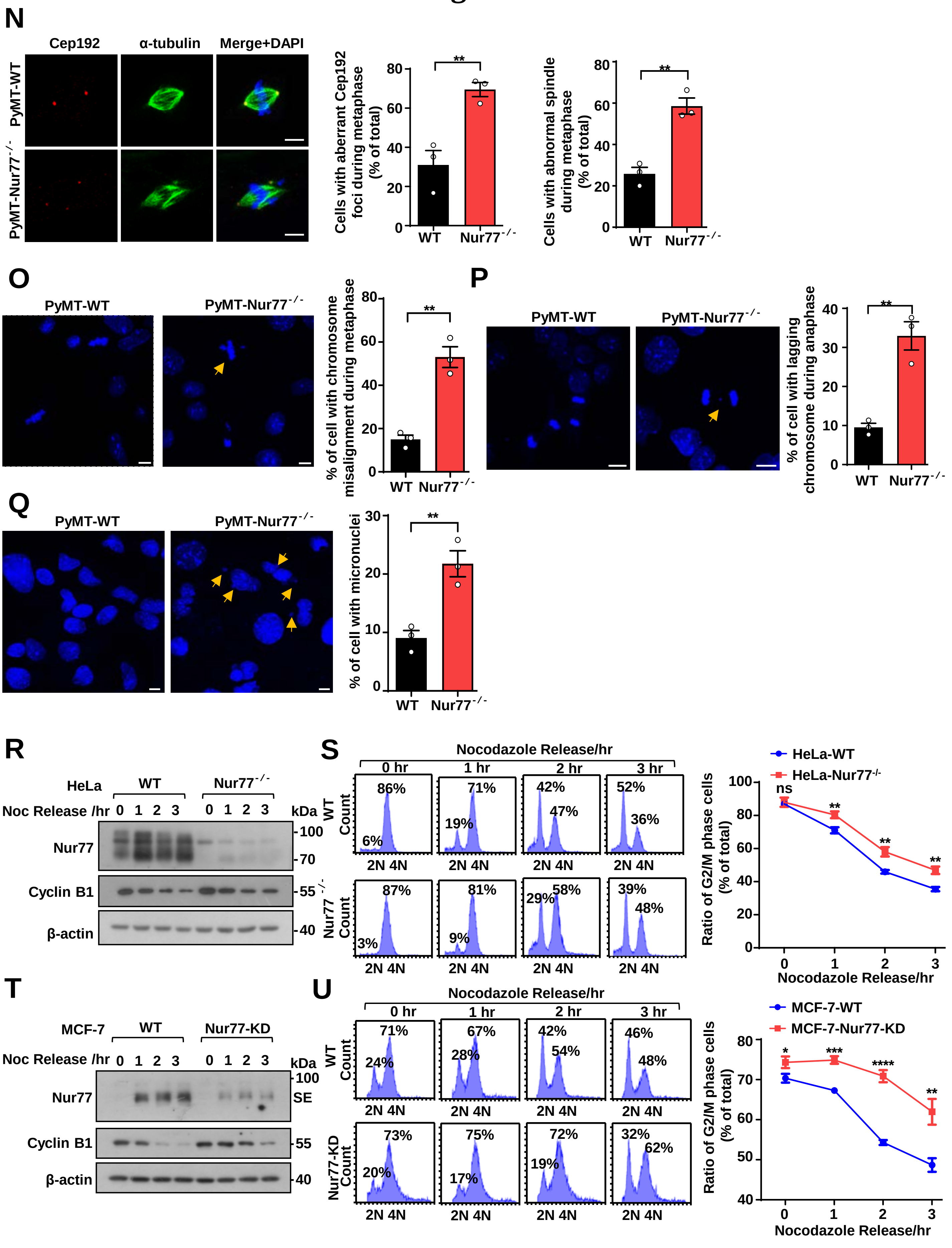

N
Cep192
α-tubulin
Merge+DAPI
PyMT-WT
PyMT-Nur77-/-
Cells with aberrant Cep192 foci during metaphase (% of total)
Cells with abnormal spindle during metaphase (% of total)
WT
Nur77-/-
O
PyMT-WT
PyMT-Nur77-/-
% of cell with chromosome misalignment during metaphase
P
PyMT-WT
PyMT-Nur77-/-
% of cell with lagging chromosome during anaphase
Q
PyMT-WT
PyMT-Nur77-/-
% of cell with micronuclei
R
HeLa
WT
Nur77-/-
Noc Release /hr
kDa
Nur77
Cyclin B1
β-actin
S
Nocodazole Release/hr
0 hr
1 hr
2 hr
3 hr
WT Count
Nur77-/- Count
2N 4N
86%
6%
71%
19%
42%
47%
52%
36%
87%
3%
81%
9%
29%
58%
39%
48%
HeLa-WT
HeLa-Nur77-/-
Ratio of G2/M phase cells (% of total)
Nocodazole Release/hr
T
MCF-7
WT
Nur77-KD
Noc Release /hr
kDa
Nur77
SE
Cyclin B1
β-actin
U
Nocodazole Release/hr
0 hr
1 hr
2 hr
3 hr
WT Count
Nur77-KD Count
2N 4N
71%
24%
67%
28%
42%
54%
46%
48%
73%
20%
75%
17%
72%
19%
32%
62%
MCF-7-WT
MCF-7-Nur77-KD
Ratio of G2/M phase cells (% of total)
Nocodazole Release/hr

Figure S4

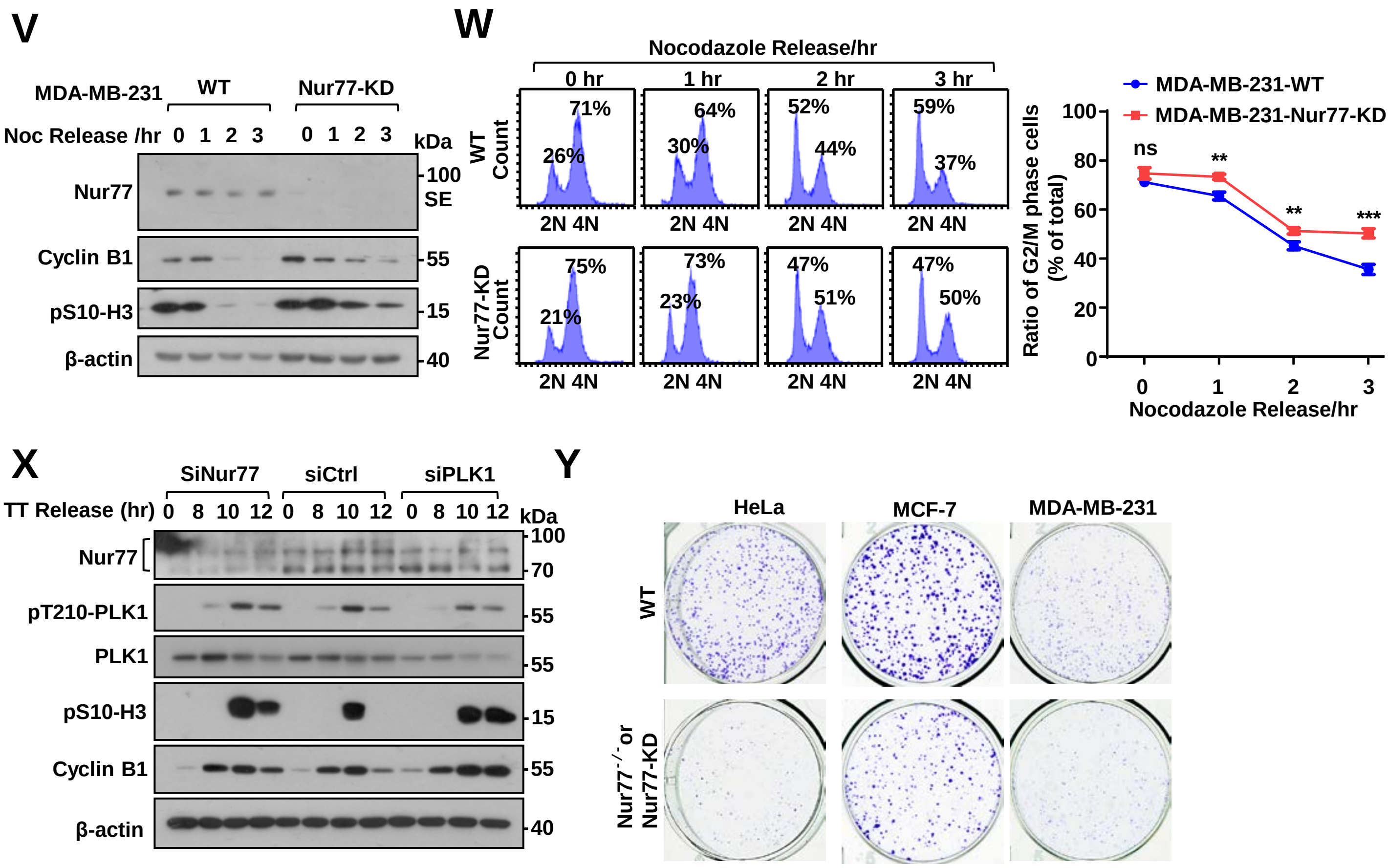
V
MDA-MB-231
WT
Nur77-KD
Noc Release /hr 0 1 2 3 0 1 2 3
kDa
Nur77
100
SE
Cyclin B1
55
pS10-H3
15
β-actin
40
W
Nocodazole Release/hr
0 hr
1 hr
2 hr
3 hr
WT Count
71%
26%
64%
30%
52%
44%
59%
37%
2N 4N
Nur77-KD Count
75%
21%
73%
23%
47%
51%
47%
50%
MDA-MB-231-WT
MDA-MB-231-Nur77-KD
Ratio of G2/M phase cells (% of total)
ns
**
**
***
Nocodazole Release/hr
X
SiNur77
siCtrl
siPLK1
TT Release (hr) 0 8 10 12 0 8 10 12 0 8 10 12
kDa
Nur77
100
70
pT210-PLK1
55
PLK1
55
pS10-H3
15
Cyclin B1
55
β-actin
40
Y
HeLa
MCF-7
MDA-MB-231
WT
Nur77-/- or Nur77-KD

Figure S4

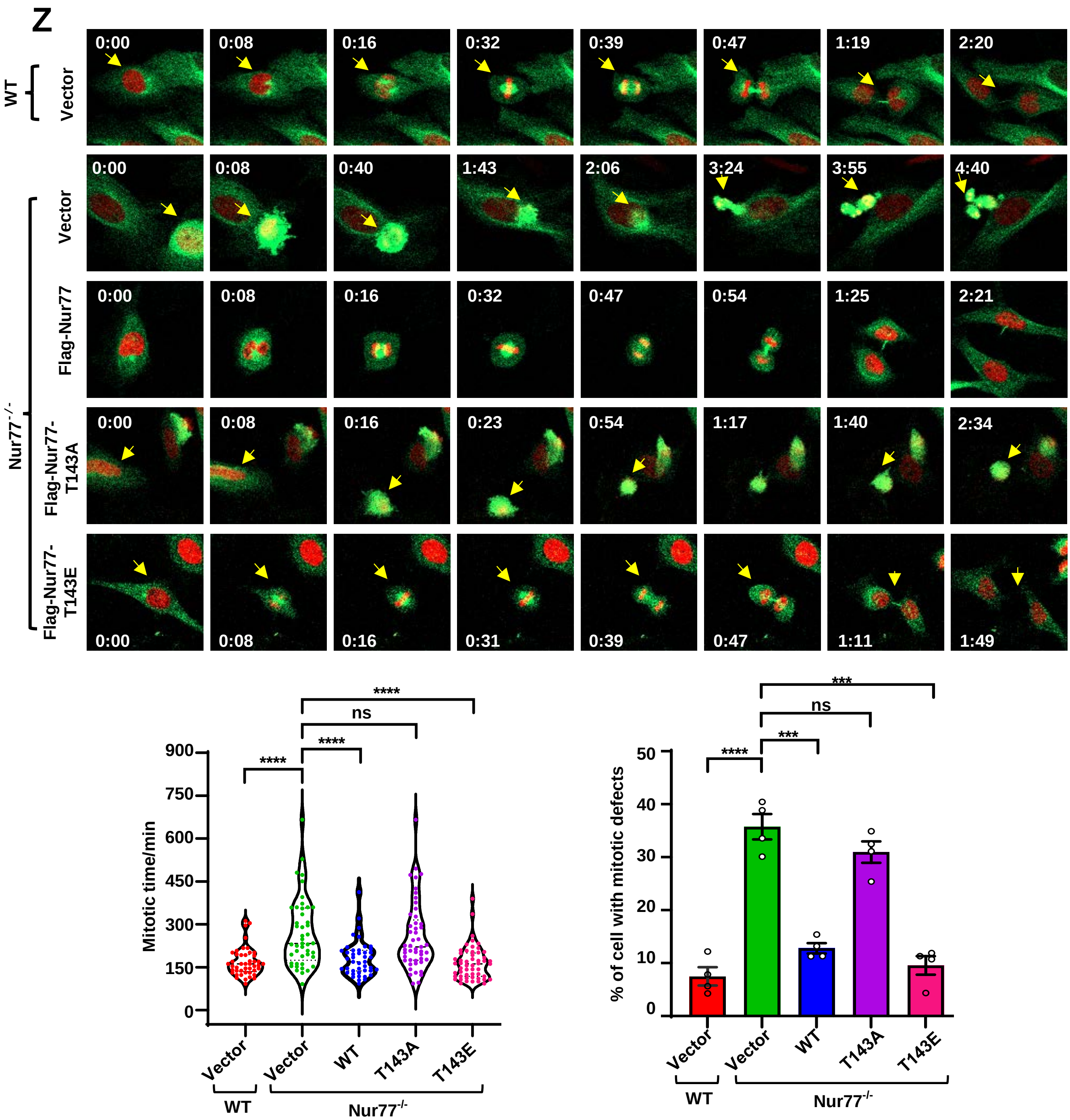

Z
WT
Vector
Nur77-/-
Vector
Flag-Nur77
Flag-Nur77-T143A
Flag-Nur77-T143E
0:00
0:08
0:16
0:32
0:39
0:47
1:19
2:20
0:40
1:43
2:06
3:24
3:55
4:40
0:47
0:54
1:25
2:21
0:23
0:54
1:17
1:40
2:34
0:31
0:39
1:11
1:49
Mitotic time/min
900
750
600
450
300
150
0
****
ns
****
****
Vector
Vector
WT
T143A
T143E
WT
Nur77-/-
% of cell with mitotic defects
50
40
30
20
10
0
***
ns
***
****

Figure S4. p-Nur77 regulate centrosome maturation and mitotic progression.

(A) The expression of transfected Flag-Nur77 or mutants in Nur77 depletion HeLa cells was examined by WB, *related to Fig. 4A.*

(B-D) HeLa cells transfected with control siRNA and Nur77 siRNA were synchronized at mitosis by TT treatment and stained by γ-tubulin (red), centrin1 (green), and DAPI (blue). Scale bar, 10 μm. The efficiency of Nur77 siRNA was examined by WB (**C**).Relative fluorescence intensity of γ-tubulin at the centrosome was calculated (**D**) (n= 46, and 44 cells, respectively; ****p< 0.0001; two-tailed unpaired t-test).

(E) HeLa cells transfected with control siRNA and Cep192 siRNA were synchronized at mitosis by TT treatment and stained by Nur77(red), γ-tubulin (green) and DAPI (blue). Scale bar, 10 μm. Relative fluorescence intensity of γ-tubulin at the centrosome was calculated (n= 49, and 44 cells, respectively; ****p< 0.0001; two-tailed unpaired t-test).

(F-I) The effect of Nur77 on the centrosomal localization of Cep215, pericentrin, NEDD1 and Aurora A. HeLa cells transfected with control siRNA or Nur77 siRNA were synchronized at mitosis using a double thymidine block and then analyzed by immunofluorescence (IF) with the indicated antibodies. Percentage of cells with fragmented Cep215 was calculated (n=600 cells across three experiments; ****p < 0.0001; two-tailed unpaired t-test). Relative fluorescence intensity of pericentrin, NEDD1 and Aurora A at the centrosome were calculated respectively (n=30 cells per group; ****p < 0.0001; two-tailed unpaired t-test). Scale bar, 10 μm.

(J) HeLa transfected with Flag-Nur77 were synchronized at mitosis using a double thymidine block and then analyzed by Co-IP.

(K) The efficiency of Nur77 siRNA was examined by WB, *related to Fig. 4C.*

(L, M) HeLa cells transfected with control and Nur77 siRNA were synchronized at mitosis by TT treatment and stained by Cep192 (red), α-tubulin (green) and DAPI (blue). Scale bar, 10 μm. The efficiency of Nur77 siRNA was examined by WB (**L**). The red arrows indicate aberrant mitotic spindle (**M**).

(N) The primary mouse breast tumor cells (wild type and Nur77 deletion) during mitosis were stained by Cep192 (red), α-tubulin (green), and DAPI (blue). Scale bar, 10 μm. Percentage of aberrant Cep192 foci and abnormal mitotic spindle were calculated respectively (left graph: n=100 cells across three experiments, **p= 0.0093 ; right graph: n=100 cells across three experiments, **p= 0.0028; two-tailed unpaired *t*-test).

(O-Q) The primary mouse breast tumor cells (wild type and Nur77 deletion) were stained by DAPI (blue). Percentage of cells with chromosome misalignment (**O**), lagging chromosome (**P**), and micronuclei (**Q**) were calculated respectively (n=100 cells across three experiments; for chromosome misalignment analysis, **p= 0.0075; for lagging chromosome analysis, **p= 0.0034; for micronuclei analysis, **p= 0.0077; two-tailed unpaired t-test).

(R-W) HeLa cells, MCF-7 cells and MDA-MB-231 (wild type and Nur77 depletion) were synchronized at mitosis by nocodazole treatment for 12 hr, released for the indicated time, and analyzed by WB and FACS (n=3 biological replicates; for HeLa, ns p= 0.5935, **p= 0.0041, **p= 0.0023, **p= 0.00416; for MCF-7, *p= 0.0194, ***p= 0.0003, ****p< 0.0001, **p= 0.0032; for MDA-MB-231, ns p= 0.1079, **p= 0.0021, **p= 0.0067, ***p= 0.0008; two-tailed unpaired t-test).

(X) HeLa cells transfected with control siRNA or Nur77 siRNA or PLK1 siRNA were released from TT block for the indicated time and analyzed by WB.

(Y) HeLa cells, MCF-7 cells and MDA-MB-231 (WT and Nur77 depletion) were maintained in normal growth medium for 10 days for colony formation, and cells colonies were fixed and stained with crystal violet.

(Z) Wild type and Nur77 depletion HeLa cells expressing EGFP-α-tubulin and mCherry-H2B were transfected with or without the indicated Nur77 expression vector and synchronized at mitosis by TT treatment, then imaged by fluorescence time-lapse microscopy. The numbers on images indicate the time. The mitotic time of cells and percentage of cells with mitotic defects were calculated respectively (left graph: independent cells measured from left to right: 45, 45, 43, 49, 53; ****p< 0.0001, ns p=0.6642; right graph: n=100 cells across four experiments; ****p< 0.0001, ns p=0.1761, ***p= 0.0001, ***p= 0.0001; two-tailed unpaired t-test).

All quantitative data in this figure are presented as mean values ±SEM.

Figure S5

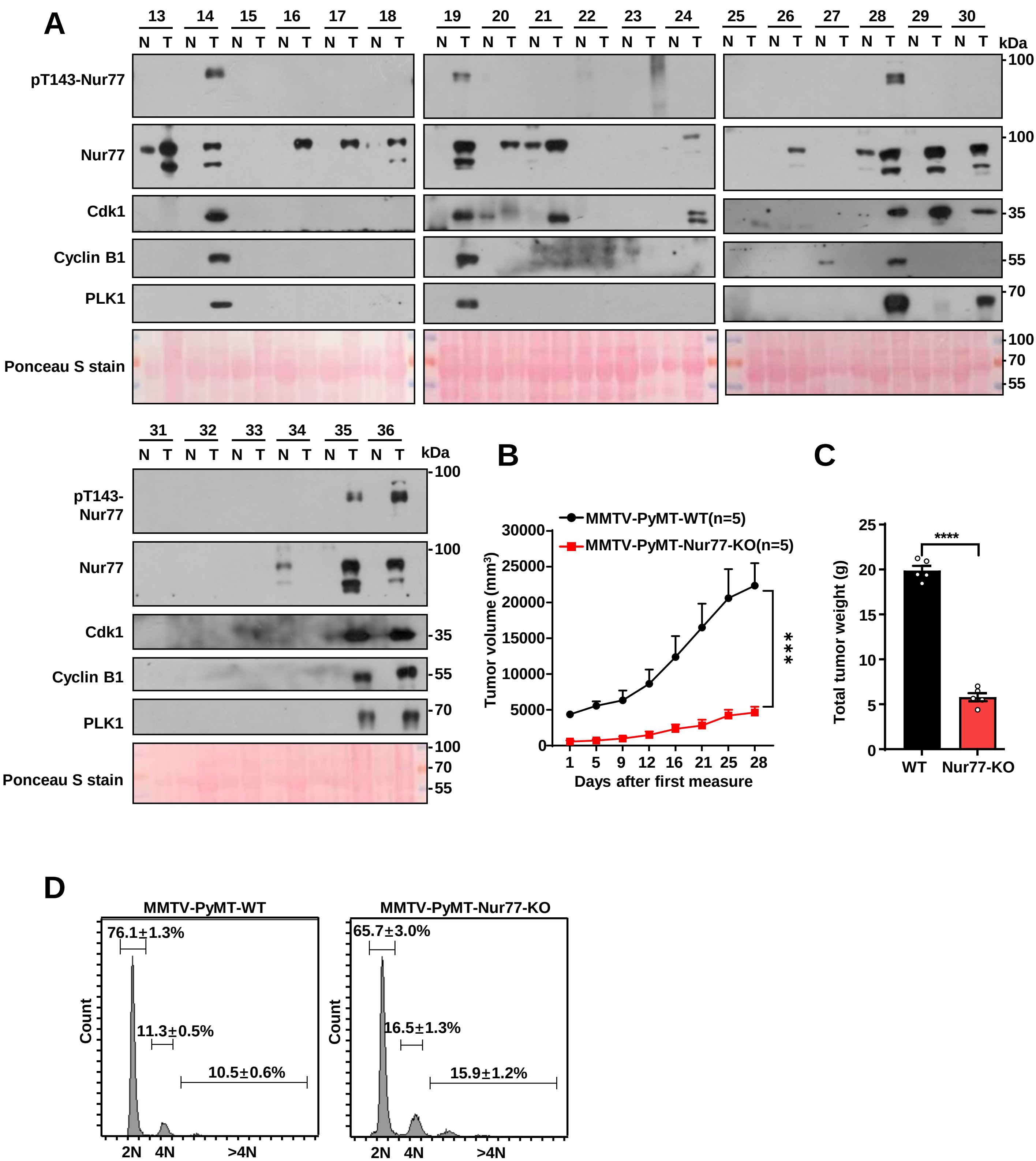
A
13 14 15 16 17 18
19 20 21 22 23 24
25 26 27 28 29 30
N T N T N T N T N T N T
kDa
pT143-Nur77
Nur77
Cdk1
Cyclin B1
PLK1
Ponceau S stain
100
100
35
55
70
100
70
55
31 32 33 34 35 36
pT143-Nur77
B
MMTV-PyMT-WT(n=5)
MMTV-PyMT-Nur77-KO(n=5)
Tumor volume (mm³)
30000
25000
20000
15000
10000
5000
0
1 5 9 12 16 21 25 28
Days after first measure
***
C
Total tumor weight (g)
25
20
15
10
5
0
****
WT
Nur77-KO
D
MMTV-PyMT-WT
76.1±1.3%
11.3±0.5%
10.5±0.6%
Count
2N 4N >4N
MMTV-PyMT-Nur77-KO
65.7±3.0%
16.5±1.3%
15.9±1.2%

Figure S5

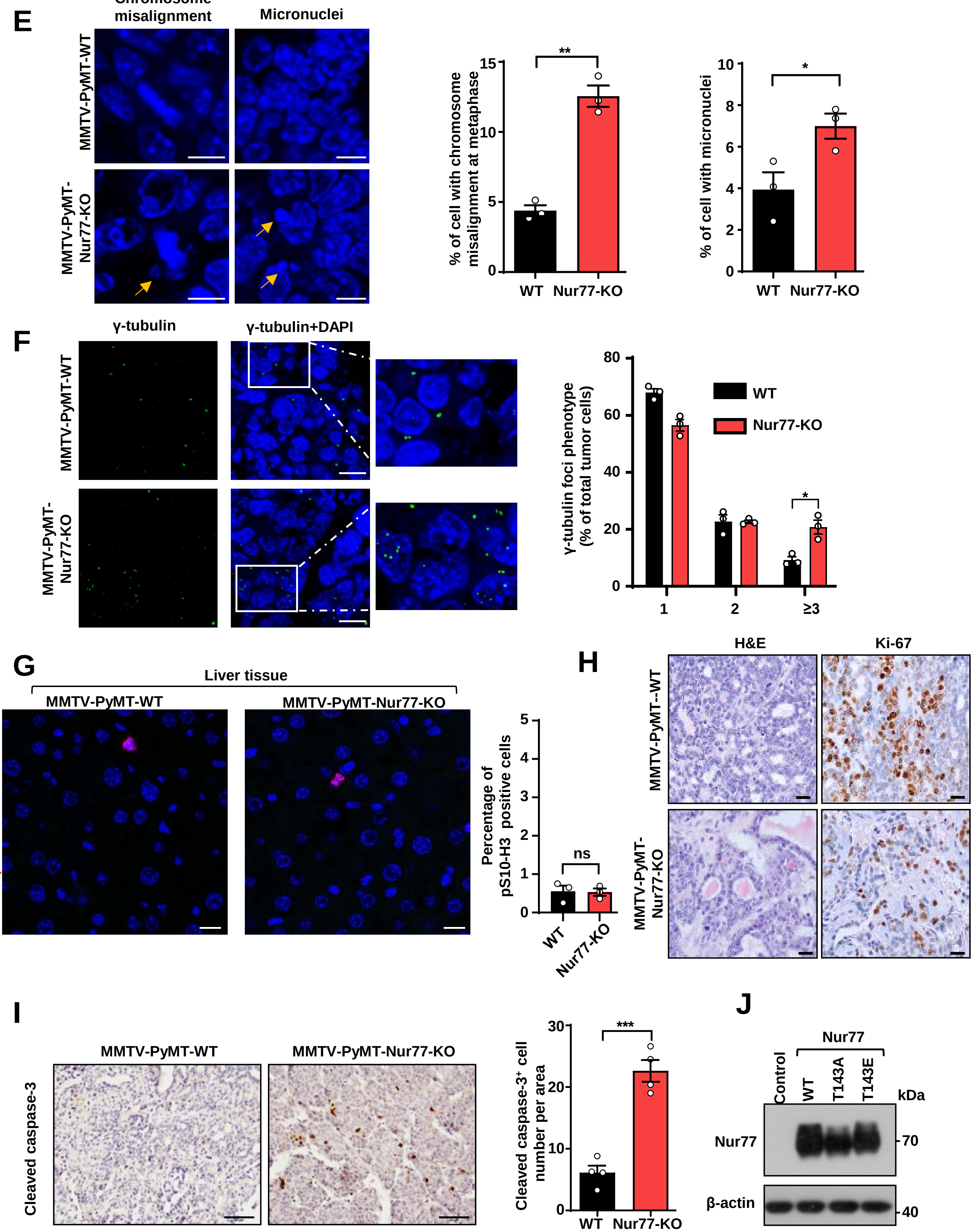

E
Chromosome misalignment
Micronuclei
MMTV-PyMT-WT
MMTV-PyMT-Nur77-KO
% of cell with chromosome misalignment at metaphase
**
15
10
5
0
WT
Nur77-KO
% of cell with micronuclei
*
10
8
6
4
2
0
WT
Nur77-KO
F
γ-tubulin
γ-tubulin+DAPI
MMTV-PyMT-WT
MMTV-PyMT-Nur77-KO
γ-tubulin foci phenotype (% of total tumor cells)
80
60
40
20
0
WT
Nur77-KO
*
1
2
≥3
G
Liver tissue
MMTV-PyMT-WT
MMTV-PyMT-Nur77-KO
pS10-H3/DAPI
Percentage of pS10-H3 positive cells
5
4
3
2
1
0
ns
WT
Nur77-KO
H
H&E
Ki-67
MMTV-PyMT--WT
MMTV-PyMT-Nur77-KO
I
MMTV-PyMT-WT
MMTV-PyMT-Nur77-KO
Cleaved caspase-3
Cleaved caspase-3+ cell number per area
***
30
20
10
0
WT
Nur77-KO
J
Nur77
Control
WT
T143A
T143E
kDa
Nur77
70
β-actin
40

Figure S5. p-Nur77 is abnormal in tumor and promotes tumor growth in vivo.

(A) Correlation of pT143-Nur77 expression with the activation of Cdk1 and PLK1 in clinical breast tumor sample from patients. Human breast cancer tissues (T) and their corresponding tumor adjacent normal tissues(N) were analyzed by WB.

(B, C) Nur77 deletion in MMTV-PyMT mice inhibits the growth of breast tumor. The tumor volume were monitored and recorded once in three or five days since mice were 12 weeks old **(B).** Tumor excised from wild type and Nur77 knockout MMTV-PyMT mice were weighed **(C)** (n=5 per group; ****p< 0.0001; two-tailed unpaired *t*-test), *related to Fig. 5D.*

(D) Breast tumor specimens excised from MMTV-PyMT-WT and MMTV-PyMT-WT-Nur77-KO mice were digested by collagenase, then isolated tumor cells were collected and analyzed by FACS. Data are means ±SD (three independent samples per group).

(E) Tumor specimens from wild type and Nur77 knockout MMTV-PyMT mice were analyzed by stained with DAPI (blue). Scale bar, 5 μm. Percentage of cells with chromosome misalignment and micronuclei were calculated respectively (left graph, n=100 cells across three experiments; right graph, n=300 cells across three experiments; **p= 0.0025, *p= 0.0468; two-tailed unpaired *t*-test).

(F) Tumor specimens from wild type and Nur77 knockout MMTV-PyMT mice were analyzed by stained with γ-tubulin (green) and DAPI (blue). Scale bar, 10 μm. Percentage of cells with multicentrosomes was calculated (n=300 cells across three experiments; *p= 0.0128; two-tailed unpaired *t*-test).

(G) Nur77 deletion doesn't induce mitotic arrest in normal liver tissue from MMTV-PyMT mice. Normal liver tissue from wild type and Nur77 knockout MMTV-PyMT mice were stained by pS10-H3 (red) and DAPI (blue). Scale bar, 10 μm. Percentage of pS10-H3 positive cells was calculated (ten random fields per sample were analyzed; ns p= 0.9169; two-tailed unpaired t-test).

(H) H&E and Ki-67 staining of breast tumor tissues from MMTV-PyMT-WT and MMTV-PyMT-WT-Nur77-KO mice were shown. Scale bar, 20μm.

(I) Tumor specimens from wild type and Nur77 knockout MMTV-PyMT mice were analyzed by immunohistochemical staining. Scale bar, 50 μm. Percentage of positive cleaved caspase-3 cells was calculated (ten random fields per sample were analyzed; ***p= 0.0005; two-tailed unpaired t-test).

(J) MDA-MB-231 cells stably expressing Nur77 or its mutants were examined by WB. All quantitative data in this figure are presented as mean values ±SEM.

Figure S6

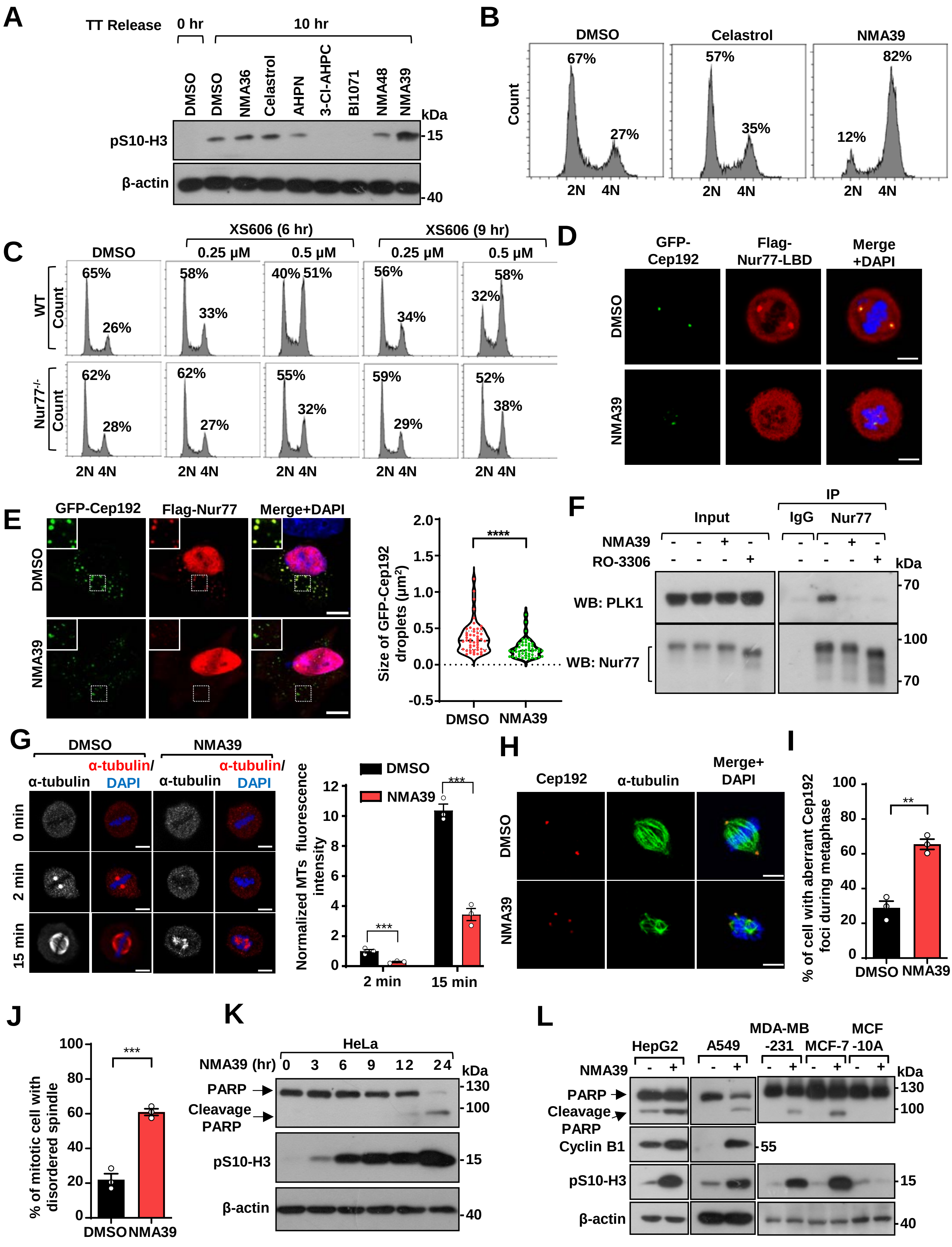

A
TT Release
0 hr
10 hr
DMSO
DMSO
NMA36
Celastrol
AHPN
3-Cl-AHPC
BI1071
NMA48
NMA39
kDa
pS10-H3
-15
β-actin
-40
B
DMSO
Celastrol
NMA39
Count
67%
27%
57%
35%
12%
82%
2N 4N
C
XS606 (6 hr)
XS606 (9 hr)
DMSO
0.25 μM
0.5 μM
WT
Count
Nur77-/-
65%
26%
58%
33%
40%
51%
56%
34%
32%
58%
62%
28%
62%
27%
55%
32%
59%
29%
52%
38%
2N 4N
D
GFP-Cep192
Flag-Nur77-LBD
Merge +DAPI
DMSO
NMA39
E
GFP-Cep192
Flag-Nur77
Merge+DAPI
DMSO
NMA39
Size of GFP-Cep192 droplets (μm²)
****
2.0
1.5
1.0
0.5
0.0
-0.5
DMSO
NMA39
F
Input
IP
IgG
Nur77
NMA39
RO-3306
kDa
WB: PLK1
WB: Nur77
70
100
70
G
DMSO
NMA39
α-tubulin
α-tubulin/DAPI
0 min
2 min
15 min
DMSO
NMA39
Normalized MTs fluorescence intensity
***
***
2 min
15 min
H
Cep192
α-tubulin
Merge+ DAPI
DMSO
NMA39
I
% of cell with aberrant Cep192 foci during metaphase
**
DMSO
NMA39
J
% of mitotic cell with disordered spindle
***
DMSO
NMA39
K
HeLa
NMA39 (hr)
0
3
6
9
12
24
kDa
PARP
Cleavage PARP
pS10-H3
β-actin
-130
-100
-15
-40
L
HepG2
A549
MDA-MB -231
MCF-7
MCF -10A
NMA39
kDa
PARP
Cleavage PARP
Cyclin B1
pS10-H3
β-actin
130
100
55
15
40

Figure S6

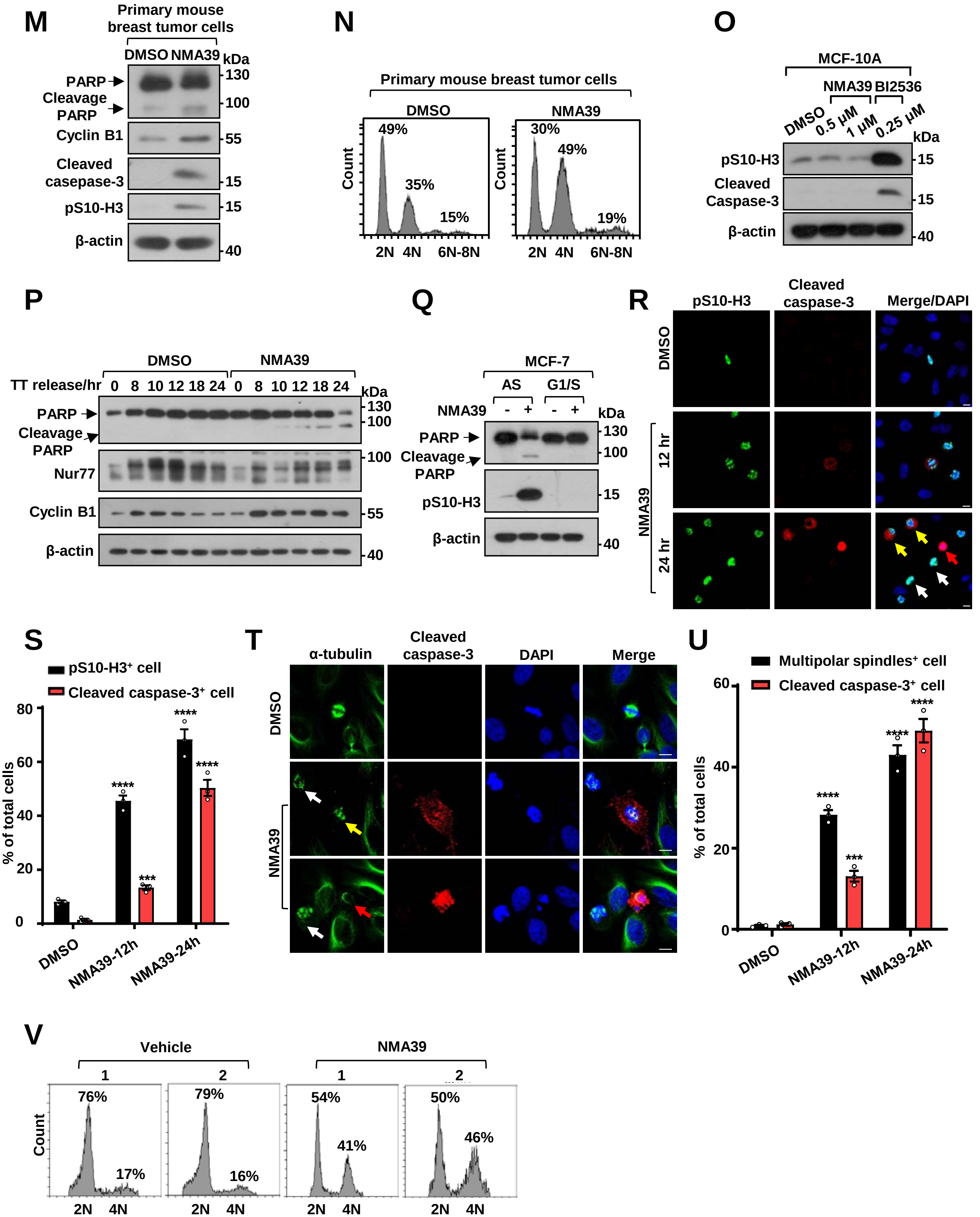
M
Primary mouse breast tumor cells
DMSO
NMA39
kDa
PARP
Cleavage PARP
Cyclin B1
Cleaved casepase-3
pS10-H3
β-actin
130
100
55
15
15
40
N
Primary mouse breast tumor cells
DMSO
NMA39
Count
49%
35%
15%
30%
49%
19%
2N
4N
6N-8N
O
MCF-10A
NMA39
BI2536
DMSO
0.5 μM
1 μM
0.25 μM
kDa
pS10-H3
Cleaved Caspase-3
β-actin
15
15
40
P
DMSO
NMA39
TT release/hr
0 8 10 12 18 24 0 8 10 12 18 24
kDa
PARP
Cleavage PARP
Nur77
Cyclin B1
β-actin
130
100
100
55
40
Q
MCF-7
AS
G1/S
NMA39
- + - +
kDa
PARP
Cleavage PARP
pS10-H3
β-actin
130
100
15
40
R
pS10-H3
Cleaved caspase-3
Merge/DAPI
DMSO
NMA39
12 hr
24 hr
S
pS10-H3+ cell
Cleaved caspase-3+ cell
% of total cells
0
20
40
60
80
****
****
****
***
DMSO
NMA39-12h
NMA39-24h
T
α-tubulin
Cleaved caspase-3
DAPI
Merge
DMSO
NMA39
U
Multipolar spindles+ cell
Cleaved caspase-3+ cell
% of total cells
0
20
40
60
****
***
****
****
DMSO
NMA39-12h
NMA39-24h
V
Vehicle
NMA39
1
2
1
2
Count
76%
17%
79%
16%
54%
41%
50%
46%
2N
4N

Figure S6. NMA39 inhibits centrosome maturation and induces mitotic arrest and apoptosis of tumor cells via targeting Nur77-Cep192-PLK1 signal axis.

(A) NMA39 but not other indicated compounds induces significantly mitotic arrest. HeLa cells released from TT block for 10 hr were treated with 1 μM Celastrol, 1 μM Celastrol derivates (NMA36, NMA39 and NMA48) and 1 μM Nur77 modulators (AHPN, 3-Cl-AHPC and BI1071) for 1 hr and analyzed by WB.

(B) NMA39 induces mitotic arrest in HeLa cells. HeLa cells were treated with 1 μM NMA39 or 1 μM Celastrol for 12 hr and analyzed by FACS.

(C) NMA39 induces mitotic arrest in a Nur77-dependent manner. HeLa cells (WT) and Nur77 depletion HeLa cells ($Nur77^{-/-}$) were treatment with the indicated concentration of NMA39 for the indicated time and analyzed by FACS.

(D) NMA39 blocks Nur77-LBD colocalization with Cep192 at the centrosome. HeLa cells transfected with Flag-Nur77-LBD and GFP-Cep192 were released from TT block for 10 hr and treated with NMA39 (1 μM) for 1 hr then analyzed by IF. Scale bar, 10 μm.

(E) NMA39 reduces the size of droplet formed by GFP-Cep192 and Flag-Nur77. HeLa cells transfected with GFP-Cep192 and Flag-Nur77 were treated with 1 μM NMA39 for 6 hr and analyzed by immunostaining. GFP-Cep192 (green), Flag-Nur77 (red), and DAPI (blue). Scale bar, 10 μm. The size of droplets was calculated (n=50 cells per group; ****p< 0.0001; two-tailed unpaired *t*-test).

(F) NMA39 inhibits endogenous Nur77 interaction with PLK1 during mitosis. Mitotic HeLa cells released from TT block for 10 hr were treated with 1 μM NMA39 or 10 μM RO-3306 for 30 min and analyzed by CoIP assay.

(G) NMA39 attenuates the activity of MT nucleation and spindle assembly at the centrosome**.** HeLa cells were cold treated, reheated for indicated time at 37°C in the presence or absence of 1 μM NMA39 and then stained with α-tubulin (red) and DAPI (blue). Scale bar, 10 μm. Relative MT nucleation activity at the centrosome during metaphase was shown (n= 40 cells across three experiments; **p= 0.0044, ***p= 0.0003; two-tailed unpaired t-test).

(H-J) NMA39 induces aberrant Cep192 foci and disordered spindle in primary mouse breast tumor cells. Primary mouse breast tumor cells isolated from MMTV-PyMT mice were treated with 1 μM NMA39 for 12 hr then analyzed by IF. Cep192 (red), α-tubulin (green), and DAPI (blue). Scale bar,10 μm. Percentage of mitotic cells with aberrant Cep192 foci (**I**) or disordered spindle (**J**) were calculated respectively (n= 100 cells across three experiments; **p= 0.0017, ***p= 0.0006; two-tailed unpaired t-test).

(K) NMA39 induces mitotic arrest and apoptosis in a time-dependent manner. HeLa cells treated with 1 μM NMA39 for the indicated time were analyzed by WB.

(L) NMA39 induces mitotic arrest and apoptosis in many tumor cell lines but not breast epithelial cell line MCF-10A. The indicated cell lines were treated with 0.5 μM NMA39 for 12 hr and analyzed by WB.

(M, N) NMA39 induces mitotic arrest and apoptosis in primary mouse breast tumor cells. Primary mouse breast tumor cells isolated from MMTV-PyMT mice were treated with 1 μM NMA39 for 12 hr then analyzed by WB **(M)** and FACS **(N)**.

(O) BI2536 but not NMA39 induces mitotic arrest and apoptosis in MCF-10A. MCF-10A were treated with 0.5 μM NMA39, 1 μM NMA39 or 0.25 μM BI2536 for 12 hr and analyzed by WB.

(P) HeLa cells synchronized at the G1/S transition by TT treatment were incubated with or without 0.5 μM NMA39 for the indicated time after released from thymidine treatment and analyzed by WB.

(Q) NMA39 induces apoptosis in a mitotic arrest-dependent manner. Asynchronous MCF-7 cells (AS) or MCF-7 cells synchronized at the G1/S transition by TT block were incubated with 1 μM NMA39 for 6 hr and analyzed by WB.

(R, S) NMA39 induces time-dependent mitotic arrest and apoptosis. HeLa cells were treated with 0.5 μM NMA39 for 12 hr and 24 hr then analyzed by IF. pS10-H3 (green), cleaved caspase-3 (red), and DAPI (blue). Scale bar, 10 μm. A subset of cells was in early mitotic arrest, positive for mitotic markers but negative for apoptosis (white arrow). Other cells exhibited both mitotic arrest and apoptotic signals (yellow arrow), while a third population showed diminished mitotic signals and strong apoptotic signals, indicating late-stage mitotic arrest (red arrow). Percentage of pS10-H3$^+$ cell and cleaved caspase-3$^+$ cell were calculated respectively (n= 2000 cells across three experiments; ***p=0.0003, ****p< 0.0001; two-tailed unpaired t-test).

(T, U) NMA39 induces time-dependent spindle multipolarity and apoptosis. HeLa cells were treated with 0.5 μM NMA39 for 12 hr and 24 hr then analyzed by IF. α-tubulin (green), cleaved caspase-3 (red), and DAPI (blue). Scale bar, 10 μm. Multipolar spindles were observed in a subset of cells without apoptotic signals (white arrow), some displayed both (yellow arrow), and others showed strong apoptosis with few discernible spindles (red arrow). Percentage of multipolar spindles$^+$ cell and cleaved caspase-3$^+$ cell were calculated

respectively (n= 2000 cells across three experiments; ***p=0.001, ****p< 0.0001; two-tailed unpaired t-test).

(V) NMA39 induces mitotic arrest in breast tumor specimens from MMTV-PyMT mice. Breast tumor specimens excised from MMTV-PyMT mice treated with or without NMA39 were digested by collagenase then isolated tumor cells were collected and analyzed by FACS, *related to Fig. 6O*.

All quantitative data in this figure are presented as mean values ±SEM.

Figure S7

A

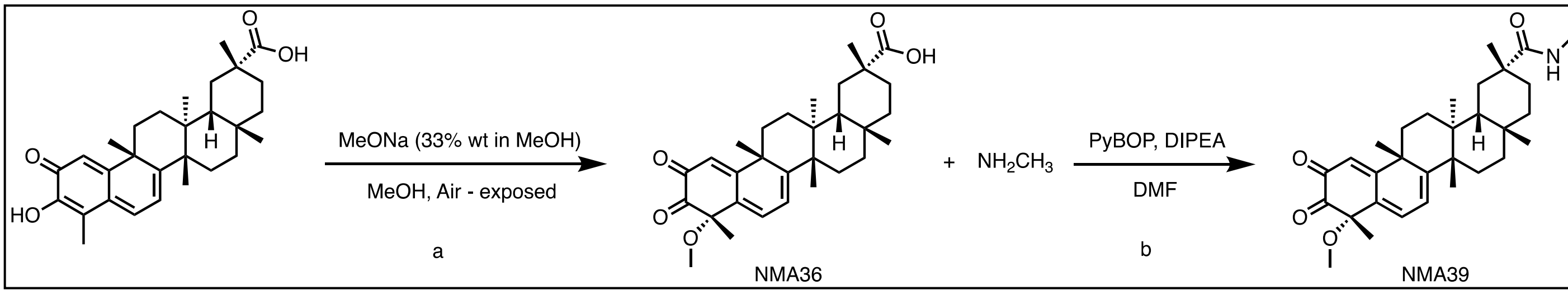

B

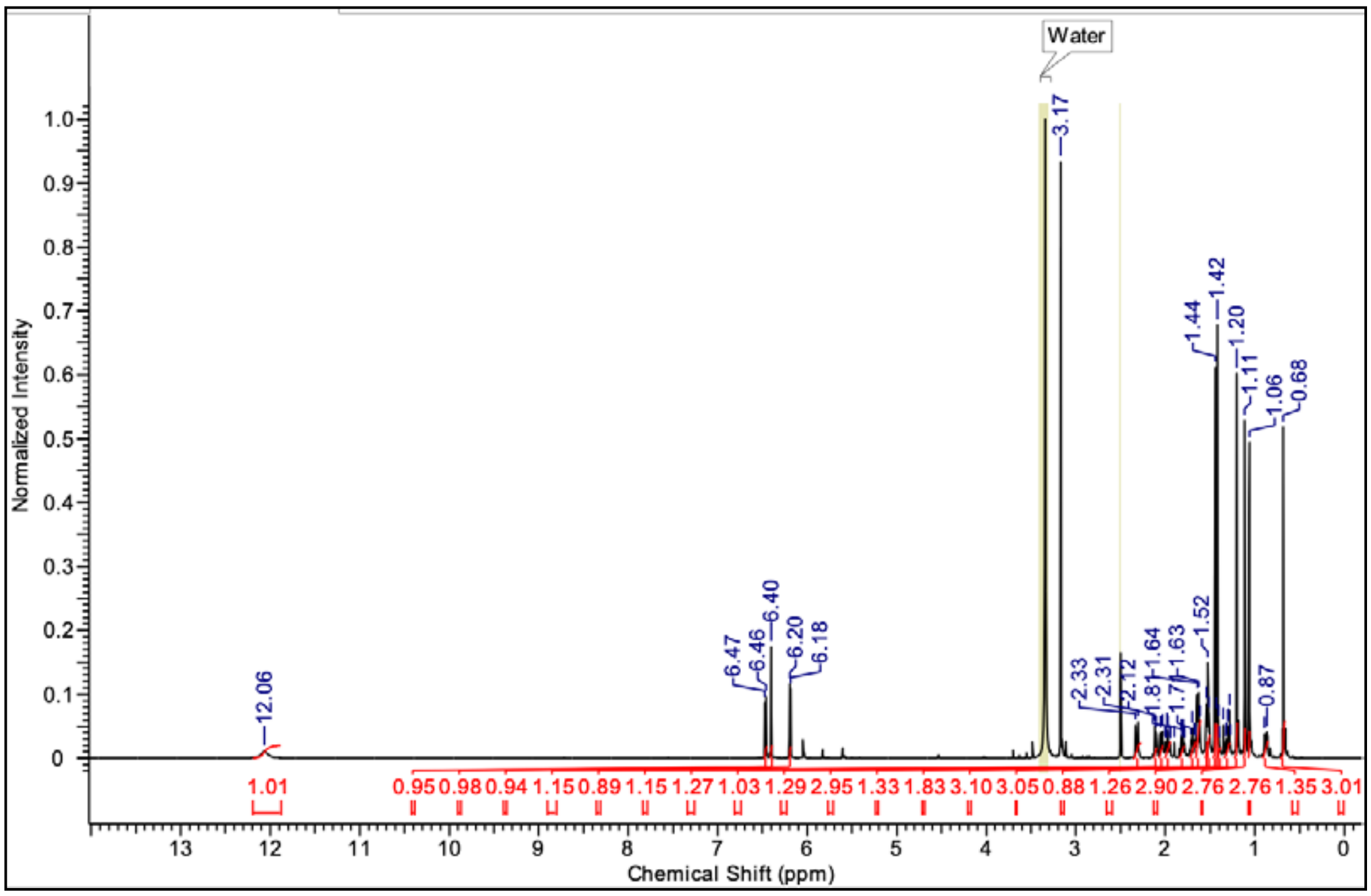

C

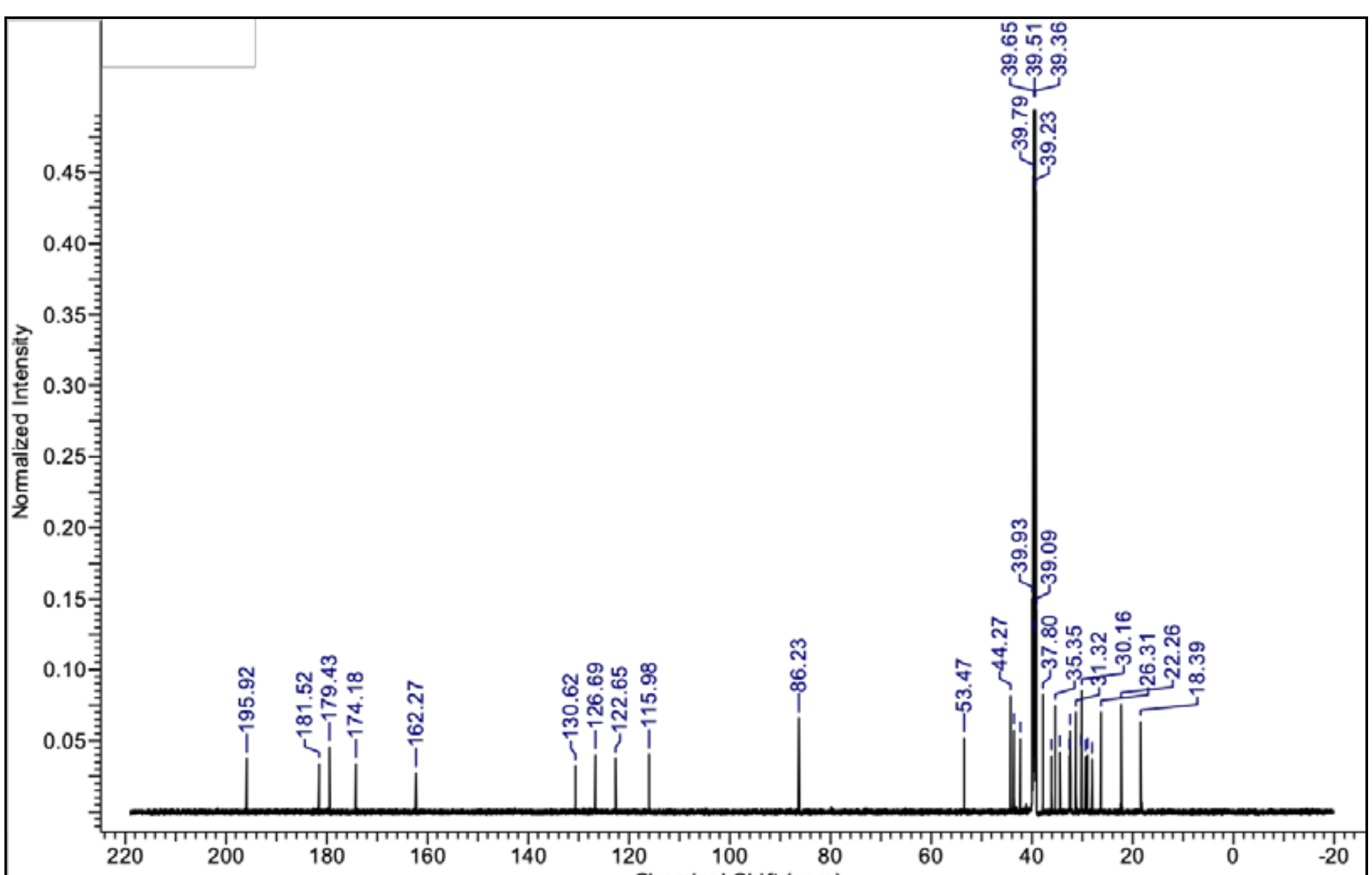

Figure S7

D

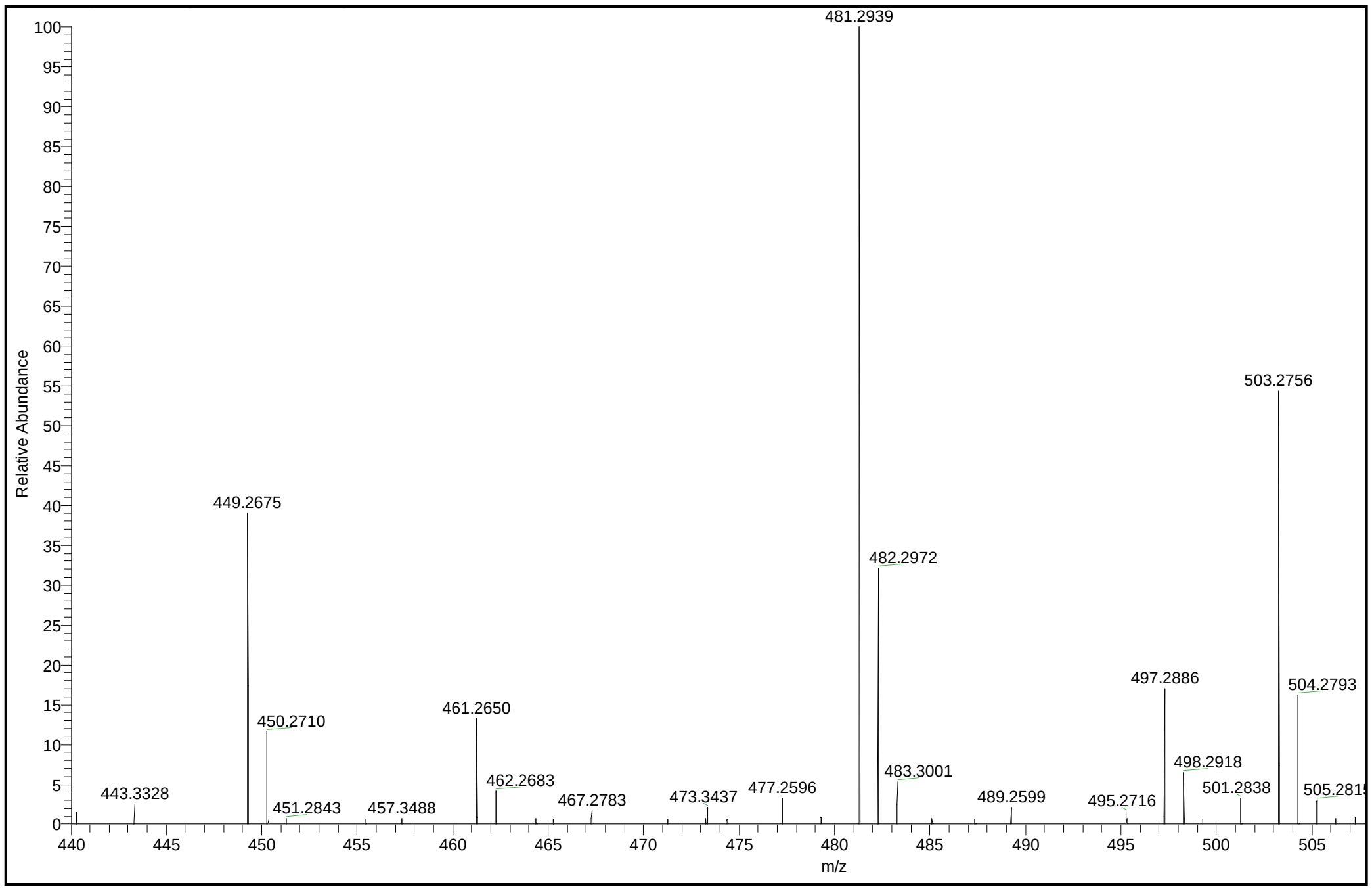

Relative Abundance
m/z
481.2939
503.2756
449.2675
482.2972
497.2886
504.2793
461.2650
450.2710
498.2918
483.3001
462.2683
501.2838
505.2815
443.3328
451.2843
457.3488
467.2783
473.3437
477.2596
489.2599
495.2716

E

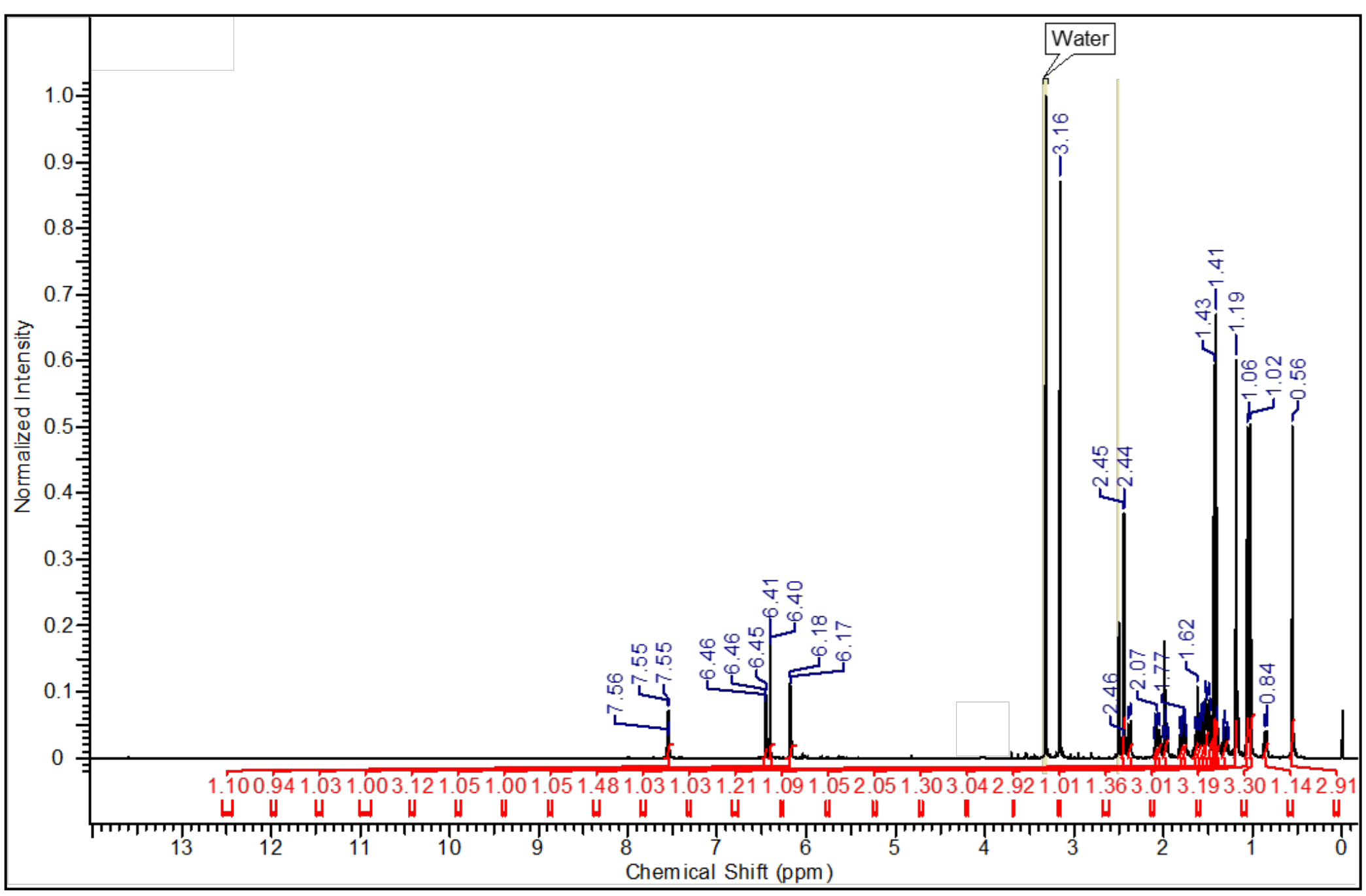

Water
Normalized Intensity
Chemical Shift (ppm)

Figure S7

F

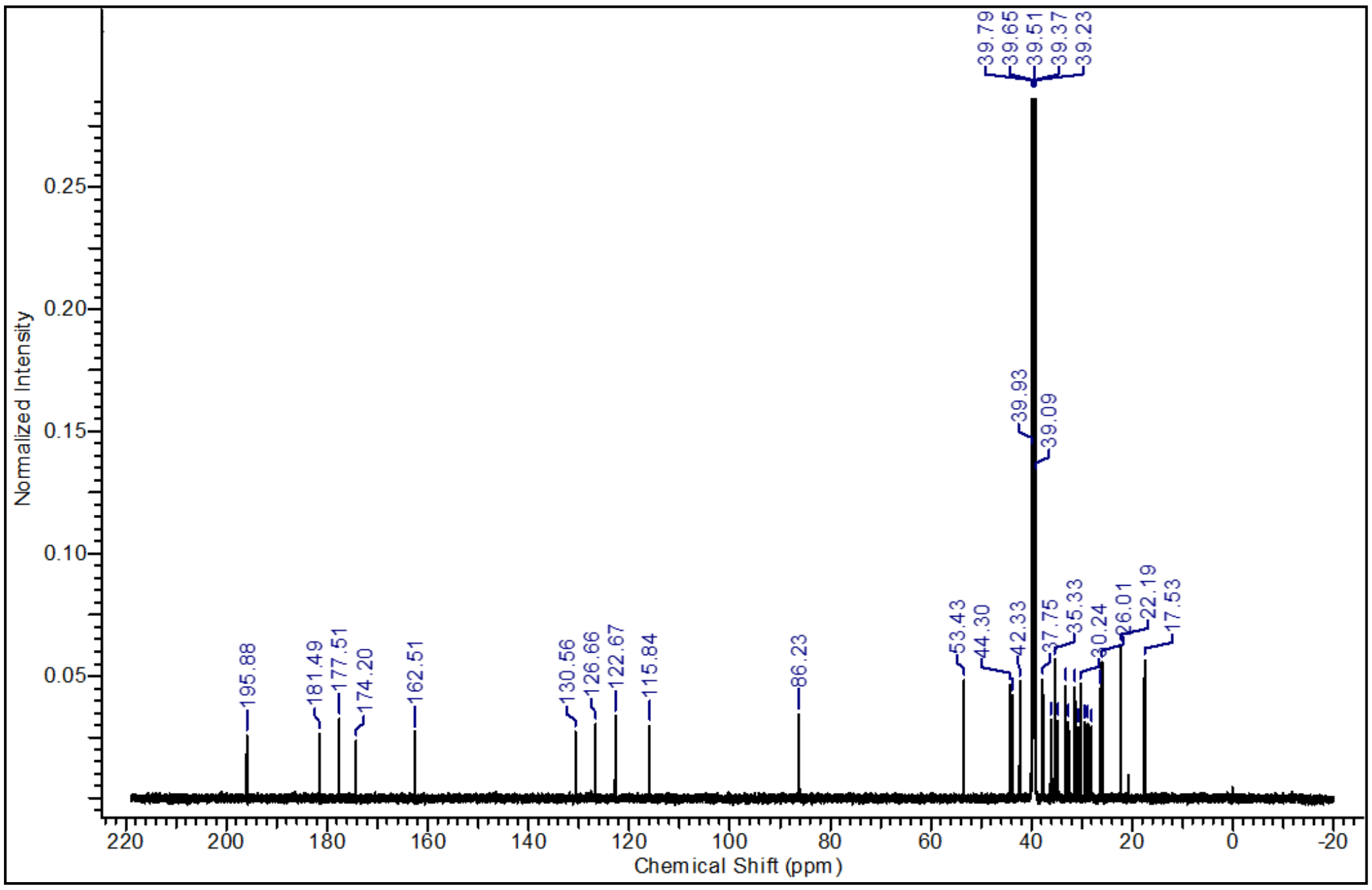
39.79
39.65
39.51
39.37
39.23
39.93
39.09
195.88
181.49
177.51
174.20
162.51
130.56
126.66
122.67
115.84
86.23
53.43
44.30
42.33
37.75
35.33
30.24
26.01
22.19
17.53
Normalized Intensity
Chemical Shift (ppm)

G

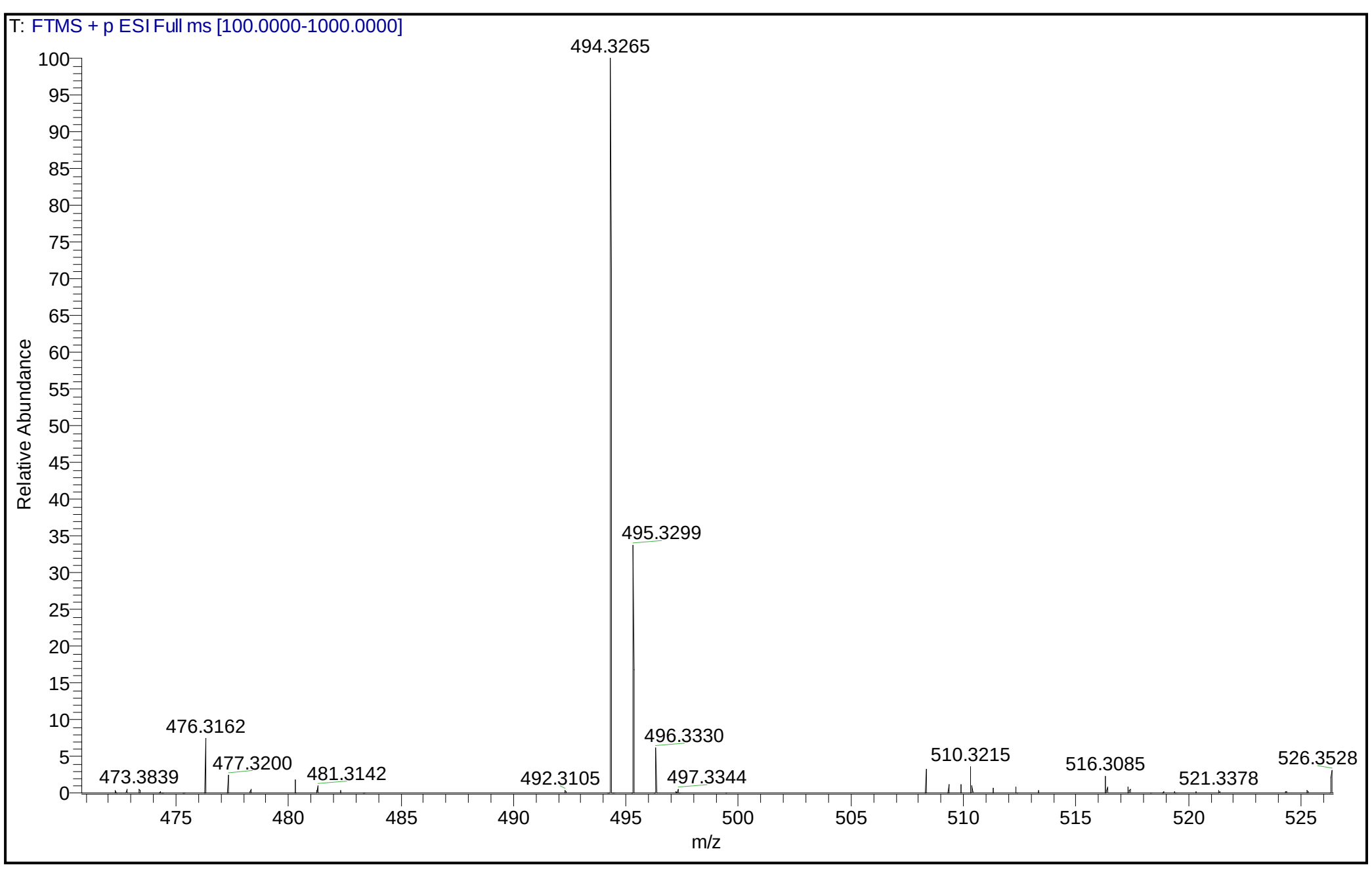
T: FTMS + p ESI Full ms [100.0000-1000.0000]
494.3265
495.3299
476.3162
477.3200
473.3839
481.3142
492.3105
496.3330
497.3344
510.3215
516.3085
521.3378
526.3528
Relative Abundance
m/z

Figure S7. Synthesis and characterization of NMA39.

(A) The synthetic method of NMA36 and NMA39, Reagents and conditions: a) Sodium methoxide (33% wt. in MeOH), air, room temperature; b) CH_3NH_2 (33% in absolutely ethanol), PyBOP, DIPEA, DMF, room temperature.

(B) The ^{1}H-NMR (600 MHz) spectrum of NMA36 with DMSO-D_6;

(C) The ^{13}C-NMR (151 MHz) spectrum of NMA36 with DMSO-D_6.

(D) The high resolution mass spectrum of NMA36.

(E) The ^{1}H-NMR (600 MHz) spectrum of NMA39 with DMSO-D_6;

(F) The ^{13}C-NMR (151 MHz) spectrum of NMA39 with DMSO-D_6.

(G) The high resolution mass spectrum of NMA39.

Chemical shifts were reported in units (ppm) by assigning DMSO resonance in the ^{1}H spectrum as 2.50 ppm and in the ^{13}C spectrum as 39.51 ppm.
