# Supplementary material for: Cdk1-phosphorylated Nur77 accumulates at the centrosome during mitosis to regulate the Cep192–PLK1 signaling axis
Source: Cell Death Dis. 2026 Jun 3;17(1):695. doi: 10.1038/s41419-026-08870-3 (PMC13448657; doi:10.1038/s41419-026-08870-3)

Fig. 1A

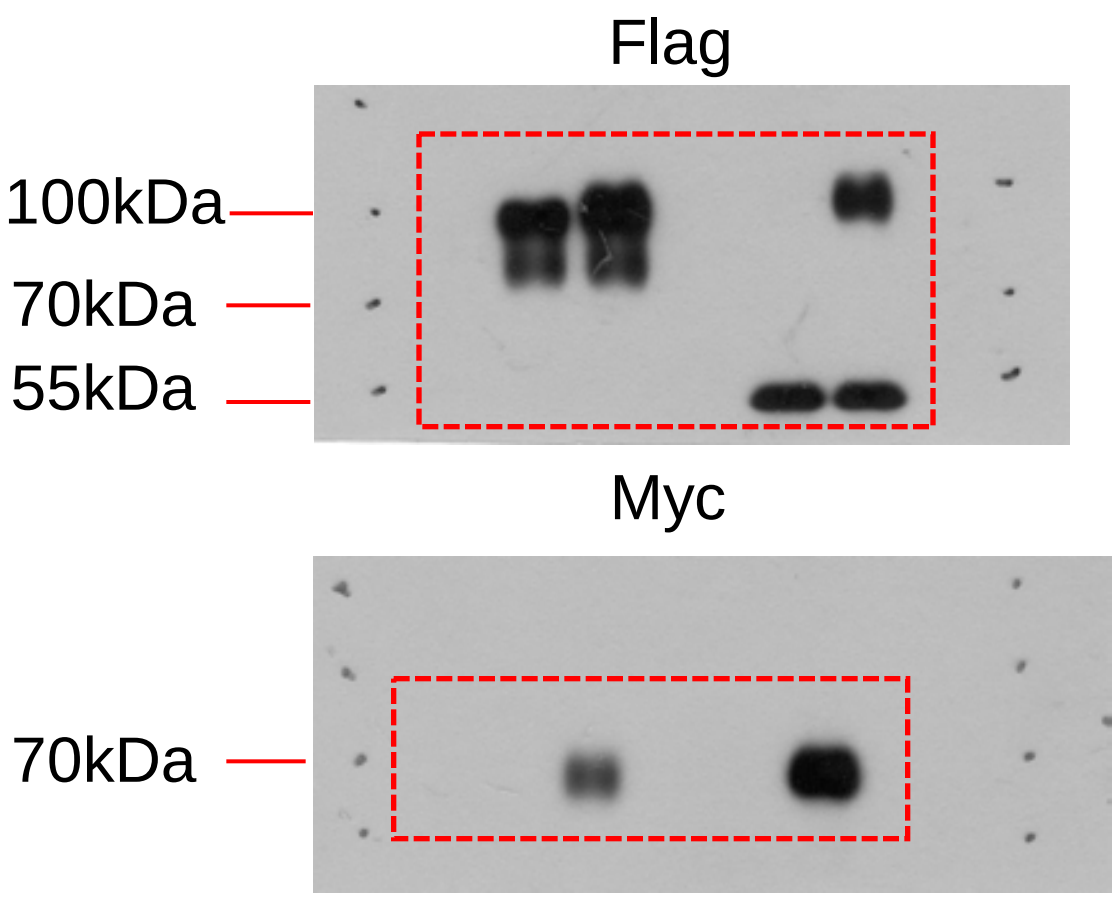

Fig. 1C

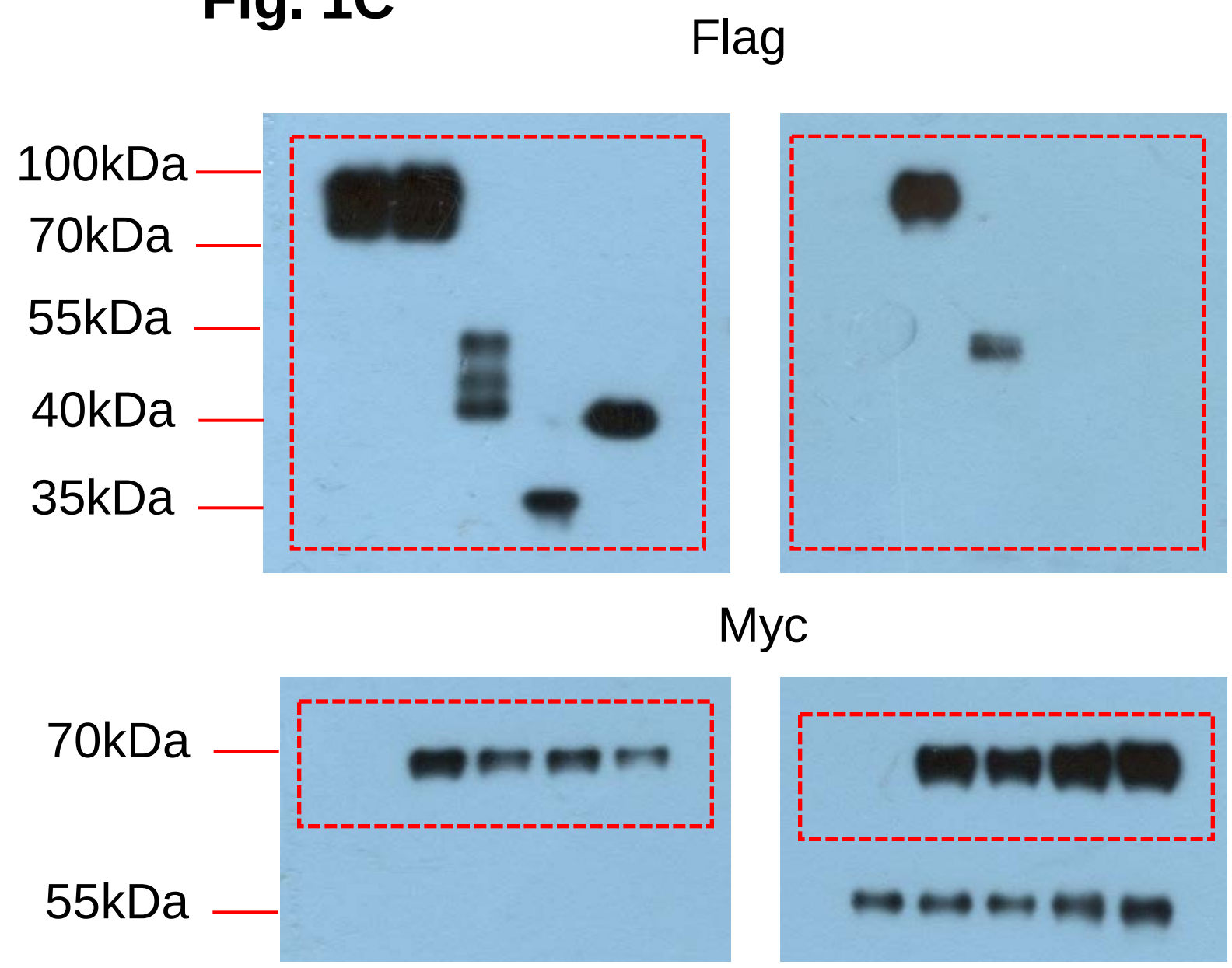

Fig. 1D

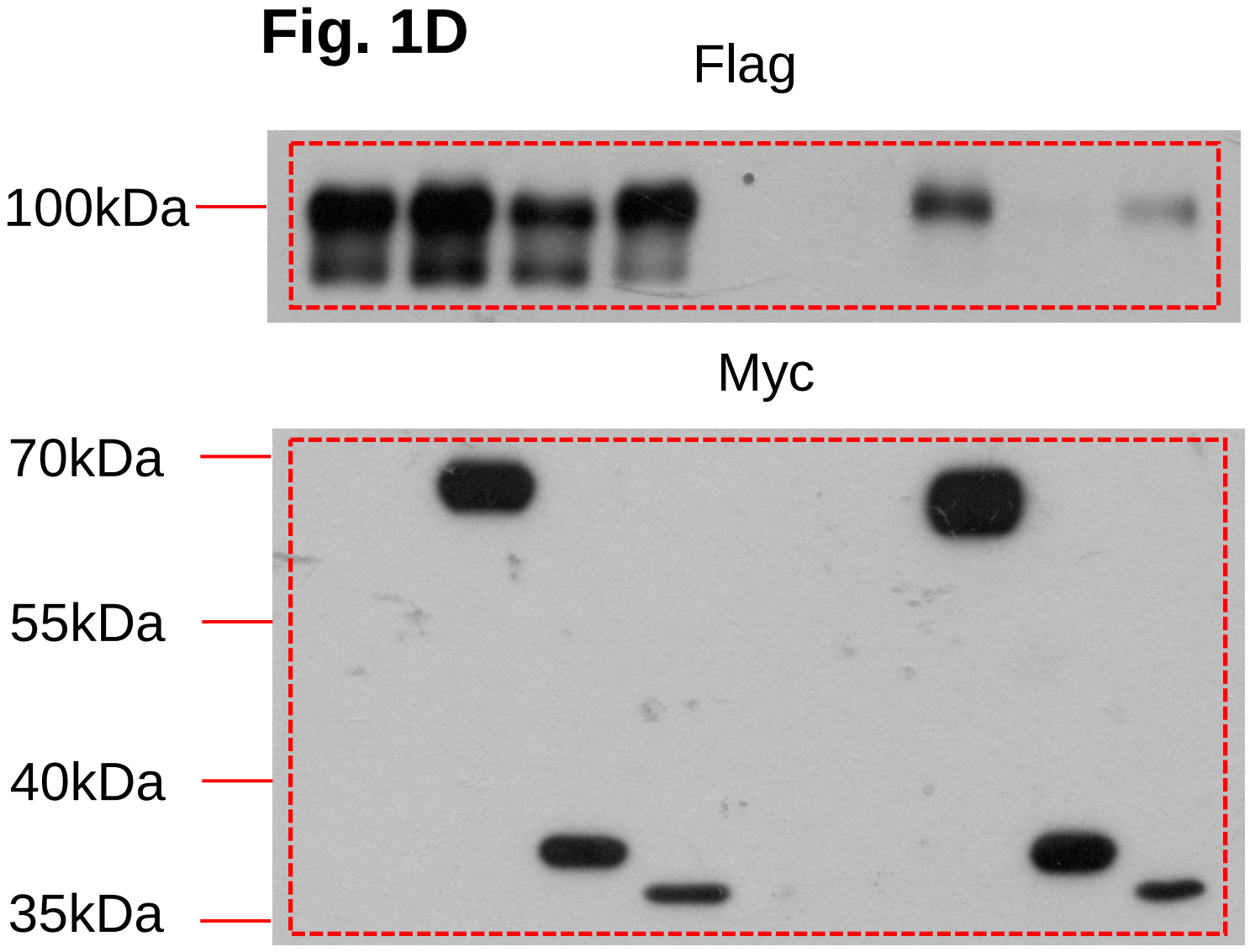

Fig. 1E

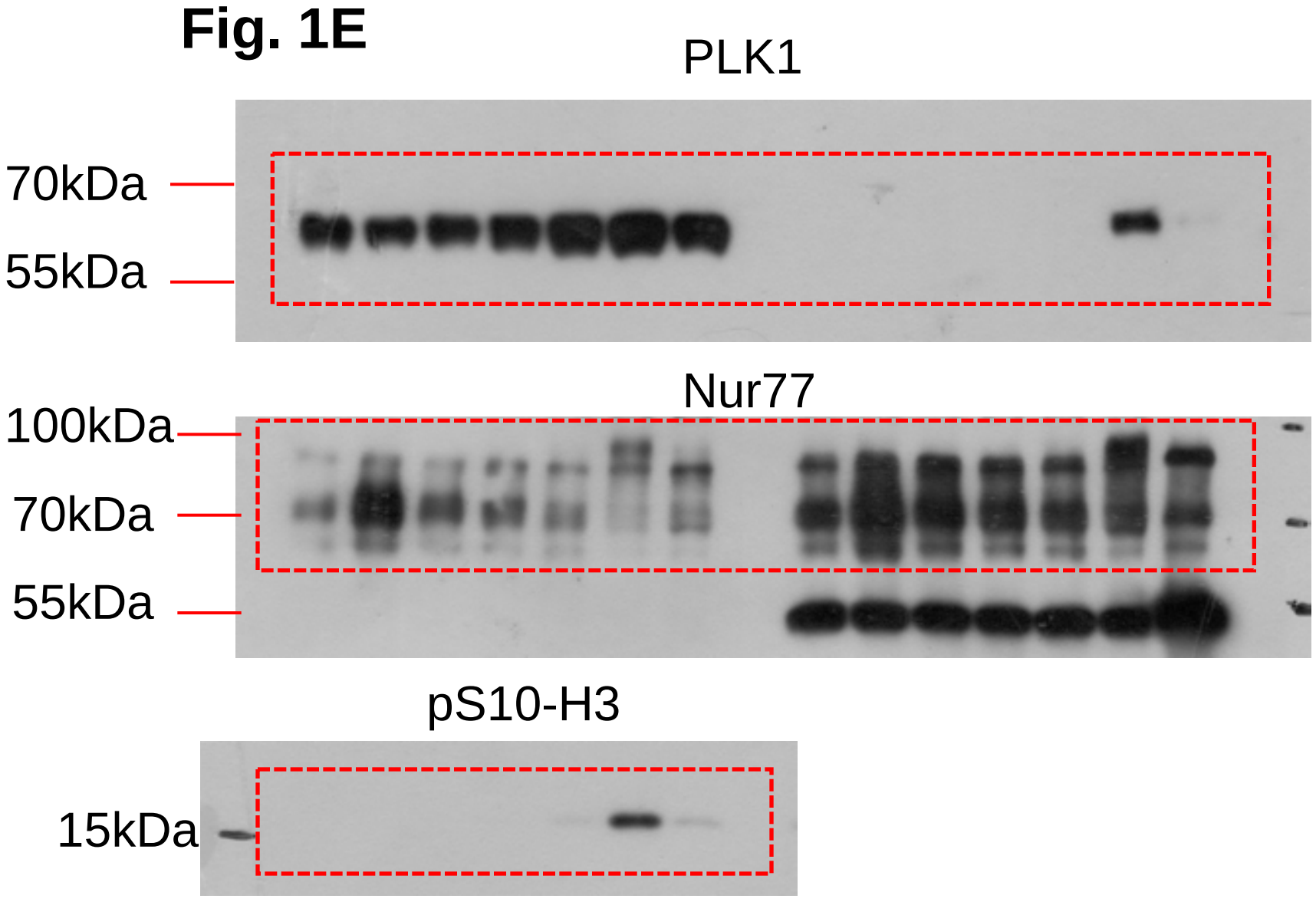

Fig. 1F

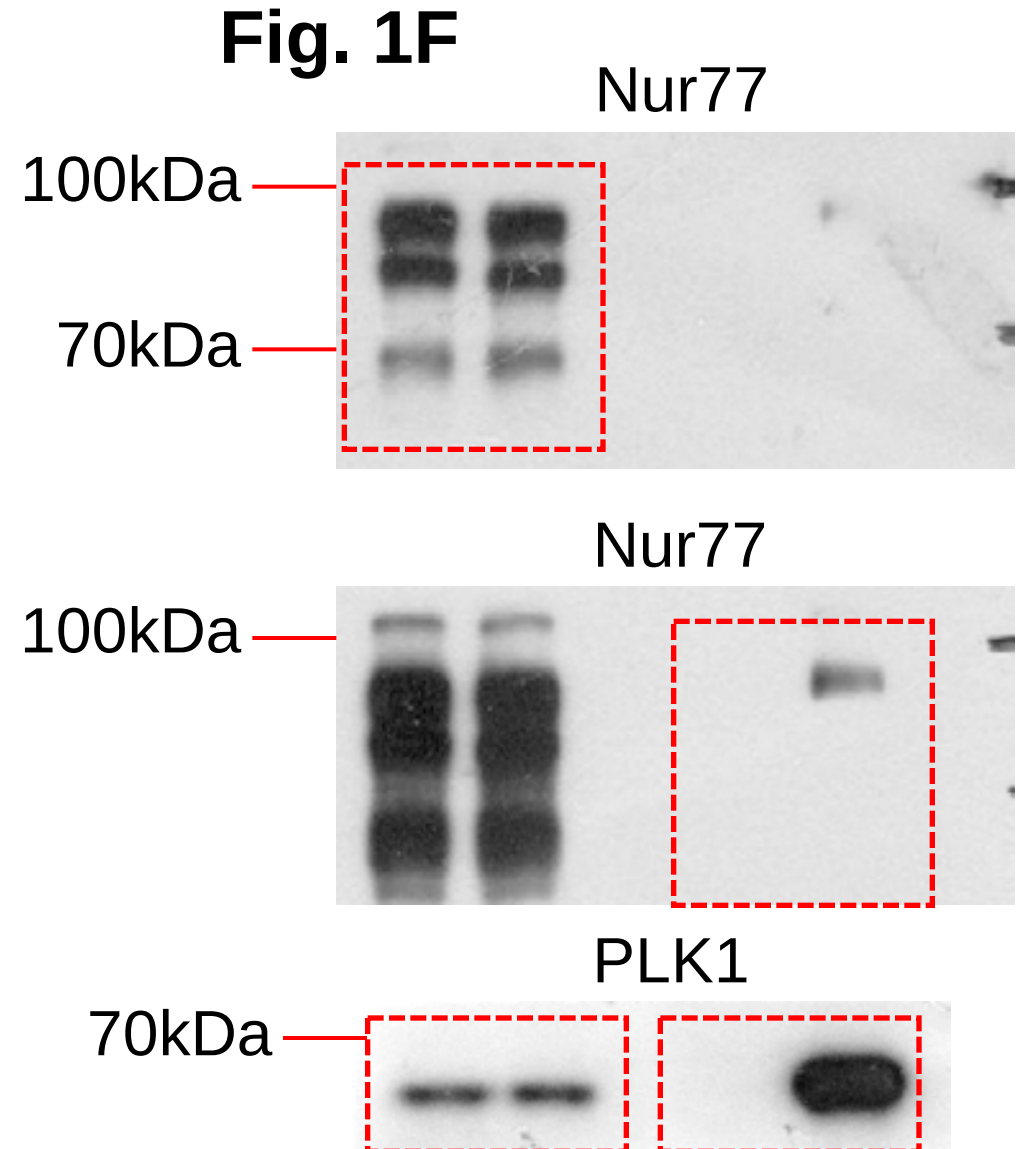

Fig. 2B

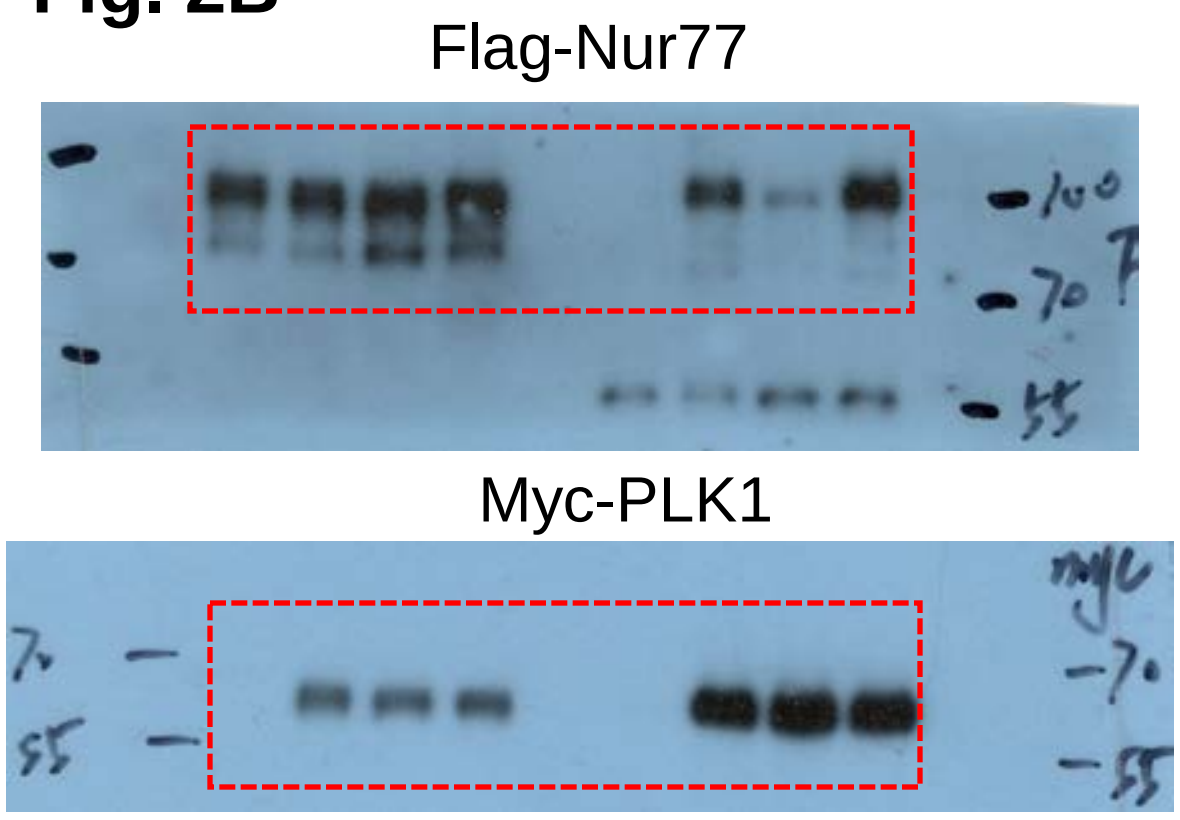

Fig. 2C

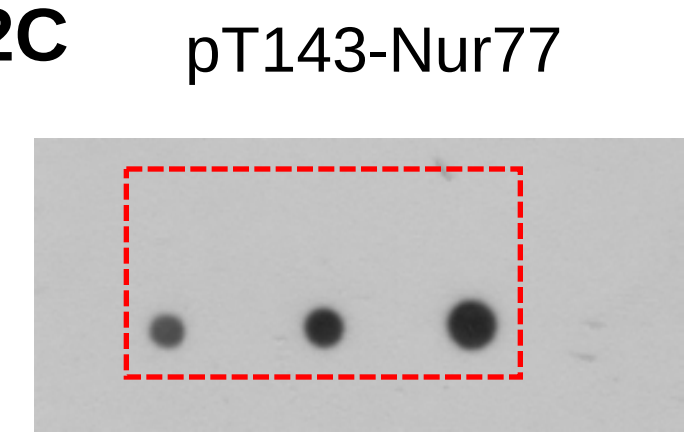

Fig. 2D

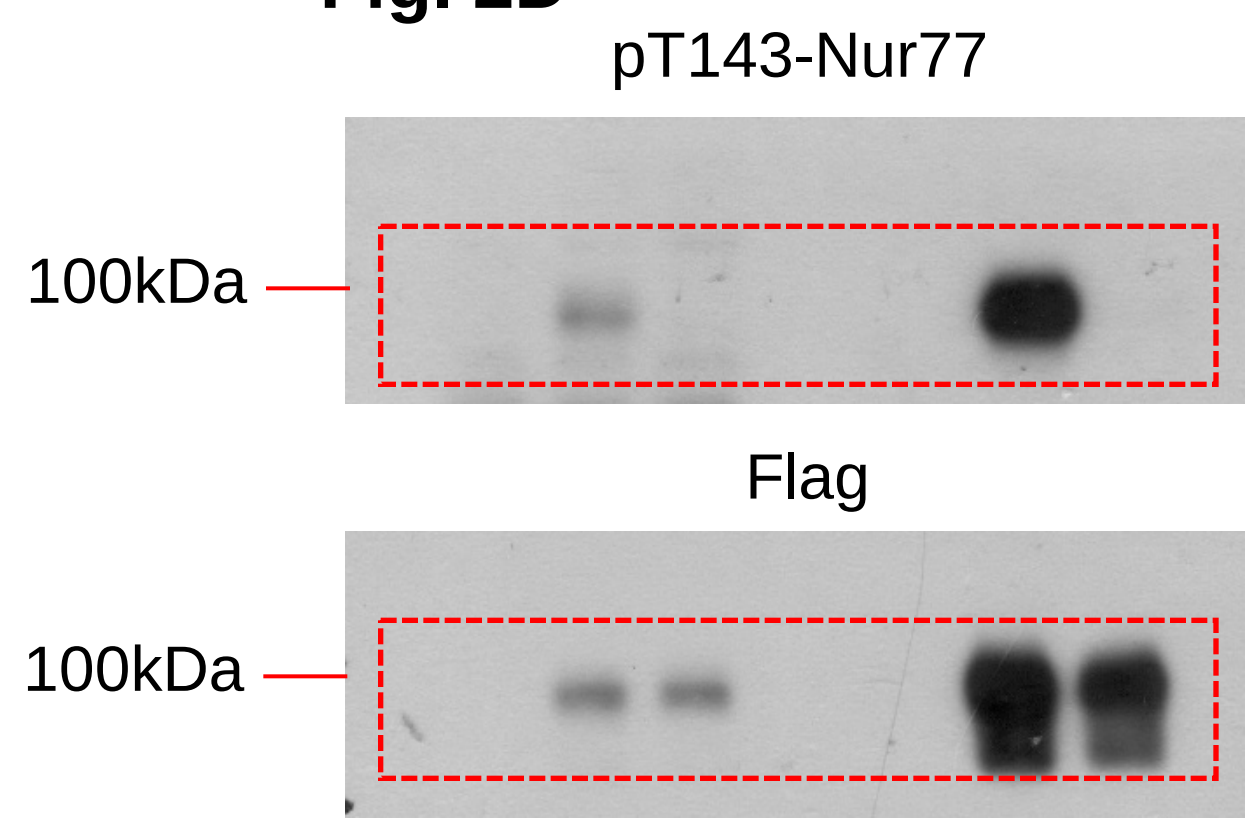

Fig. 2E

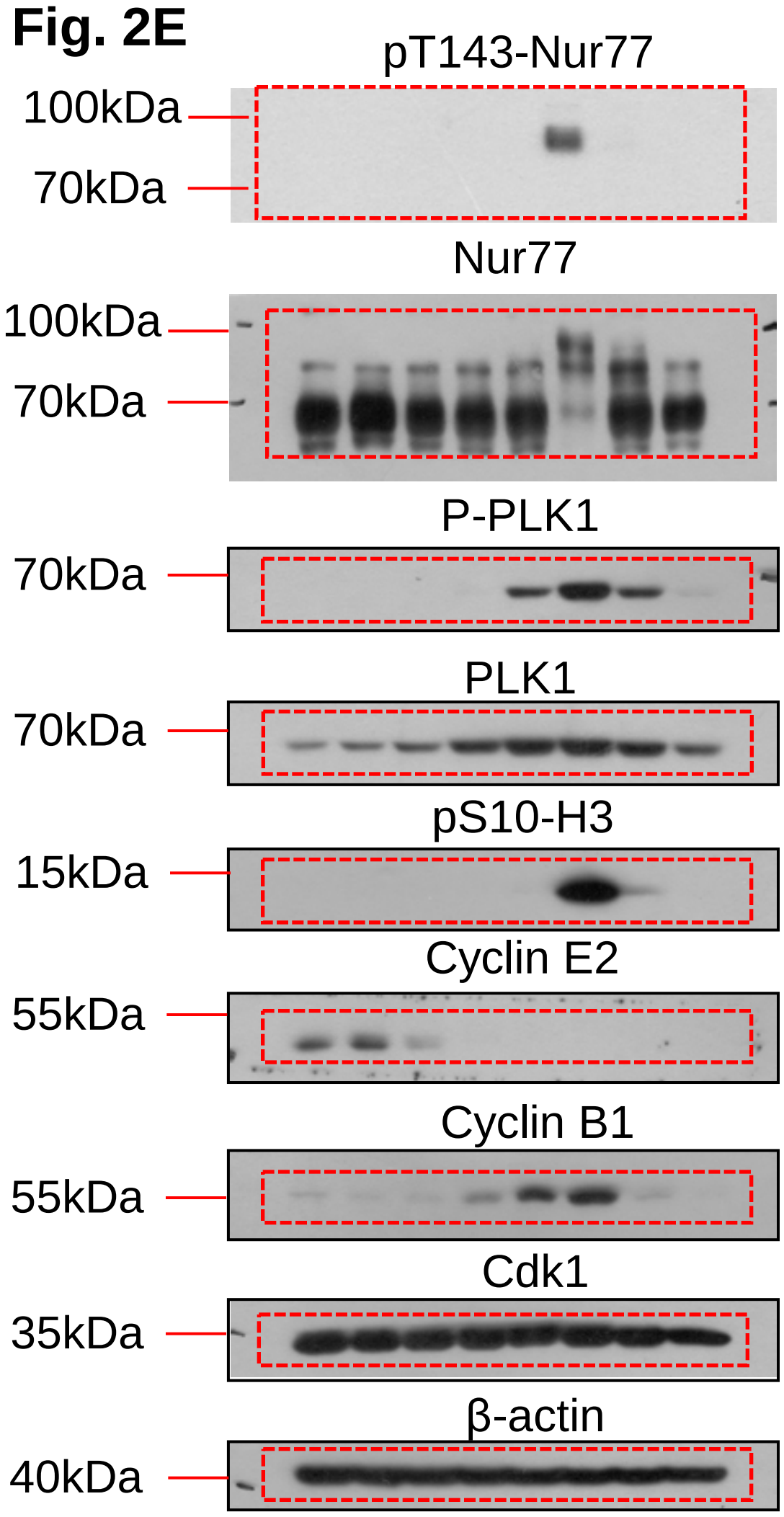

Fig. 2F

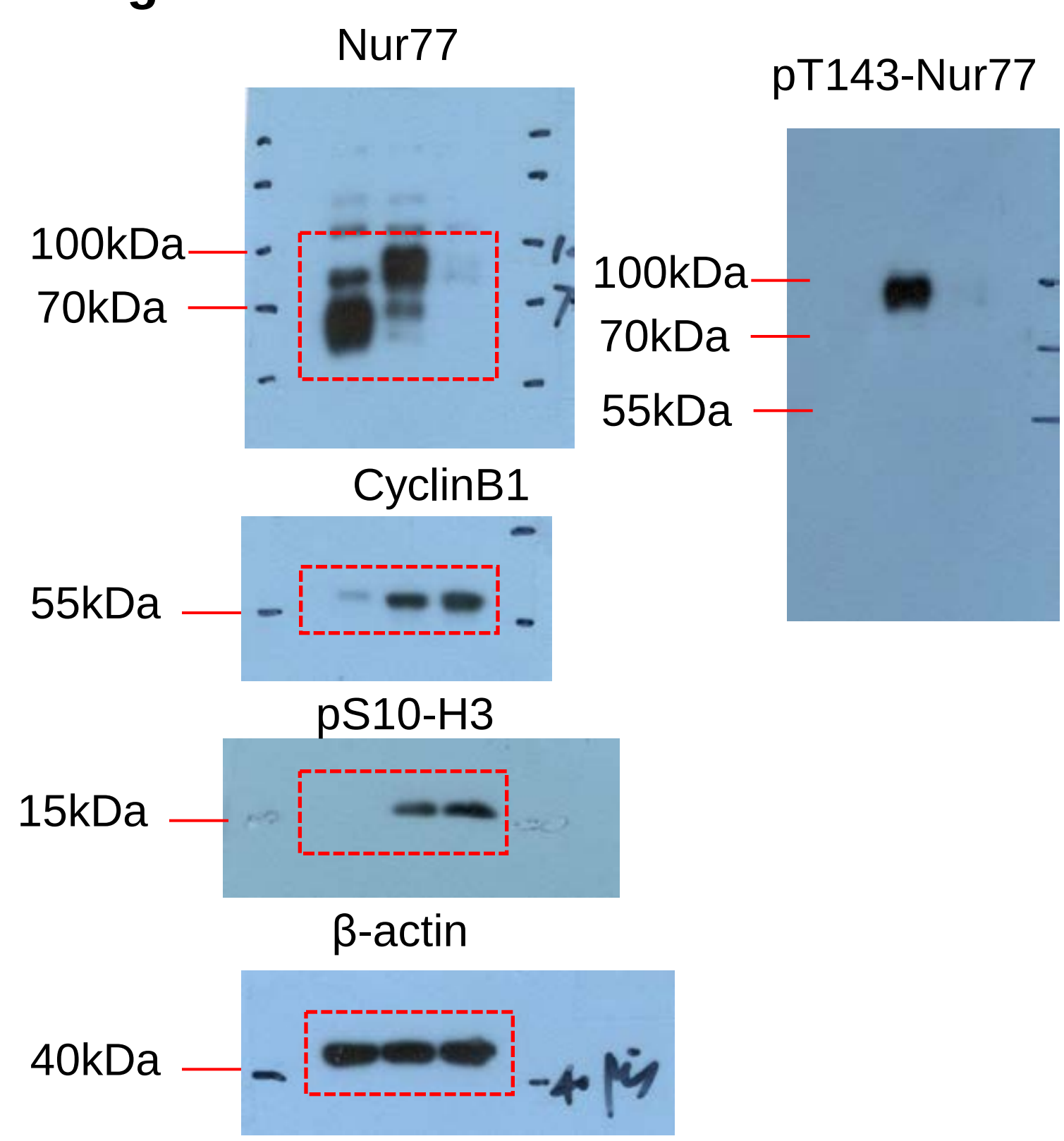

Fig. 2G

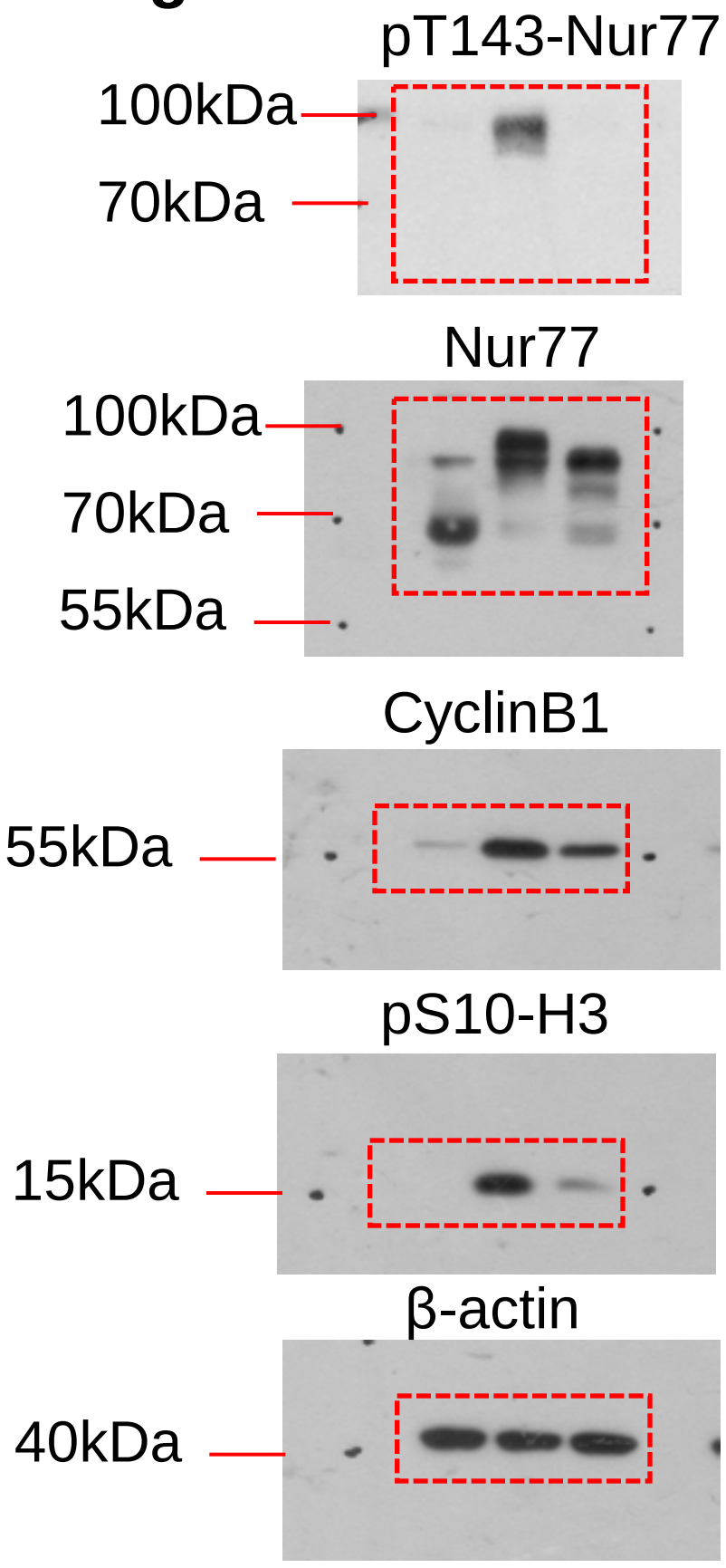

Fig. 2I

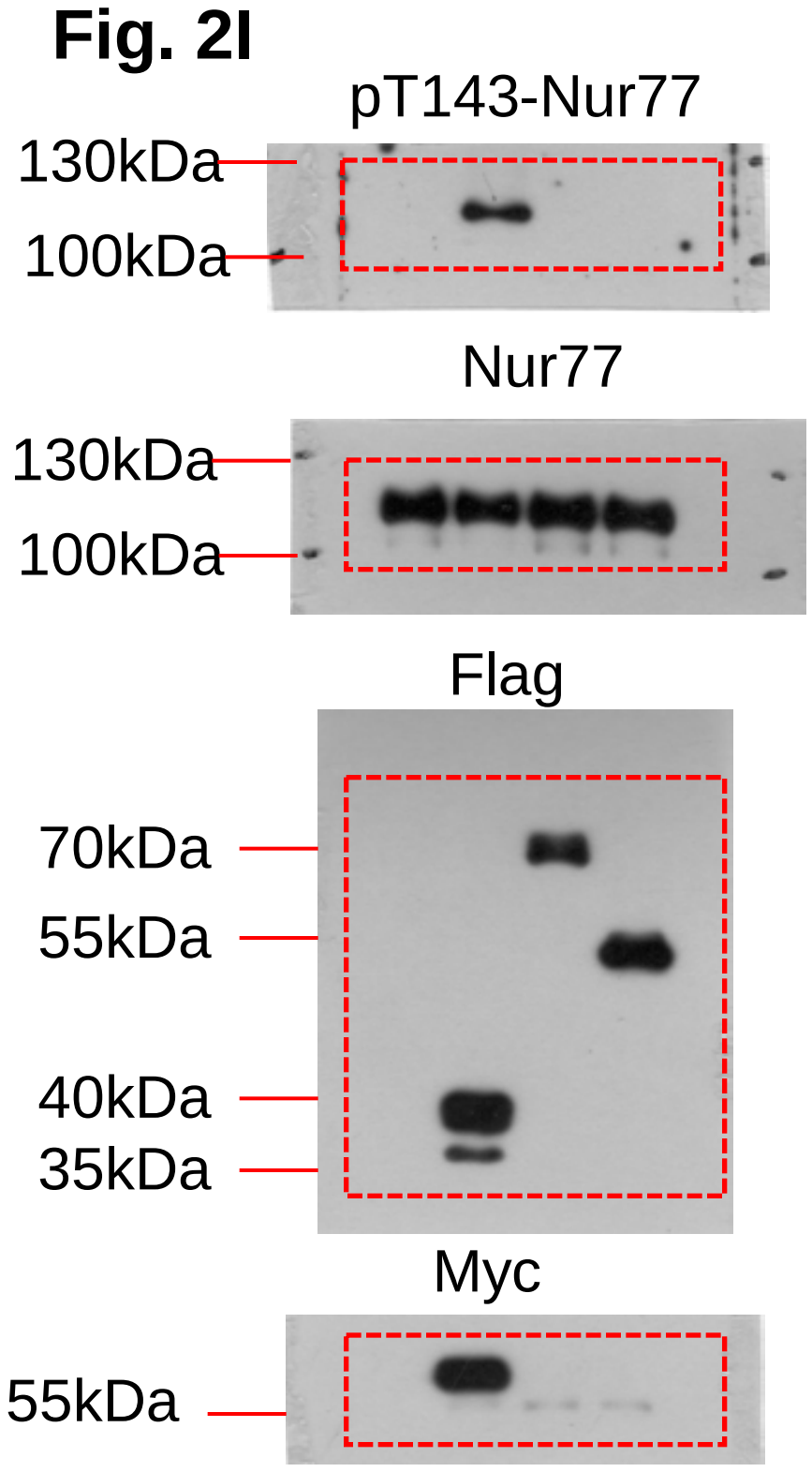

Fig. 2H

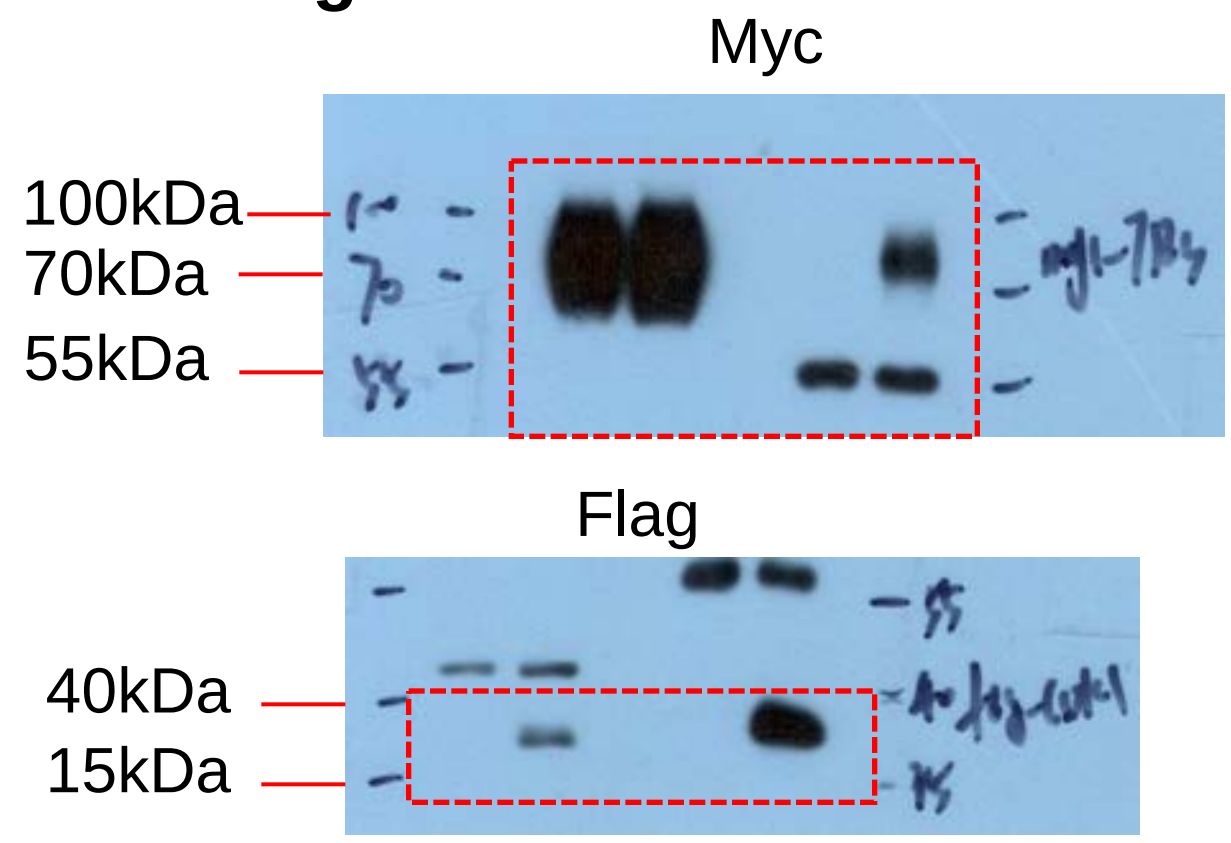

Fig. 2J

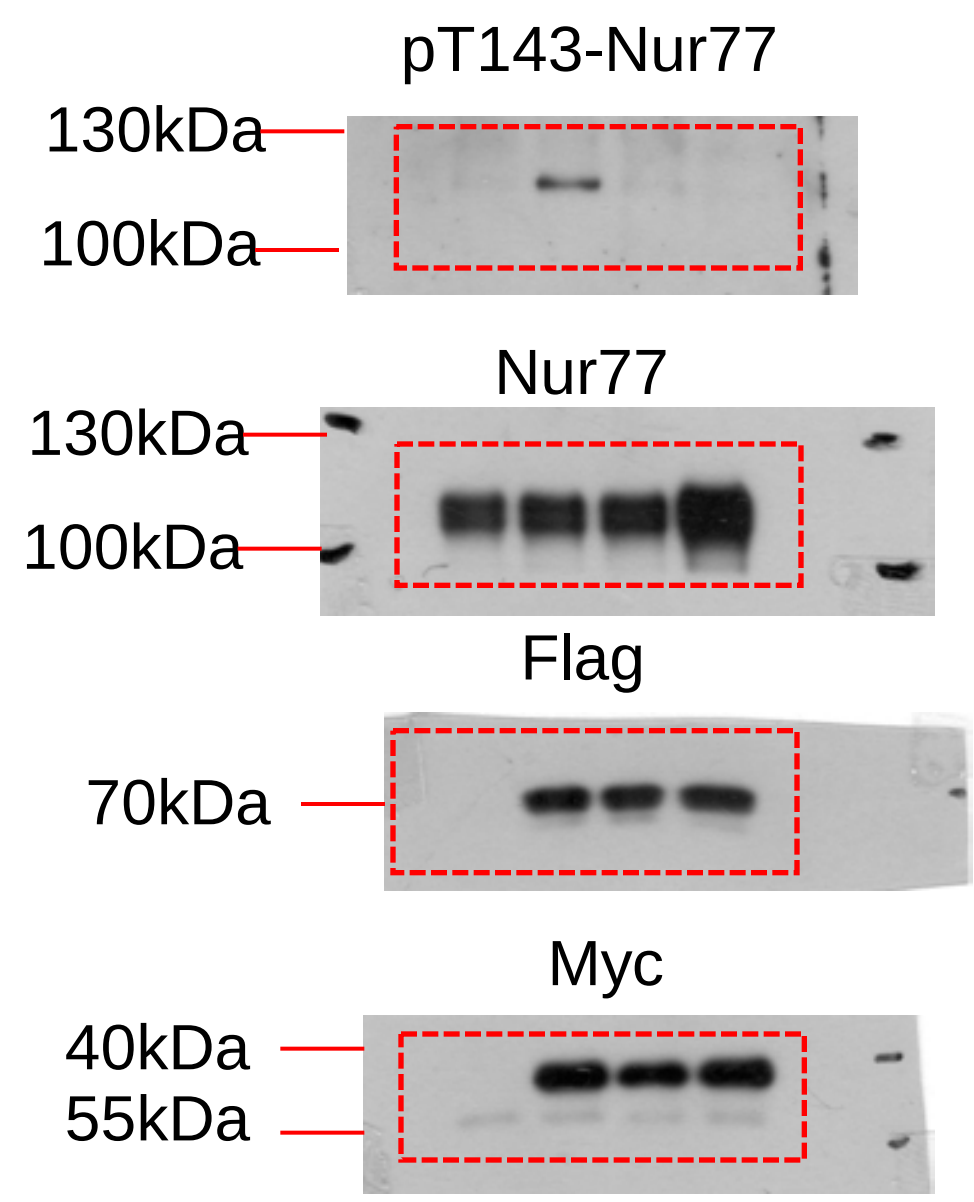

Fig. 2K

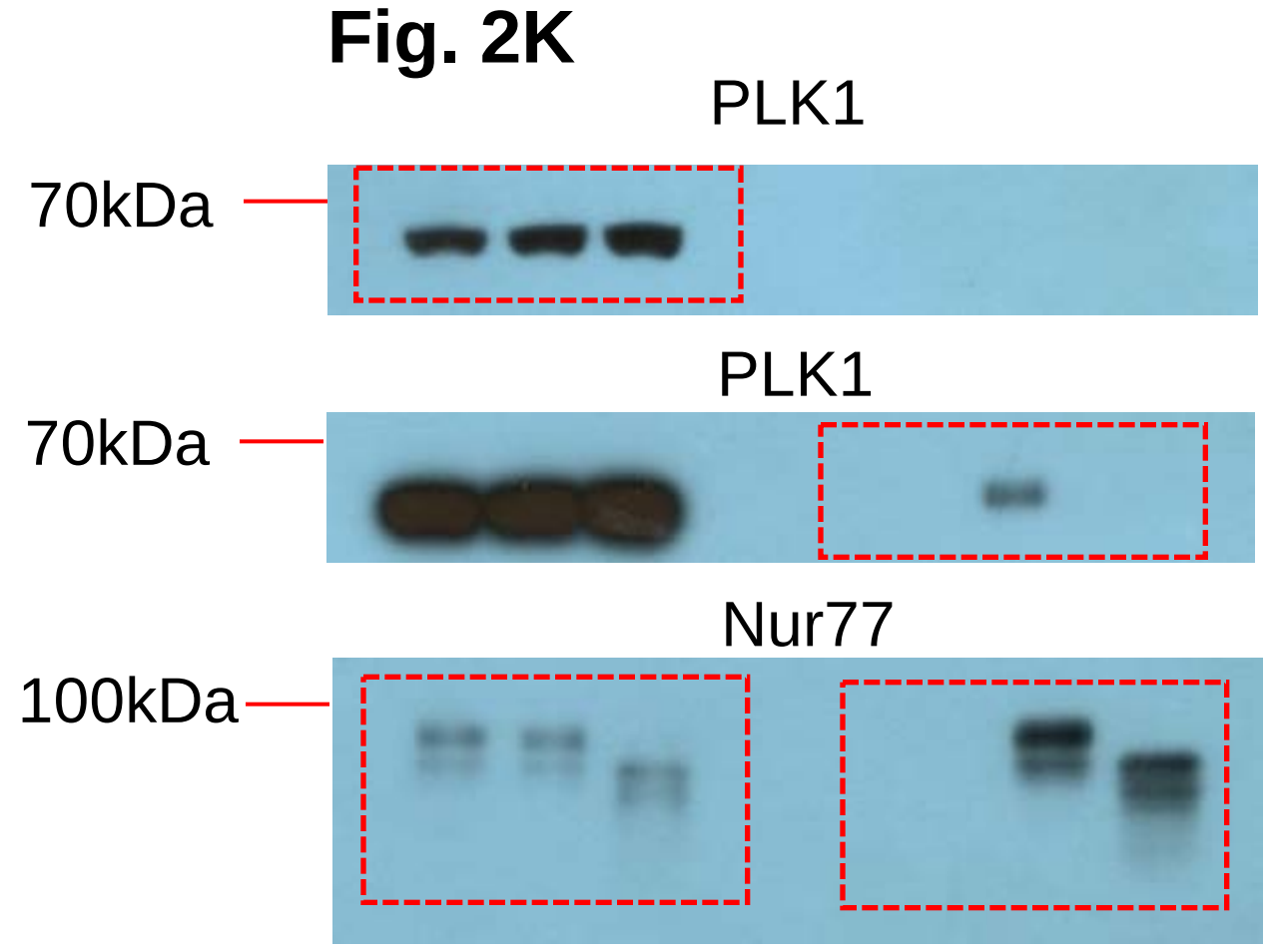

Fig.3H

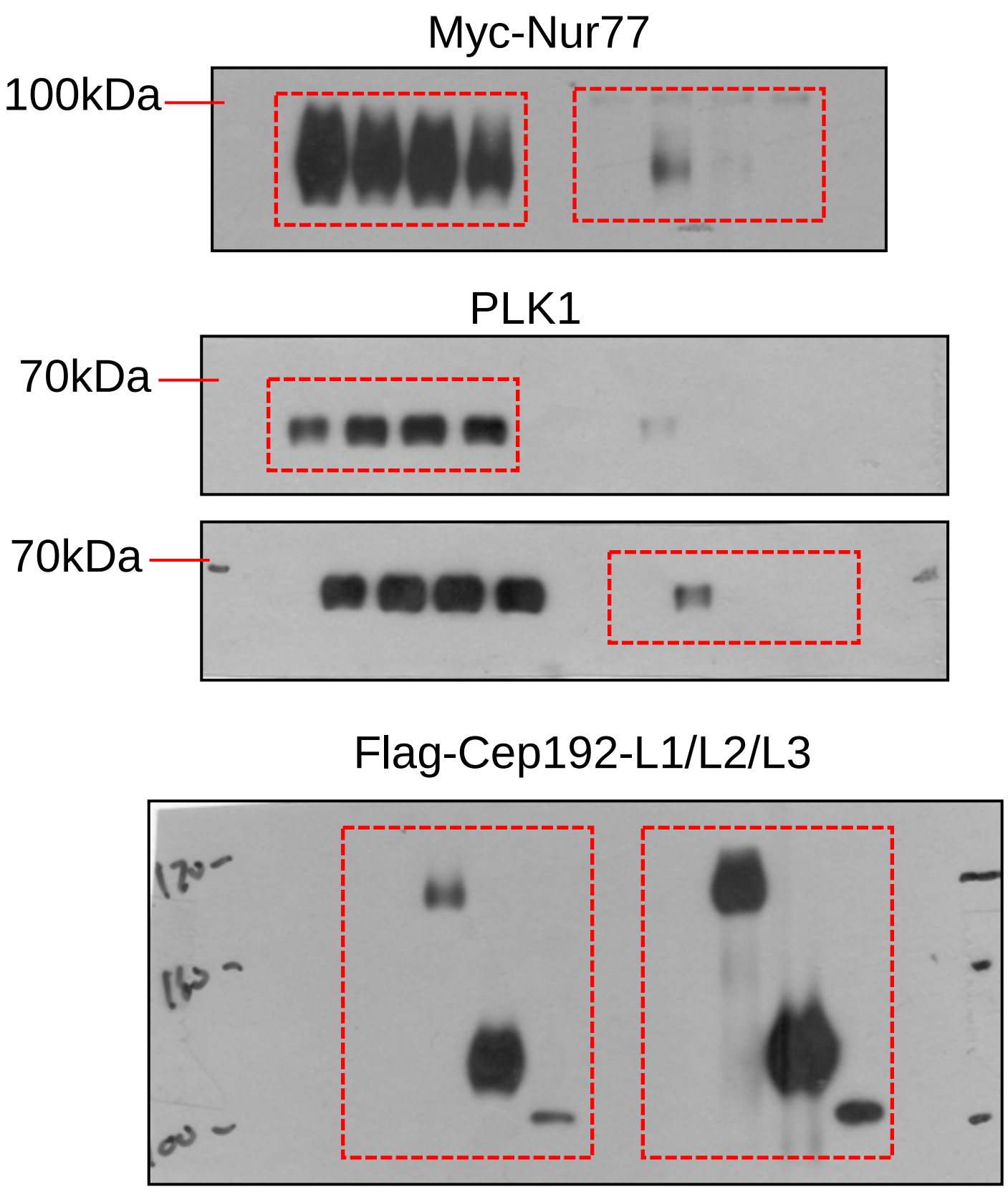

Fig.3I

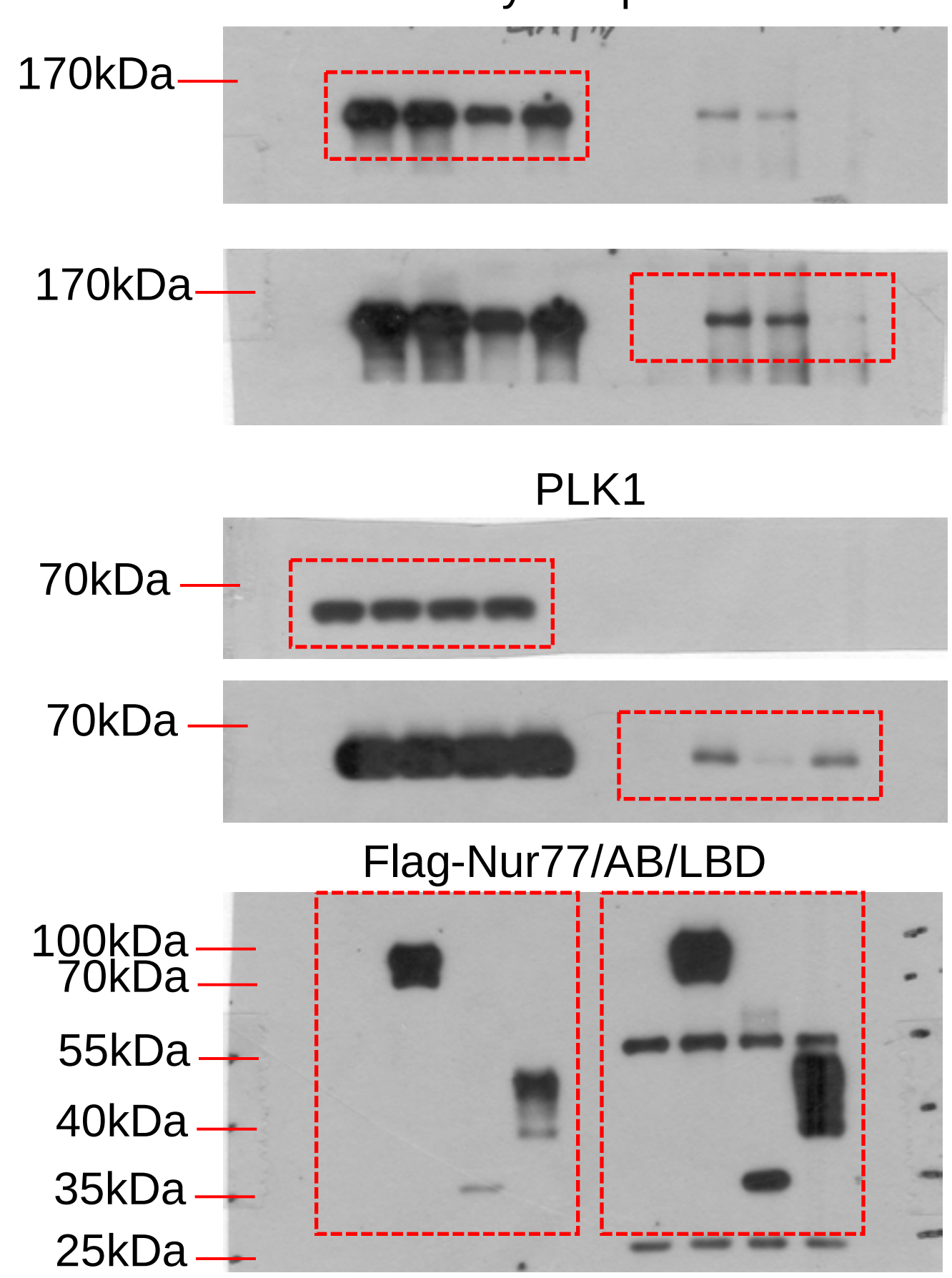

Fig.4I

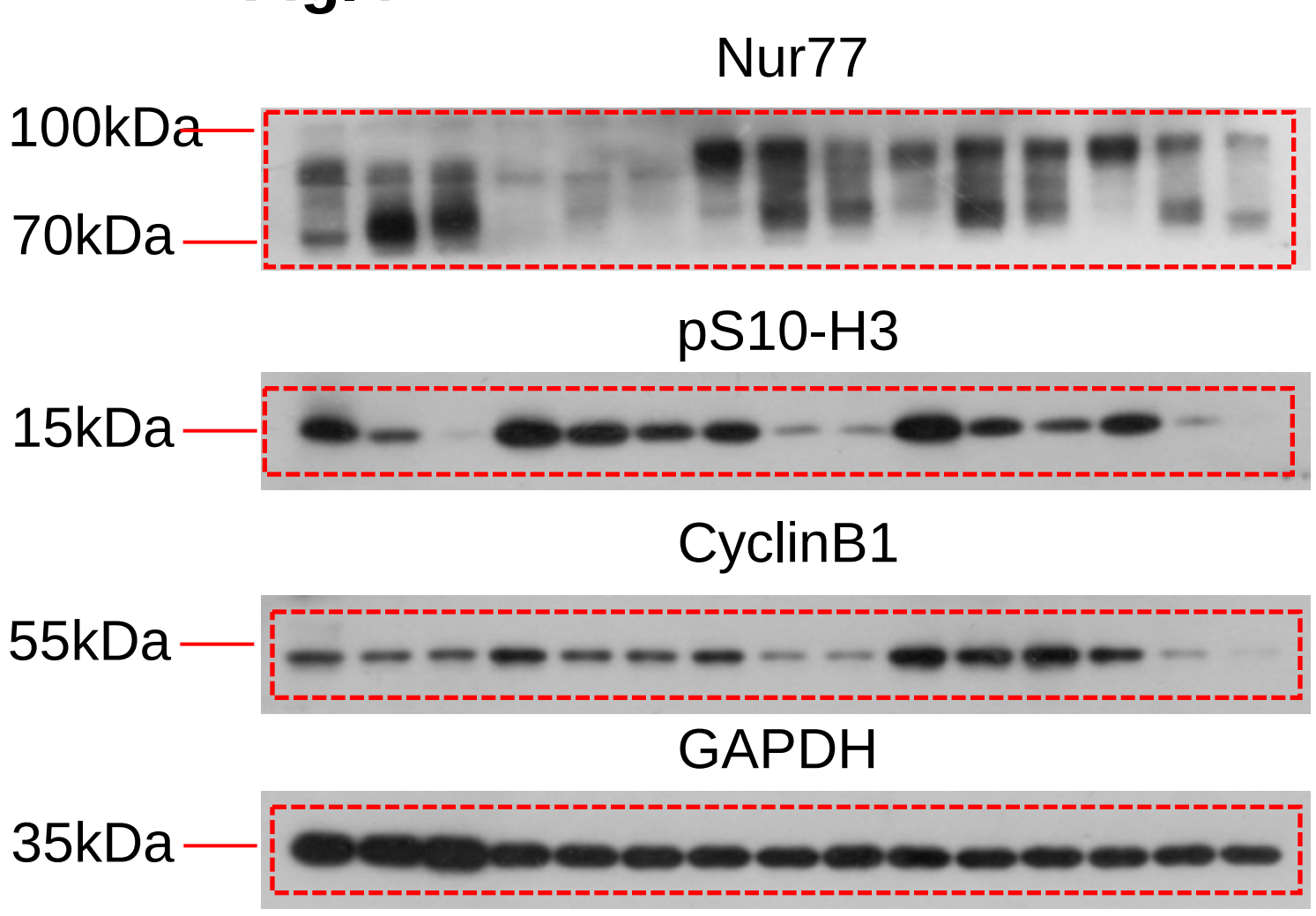

Fig. 5A

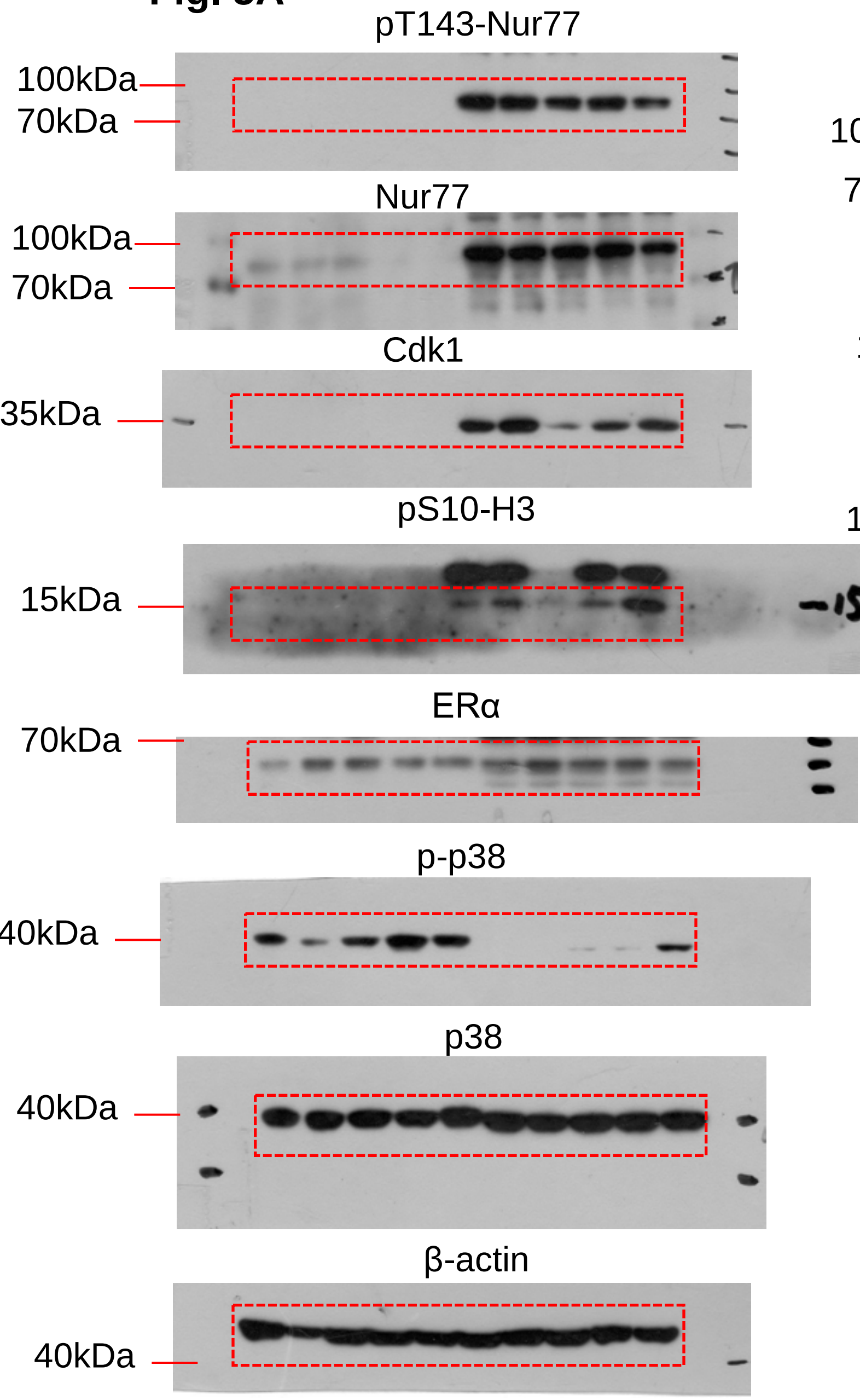

Fig.5E

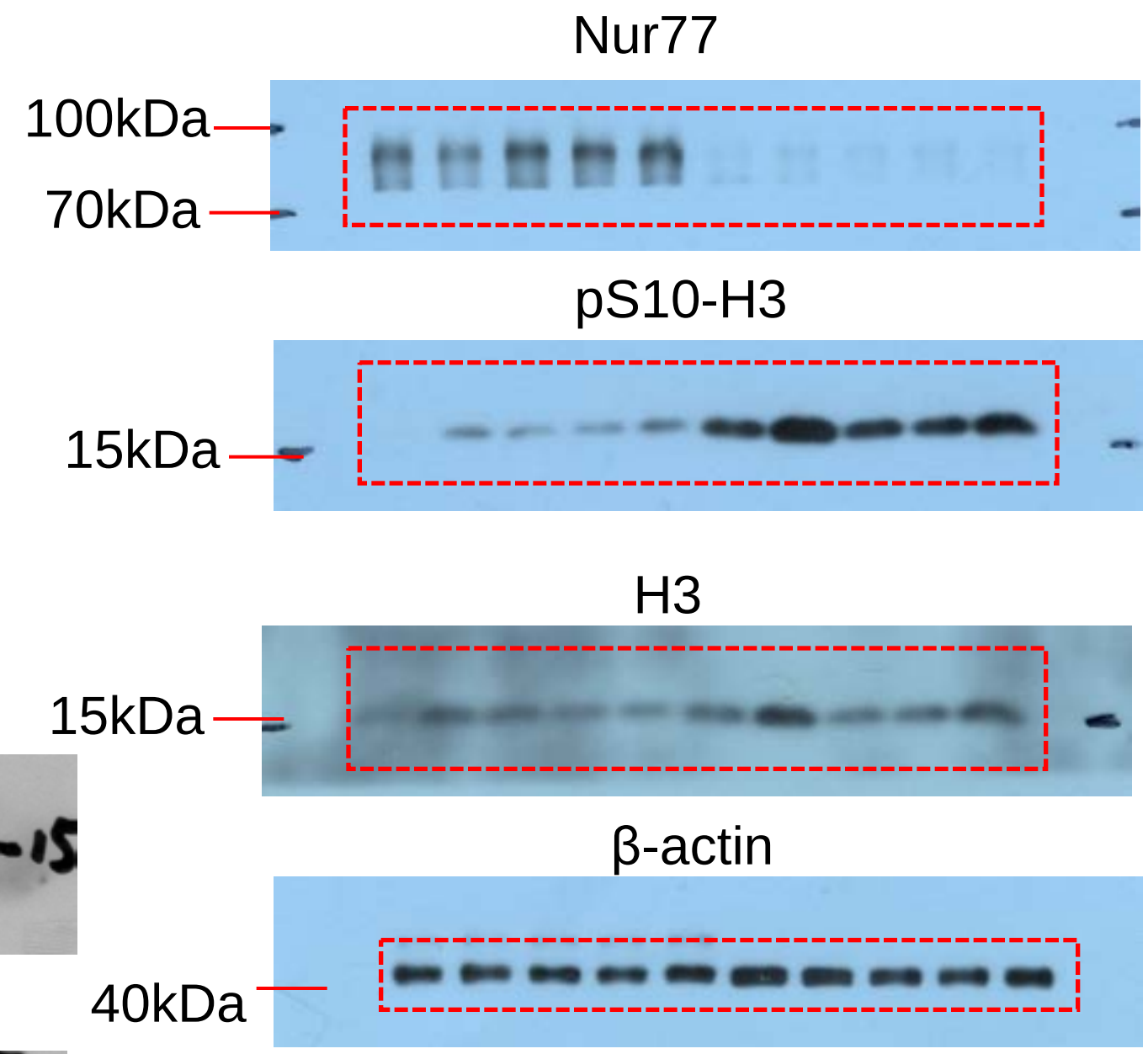

Fig.5L

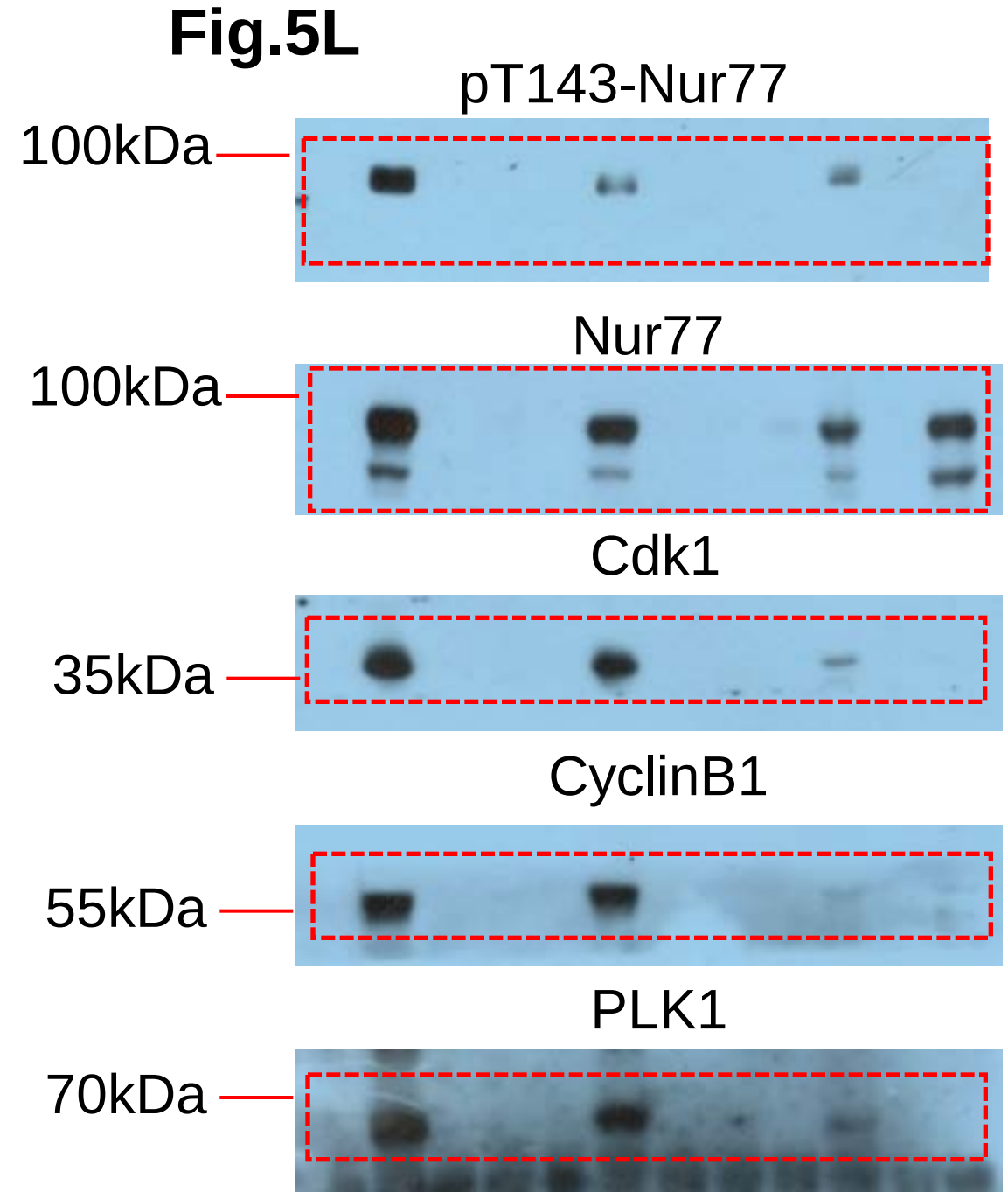

Fig.6C

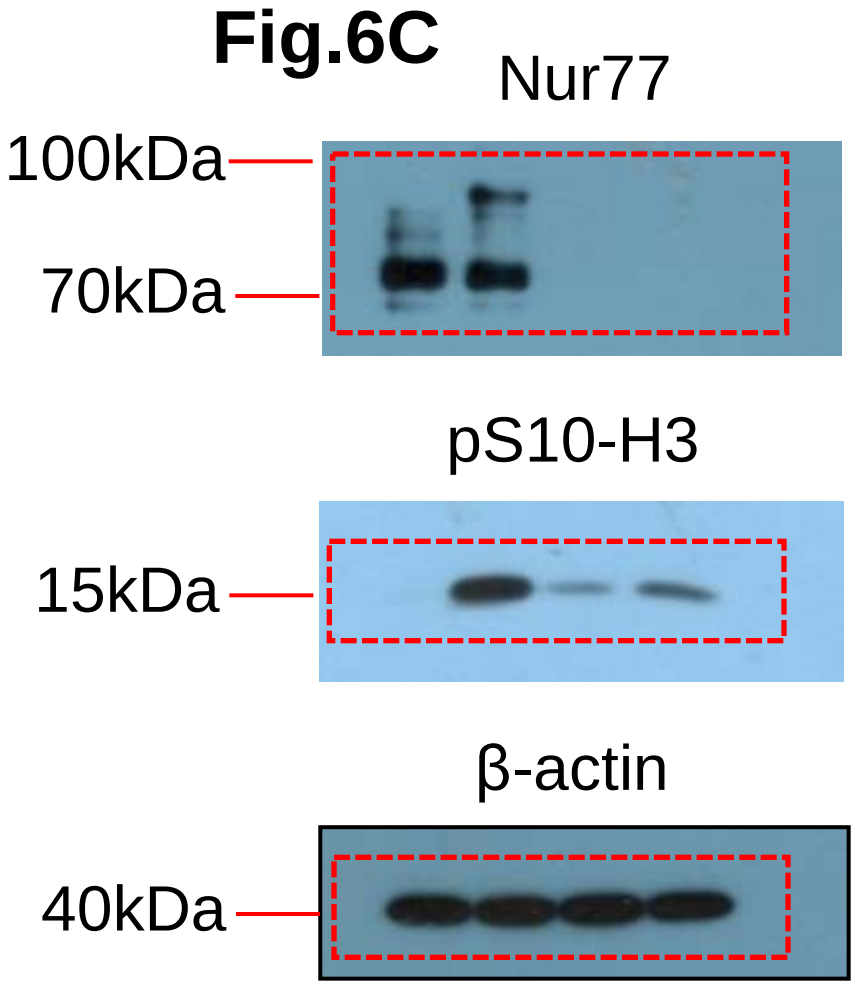

Fig.6D

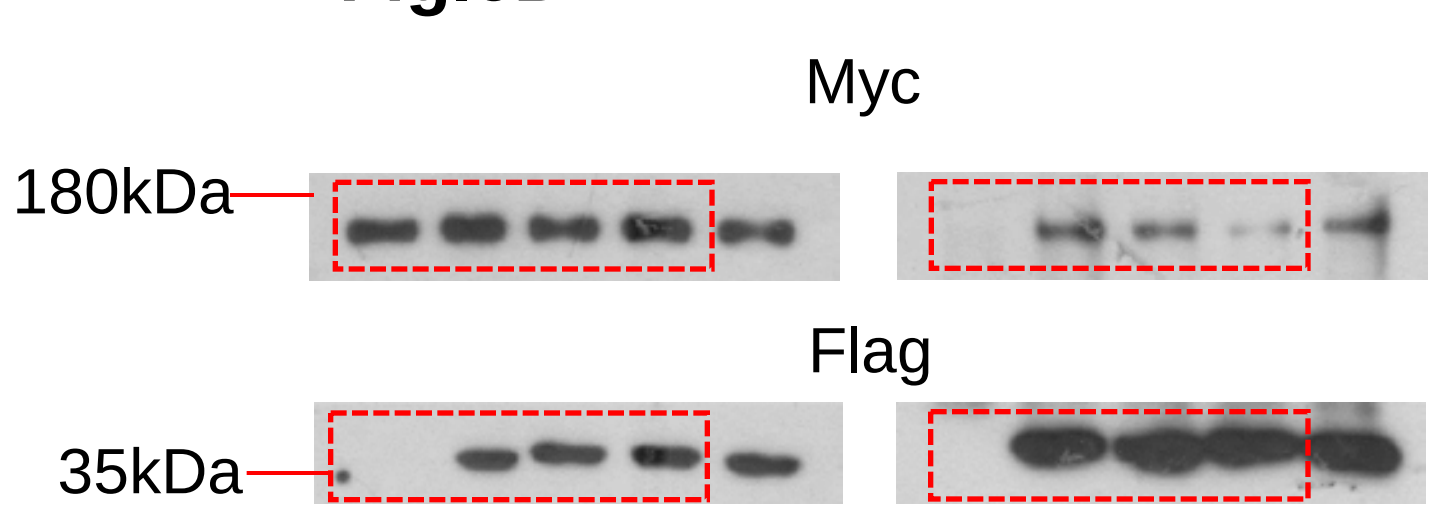

Fig.6M

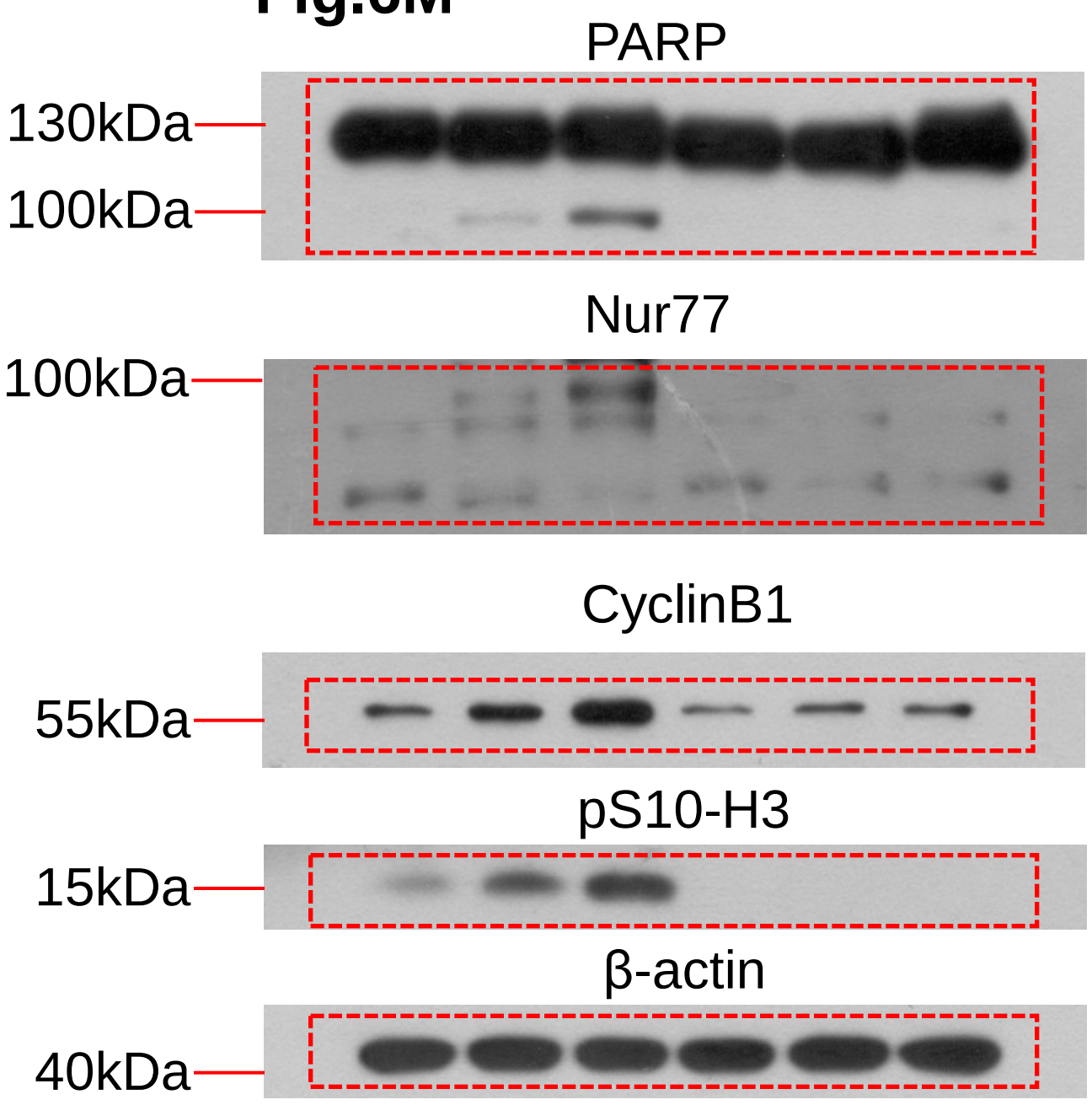

Fig.6Q

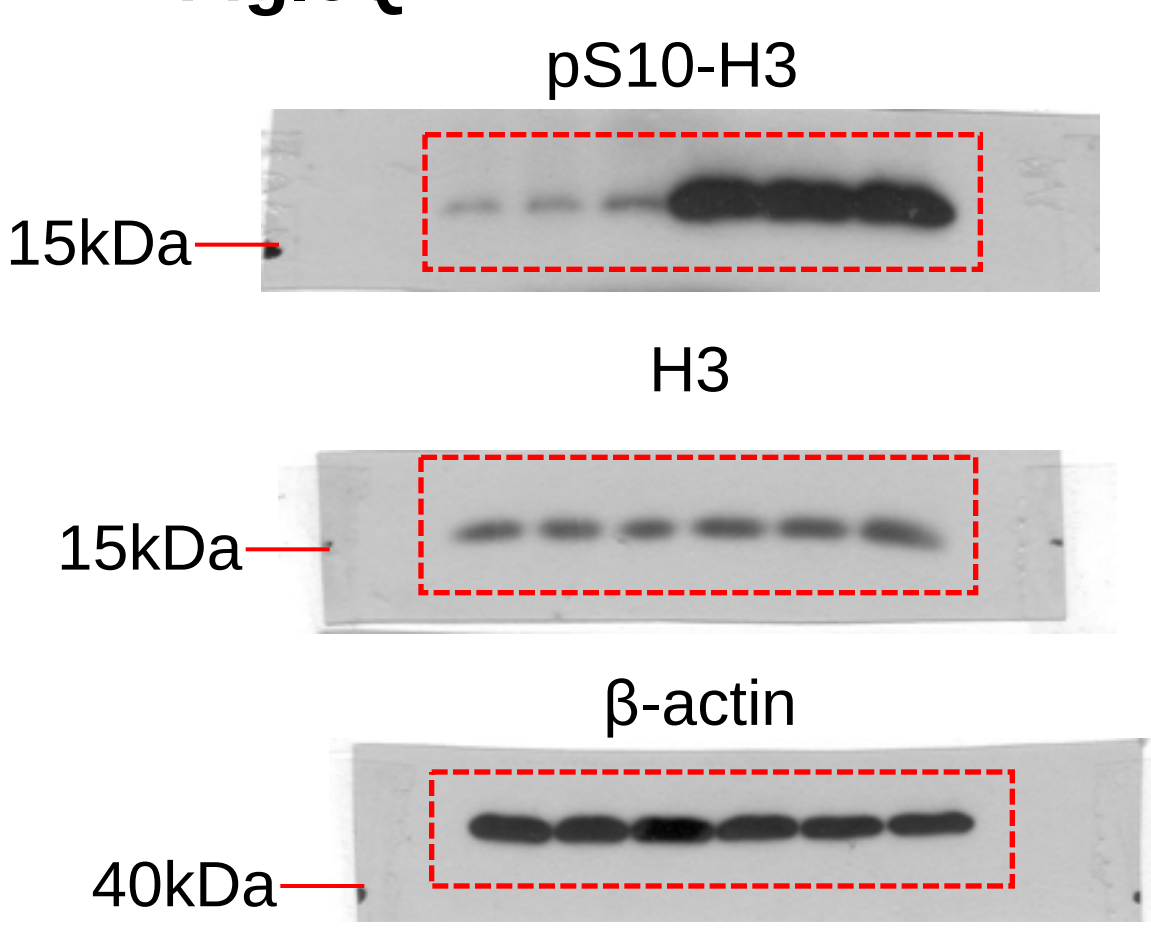

Fig. S1B

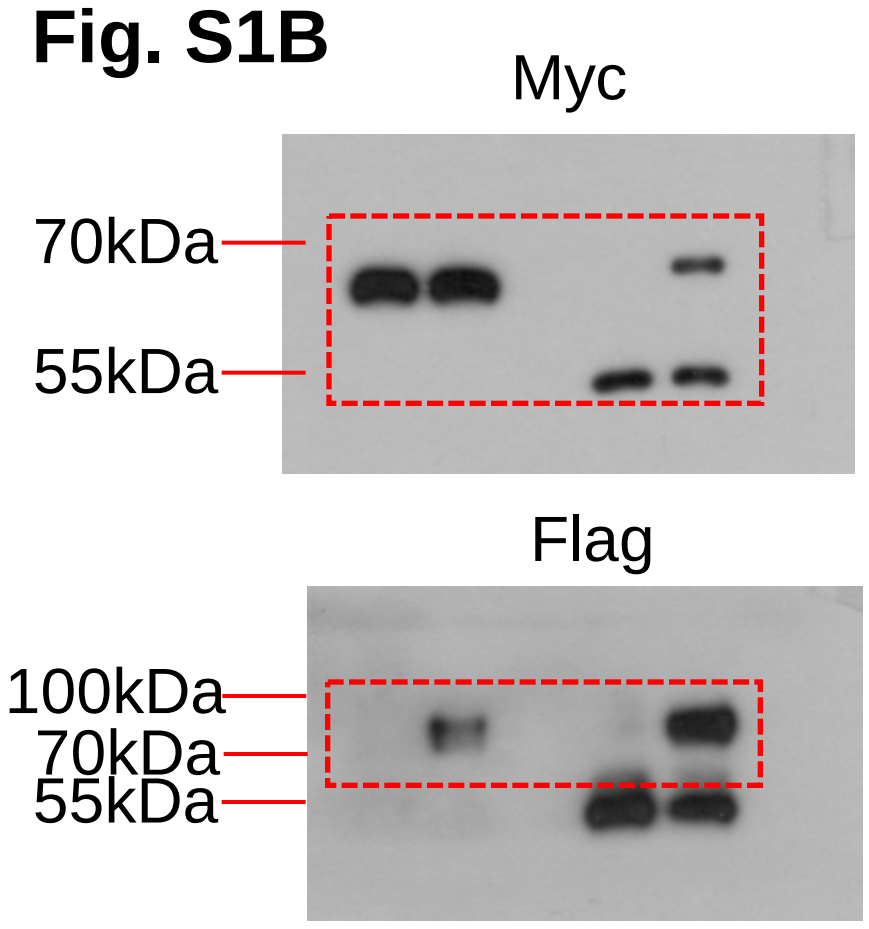

Fig.6N

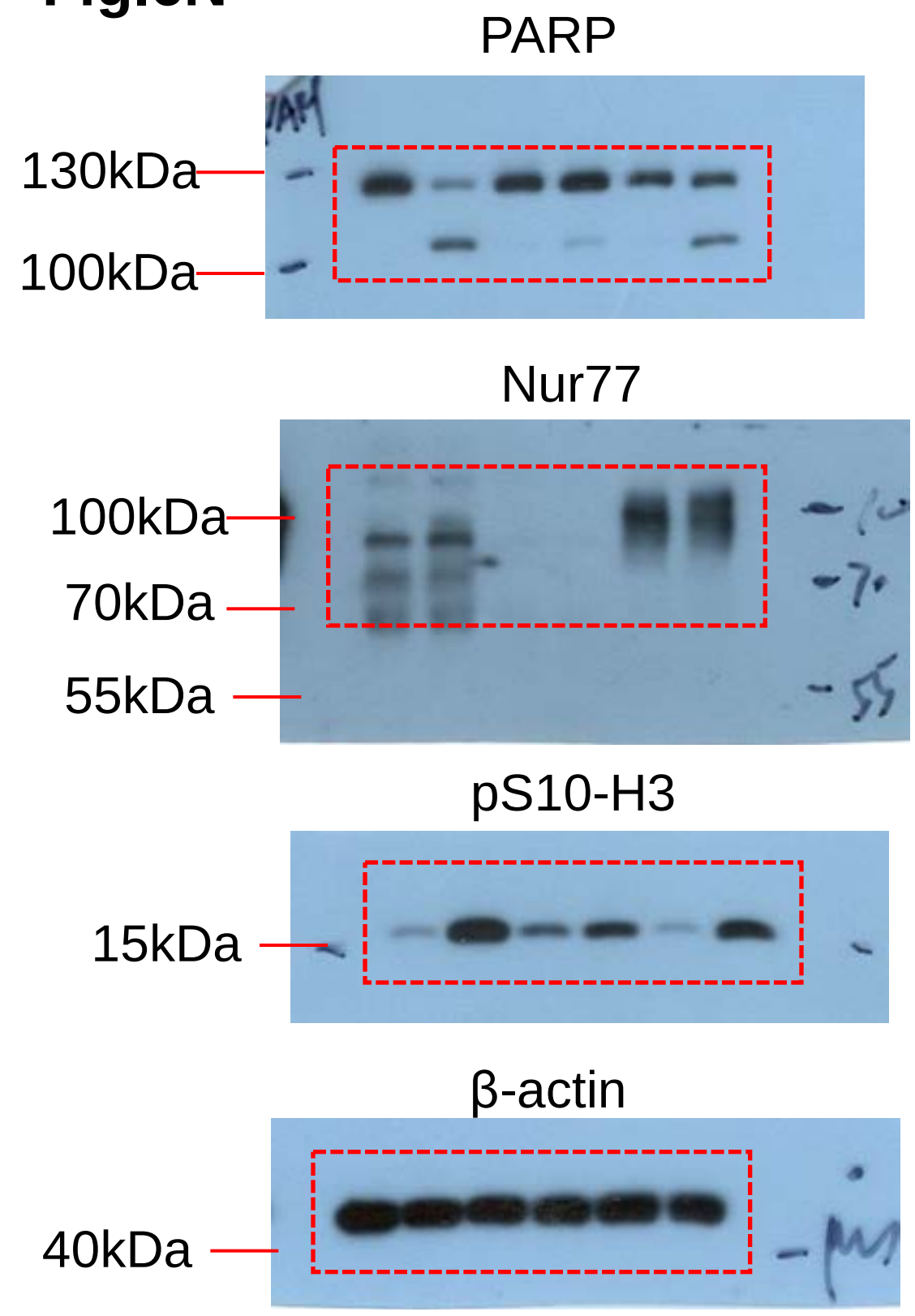

Fig. S1A

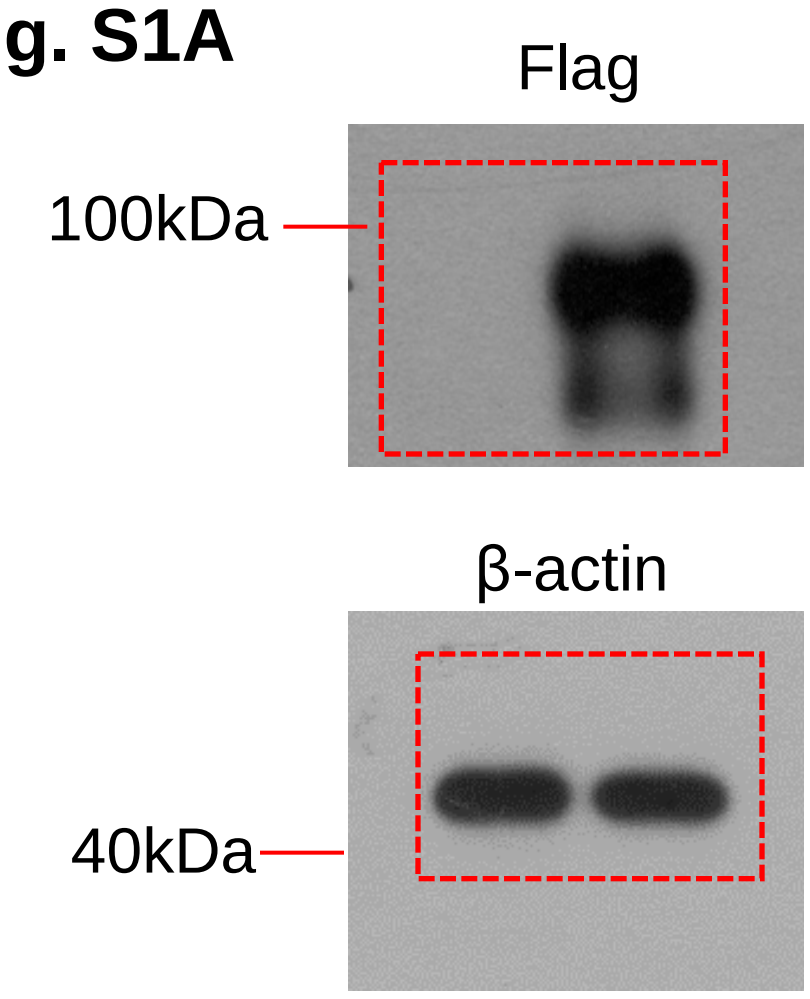

Fig. S1D

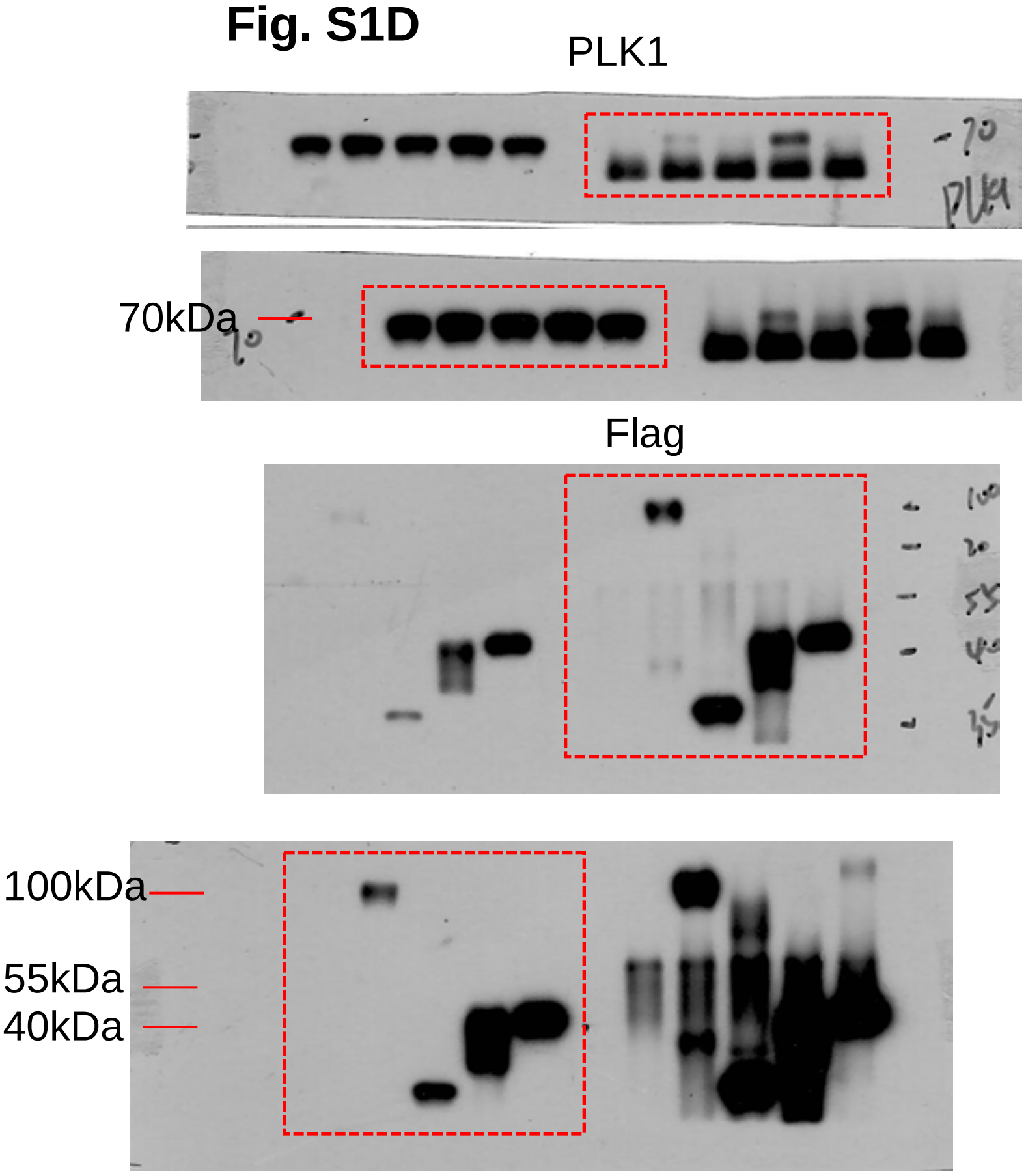

Fig. S1E

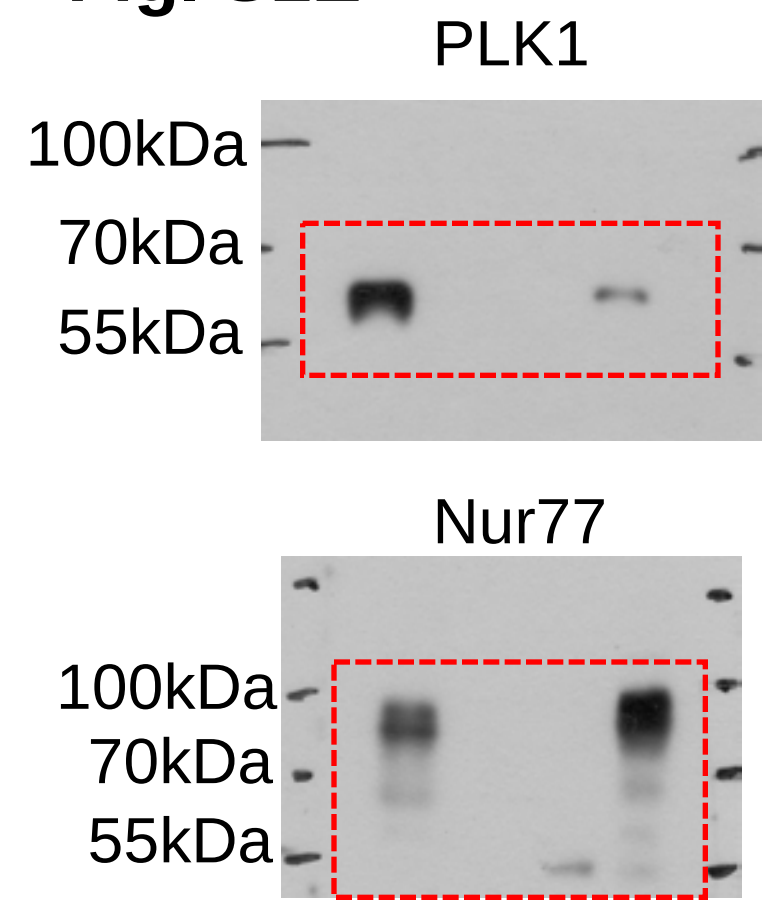

Fig. S1F

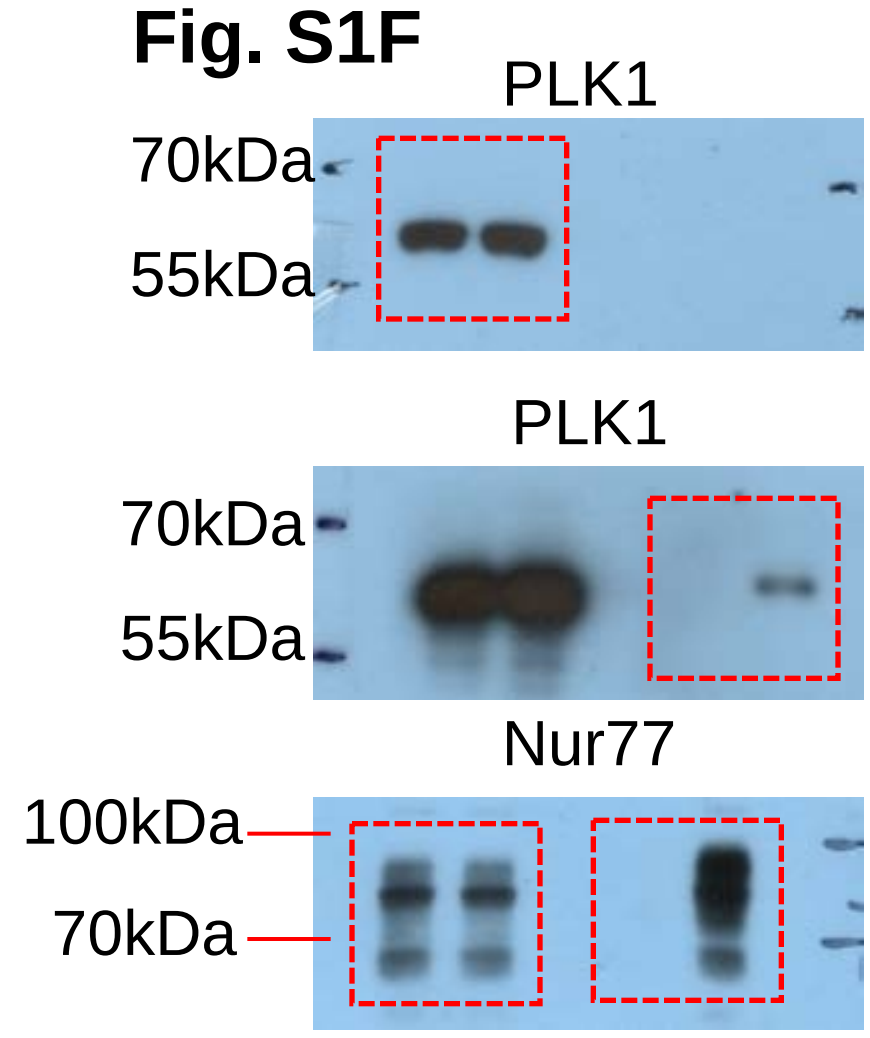

Fig. S1G

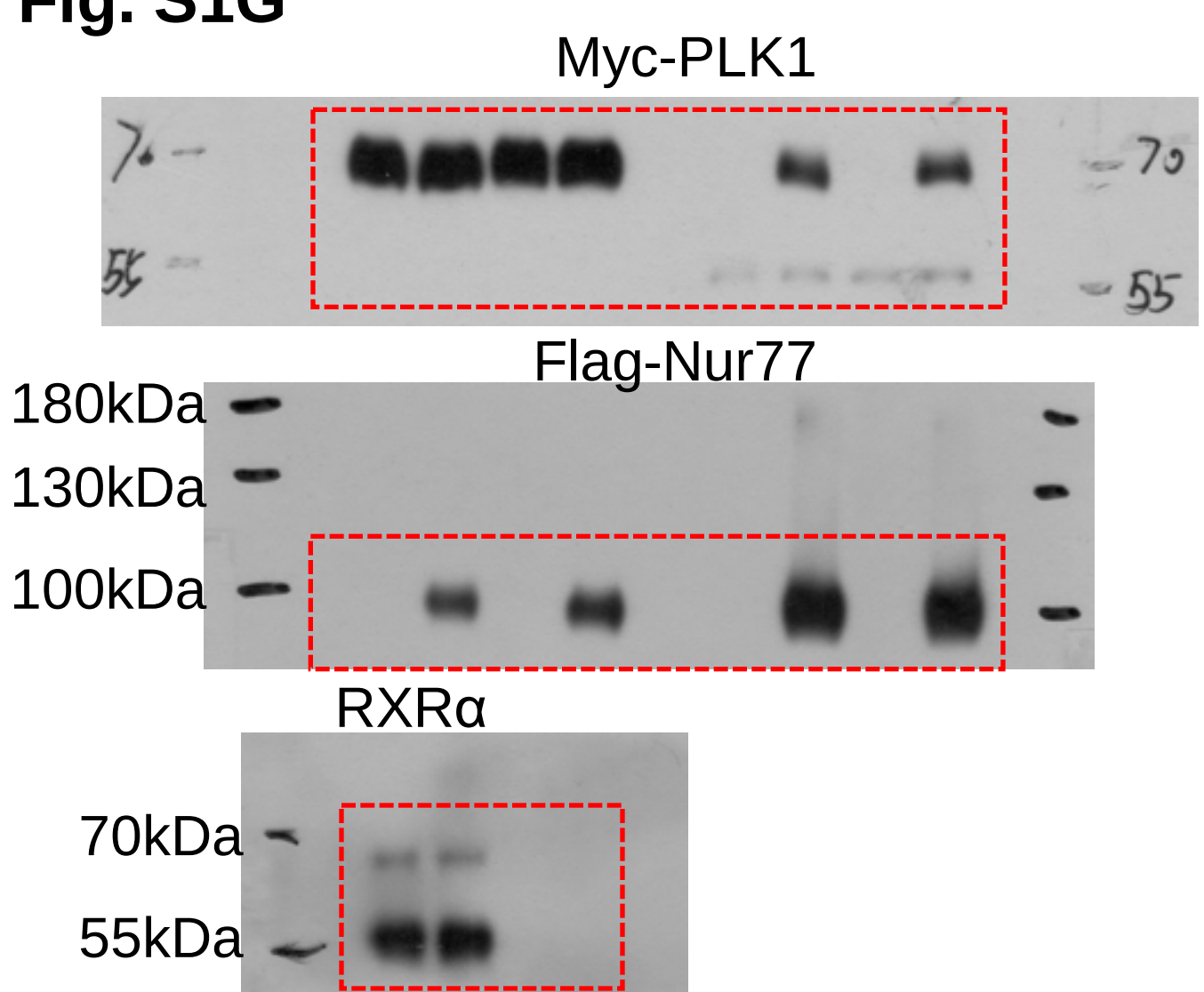

Fig. S2A

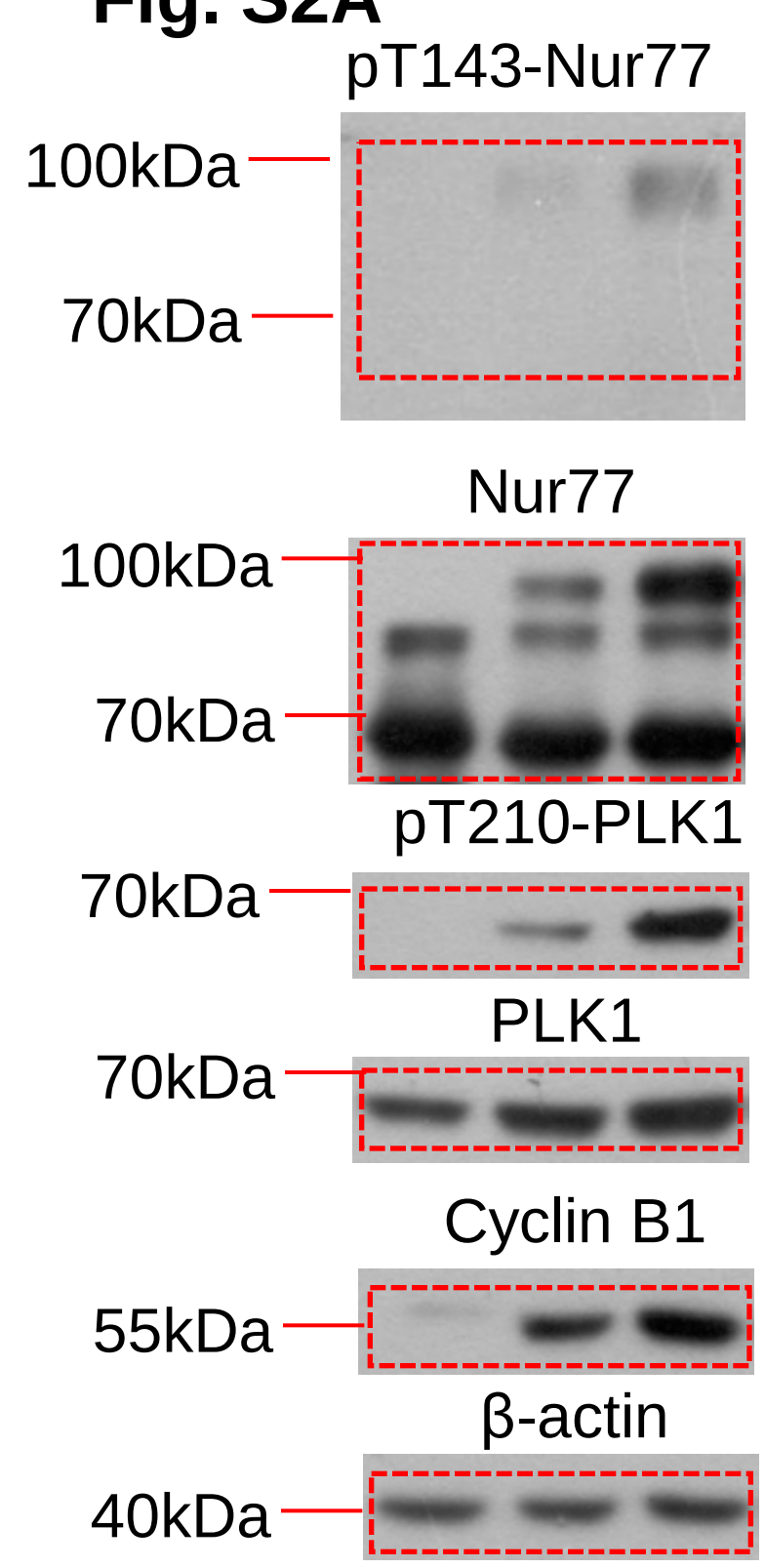

Fig. S2B

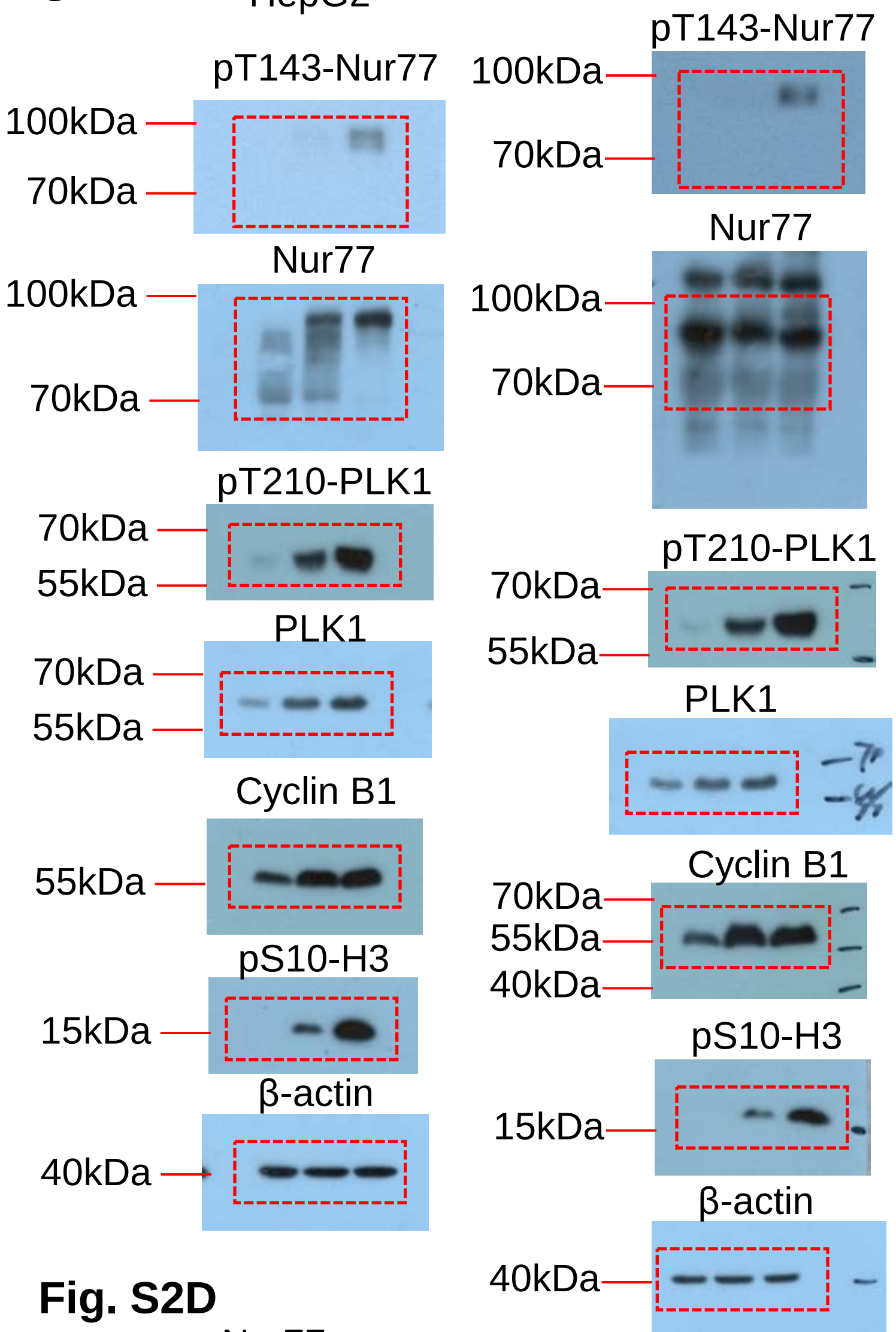

Fig. S2C

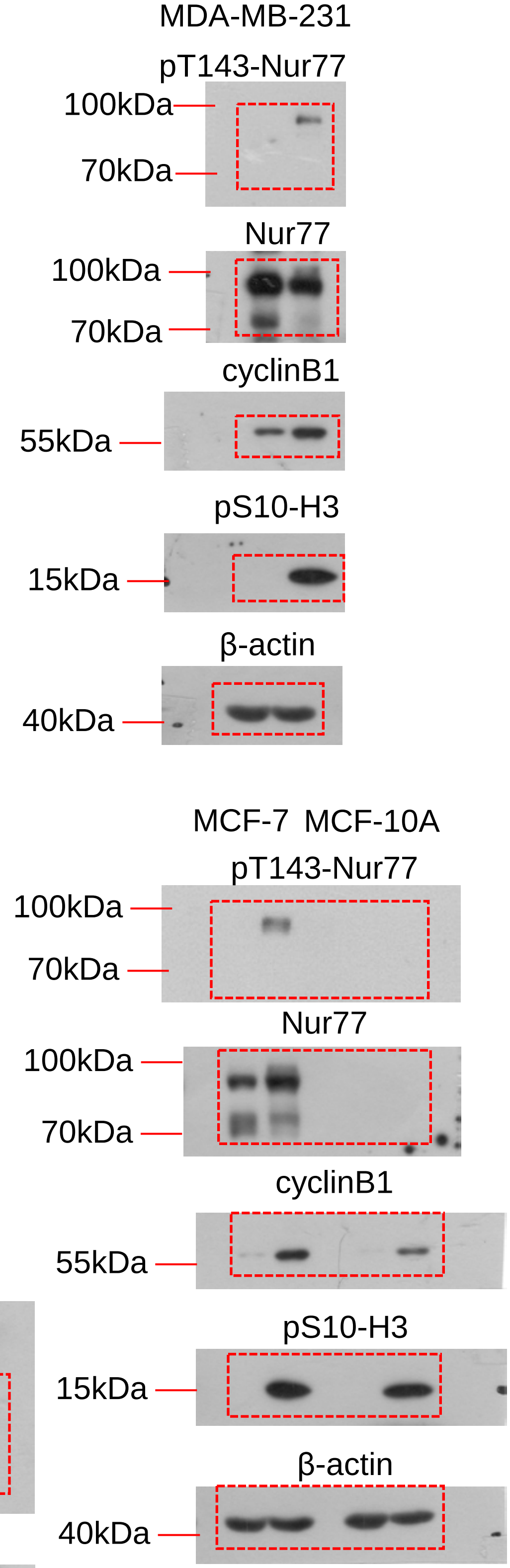

Fig. S2D

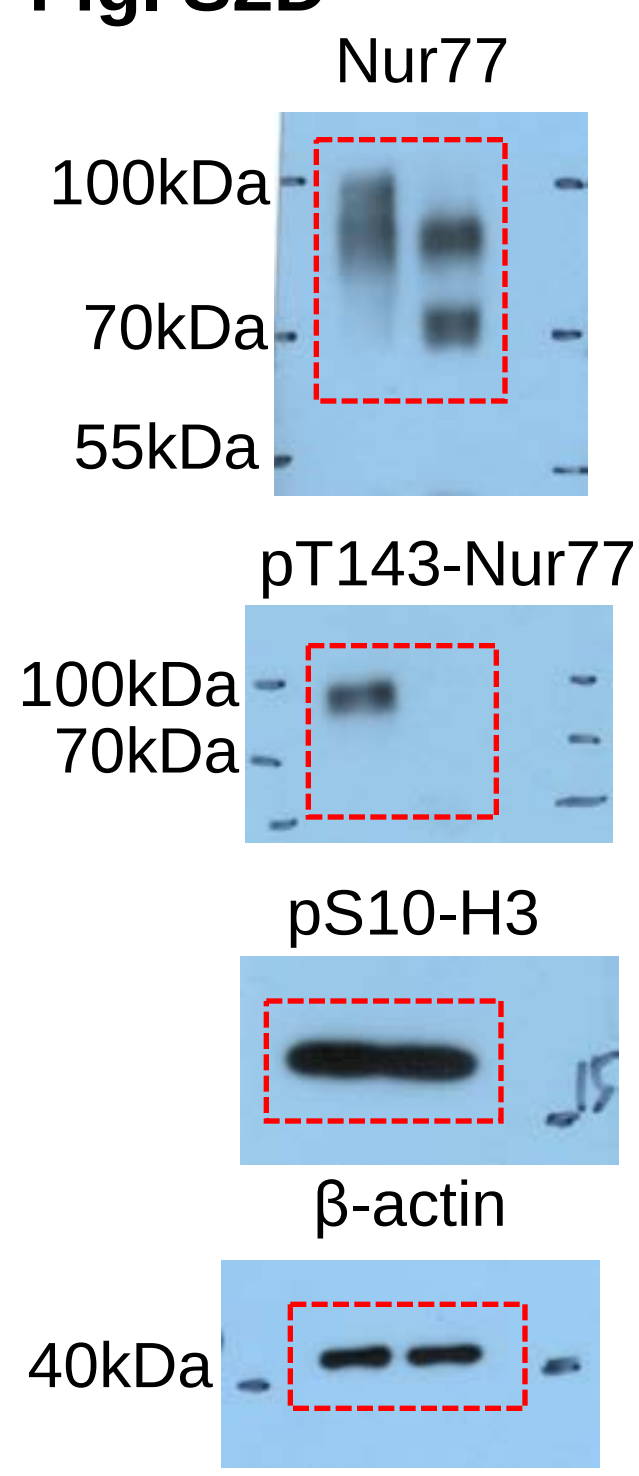

Fig. S2E

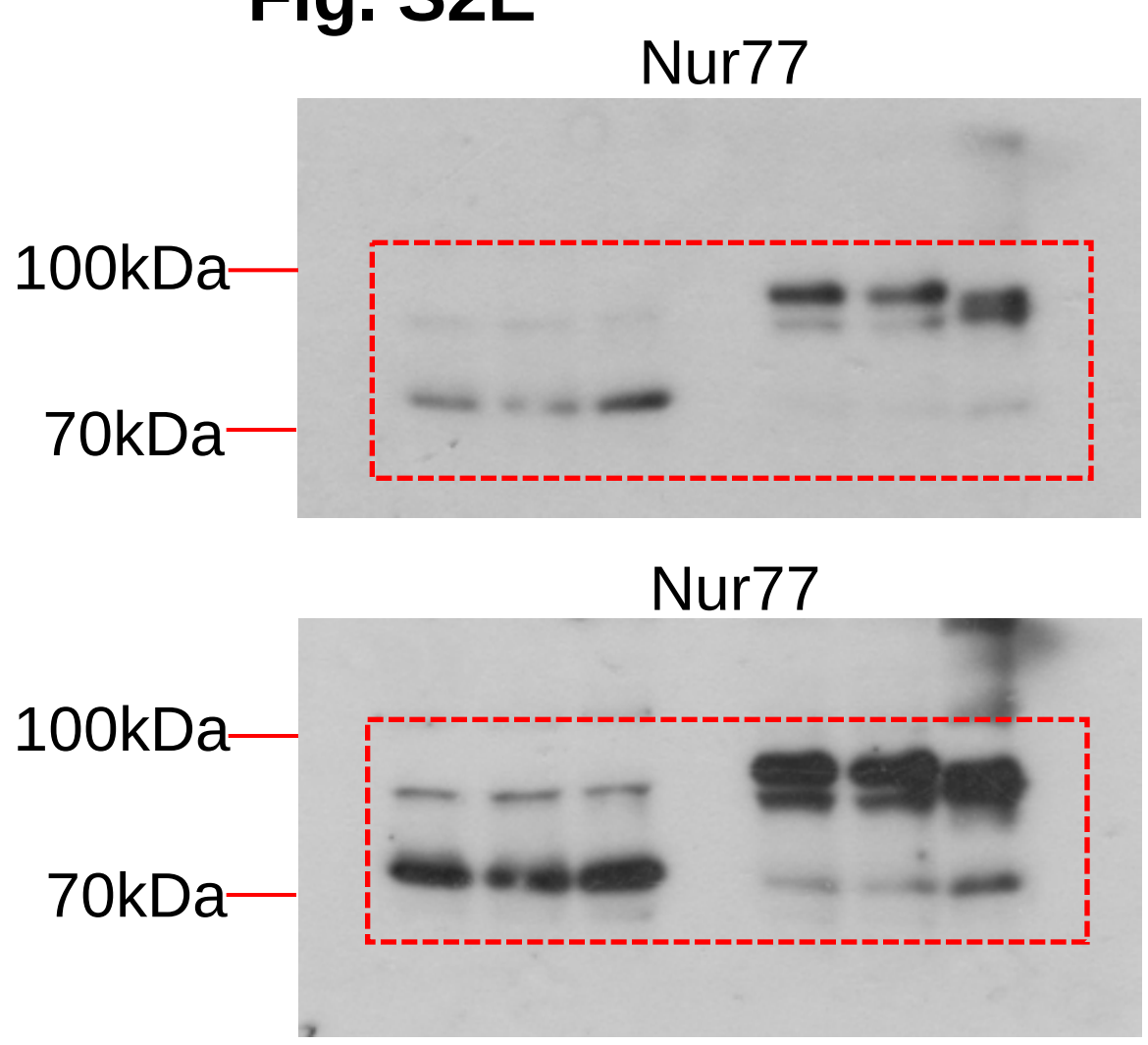

Fig. S2F

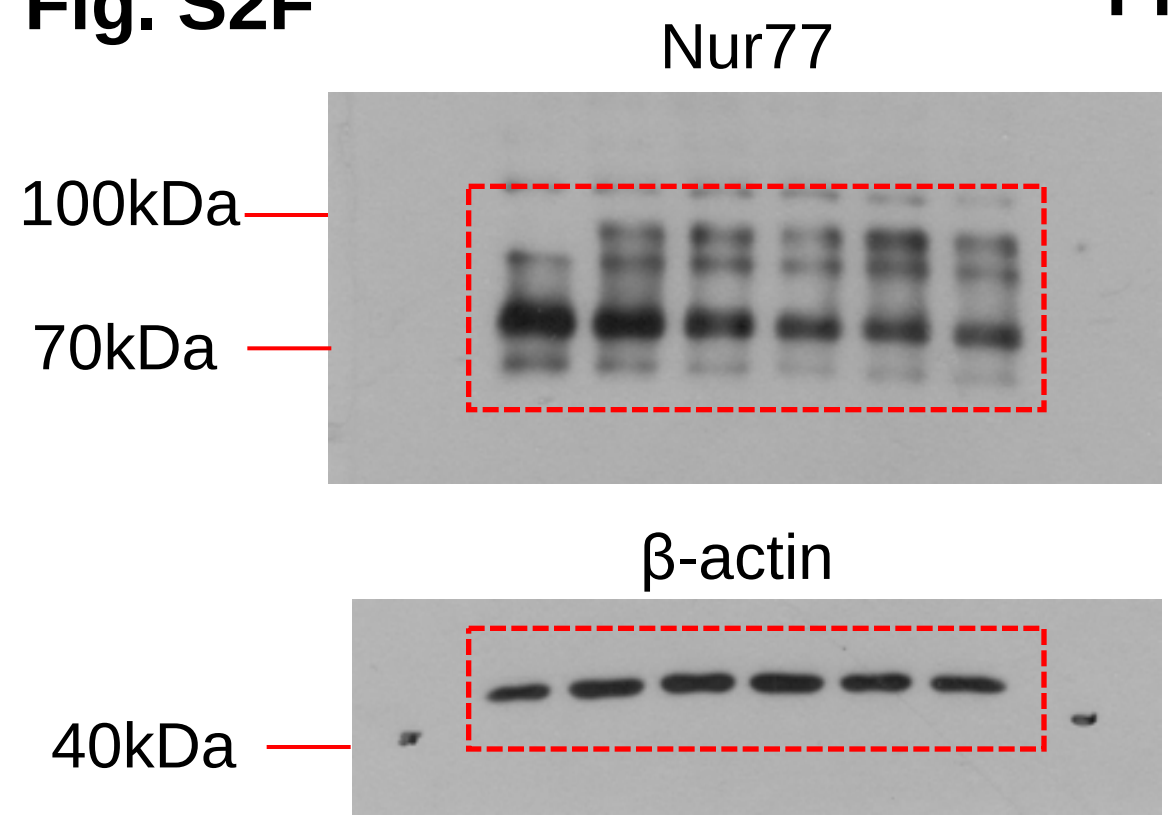

Fig. S2G

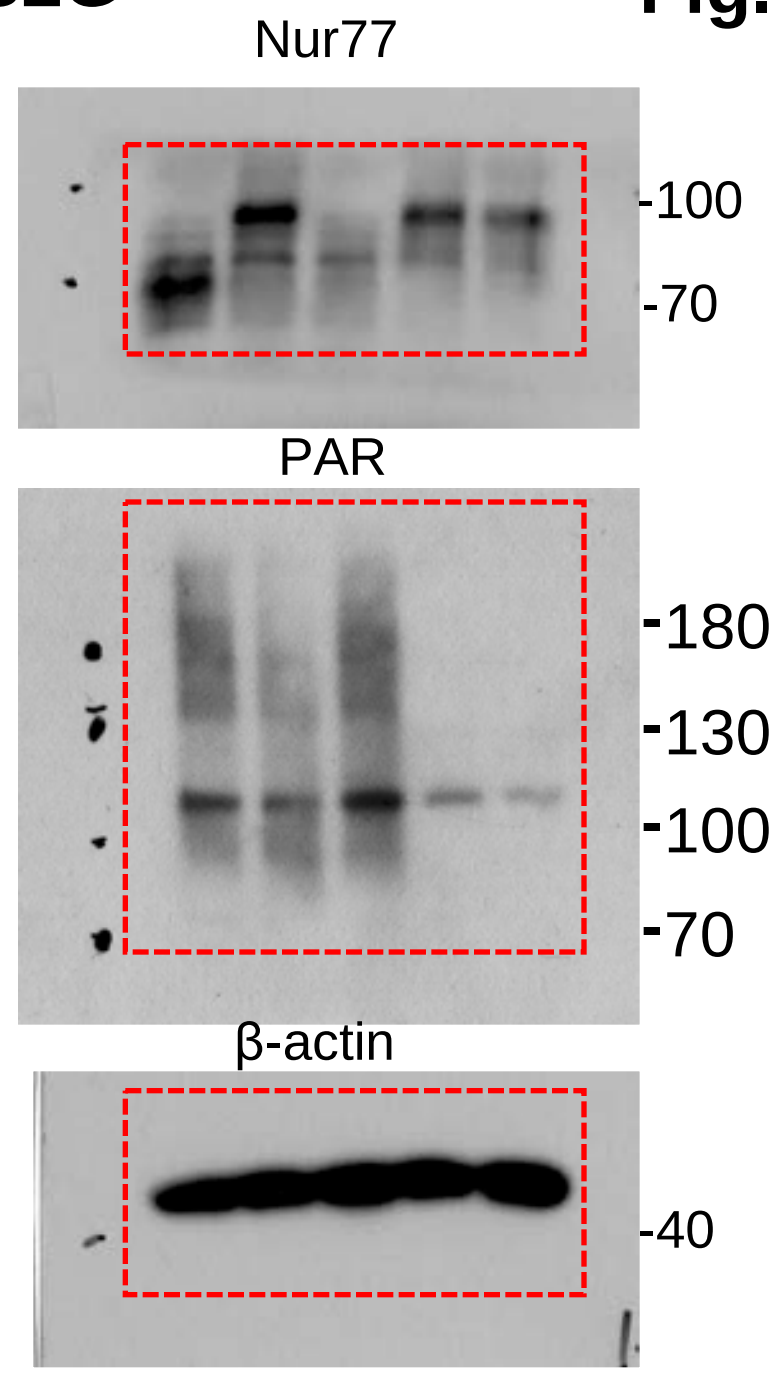

Fig. S2H

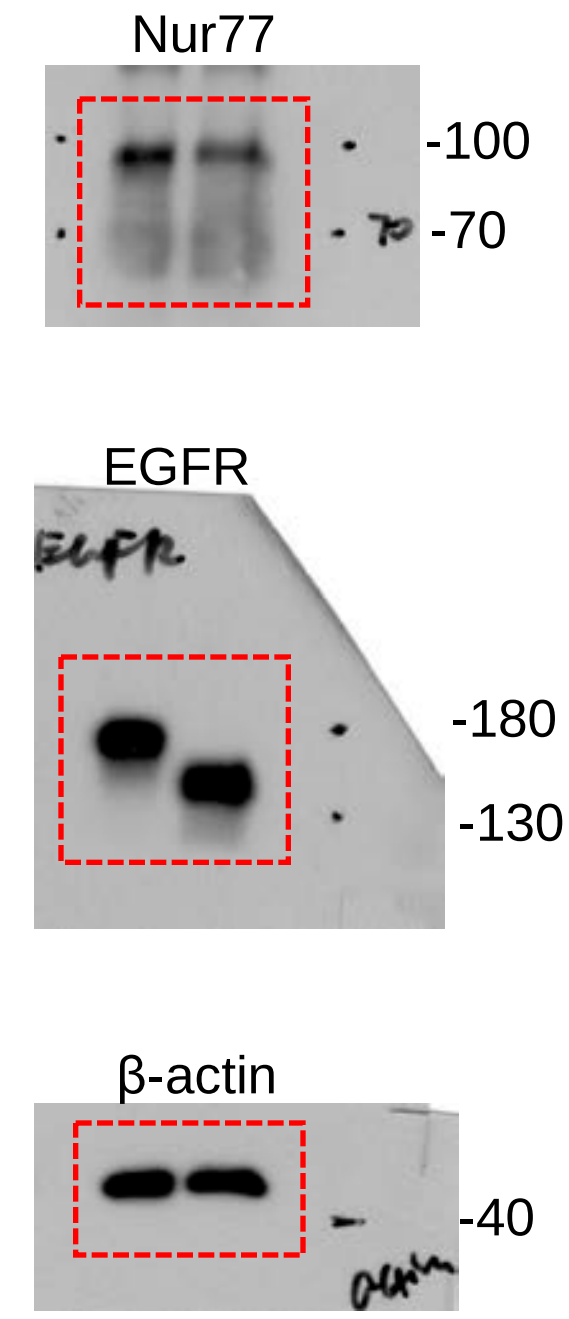

Fig. S2I

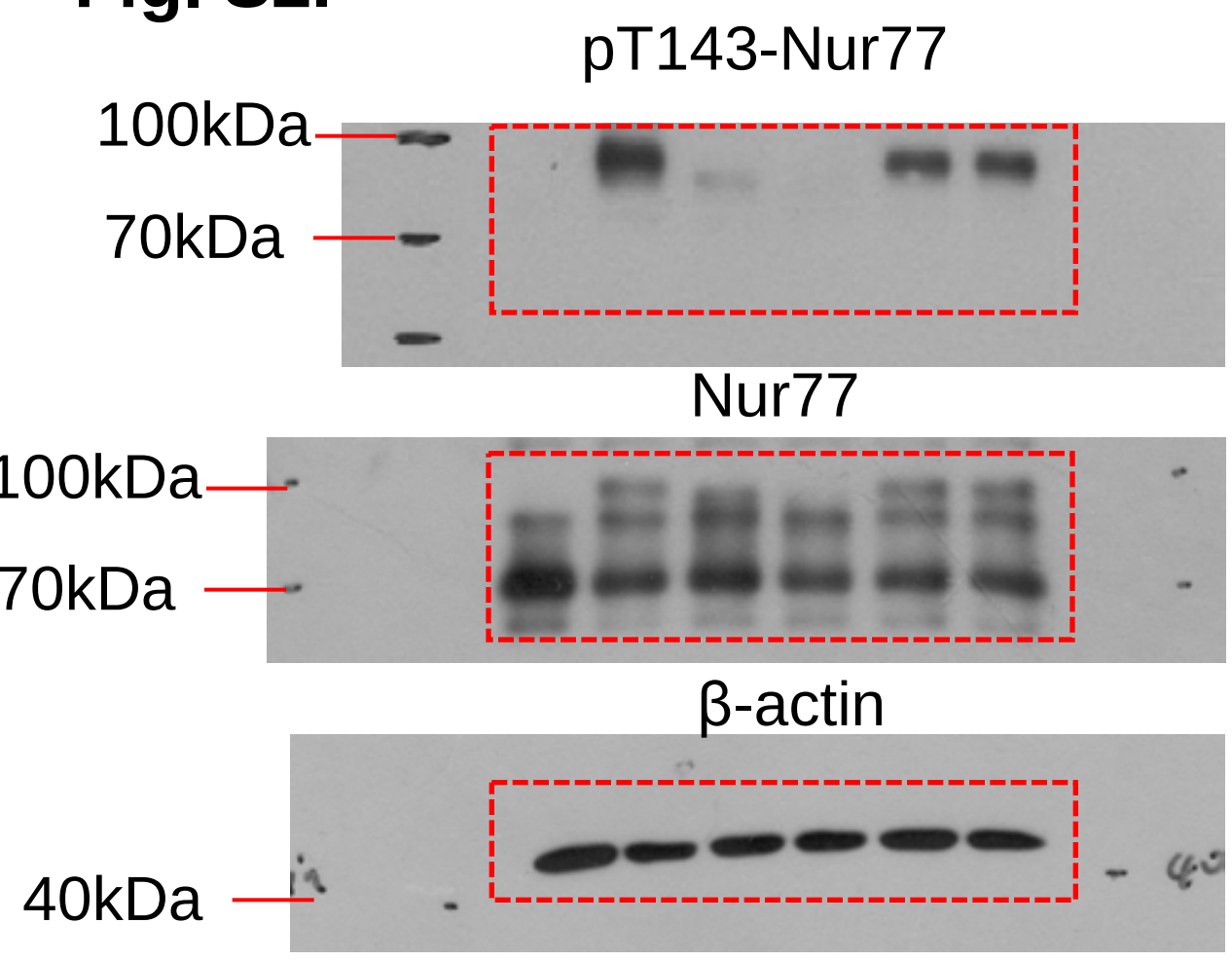

Fig. S2J

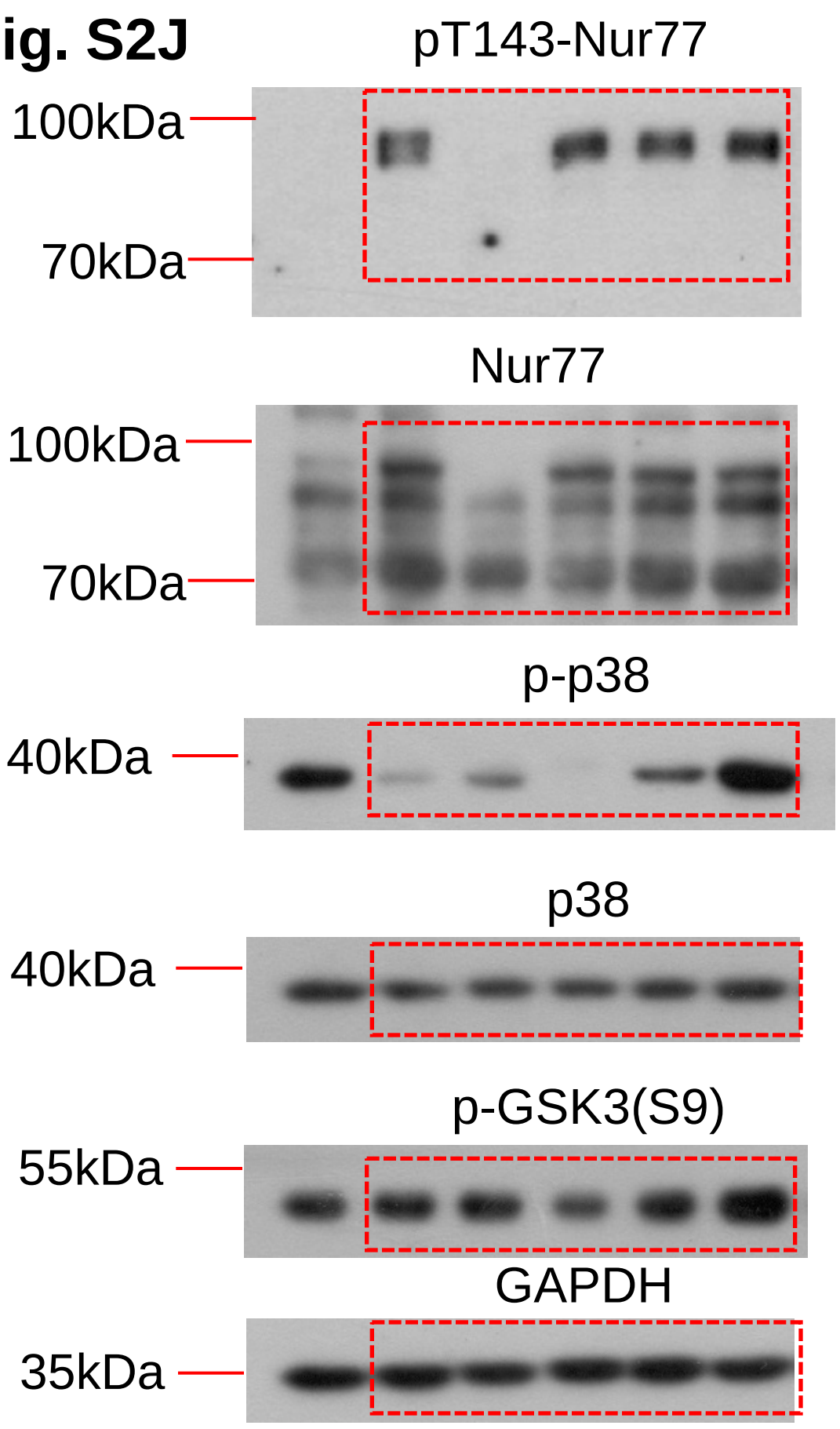

Fig. S2K

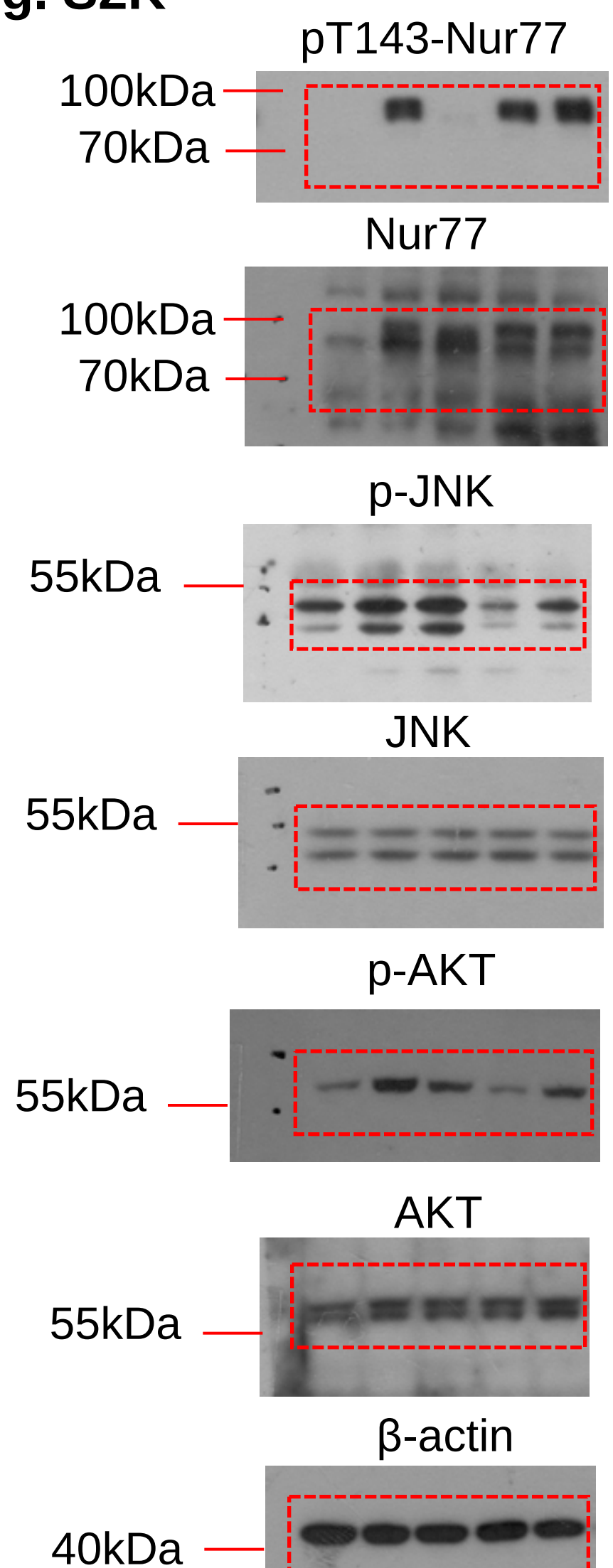

Fig. S3C

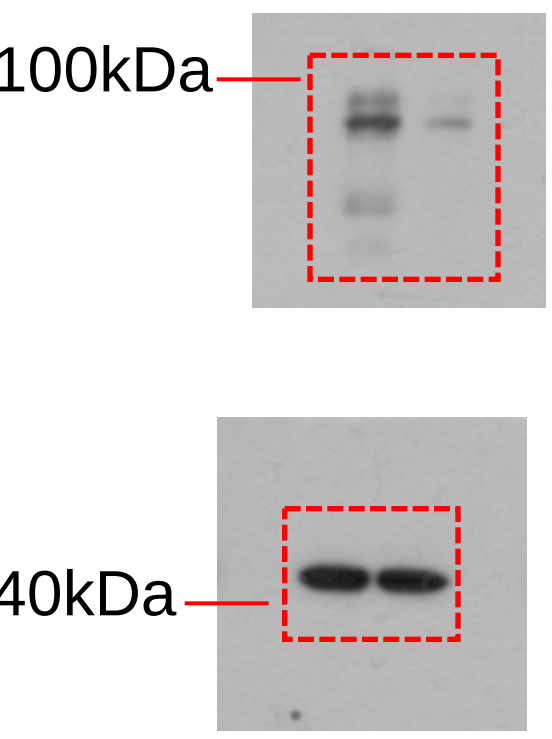

Fig. S3D

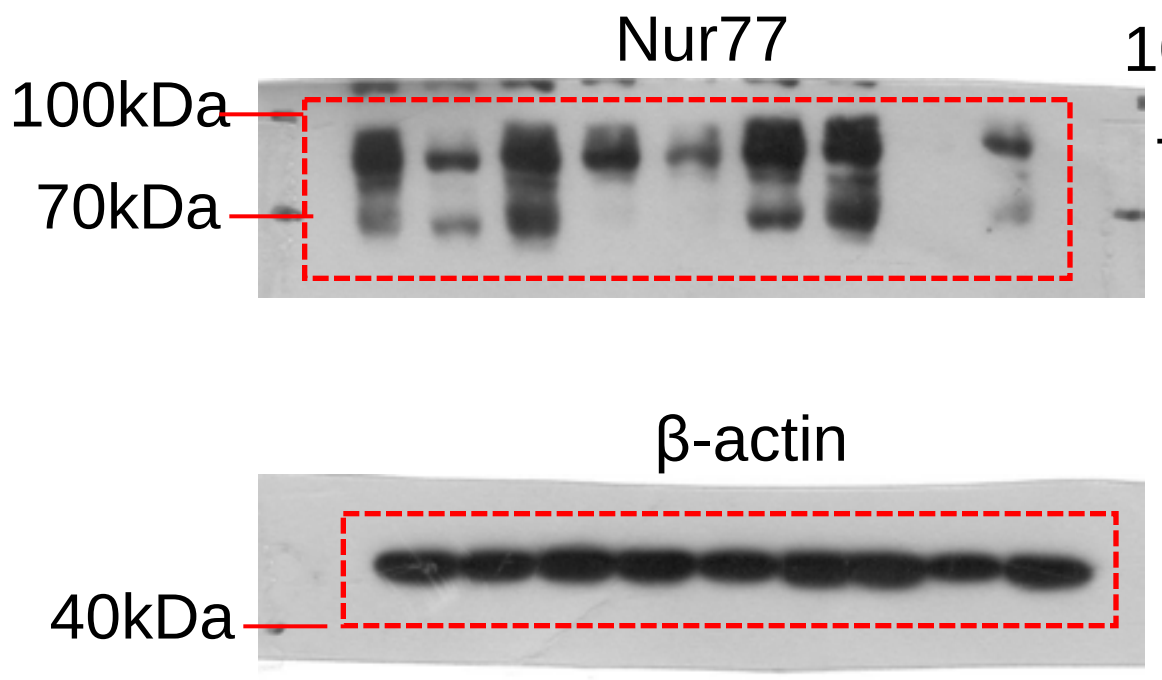

Fig. S3E

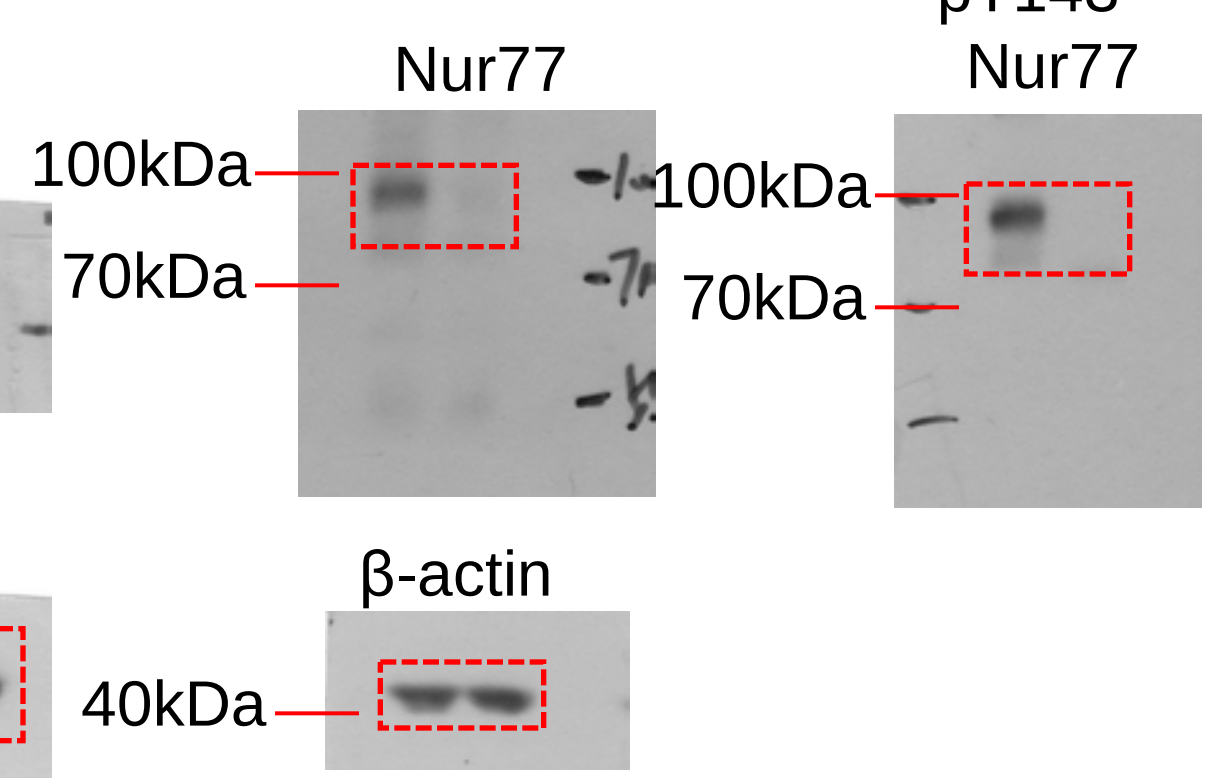

Fig. S3N

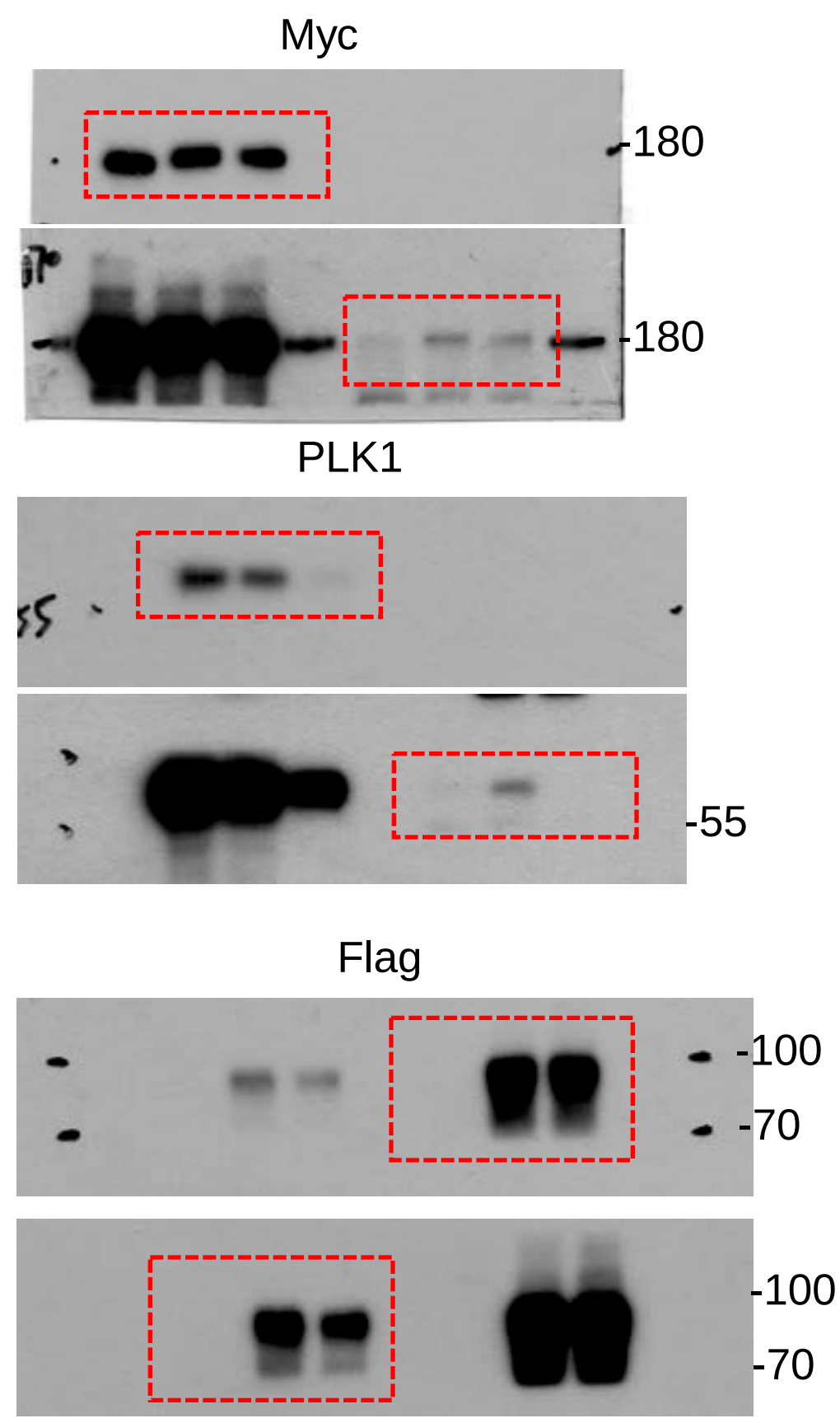

Fig. S3M

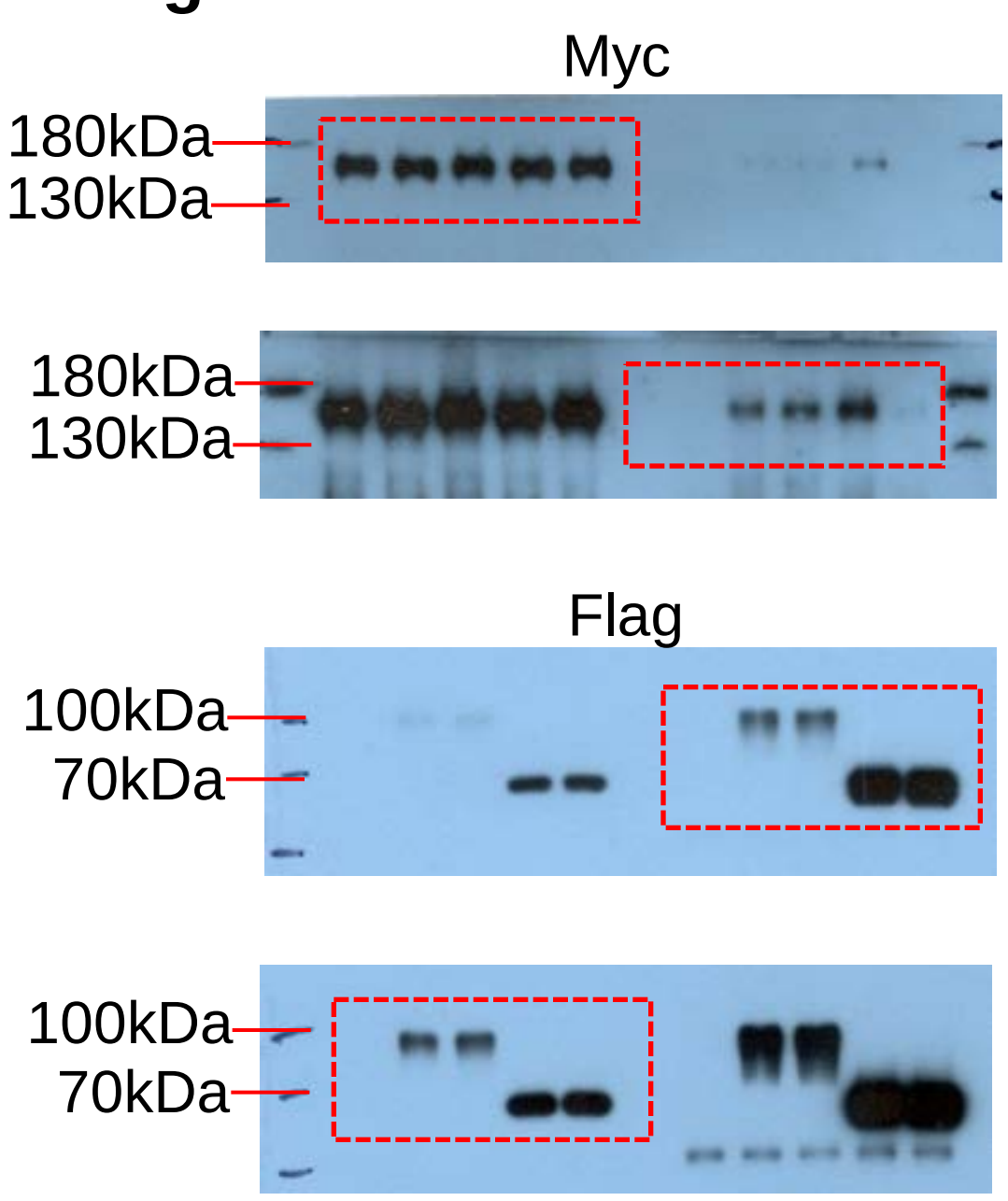

Fig. S3O

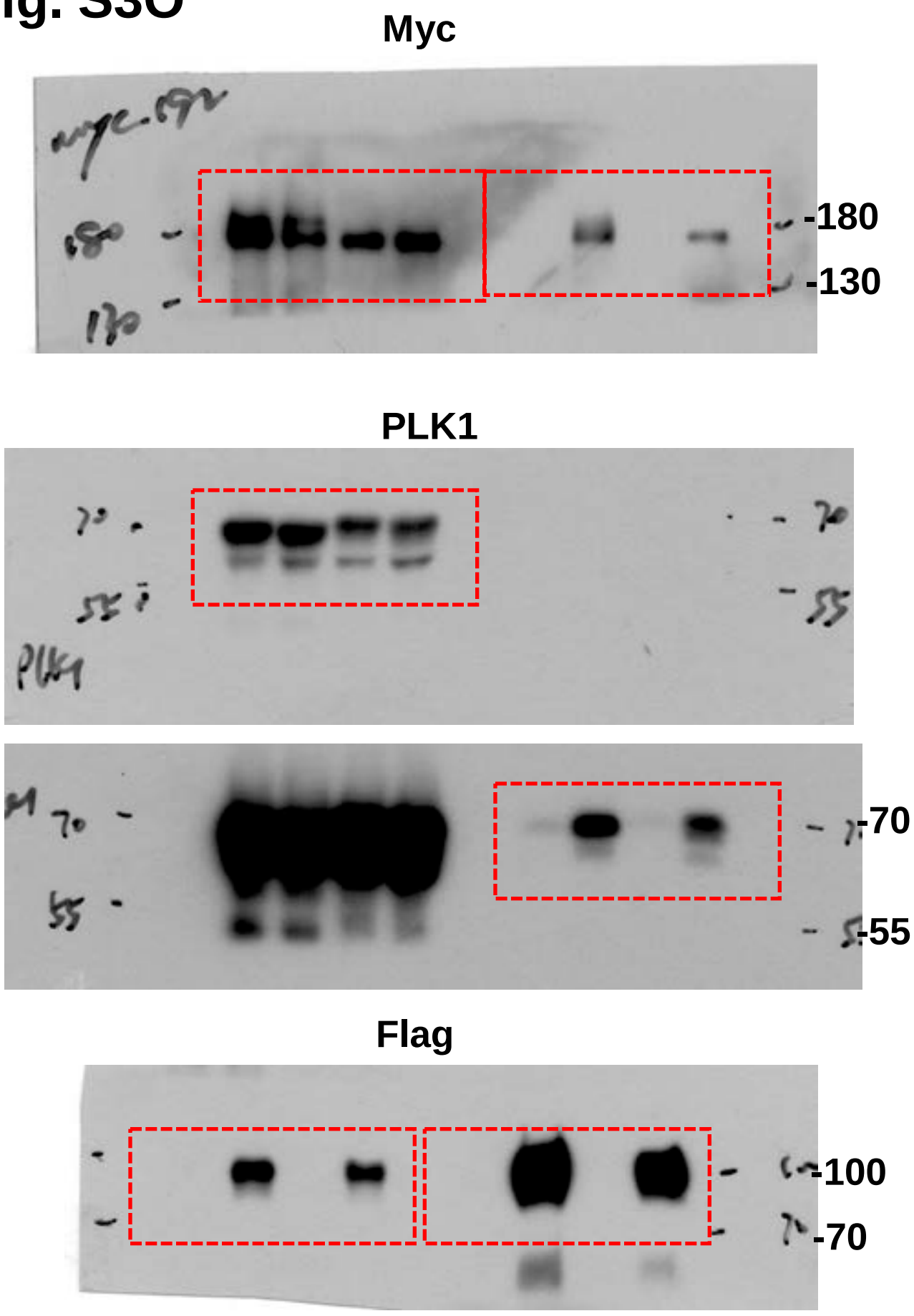

Fig. S3P

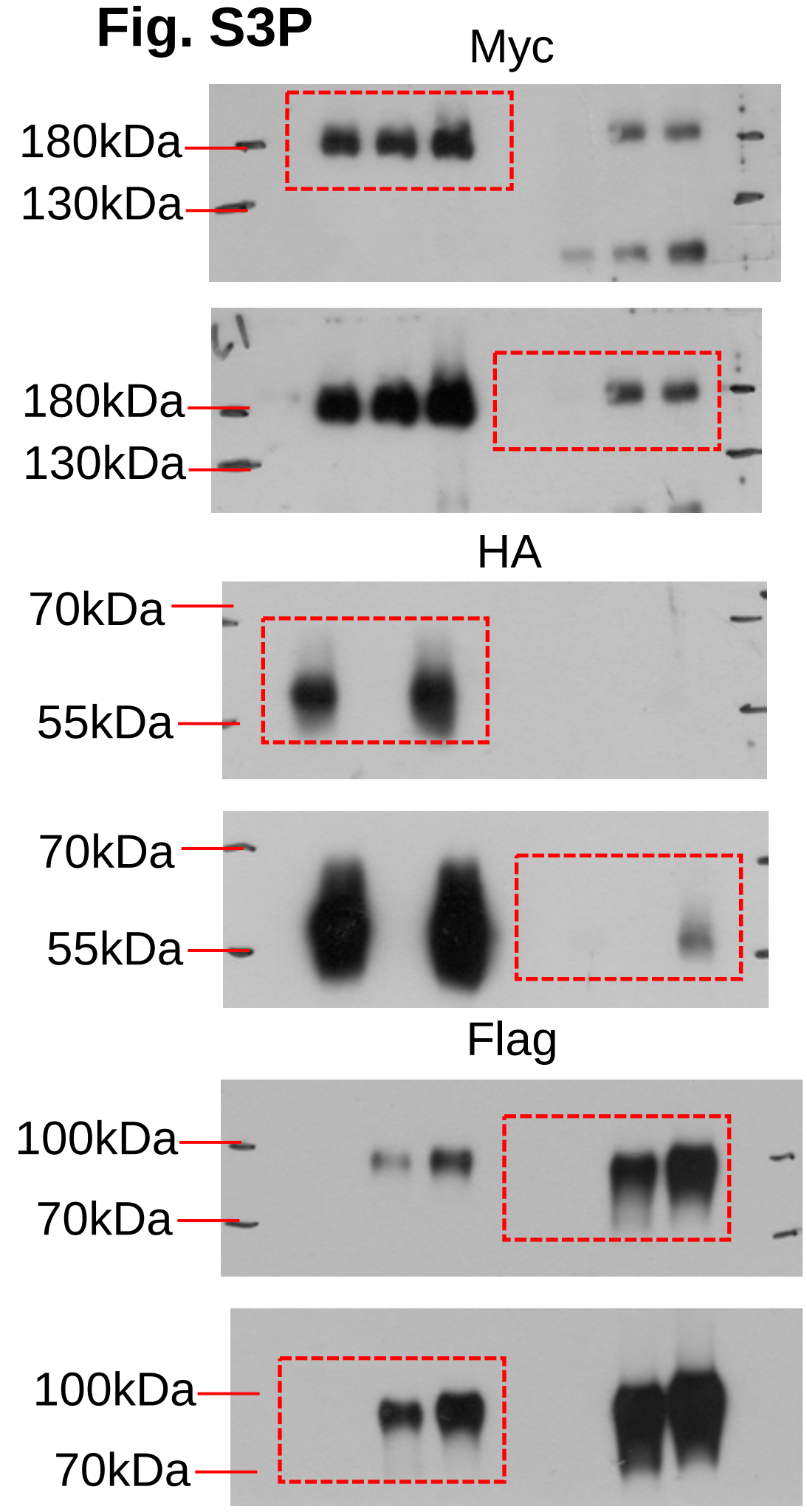

Fig. S3Q

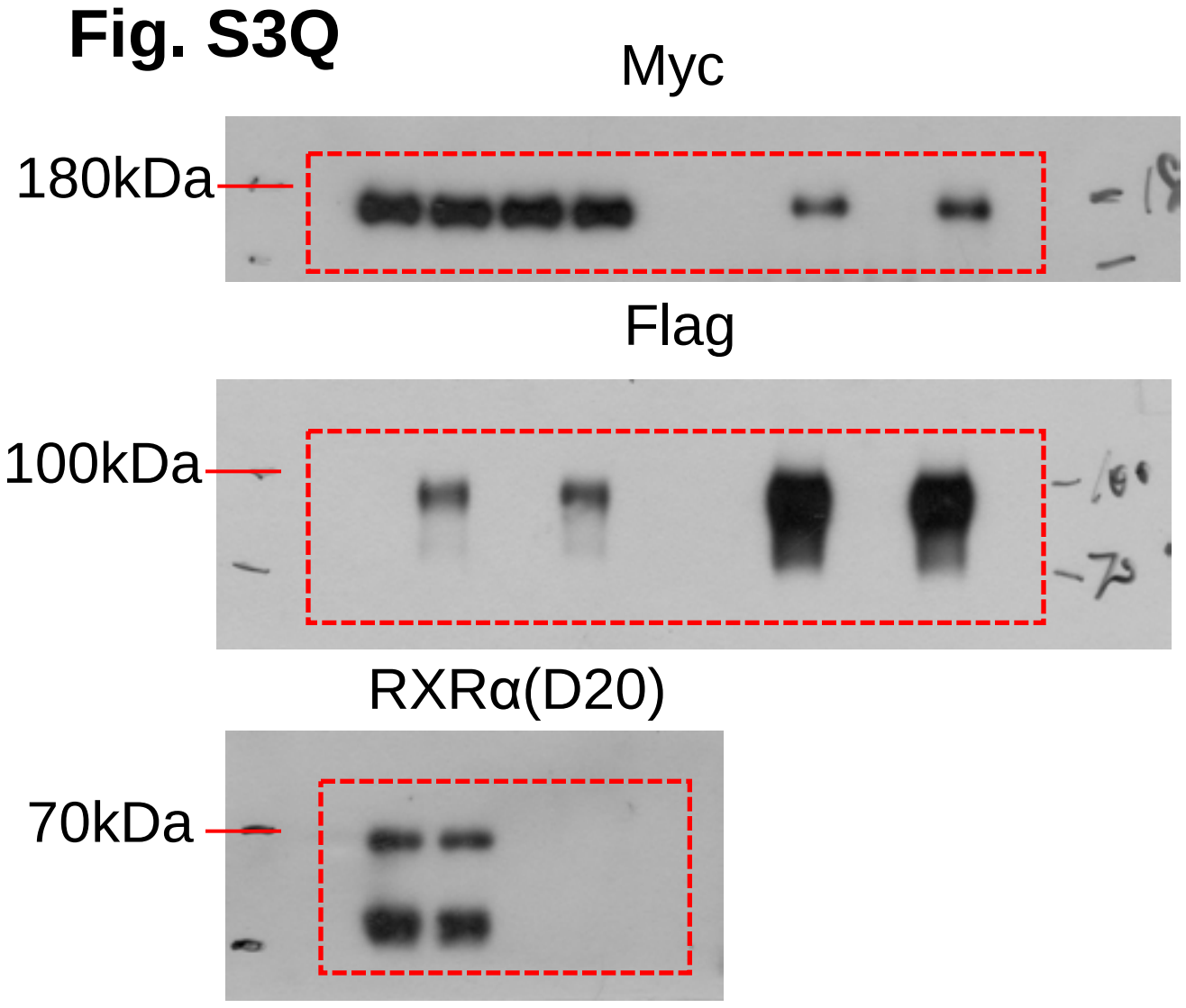

Fig. S3T

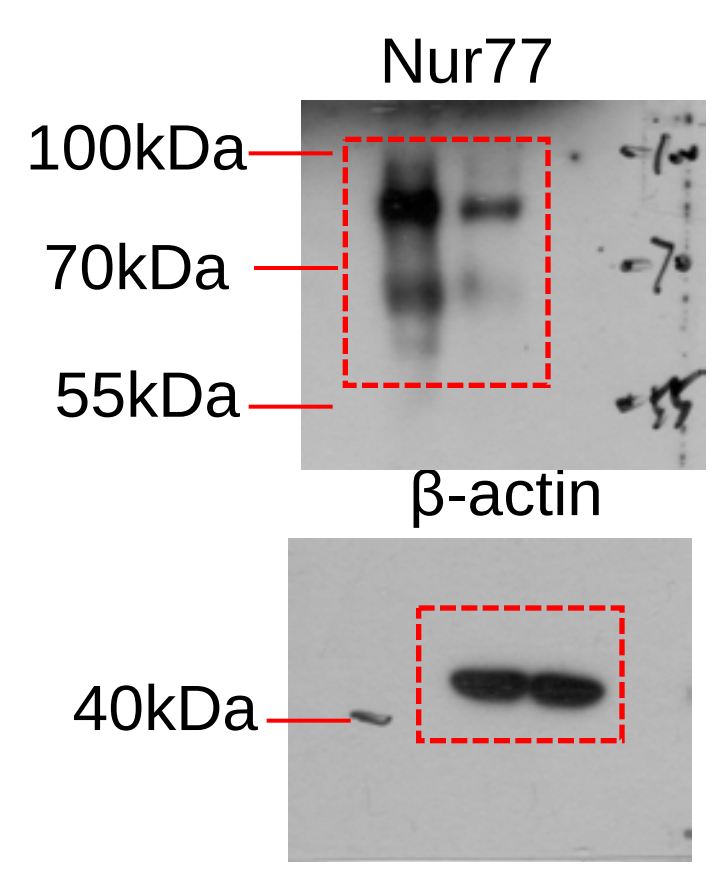

Fig.S3V

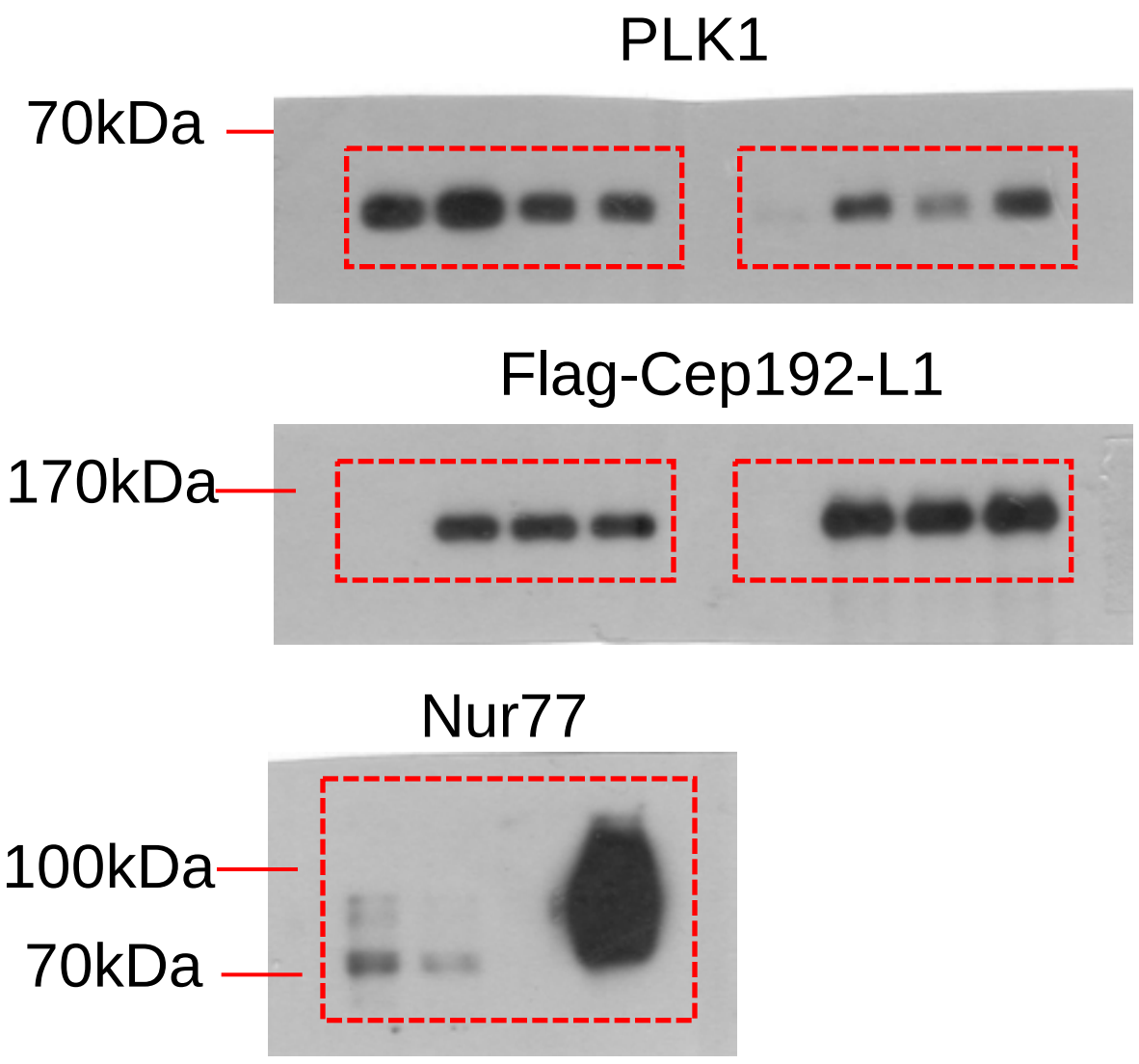

Fig.S4A

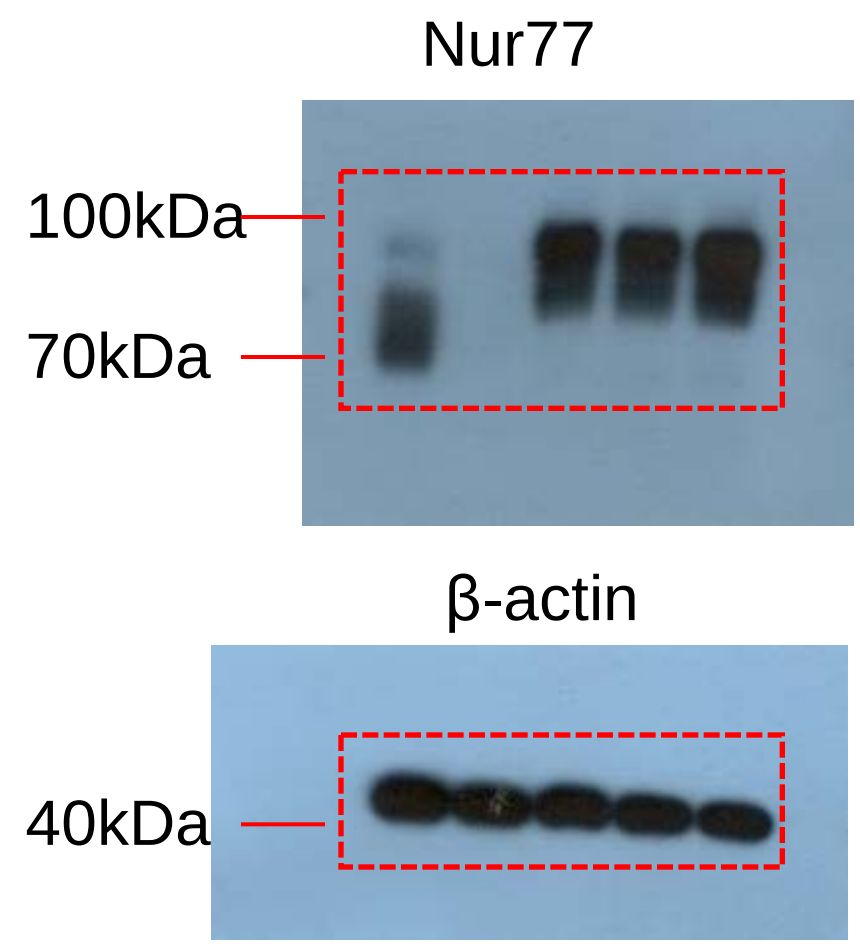

Fig.S4C

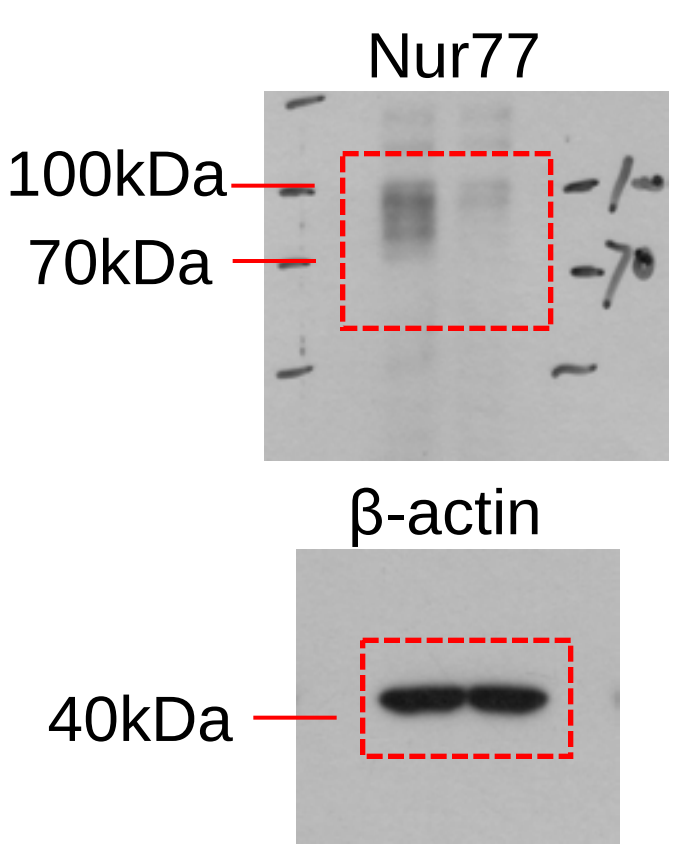

Fig.S4J

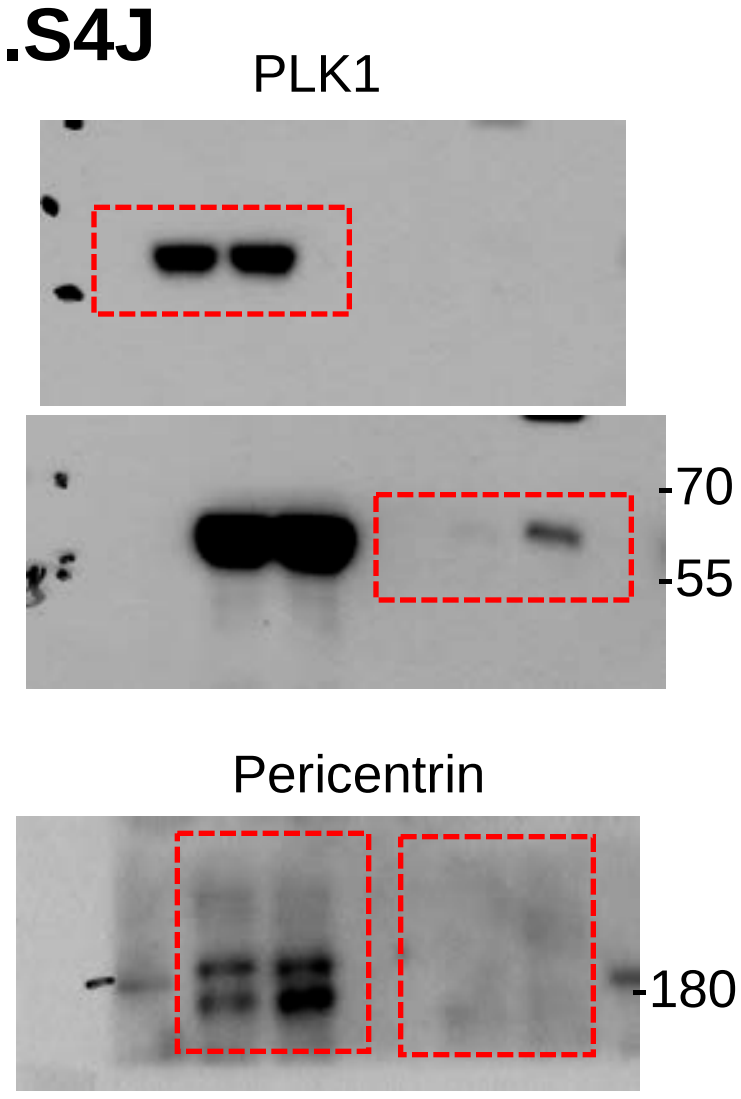

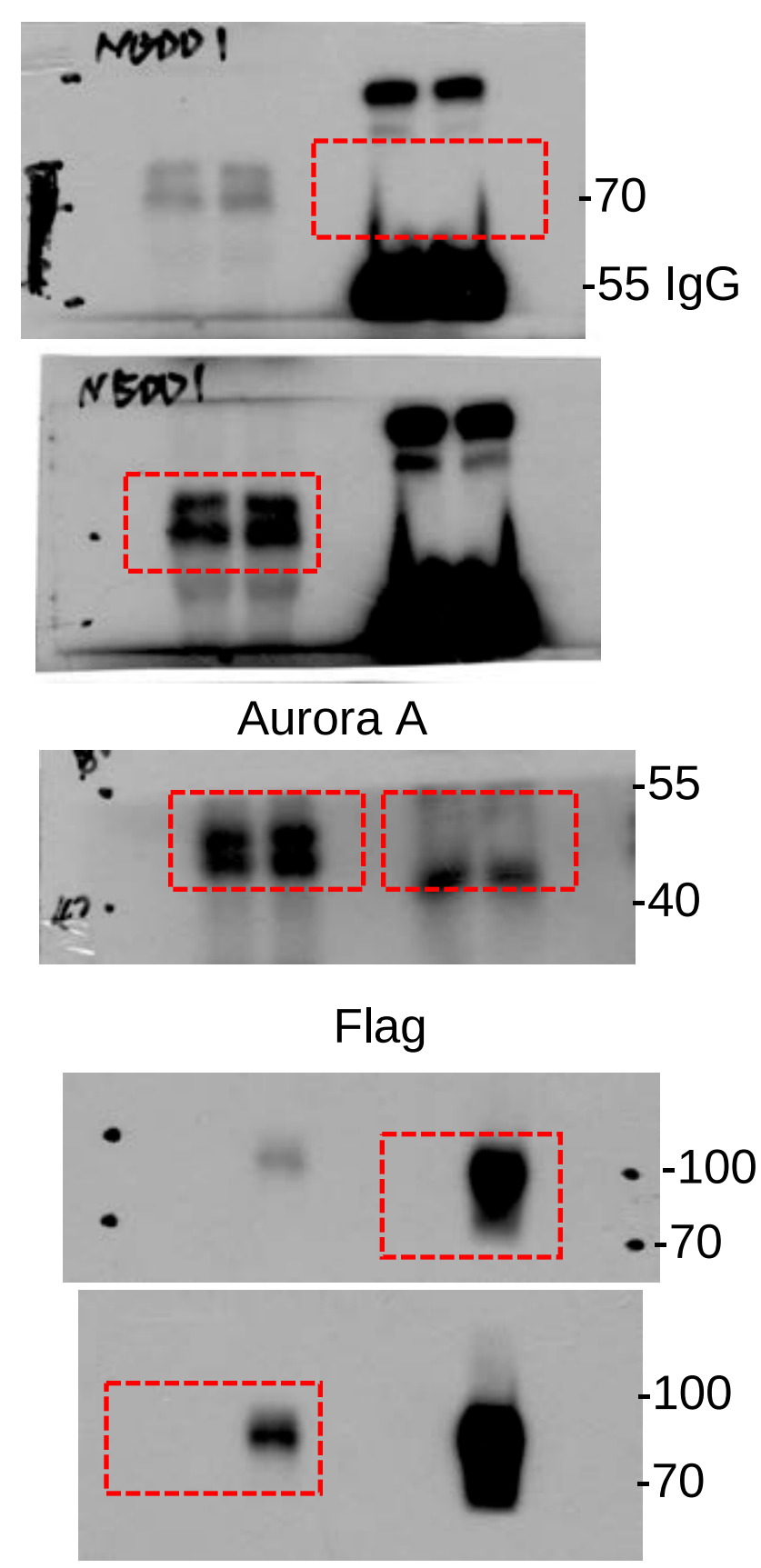

Fig.S4K

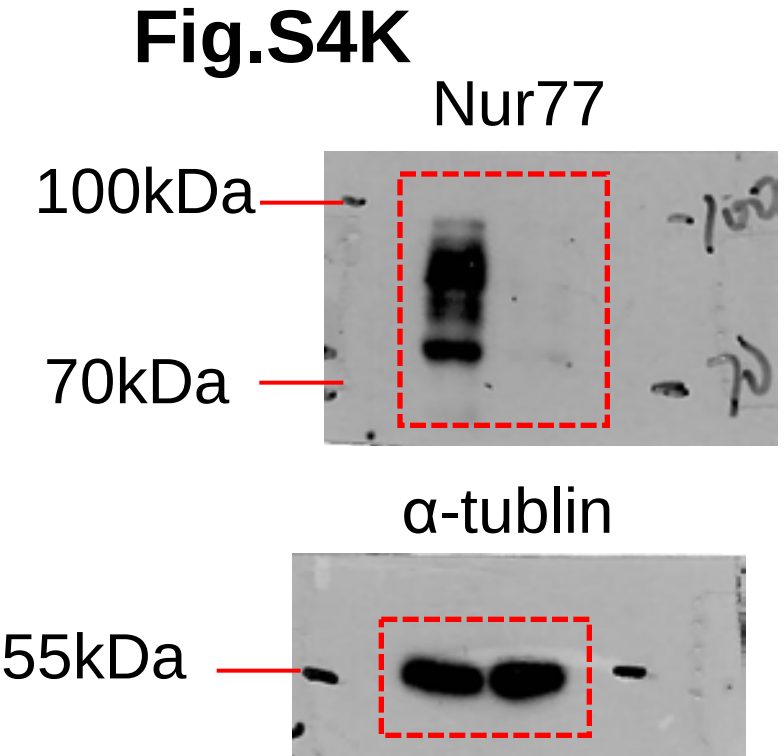

Fig.S4L

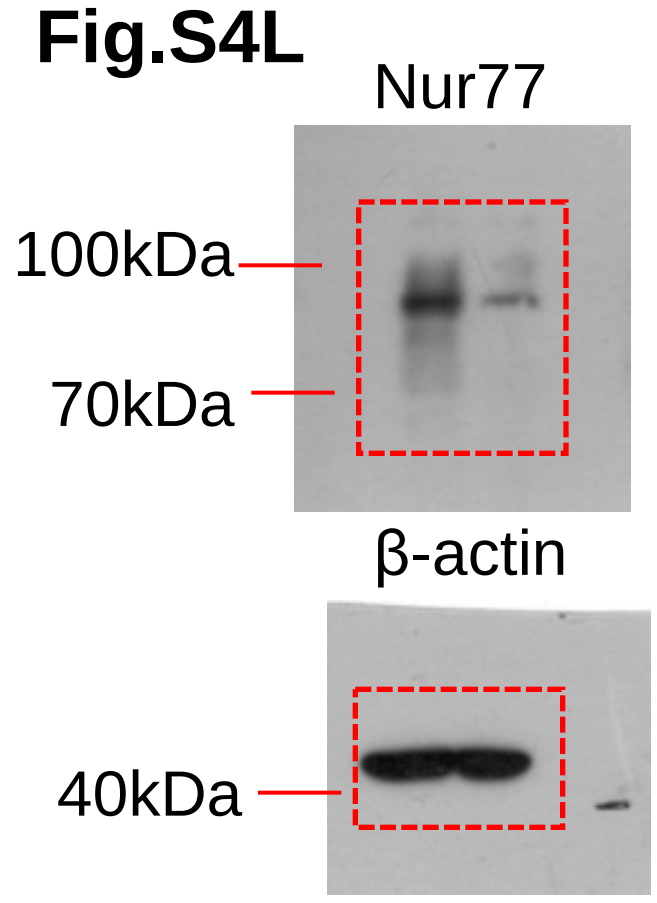

Fig. S4R

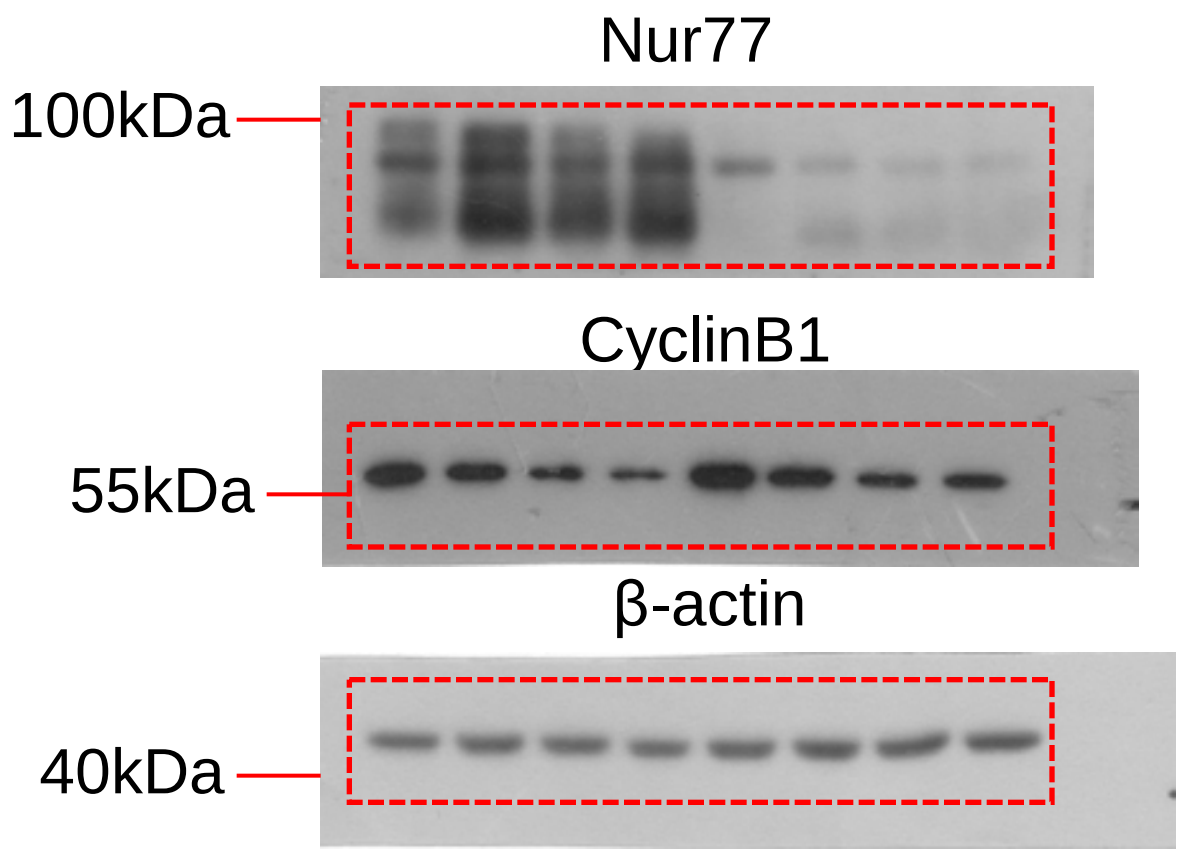

Fig. S4T

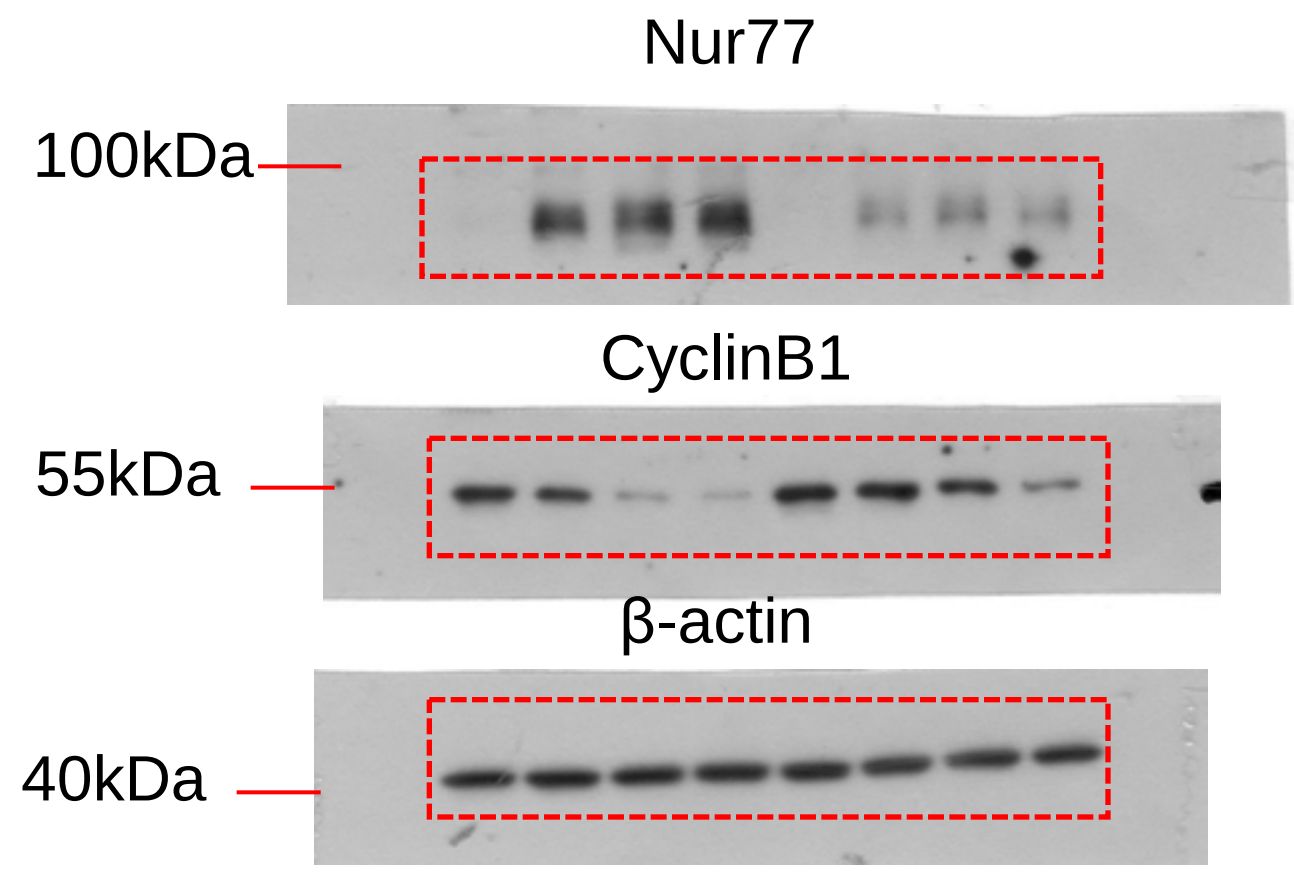

Fig. S4V

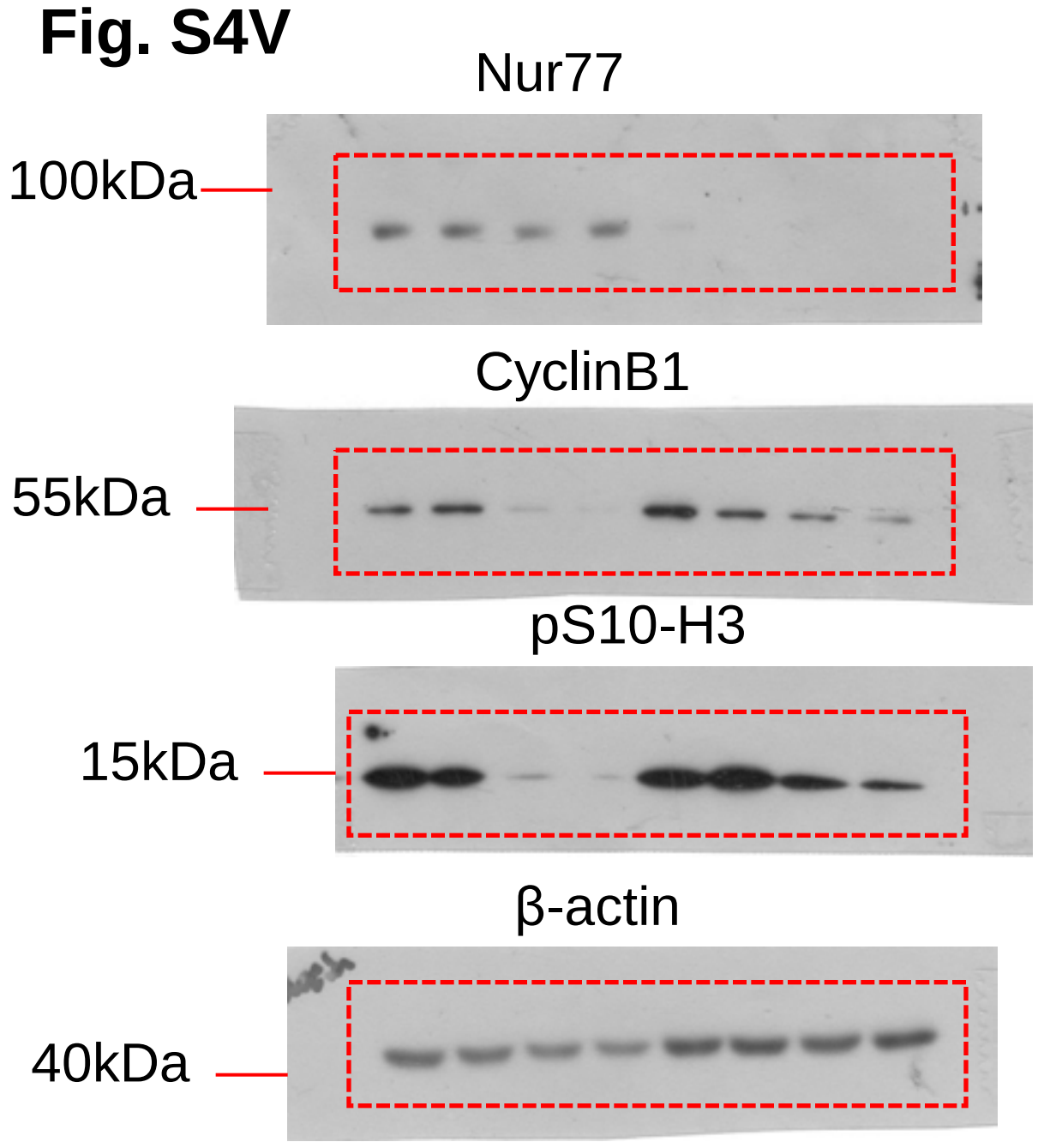

Fig. S4X

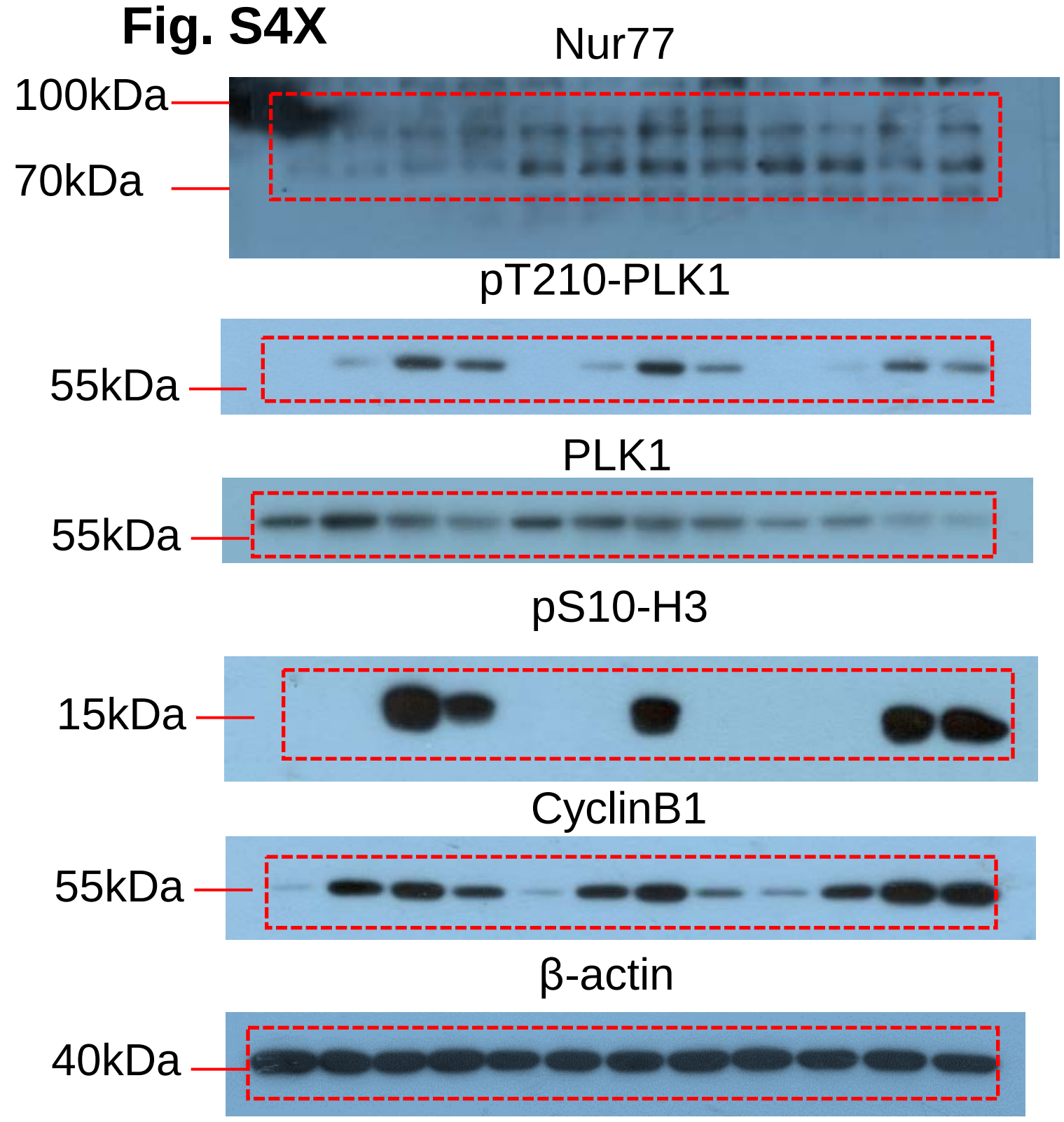

Fig.S5A

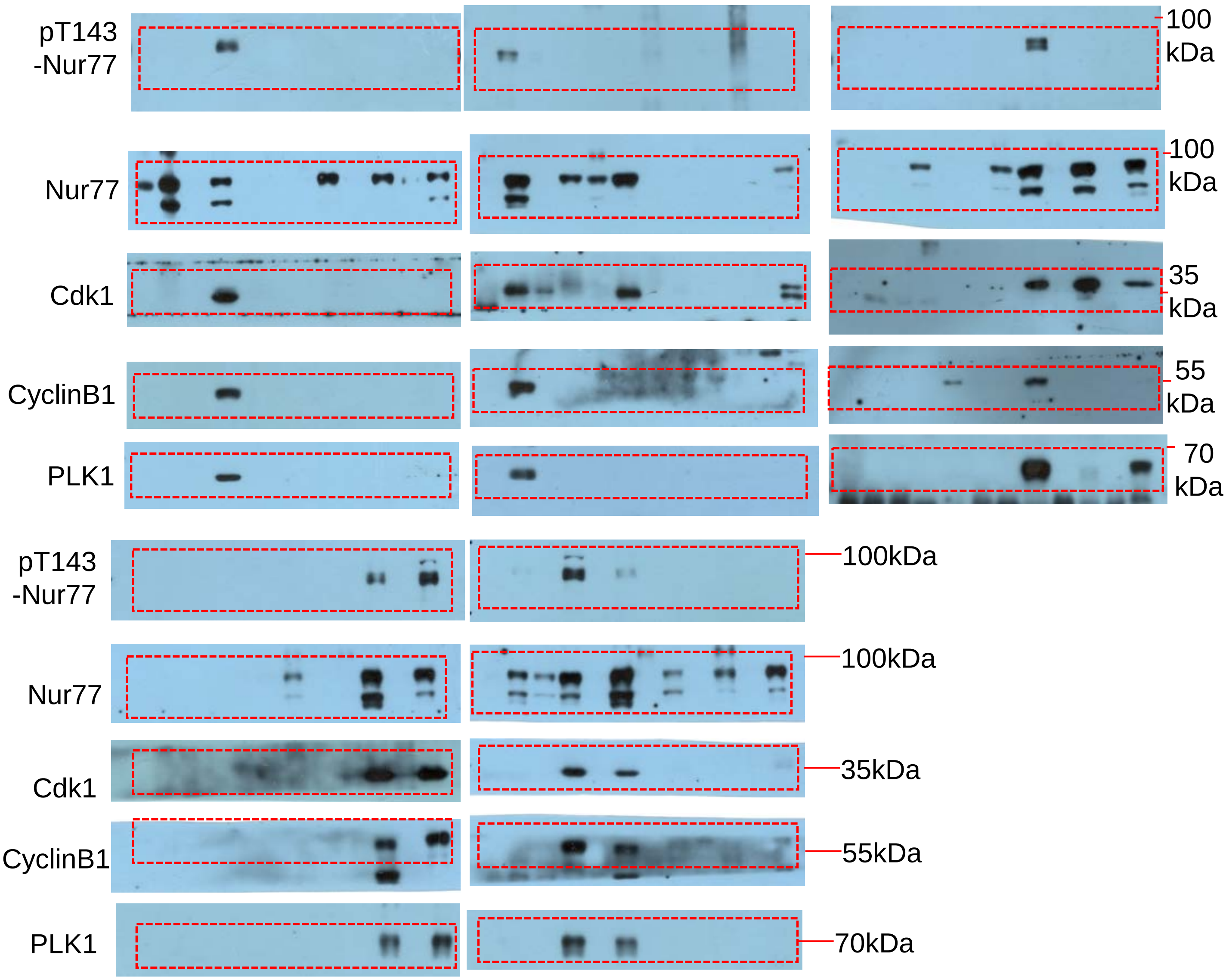

Fig. S5J

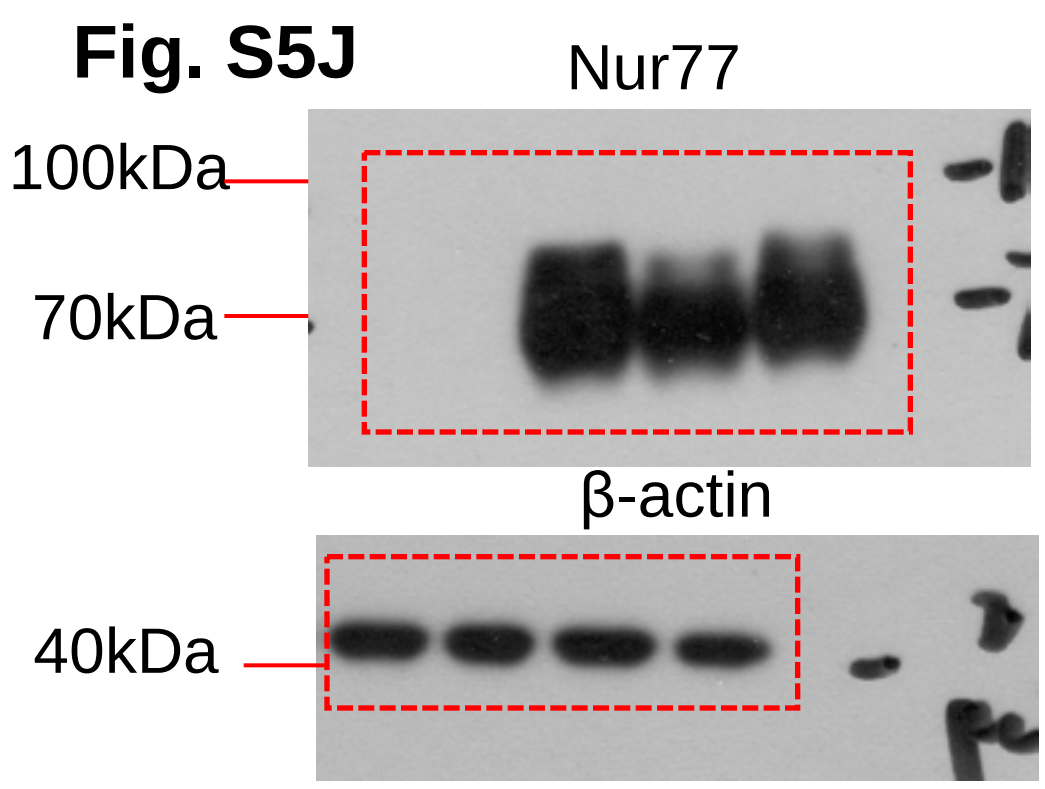

Fig. S6F

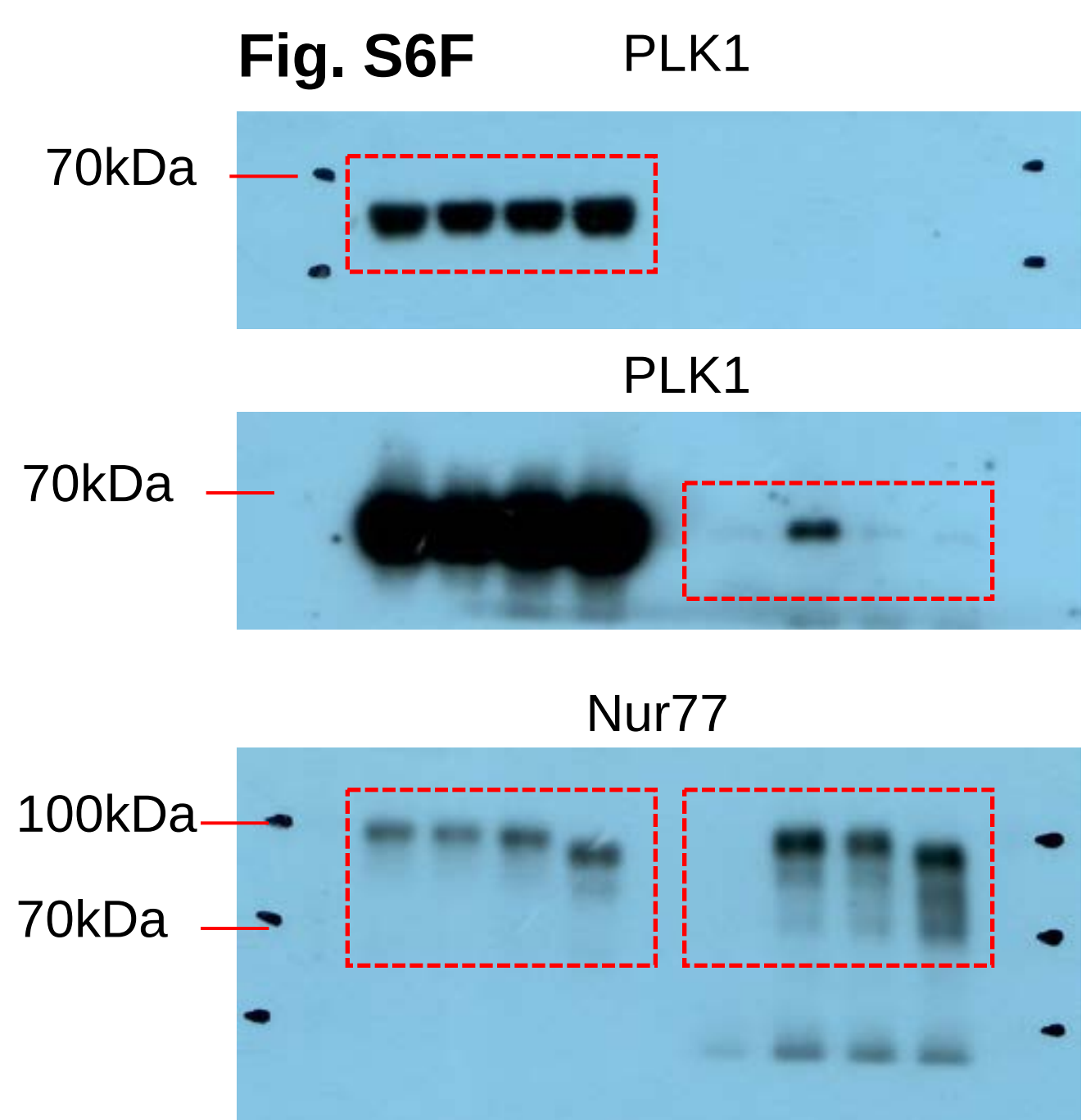

Fig. S6A

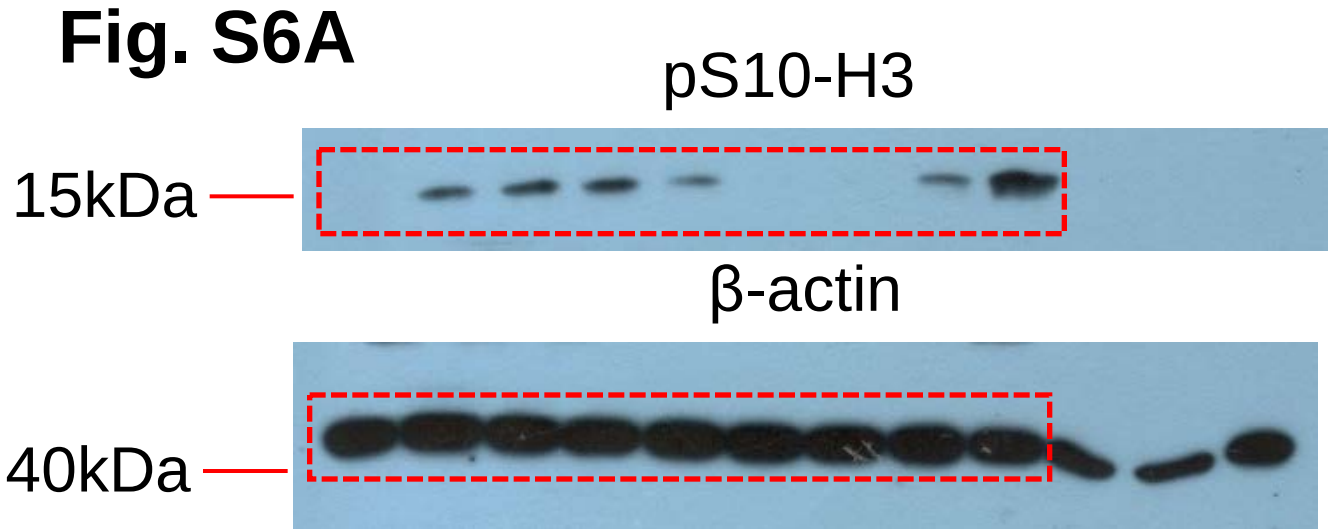

Fig. S6K

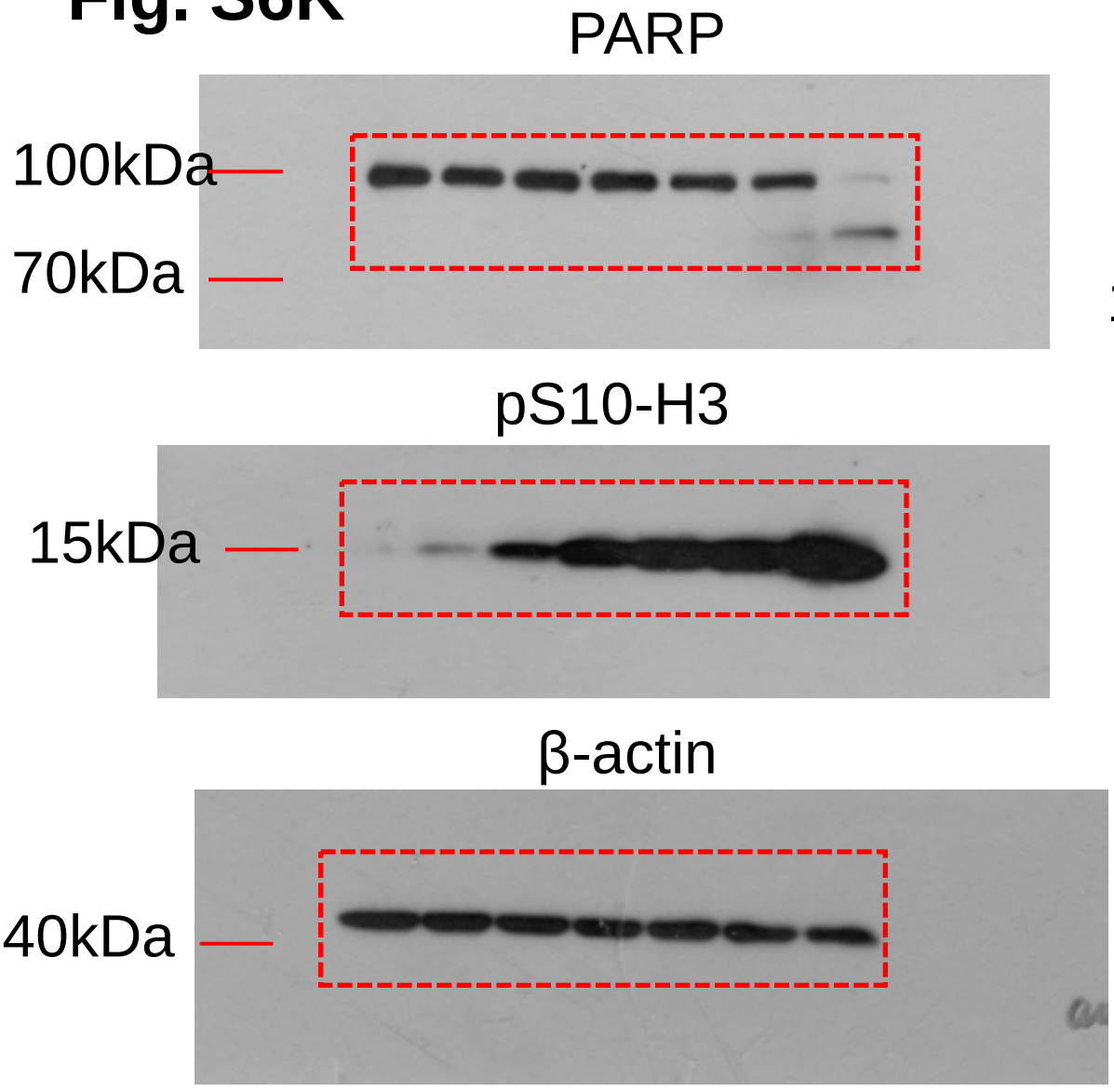

Fig. S6M

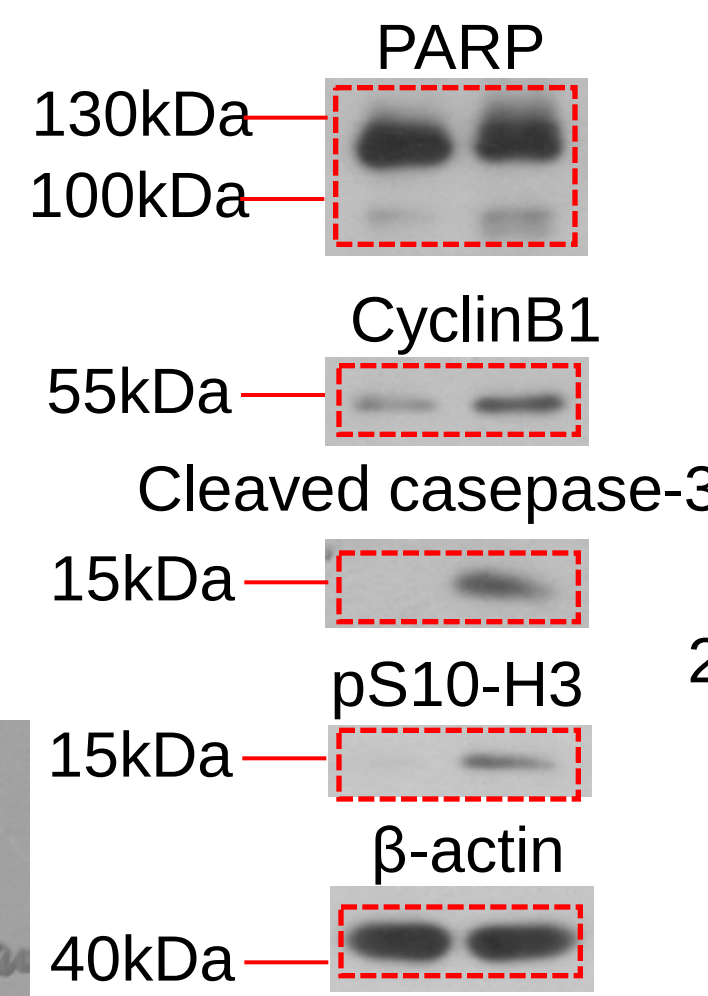

Fig. S6O

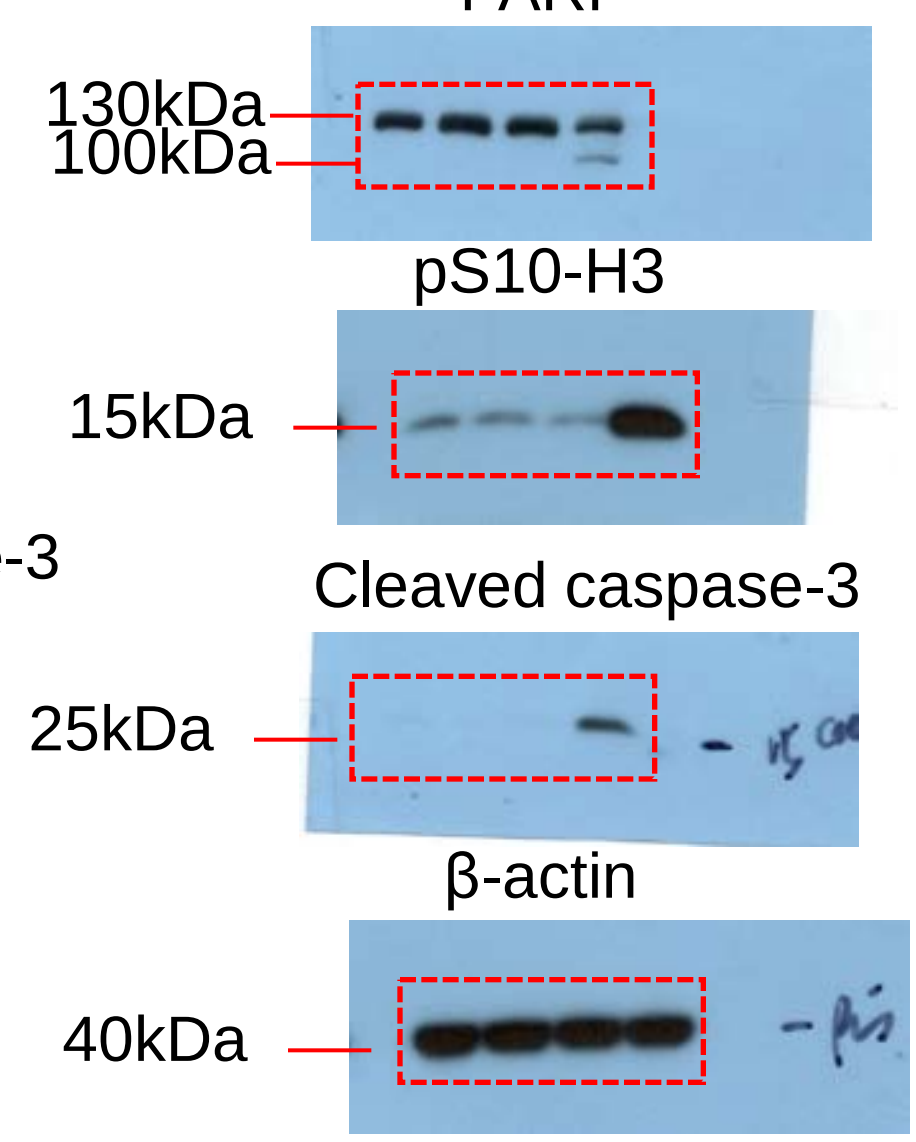

Fig. S6L

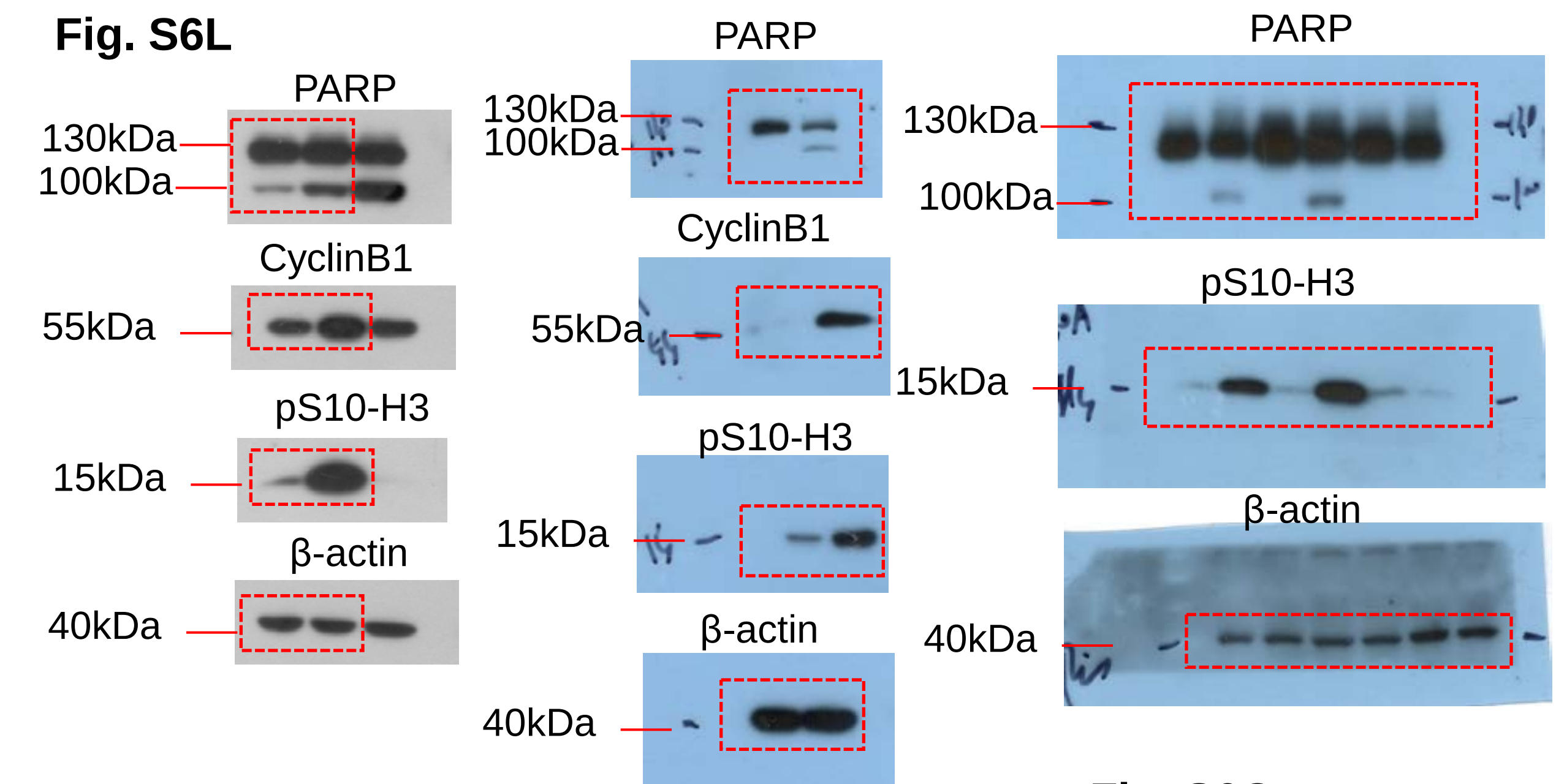

Fig. S6Q

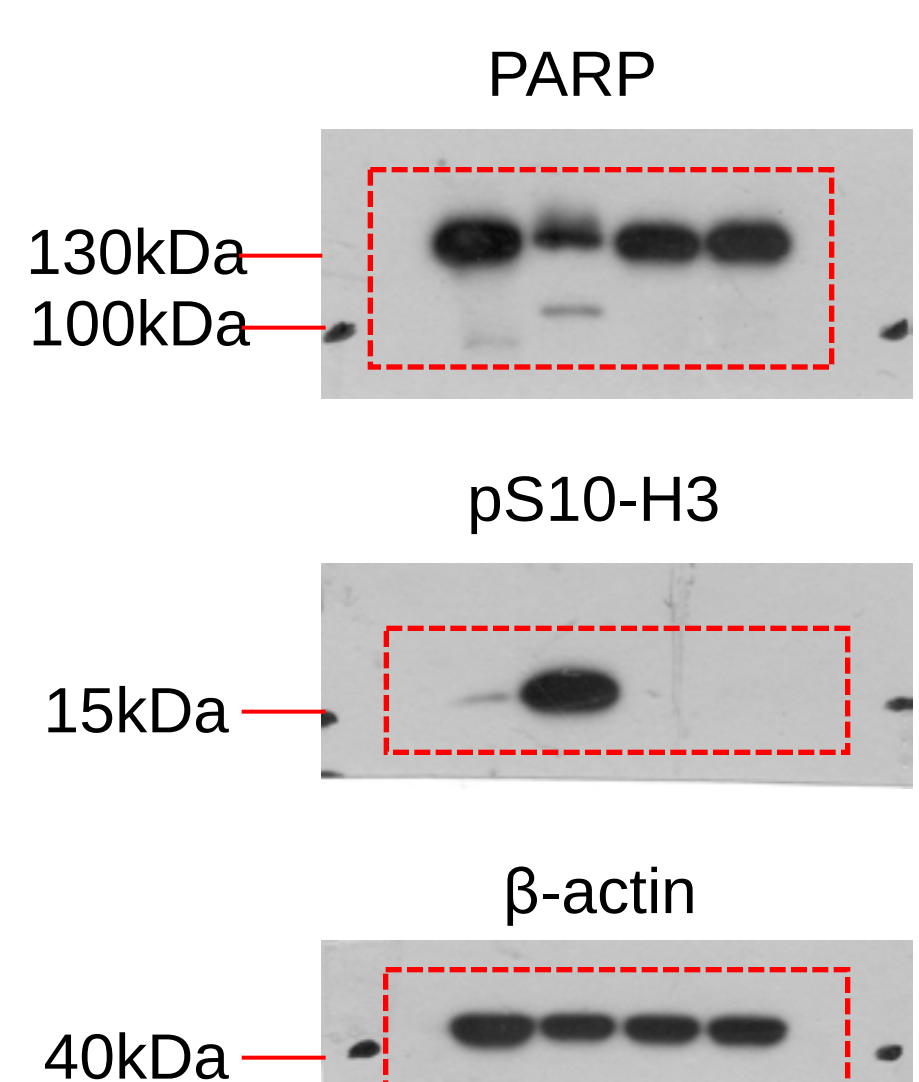

Fig. S6P

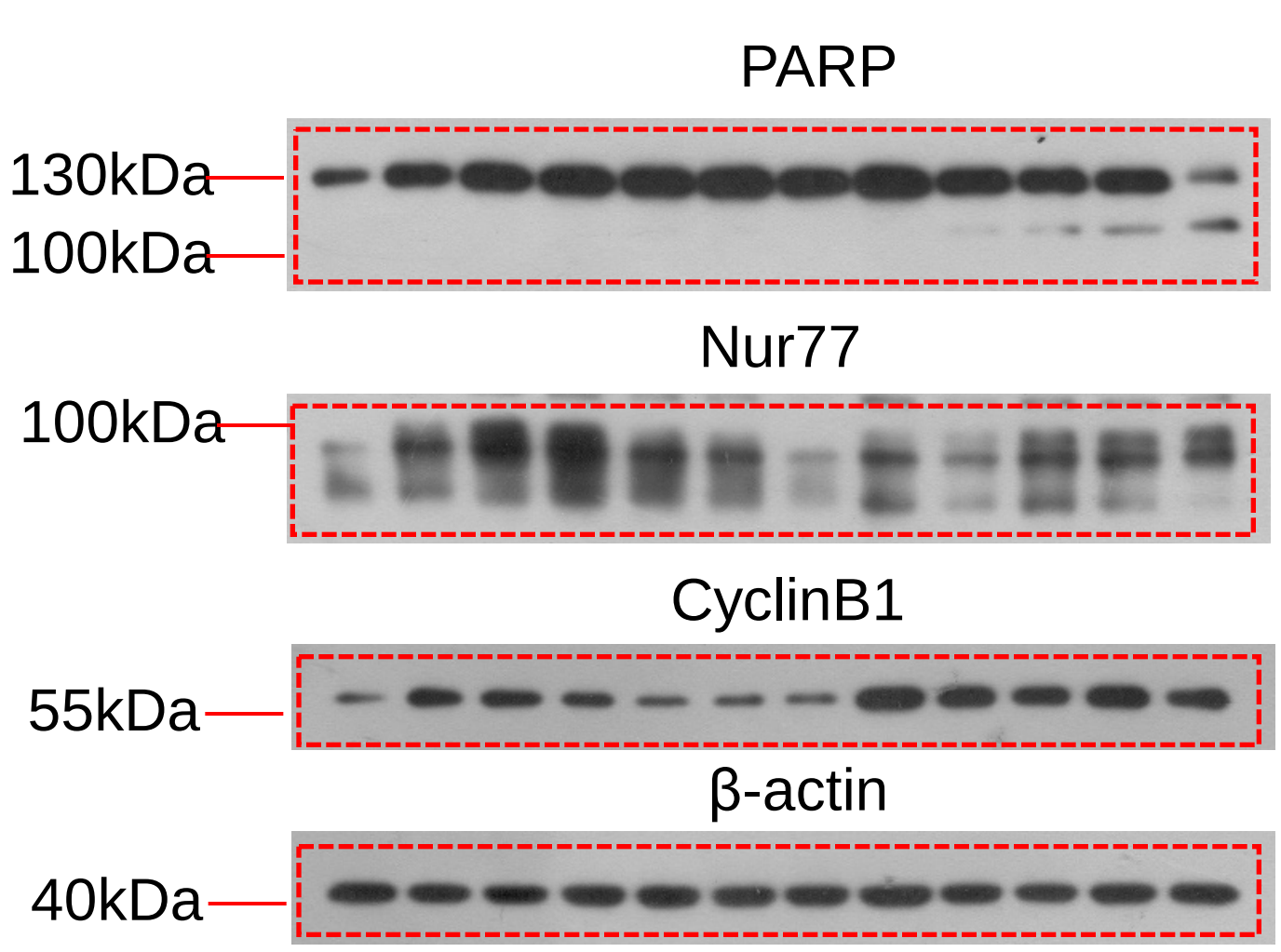

Supplement: Supplementary file 2 [file 41419_2026_8870_MOESM3_ESM.pdf]
