# Supplementary material for: Cdk1-phosphorylated Nur77 accumulates at the centrosome during mitosis to regulate the Cep192–PLK1 signaling axis
Source: Cell Death Dis. 2026 Jun 3;17(1):695. doi: 10.1038/s41419-026-08870-3 (PMC13448657; doi:10.1038/s41419-026-08870-3)
Supplement: Supplementary file 3 [file 41419_2026_8870_MOESM2_ESM.docx]

**Table S1, Correlation between parameters and pT143-Nur77 low or high expression, *related to Fig. 5B and Supplementary Fig. 5A*.**

| **Parameters** | **Category** | **Cases** | **pT143-Nur77 expression**  **Low High** | **p** |
| --- | --- | --- | --- | --- |
| Cdk1 expression | low | 22 | 22 0 | 3.09e-6^a^ |
|  | high | 14 | 4 10 |  |
| CyclinB1 expression | low | 25 | 25 0 | 2.04e-8^a^ |
|  | high | 11 | 1 10 |  |
| PLK1 expression | low  high | 24  12 | 24 0  2 10 | 1.42e-7^a^ |

a, Chi-square test
